# The entorhinal spatial map integrates visual identity information of landmarks

**DOI:** 10.1038/s41467-026-72453-1

**Published:** 2026-05-07

**Authors:** Garret Wang, Farid Shahid, Taylor J. Malone, Jean Tyan, Kyle Cekada, Lujia Chen, Yi Gu

**Affiliations:** 1 Spatial Navigation and Memory Unit, National Institute of Neurological Disorders and Stroke, National Institutes of Health, Bethesda, MD USA; 2 Present Address: Medical Scientist Training Program, Medical College of Wisconsin, Milwaukee, WI USA; 3 Present Address: Division of Clinical Geriatrics, Center for Alzheimer Research, Department of Neurobiology, Care Sciences and Society, Karolinska Institutet, Stockholm, Sweden; 4 Present Address: Department of Psychological and Brain Sciences, University of California, Santa Barbara, Santa Barbara, CA USA

**Keywords:** Perception, Cognitive control

## Abstract

Landmarks guide navigation by providing information through their location and identity. The medial entorhinal cortex (MEC) is well known for representing landmark location, but whether it also encodes landmark identity remains unclear. Here we show, using two-photon calcium imaging of MEC neurons in mice navigating multiple virtual environments, that a population of neurons known as cue cells encodes landmark identity. Cue cells respond selectively to individual landmarks and produce more distinct activity patterns for visually disparate landmarks than for identical ones. Identity encoding is modulated by the spatial shift of cue cell activity relative to landmark location and is context dependent, changing across environments, but remaining stable within the same environment despite repeated experience. In contrast, cue cells’ representation of landmark location changes with experience. Grid cells, another major MEC cell type, more strongly represent landmark location, but only weakly encode identity. These findings suggest that the MEC integrates both the location and identity of landmarks to support navigation.

## Abstract (web summary)

It remains unclear whether and how the MEC (medial entorhinal cortex) encodes landmark identity. Here authors show that the MEC integrates visual landmark identity and location. Cue cells encode both, with identity encoding modulated by spatial activity shifts and context but independent of experience, while grid cells primarily encode location.

## Introduction

Landmarks play a critical role in spatial navigation by providing both spatial and nonspatial cues^1^. The location of landmarks conveys spatial information about the environment, while the identity of landmarks, such as whether two landmarks are visually similar or distinct, provides nonspatial information. Despite this distinction, it remains unclear how spatial and nonspatial attributes of landmarks are integrated within a unified cognitive map.

To address this question, we focus on the medial entorhinal cortex (MEC), a brain region critical for spatial information encoding^2^. The MEC contains a diverse population of spatially modulated neurons, including grid cells, which exhibit a characteristic triangular firing pattern in open arenas^3^, as well as cells encoding head direction^4^ and environmental boundaries^5^. These cells are hypothesized to form a cognitive map that supports spatial navigation^6^.

However, growing evidence shows that the MEC encodes both spatial and nonspatial information^7^. MEC dysfunctions or lesions impair not only spatial navigation and memory^8–12^, but also item recognition^13,14^, implicating its role in nonspatial processing as well. Immediate early gene expression study indicates that the MEC is similarly active during both spatial and nonspatial tasks^15^. Additionally, MEC neurons encode task variables in auditory^16^ and time-interval tasks^17,18^ even  when physical locations of the animals remain constant. The MEC also adjusts its response when environmental features (shape or color) are altered^19^, when spatial information is represented through different sensory modalities^20^, or when reward signals are associated with cues of varying identities^21^. Collectively, the nonspatial information encoding by the MEC suggests that it plays a role in integrating both spatial and nonspatial information for navigation.

Yet, in landmark-rich environments, it remains unclear whether and how the MEC encodes landmark identity. Notably, the MEC harbors object-vector cells and cue cells, which respond specifically to objects/visual landmarks during navigation in real^22^ and virtual environments^23^, respectively. These cells are thought to provide vectorial representation of an animal’s location relative to landmarks, because they are active at consistent distances and directions from all salient objects, regardless of their identity^22,23^. However, whether the activity of these cells varies according to landmark identity has not been systematically investigated. Thus, it remains an open question whether MEC neurons can also encode landmark identity during spatial navigation.

**Fig. 1:**
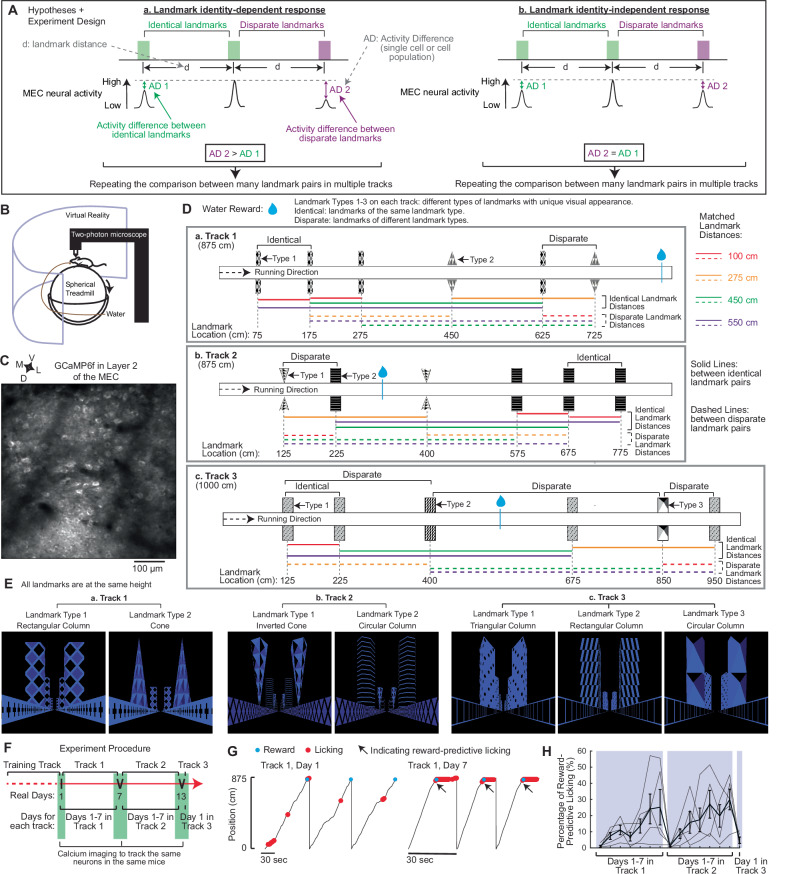
Experimental design. **A** Hypotheses about the encoding of landmark identity. Green and magenta represent comparisons between landmarks of identical and disparate visual identity, respectively. **B** Experimental setup. **C** Example two-photon field of view (FOV) showing excitatory neurons in layer 2 of the MEC expressing GCaMP6f. V: ventral, D: dorsal, M: medial, L: lateral. **D** The design of tracks 1-3 with four matched landmark distances: 100 cm (red), 275 cm (orange), 450 cm (green), and 550 cm (purple). **E** Example views of tracks 1-3 from a mouse’s perspective, showing different types of landmarks. **F** Experiment procedure. Note that on real days 7 and 13, the mouse performed in two sessions, which are considered as day 7 of track 1 and day 1 of track 2 (real day 7) or day 7 of track 2 and day 1 of track 3 (real day 13). Track switch days are indicated in green. **G** Behavioral examples mouse licking (red) behavior in track on days 1 and 7 1. Black arrows indicate the occurrence of predictive licking before the reward (blue). **H** The percentage of reward-predictive licking in tracks 1-3. Translucent blue blocks highlight the days in the same track. The percentage was calculated as the percentage of licking events right before the reward location among those in the whole track, excluding the licking for reward consumption. Data were from 5 mice, shown as individual light gray curves. Data are presented as mean ± standard error of the mean (SEM).

To directly investigate the MEC encoding of landmark identity during navigation, we measured MEC calcium dynamics, which approximated its neural activity, as mice navigated in multiple virtual reality (VR) linear tracks containing visual landmarks with identical and disparate appearances, which corresponded to the same and different identity, respectively. We hypothesized that if MEC neurons encode landmark visual identity, they should exhibit a larger activity difference between disparate compared to identical landmarks (Fig. 1A, a). Otherwise, the differences between these landmarks should be comparable (Fig. 1A, b). Since the activity difference could be confounded by the encoding of landmark distance and location, we eliminated the effect of landmark distance by specifically comparing the activity difference between identical and disparate landmark pairs at matched distances. We further eliminated the effect of landmark location by repeating the comparison across multiple tracks with many landmark pairs at various locations. Thus, we uncovered the encoding of landmark identity by cue cells, which exhibited greater activity difference between disparate landmarks than identical ones. The encoding was modulated by the spatial shift of cue cell activity relative to landmark location and by track identity, but was not influenced by increased track experience. However, the encoding of landmark location by cue cells was regulated by experience. In contrast to cue cells, grid cells weakly represented landmark identity but more robustly encoded landmark location. Together, these findings demonstrate that during navigation, the MEC integrates both spatial and nonspatial information into its construction of the spatial map.

## Results

### Experiment design for investigating the encoding of landmark identity

We conducted cellular-resolution two-photon calcium imaging of the MEC in head-fixed mice while they unidirectionally navigated linear VR tracks for multiple runs via teleportation at the end of the tracks^24^ (Fig. 1B). GP5.3 transgenic mice were used to enable the imaging of stably expressed fluorescent calcium indicator GCaMP6f^25^ in MEC layer 2 excitatory neurons, which are comprised of many functional cell types for navigation, including grid cells and cue cells^23,26^ (Fig. 1C).

As proposed in Fig. 1A, we designed three VR tracks with visual landmarks symmetrically arranged on both sides of each track (Fig. 1D). Track 1 was 875 cm long and contained two sets of landmarks. These had identical appearances (i.e., shapes and surface patterns) within the same set (identical landmarks) and distinct appearances across sets (disparate landmarks) (Fig. 1D, E, a). The landmarks were repeated at multiple locations such that there were four matched distances (100, 275, 450, and 550 cm) for both the identical and disparate pairs. The four distances were also preserved for different sets of identical and disparate landmarks at distinct locations in tracks 2 (875 cm) and 3 (1000 cm) (Fig. 1D, E, b and c). Tracks 1-3 also had different wall patterns and reward locations. Thus, tracks 1-3 together enabled a study of the general ability of the MEC to encode landmark identity (identical versus disparate landmarks), independent of landmark distance, location, and other track features.

After the initial acclimation to VR navigation in a training track, water-restricted GP5.3 mice explored tracks 1-3, where they received a water reward upon reaching a specific location in each track. The mice were exposed to tracks 1 and 2 for seven days per track and to track 3 for one day (Fig. 1F). During the seven days in tracks 1 and 2, the mice gradually improved their specificity to predictively lick right before the reward, indicating their learning of the tracks^27,28^ (Fig. 1G, H). Calcium imaging was conducted on days 1 and 7 in tracks 1 and 2 as well as on day 1 in track 3. The same layer 2 excitatory neurons in the MEC of each mouse were tracked across the five sessions.

Overall, the above setting allowed us to investigate the MEC’s encoding of landmark identity in multiple tracks (tracks 1-3) and during learning in the same track (tracks 1 and 2).

### Encoding of landmark identity by cue cells at the individual-cell level

**Fig. 2:**
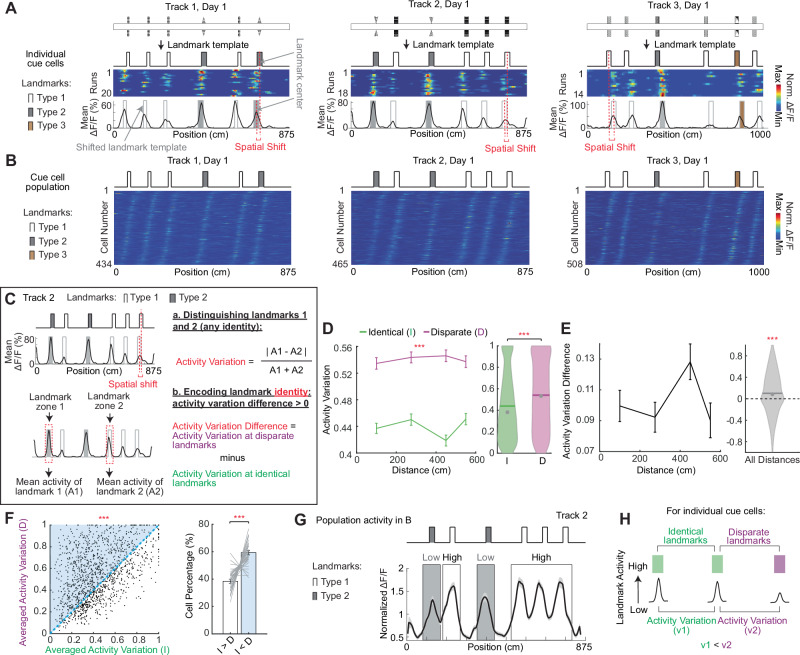
Individual cue cells encode landmark identity. **A** Cue cell examples in individual tracks. For each track: from top to bottom: real track; simplified landmark template with different landmark types (marked in white, gray, or brown); run-by-run calcium dynamics (hotter colors indicate higher normalized activity); mean ΔF/F (spatially binned) along the track. The landmark template is aligned to the mean ΔF/F under a spatial shift (the distance between red dashed lines). **B** Cue cells in individual tracks sorted by their spatial shifts. **C** Descriptions of activity variation and activity variation difference calculations. **D** Activity variation for identical (I, green) and disparate (D, magenta) landmark pairs pooled from tracks 1-3 at individual (left) and combined (right) matched distances. The asterisks in the left panel indicate the result of a two-way ANOVA for I and D curves (1407 cells × 4 distances, *p* = 3.53e-31, F = 138). Those in the right panel indicate the result of a two-tailed paired t-tests (*p* = 3.81e-79, *n* = 5628 activity variations for I and D). **E** Activity variation difference pooled from all three tracks at individual (left) and combined distances (right). The asterisks in the right panel indicate the result of a two-tailed paired t-test comparison with zero (*p* = 3.81e-79, *n* = 5628 activity variation differences). **F** For all three tracks combined, left: averaged activity variations for identical landmark pairs (I) versus those for disparate pairs (D) in individual cells. Asterisks represent the *p*-value of the two-tailed Pearson’s linear correlation of the averaged activity variation coordinates for all cells (r = 0.499, *p* = 5.50e-89, *n* = 1407 cells). Blue and white zones include cells with larger variations for disparate and identical landmark pairs, respectively. Right: the percentage of cells per FOV with larger activity variations for identical (white) or disparate (blue) landmark pairs. Asterisks represent the result of a two-tailed paired t-test (*p* = 2.99e-07, *n* = 30 FOVs). **G** Averaged normalized activity of cue cells reflects an amplitude-based landmark identity encoding. **H** A summary for activity variation of individual cells at identical and disparate landmark pairs. Data were from 5 mice, including 434, 465, 508 cells aggregated from tracks 1, 2, and 3, respectively. *p <  0.05, **p <  0.01, ***p  < 0.001, n.s. *p* ≥ 0.05. Violin plots have dot and horizontal bar representing median and mean, respectively. Same for all other figures. All other data are presented as mean ± SEM.

We first asked whether the MEC neural response differentiated identical and disparate landmarks on day 1 in tracks 1-3. We focused on cue cells as they have highly specific activity toward landmarks. Cue cells were identified in each track based on cue scores, which quantified their landmark-dependent activation^23^. Approximately 20% of active cells were identified as cue cells (track 1: 16.5% ± 1.9%; track 2: 22.0% ± 2.0%; track 3: 22.1% ± 2.1%), defined as those with cue scores above the 95^th^ percentile of the shuffled distribution. As previously reported^23^, each cue cell exhibited a characteristic spatial shift, reflecting the distance and direction (before, at, or after) of cue cell activity relative to landmarks (Fig. 2A). The activity and landmark template, which represented the pattern of landmark arrangement, were best matched after shifting the template according to the spatial shift. Sorted activities of cue cells by their shifts formed consistent sequences around individual landmarks^23^ (Fig. 2B).

We examined the encoding of landmark identity at the individual-cell level by evaluating activity variation of each cue cell between landmark pairs (Fig. 2C). By aligning the activity of each cell based on its spatial shift with the landmark template, we identified its activity zone around each landmark (landmark zone). Its landmark activity was the mean activity in each landmark zone and its activity variation between a landmark pair was the absolute difference over the sum of its landmark activities at the two landmarks (Fig. 2C, a). Large and small activity variations indicate stronger and weaker landmark discrimination, respectively. If a cue cell encodes landmark identity, it should exhibit larger activity variation between disparate compared to identical landmarks. Consequently, its activity variation difference, which is its activity variation between disparate landmarks minus that between identical ones at matched distances, should be above zero (Fig. 2C, b).

We observed that cue cell activity varied more between different landmarks than identical ones, both at individual and combined distances, whether analyzed across all three tracks (Fig. 2D), or for individual tracks (Fig. S1A). This result suggests that variation in the activity of cue cells at different landmark types represents identity encoding. Additionally, there was no consistent relationship between the variation and landmark distance for either identical or disparate pairs (Fig. 2D and Fig. S1A, the trends of magenta and green curves), indicating that the variation does not represent landmark distance. As expected, activity variation difference was above zero (Fig. 2E, right) and did not change consistently with landmark distance (Fig. 2E and Fig. S1B), suggesting that landmark identity encoding is distance independent. The above results remained true after grouping the cells by imaging FOV (Fig. S2A, B) and by mouse (Fig. S2C, D), confirming that the results were not dominated by cells in particular FOVs or mice despite the differences in cue cell numbers (Fig. S2E, F).

We further evaluated landmark identity encoding in each cue cell by comparing its averaged activity variations across all matched distances for both identical and disparate landmark pairs. The averaged variations for the two types of landmark pairs were positively correlated (Fig. 2F, left) and more cells showed greater variations for disparate pairs (Fig. 2F, right). This finding remained when considering each track separately, as well (Fig. S3A). Therefore, while cue cells have a general ability to distinguish landmarks, more cells better discriminated disparate landmarks. Finally, activity variation differences between disparate and identical landmarks could also be directly visualized in the population activity of cue cells, which exhibited more distinct and similar activity amplitudes between disparate and identical landmarks in each track, respectively (Fig. 2G and Fig. S3B).

While the above results support landmark identity encoding by cue cells at the individual-cell level, it is noteworthy that in all three tracks, the types of individual landmarks differed not only in their visual identity but also in their saliency. For example, type 1 landmarks appeared more frequently than other types (Fig. 1D). To eliminate the effect of landmark frequency-based saliency on identity encoding, we analyzed two tracks (tracks 4 and 5) that featured landmarks with different identities, but equal frequencies (Fig. S4A, B). For both tracks, regardless of whether we included all landmarks or restricted the analysis to landmarks of equal frequencies, cue cells exhibited greater activity variation between disparate landmarks compared to identical ones (Fig. S4C, D). These results indicate that the observed activity variation between different landmark pairs reflects genuine encoding of landmark identity, rather than differences in frequency-based salience.

In addition, we considered whether proximity to the water reward influenced cue cells’ ability to discriminate landmark identity (Fig. S5A) since identical landmark pairs tended to be slightly further from the reward than disparate landmark pairs (Fig. S5B). We found that activity variations for disparate and identical landmarks did not correlate with absolute distance from the water reward (Fig. S5C), nor did the differences between these variations (Fig. S5D). These results suggest that landmark identity encoding by individual cue cells is not modulated by the proximity of landmarks to the reward.

Therefore, individual cue cells better discriminate disparate compared to identical landmarks by exhibiting greater activity variation between the disparate ones (Fig. 2H).

### Encoding of landmark identity by cue cells at the population level

**Fig. 3:**
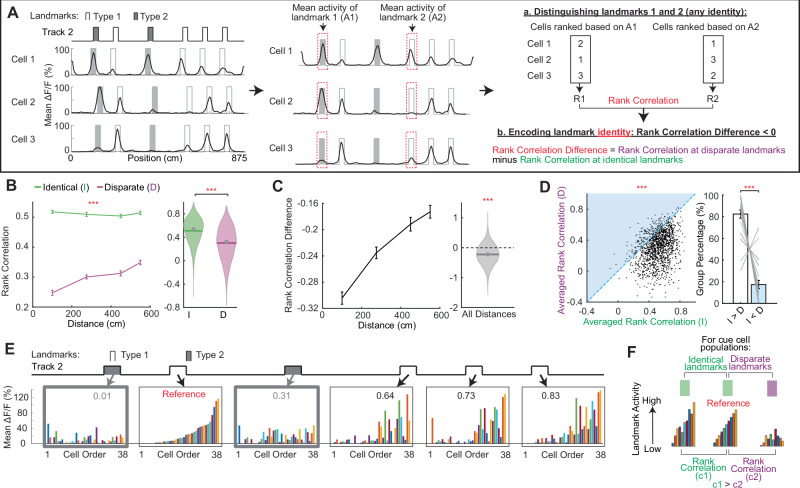
Populations of cue cells encode landmark identity. **A** Descriptions of rank correlation and rank correlation difference calculations. Landmark colored in accordance with their type (white or gray). **B** Averaged rank correlation for identical (I, green) and disparate (D, magenta) landmark pairs pooled from tracks 1-3 at individual (left) and combined (right) distances. Each cue cell group contains 15 cells. The asterisks in the left panel indicate the result of a two-way ANOVA between the I and D curves (1407 cell groups × 4 distances, *p* = 4.61e-144, F = 764). The asterisks in the right panel indicate the result of a two-tailed paired t-test (*n* = 5628 rank correlations for I and D, *p* = 0, as reported by t-test function in MATLAB). **C** Rank correlation differences pooled from all three tracks (*n* = 5628 rank correlations for I and D, *p* = 0, as reported by t-test function in MATLAB). **D** For all tracks combined, Left: averaged rank correlations for identical landmark pairs (I) versus those for disparate pairs (D) for individual cell groups. Asterisks indicate the *p*-value of the two-tailed Pearson’s linear correlation of the averaged rank correlation coordinates for all cell groups (r = 0.31, *p* = 5.09e-32, *n* = 1407 cell groups). Blue and white zones include cell groups with larger rank correlations for disparate and identical landmark pairs, respectively. Right: the percentage of cell groups per FOV with larger rank correlations for identical (white) or disparate (blue) landmark pairs. Asterisks indicate the result of a two-tailed paired t-test (*p* = 1.78e-09, *n* = 30 FOVs). **E** Visualization of the activity of 38 simultaneously imaged cue cells in the same FOV from track 2 at individual landmarks. Cue cells were ranked ascendingly based on their landmark activity at the reference landmark in all cases. Individual cue cells are colored uniquely. Rank correlations for these cells between each landmark and the reference are shown at the top of each landmark box. Relative activities of the cells are more similar for identical landmarks. **F** A summary of rank correlation of cue cell population at identical and disparate landmark pairs. Data were from 5 mice, including 434, 465, and 508 cells aggregated from tracks 1, 2, and 3, respectively. Data are presented as mean ± SEM.

We next examined landmark identity encoding by cue cells at the population level (Fig. 3A). We hypothesized that relative activity levels between simultaneously recorded cue cells serve as a population code for each landmark, and that the code is more different between disparate compared to identical landmarks. To quantify the code, we randomly picked a fixed number of cue cells (referred to as a cue cell group, consisting of 15 cells per group unless otherwise specified) in an FOV and ranked their landmark activities at each landmark. The similarity of the rankings at different landmarks was quantified by a Pearson correlation (Rank Correlation, Fig. 3A, a) of the two ranking vectors. Higher and lower correlations reflected similar and different codes for each pair of landmarks, corresponding to weaker and stronger landmark discrimination, respectively. If cue cells encode landmark identity, they should exhibit lower rank correlation between disparate compared to identical landmarks. Thus, their rank correlation difference, which was the rank correlation between disparate landmarks subtracted by that between identical landmarks at matched distances, should be below zero (Fig. 3A, b). For each FOV, the number of cue cell groups equaled the number of cells in the FOV (see Methods), enabling comparable statistical power of this calculation with that for activity variation (Fig. 2C).

We found that rank correlations were consistently lower between disparate landmarks compared to identical ones, both at individual and combined distances. This pattern held true whether we analyzed the tracks collectively (Fig. 3B) or individually (Fig. S6A), suggesting that the population code effectively represents landmark identity. Accordingly, rank correlation differences were significantly below zero (Fig. 3C and Fig. S6B). While a trend of positive correlation between rank correlation difference and landmark distance was apparent when pooling data from all tracks (Fig. 3C), the trend was inconsistent across individual tracks (Fig. S6B). This inconsistency, similar to the analysis of activity variation difference across individual tracks (Fig. S1B), suggests that landmark identity encoding is not significantly influenced by landmark distance. Moreover, the finding that rank correlation difference is below zero was robust to variations in the number of cells per group (Fig. S6C). In addition, population-level encoding of landmark identity by the rank correlation metric was largely preserved when grouping cell groups by FOV (Fig. S7A, B) as well as by mouse (Fig. S7C, D).

For individual cue cell groups, their averaged rank correlations for identical and disparate landmark pairs across matched distances were positively correlated and more groups showed lower correlations for disparate landmarks (Fig. 3D and Fig. S8A), indicating a consistent ability of cue cell groups to discriminate both types of landmark pairs, but a stronger ability towards the disparate ones. Moreover, the difference in rank correlations for identical and disparate landmark pairs could be directly revealed by the relative activity of all simultaneously imaged cue cells in a FOV (Fig. 3E and Fig. S8B). We ranked cue cells in an ascending order based on their activities at a reference landmark and arranged their activities at other landmarks with the same ranking. The ascending trend was less apparent at disparate compared to identical landmarks, reflecting the larger difference in relative activity levels between cue cells at disparate landmarks compared to identical ones.

Furthermore, we confirmed that rank correlation difference reflects encoding of landmark identity, rather than frequency-based salience (Fig. S4E, F). This identity encoding is not affected by the distance between landmarks and the reward location (Fig. S5E, F). Together, these findings indicate that the cue cell population code reliably distinguishes between identical and disparate landmarks (Fig. 3F).

### Landmark identity is modulated by spatial shifts of cue cells

**Fig. 4:**
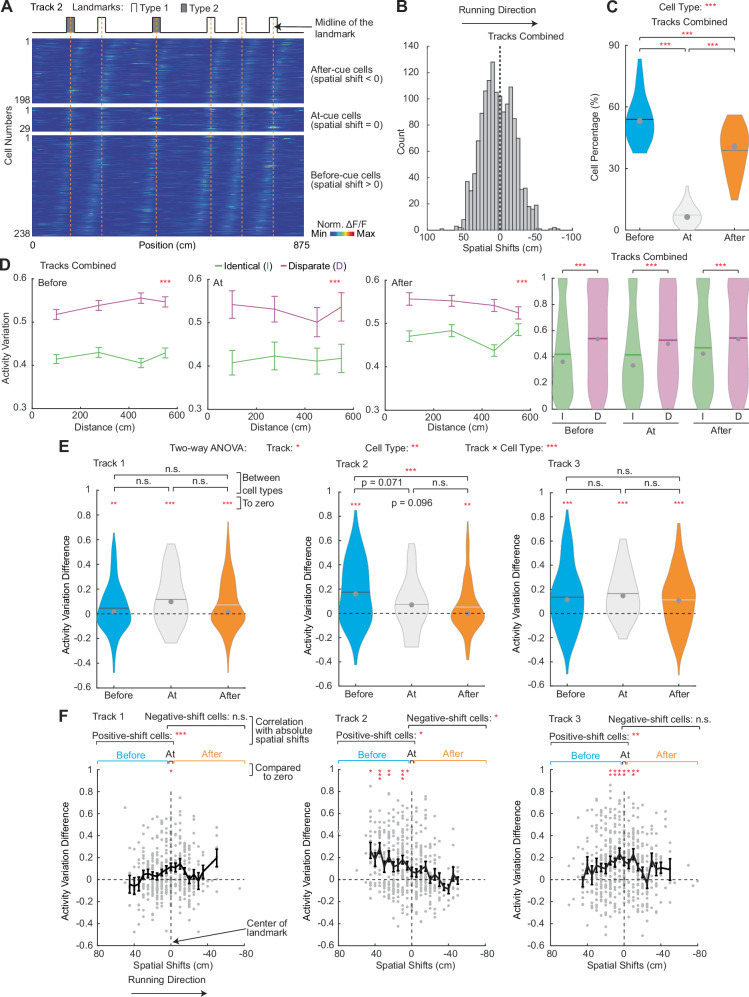
Landmark identity encoding of cue cells is modulated by spatial shift. **A** Activity of before-cue, at-cue, and after-cue cells in track 2, sorted spatial shifts. Hotter color indicates higher normalized activity. **B** Spatial shifts of cue cells pooled from all tracks roughly fall within 50 cm of the landmark template midpoint. **C** The percentages of before-cue (Before), at-cue (At), and after-cue (After) cells pooled from all three tracks. Statistical tests are one-way ANOVA (*p* = 1.15e-21, F = 124, *n* = 30 FOVs for each violin) and two-tailed paired t-tests with Bonferroni-Holm correction (*p*_Before-At_ = 1.71e-17, *p*_At-After_ = 1.85e-13, *p*_Before-After_ = 6.55e-4). **D** Tracks combined: activity variation of before-cue, at-cue, and after-cue cells for identical (I, green) and disparate (D, magenta) landmark pairs at individual (panels 1-3) and combined (panel 4) distances. Panel 1-3: two-way ANOVA comparisons between identical (I, green) and disparate (D, magenta) curves with Bonferroni-Holm correction (Before-cue: 734 cells × 4 distances, *p* = 1.29e-23, F = 106; At-cue: 102 cells × 4 distances, *p* = 2.20e-04, F = 14; 571 cells × 4 distances, *p* = 5.48e-07, F = 27). Panel 4: two-tailed paired t-tests with Bonferroni-Holm correction (*p*_Before_ = 3.88e-56, *n* = 2936 activity variations, *p*_At_ = 3.32e-10, *n* = 408 activity variations, *p*_After_ = 1.50e-18, *n* = 2284 activity variations). **E** Activity variation differences of before-cue, at-cue, and after-cue cells compared with zero and between these cell types. Two-way ANOVA demonstrates track-specific differences in activity variation differences of the cell types (Track: *p* = 0.018, F = 4.0; Cell Type: *p* = 0.0072, F = 4.9; Interaction: *p* = 3.04e-05, F = 6.6). Comparisons with zero: two-tailed paired t-tests. Comparisons between cell types: two-tailed nonpaired t-tests. Bonferroni-Holm correction conducted separately for both comparisons and independently for each track. **F** For each track, activity variation differences of individual cue cells (gray dots) as functions of their spatial shifts. The dashed horizontal line indicates zero difference. Black curve shown for spatial shifts with ≥ 5 cells. Statistical tests include two-tailed Pearson’s linear correlation between the averaged differences and the absolute spatial shifts with ≥ 5 cells for both positive-shift and negative-shift condition as well as two-tailed paired t-tests for comparisons with zero (Bonferroni-Holm-corrected within each track). Exact *n* and *p*-values available in Supplementary Table 1. Data were from 5 mice, including 434, 465, and 508 cells from tracks 1, 2, and 3, respectively. Data are presented as mean ± SEM. See Supplementary Table 1 for statistical details.

The spatial shift of cue cell activity potentially provides vectorial information about an animal’s spatial location relative to landmarks^23^. To understand how this spatial encoding interacts with landmark identity encoding, we investigated the identity encoding by cue cells at various spatial shifts (Fig. 4A). Spatial shifts of cue cells were largely limited to a 50-cm region before and after landmarks (Fig. 4B), as previously reported^23^. Besides a small fraction of cue cells active at landmarks (at-cue cells with zero spatial shifts), most cue cells were active at certain distances away from landmarks, and there were more cells active before compared to after landmarks (before-cue and after-cue cells with positive and negative spatial shifts, respectively) (Fig. 4C). These findings were also apparent for individual tracks (Fig. S9A, B).

Following this, we asked whether landmark identity encoding was regulated by the direction of cue cell activity (i.e., before, at, or after landmarks). When analyzing all tracks together as well as separately, before-cue, at-cue, and after-cue cells all exhibited greater activity variations between disparate compared to identical landmarks at individual and combined distances (Fig. 4D and Fig. S9C–E). Across tracks, before-cue, at-cue, and after-cue cells’ activity variation differences were almost always significantly above zero (Fig. 4E, to zero comparison), indicating that landmark identity is generally encoded regardless of the direction of cue cell activity. However, the relative levels of activity variation differences among these cell types varied across different tracks (Fig. 4E, two-way ANOVA results and between cell types comparison), suggesting a track-specific modulation of landmark identity encoding by the activity direction.

We next examined how landmark identity encoding changes with the distance between cue cell activity and landmarks (i.e., the absolute spatial shifts). We calculated the activity variation difference of each cell by subtracting its averaged activity variation for identical landmarks from that of disparate ones and plotted the differences of individual cells as functions of their spatial shifts (Fig. 4F). For each track, we further grouped cue cells into positive-shift cells (at-cue and before-cue cells) and negative-shift cells (at-cue and after-cue cells), allowing us to separately calculate the correlation between activity variation differences and absolute spatial shifts. We observed significant correlations for positive-shift cells in all tracks, but the trends of correlation varied: the cells in tracks 1 and 3 showed increased activity variation differences with reduced absolute spatial shifts, whereas the cells in track 2 showed the opposite trend. In contrast, significant correlations were not always observed in negative-shift cells. Additionally, the difference in the correlation significance for positive-shift and negative-shift cells was not due to the fewer number of negative-shift cells (Fig. S10A). The track-specific relationship between activity variation difference and spatial shift was also true for individual mice (Fig. S10B). Thus, while landmark identity is modulated by the distance between cue cell activity and landmarks, more consistent modulation occurs when a mouse is approaching (i.e. when positive-shift cells are active) rather than moving away from (i.e. when negative-shift cells are active) landmarks. The trend of the modulation is track specific.

Collectively, these results reveal both general and track-specific interactions between landmark identity encoding and spatial shifts of cue cells. Landmark identity is encoded by cue cells active in all directions (before, at, and after landmarks). This encoding is more consistently modulated by the distance of cue cell activity from landmarks when a mouse is approaching, yet the detailed modulations by direction and distance are track specific.

### The encoding of landmark identity by cue cells changes in different environments

Given the above track-specific modulation of landmark identity encoding by spatial shifts of cue cells, we further asked whether landmark identity encoding by the same cue cell population also varies in different tracks. We focused on common cue cells in each pair of tracks (tracks 1 and 2, 2 and 3, or 1 and 3) (Fig. S11A). To increase the statistical power for the following analyses, cue cells in each track were classified using a lower threshold (above the 80^th^ percentile of the shuffled distribution) and common cue cells across tracks were further identified (Fig. S12A). These common cue cells showed strong landmark-associated activity (Fig. S12B–D) and weakly correlated spatial shifts across tracks (significant correlations in two out of three track pairs) (Fig. S11B), as previously reported^23^, but uncorrelated cue scores (Fig. S11C).

**Fig. 5:**
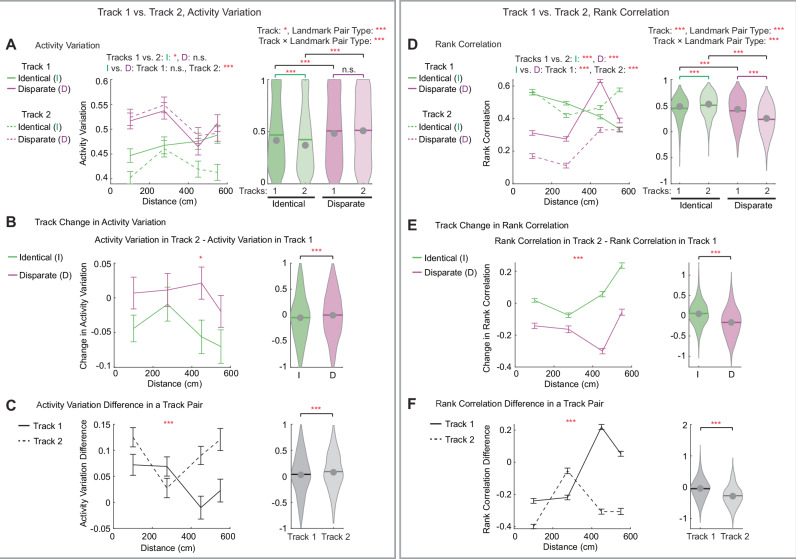
Cue cells encode landmark identity differently in different tracks. **A** Left: Activity variation of common cue cells in track 1 (solid) versus track 2 (dashed) for identical (I, green) and disparate (D, magenta) landmark pairs across distances. Statistics are two-way ANOVA comparisons with Bonferroni-Holm correction. Right: calculations for combined distances. Above statistics are two-way ANOVA comparisons showing that track and landmark pair type jointly modulate activity variation (Track: *p* = 0.0399, F = 4.2, Landmark Pair Type: *p* = 1.185e-18, F = 80.0, Interaction: *p* = 6.93e-04, F = 11.6). Post-hoc comparisons are Bonferroni-Holm-corrected two-tailed paired t-tests. **B** Track change in activity variation for identical (green) and disparate (magenta) landmark pairs between tracks 1 and 2 at individual (left) and combined (right) distances. Statistical tests are two-way ANOVA (left, 359 cells × 4 distances, *p* = 0.023, F = 5.2) and two-tailed paired t-test (right, *n* = 1436 changes in activity variation, *p* = 6.93e-04). **C** Similar to (**B**), but for activity variation difference for tracks 1 and 2. Statistical tests are the same as those in (**B**) (left, 359 cells × 4 distances, *p* = 4.95e-04, F = 12.3; right, *p* = 2.73e-04, *n* = 1436 activity variation differences). **D** Similar to **A** for rank correlations in tracks 1 and 2. Each cue cell group contains 15 cells. Left: Bonferroni-Holm-corrected two-way ANOVA. Right: Two-way ANOVA (Track: *p* = 5.72e-15, F = 62, Landmark Pair Type: *p* = 3.85e-100, F = 532, Interaction: *p* = 3.52e-81, F = 415) and Bonferroni-Holm-corrected two-tailed paired t-tests. **E** Similar to **B** for changes in rank correlations between tracks 1 and 2. Left: Two-way ANOVA, 359 cell groups × 4 distances, *p* = 8.63e-39, F = 192. Right: two-tailed paired t-test, *n* = 1436 changes in rank correlation, *p* = 3.52e-81. **F** Similar to **C** for rank correlation differences in tracks 1 and 2. Left: Two-way ANOVA, 359 cell groups × 4 distances, *p* = 7.19e-49, F = 253. Right: two-tailed paired t-test, *n* = 1436 rank correlation differences, *p* = 5.98e-62. Data were from 5 mice, including 359 cells or cell groups common to day 1 of track 1 and track 2. Data are presented as mean ± SEM. See Supplementary Table 1 for statistical details.

We first compared the activity variation of common cue cells. Between tracks 1 and 2, cue cells showed significant changes in activity variations for identical landmark pairs (Fig. 5A, the two green groups), but not for disparate pairs (Fig. 5A, the two magenta groups), suggesting that switching tracks differentially affected their ability to discriminating these two types of landmark pairs (Fig. 5A, top right: two-way ANOVA). The different degrees of change in activity variation (Fig. 5B) resulted in distinct activity variation differences between the two tracks (Fig. 5C), reflecting variable levels of landmark identity encoding by the same cue cells in different tracks. Similar results were obtained for other track pairs (Fig. S13).

Similarly, cue cell groups exhibited distinct changes in their rank correlations for identical versus disparate landmark pairs across different tracks (Fig. 5D and Fig. S14A). These differential changes (Fig. 5E and Fig. S14B) led to altered rank correlation differences across different tracks (Fig. 5F and Fig. S14C).

In addition, activity variation differences for individual cells (Fig. S15A), as well as rank correlation differences for individual cell groups (Fig. S15B) both showed differences for pairwise track comparisons. Moreover, the differential rank correlation difference for individual cell groups between different tracks was largely consistent across various numbers of cells per group, demonstrating robustness (Fig. S15C).

The findings were largely recapitulated when using a more stringent threshold (95^th^ percentile of the shuffled distribution) to classify common cue cells, despite the smaller sample size (Fig. S16). These results together suggest that the capacity of the same cue cells to encode landmark identity is modulated in an environment-specific manner.

### The encoding of landmark location but not identity is modulated by experience in cue cells

We next investigated whether landmark identity encoding by cue cells is affected by increased experience in the same environment during spatial learning. We focused on tracks 1 and 2 which allowed for the comparison of cue cell activity on day 1 (with less experience) and day 7 (with more experience) in the same track (Fig. S11D). Common cue cells on the two days, which were similarly classified using an 80^th^ percentile threshold of the shuffled distribution (Fig. S17A), exhibited strong landmark-associated activity (Fig. S17B, C). They also showed well-correlated spatial shifts (Fig. S11E) and cue scores (Fig. S11F), contrasting with the weakly correlated spatial shifts (Fig. S11B) and uncorrelated cue scores (Fig. S11C) between different tracks. Thus, cue cells better preserve their landmark-associated activation within the same track compared to between different tracks.

**Fig. 6:**
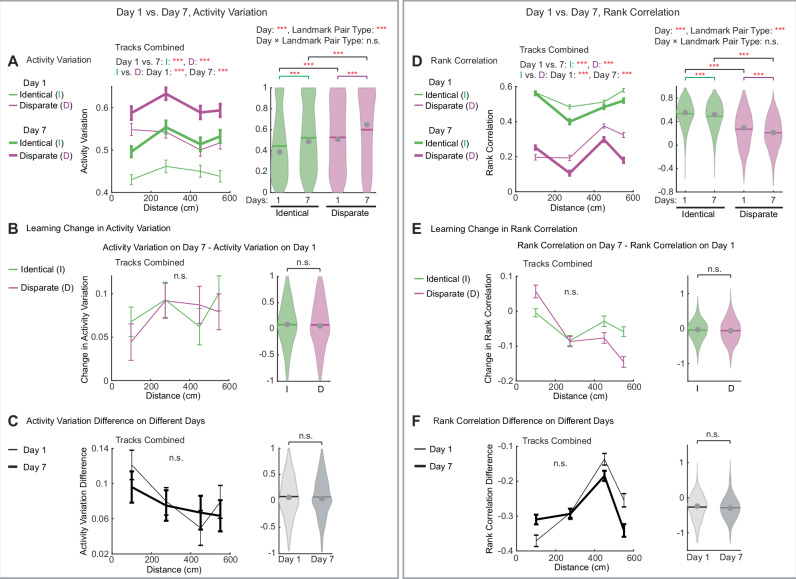
Cue cells encode landmark identity in an experience-independent manner. **A** Left: activity variation of common cue cells on day 1 (thin) vs day 7 (thick) for identical (I, green) and disparate (D, magenta) landmark pairs across distances, with data pooled from tracks 1 and 2. Statistics are two-way ANOVA comparisons with Bonferroni-Holm correction. Right: calculations for combined distances pooled from both tracks. Statistics are two-way ANOVA comparisons (Day: *p* = 4.30e-21, F = 91, Landmark Pair Type: *p* = 2.19e-31, F = 141, Interaction: *p* = 0.68, F = 0.17) with post-hoc two-tailed paired t-tests with Bonferroni-Holm correction. **B** Learning change in activity variation for identical (green) and disparate (magenta) landmark pairs on days 1 and 7 for both tracks together at individual (left) and combined (right) distances. Statistics are two-way ANOVA (left, 473 cells × 4 distances; *p* = 0.79, F = 0.07) and two-tailed paired t-tests (right, *n* = 1892 changes in activity variation, *p* = 0.68). **C** Similar to (**B**), but for activity variation differences on days 1 and 7. Statistical tests are the same as those in (**B**) (left, 473 cells × 4 distances, *p* = 0.80, F = 0.06; right, *p* = 0.68, *n* = 1892 activity variation differences). **D** Similar to **A** for rank correlations on days 1 and 7. Each cue cell group contains 15 cells. Left: Bonferroni-Holm-corrected two-way ANOVA. Right: Two-way ANOVA (Day: *p* = 5.24e-22, F = 96, Landmark Pair Type: *p* = 1.93e-281, F = 1910, Interaction: *p* = 0.07, F = 3.3). Bonferroni-Holm-corrected two-tailed paired t-tests. **E** Similar to **B** for changes in rank correlations between days 1 and 7. Left: Two-way ANOVA, 431 cell groups × 4 distances, *p* = 0.19, F = 1.7. Right: two-tailed paired t-test, *n* = 1724 changes in rank correlation, *p* = 0.07. **F** Similar to **C** for rank correlation differences on days 1 and 7. Left: Two-way ANOVA, 431 cell groups × 4 distances, *p* = 0.20, F = 1.6. Right: two-tailed paired t-test, *n* = 1724 rank correlation differences, *p* = 0.07. Activity variation data were from 5 mice, including 217 and 256 cells across days in track 1 and 2, respectively. Rank correlation data were from 3 mice (188 cell groups across days) and 5 mice (243 cell groups across days) in track 1 and 2, respectively. Data are presented as mean ± SEM. See Supplementary Table 1 for statistical details.

When examining activity variation of cue cells with less versus more experience in the same track, we observed an increase from day 1 to day 7 for both identical and disparate landmark pairs, regardless of whether we aggregated (Fig. 6A) or separated (Fig. S18A) the data from tracks 1 and 2. This suggests an improved ability to differentiate landmarks with increased experience. The increase in activity variation was consistently observed in before-cue cells but not after-cue cells, indicating a specific improvement in prospective landmark encoding (Fig. S19A). Interestingly, the degree of improvement was similar for identical and disparate landmarks (Fig. 6B and Figs. S18B and S19B), and consequently, activity variation differences on the two days were comparable (Fig. 6C and Figs. S18C and S19C).

Similarly, cue cell groups (Fig. 6D and Fig. S20A), especially before-cue cell groups (Fig. S21A), decreased their rank correlations from days 1 to 7 by the same extent for identical and disparate landmarks (Fig. 6E and Figs. S20B and S21B), leading to similar rank correlation differences on the two days (Fig. 6F and Figs. S20C and S21C).

Moreover, on an individual cell and individual cell group level, activity variation (Fig. S22A) and rank correlation (Fig. S22B), respectively, showed no significant difference between days 1 and 7. This effect with the rank correlation metric was generally robust to the choice of the number of cells per group (Fig. S22C).

The above findings were largely recapitulated when using a more stringent threshold (95^th^ percentile of the shuffled distribution) to classify common cue cells across days, despite the smaller sample size (Fig. S17D–I). These results indicate that cue cells largely maintain a consistent level of landmark identity encoding with increasing experience within the same environment, in contrast to the pronounced changes across different environments (Fig. 5 and Figs. S13–16). Therefore, landmark identity encoding by cue cells is environment dependent but experience independent. In contrast, increased experience enhances cue cells’ ability to distinguish landmarks regardless of their identity or spatial distance (Fig. 6 and Figs. S17–22), indicating experience-dependent encoding of landmark location.

### The encodings of landmark identity and location are differentially supported by cue cells and putative grid cells

While the above results revealed the integration of spatial and nonspatial information in cue cell activity, we further asked whether this integration also occurs in grid cells, which are known to encode spatial information during navigation^3^. We classified putative grid cells on days 1 and 7 in tracks 1 and 2^26,27,29–31^. We removed grid cells conjunctive with cue cells^23^ to minimize the effect of cue cell-like activity on putative grid cell response. Indeed, the remaining putative grid cells did not show cue-specific activity (Fig. S23A).

**Fig. 7:**
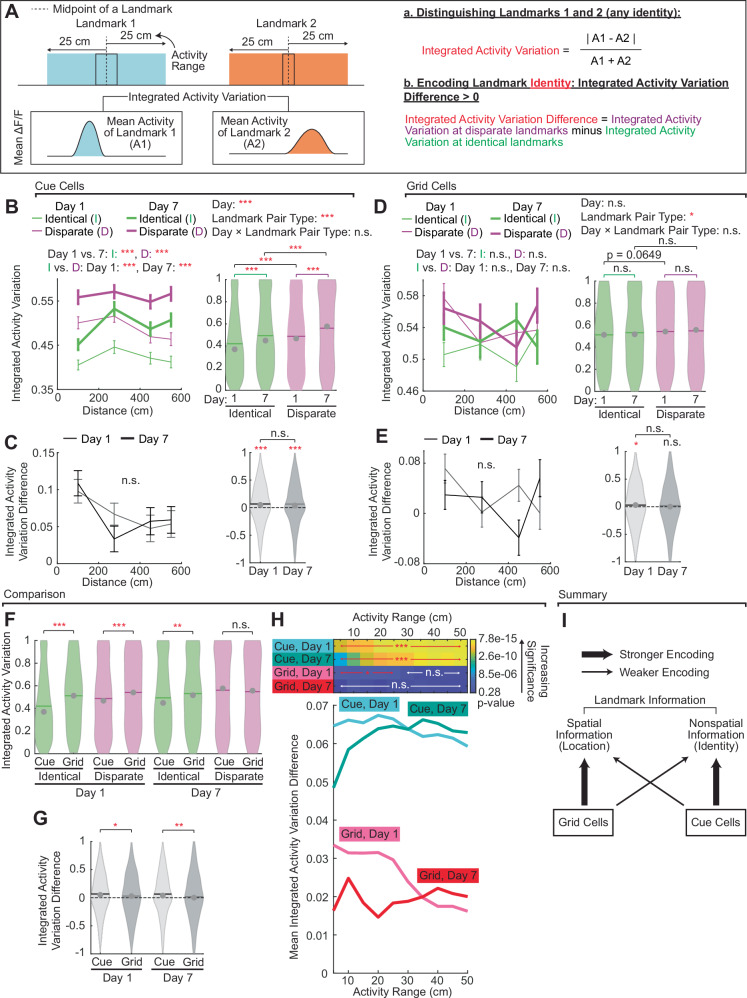
Landmark location and identity are preferentially encoded by grid cells and cue cells, respectively. **A** Schematic for integrated activity variation (IAV) calculation. Landmark midpoints are noted with dotted lines. **B** IAV for identical (I, green) and disparate (D, magenta) landmarks at individual (left) and combined (right) distances on day 1 (thin) and day 7 (thick) for cue cells. Left: Bonferroni-Holm-corrected two-way ANOVAs. Right: two-way ANOVAs (Day: *p* = 2.28e-26, F = 116, Landmark Pair Type: *p* = 1.81e-23, F = 102, Interaction: *p* = 0.88, F = 0.021) and Bonferroni-Holm-corrected two-tailed paired t-tests. **C** IAV difference for cue cells on days 1 and 7 at individual (left) and combined (right) distances. Left: two-way ANOVA (437 cells × 4 distances, *p* = 0.94, F = 0.0049). Right: Bonferroni-Holm-corrected two-tailed paired t-tests with zero as well as a two-tailed paired t-test comparing samples. **D** Similar to **B** for putative grid cells. Left: Bonferroni-Holm-corrected two-way ANOVA. Right: Two-way ANOVA (Day: *p* = 0.17, F = 1.9, Landmark Pair Type: *p* = 0.011, F = 6.5, Interaction: *p* = 0.52, F = 0.42) and Bonferroni-Holm-corrected two-tailed paired t-tests. **E** Similar to **C** for putative grid cells. Left: Two-way ANOVA, 250 cells × 4 distances, *p* = 0.46, F = 0.56. Right: Bonferroni-Holm-corrected two-tailed paired t-tests with zero as well as a two-tailed paired t-test comparing samples (*n* = 1000 IAV differences each, *p*_day1,0_ = 0.032, *p*_day7,0_ = 0.17, *p*_day1, day7_ = 0.52). **F** Comparison of IAV in cue and putative grid cells for identical and disparate landmarks on days 1 and 7 (Bonferroni-Holm-corrected two-tailed non-paired t-tests: *n*_cue_ = 1892, *n*_grid_ = 1000, *p*_I-day1_ = 1.05e-15, *p*_D-day1_ = 1.77e-05, *p*_I-day7_ = 3.33e-03, *p*_D-day7_ = 0.34). **G** IAV difference in cue and putative grid cells on days 1 and 7; Bonferroni-Holm-corrected two-tailed non-paired t-tests (*n* = 1892 and *n* = 1000 IAV differences for cue and putative grid cells, respectively, Day 1 Cue vs Putative Grid: *p* = 0.012, Day 7 Cue vs Putative Grid: *p* = 0.0059). **H** Mean IAV differences of cue cells and putative grid cells on days 1 and 7 as functions of activity range. Hotter heatmap colors indicate more significant two-tailed paired t-tests with zero, with Bonferroni-Holm correction as in (**C**) and (**E**). **I** Summary schematic: both grid and cue cells are involved with the encoding of spatial (location) and nonspatial (landmark identity) information, but to different degrees. Cue cell data were from 5 mice, including 217 and 256 cells across days in track 1 and 2, respectively. Putative grid cell data were from 5 mice, including 132 and 118 cells common to day 1 and day 7 of track 1 and track 2, respectively. Data are presented as mean ± SEM. See Supplementary Table 1 for statistical details.

Our calculation focused on common putative grid cells on days 1 and 7 in tracks 1 or 2 (Fig. S23A). Since grid cells lack clearly defined, landmark-anchored activity, we examined their integrated activity within an activity range spanning 25 cm on both sides of each landmark’s midline (Fig. 7A). For each landmark, we averaged the integrated activity and then calculated the integrated activity variation between a pair of landmarks (Fig. 7A). Integrated activity variation difference between identical and disparate landmark pairs was used as a measure of landmark identity encoding by putative grid cells. To enable a fair comparison between functional cell types, we applied the same analysis to cue cells.

Combining data from tracks 1 and 2, this analysis recapitulated landmark identity encoding by cue cells, as evidenced by greater integrated activity variation between disparate landmark pairs compared to identical ones (Fig. 7B). Consistent with the previous findings (Fig. 6), cue cells showed increased integrated activity variation for both identical and disparate landmark pairs from day 1 to day 7 (Fig. 7B), without altering integrated activity variation differences between the two days (Fig. 7C). These results reflect improved encoding of landmark location, while maintaining stable landmark identity encoding during learning.

In contrast, for putative grid cells on day 1, the integrated activity variation between disparate landmark pairs tended to be higher than that between identical ones (Fig. 7D), resulting in an integrated activity variation difference significantly greater than zero (Fig. 7E). This suggests a weak encoding of landmark identity. However, such encoding was no longer observed on day 7. Moreover, integrated activity variations for both types of landmark pairs did not increase from day 1 to day 7 (Fig. 7D), suggesting that learning-related spatial encoding improvements in grid cells, reported previously^27^, may instead be captured by other activity features, such as increased spatial activity consistency^27^.

A comparison of integrated activity variation for cue and putative grid cells for identical and disparate landmarks shows that putative grid cells generally exhibited greater variations (Fig. 7F), consistent with their strong spatial encoding. However, cue cells demonstrated significantly greater integrated activity variation differences on both days 1 and 7 (Fig. 7G), indicating that they more robustly encode landmark identity.

To fully evaluate the influence of landmark identity on grid cell activity, we analyzed the integrated activity variation difference of putative grid cells across activity ranges from 5 to 50 cm. On day 1, putative grid cells exhibited landmark identity encoding (i.e., integrated activity variation difference significantly above zero) only within 25 cm of landmarks (Fig. 7H). Including the activity further away from landmarks (>25 cm) abolished the encoding. This distance-dependent landmark identity encoding on day 1 suggests that upon initial environmental exposure, grid cell activity is influenced by landmark identity only when the animal is very close to these landmarks. On day 7, putative grid cells no longer encoded landmark identity at any activity range, whereas cue cells consistently encoded identity across all activity ranges.

Similar trends were observed when analyzing tracks 1 and 2 separately (Figs. S23 and S24), as well as by studying the activity of cue and putative grid cells on day 1 of track 3 (Fig. S25). Together with previous reports of strong spatial encoding by grid cells^3^, these results demonstrate that although both cue and grid cells encode spatial and nonspatial information of landmarks, in the form of their location and identity, respectively, cue cells more robustly encode landmark identity, whereas grid cells primarily represent landmark location (Fig. 7I).

## Discussion

Previous literature has shown an ability of the lateral entorhinal cortex (LEC), the hippocampus, and other association cortices to encode nonspatial landmark identity information^32–37^. Here, we note an encoding of landmark identity within the MEC, as well. This ability is supported by spatial activity of cue cells, which respond to individual landmarks during virtual navigation^23^. Cue cells encoded landmark identity by responding more differentially to disparate compared to identical landmarks. The encoding was conducted by cue cells active before, at, and after landmarks, but the relative encoding levels were modulated by the activity locations of individual cue cells in a track-specific manner. The same individual and populations of cue cells altered their landmark identity encoding in different tracks but maintained their encoding in the same environment despite increased environmental experience. In contrast, increased experience led to improved prospective encoding of landmark locations by cue cells. Finally, grid cells more robustly encoded landmark locations compared to cue cells and only weakly encoded landmark identity upon initial exposure to an environment. Together, our findings highlight the MEC’s capacity to encode landmark identity so that spatial and nonspatial information of an environment can be integrated in the MEC’s cognitive map before being delivered to the hippocampus.

The representation of landmark identity by MEC cue cells exhibits both uniqueness and commonality compared to other brain regions such as the hippocampus, LEC, and other association cortices^32–36^. First, object selectivity in the other regions is mostly achieved by the cells specifically active at a particular subset of objects^32–36^. However, cue cells respond to all landmarks^23^ but vary their activity at individual ones according to their identity. A similar response was also identified in landmark vector cells in the hippocampal CA1^37^. Second, object-specific activities in the LEC and many association cortices largely appear when animals reach the object location^32–35^, whereas cue cells can be active when the mice are away from individual objects at certain distances and orientations. This feature is also observed in object vector cells in the MEC^22^, landmark vector cells in the hippocampus^36^, boundary vector cells in the subiculum^38^, and some object-responsive cells in the LEC^32^. This allows cue cells to not only provide a vectorial representation of animals' location relative to landmarks, but also to represent landmark identity across a broad area around landmarks, potentially supporting precise mapping of animal’s position during real-time navigation in environments with complex landmark features. Lastly, the encoding of specific objects can be regulated by either sensory stimuli or cognition, as some object-responsive cells were only active when the objects were present (sensory stimuli-driven)^32,34–36^, whereas others in the LEC, the hippocampus, and the anterior cingulate cortex encode the previous existence of objects at specific places (cognition-driven)^32,34,36,39^. In terms of cue cells, a previous study found that their landmark response disappeared when the landmark was removed^23^, indicating that the response does not encode the previous existence of landmarks. Here, we reveal that the dependency of landmark identity encoding on cue cell activity location relative to landmarks is environment specific. The identity encoding by the same cue cell population is also environment-specific and is not regulated by increased experience. These results suggest that landmark identity encoding by cue cells is driven by sensory features of an environment. In contrast, the landmark location encoding is likely driven by cognitive experience in the environment.

In addition to cue cells, we also discovered the encoding of landmark identity by grid cells, although this encoding is weak and is only apparent in novel environments when the animal is spatially near landmarks. This observation suggests that grid cell activity, which primarily carries spatial information based on the current literature^3^, can also be modulated by nonspatial visual information. Previous studies also found that grid cells exhibit object-selective activity when the objects are associated with reward^21^, display rate remapping when environmental features such as shape and color are altered^19^, and undergo global remapping in environments that are spatially similar but defined by cues from different sensory modalities^20^. While these forms of remapping occur across distinct environments and likely contribute to context encoding, the activity variations we observe near landmarks occur within a single environment and closely track landmark identity in novel environment. Together, these results suggest that grid cells not only present spatial information but also incorporate nonspatial information from the environment into their cognitive map. Interestingly, whereas changes in environmental shape and color induced only rate remapping in grid cells, non-grid cells showed a complete reorganization of their firing patterns. This further suggests that non-grid cells may distinguish environmental features more robustly, consistent with the stronger landmark-identity encoding by cue cells than by grid cells reported here.

Classical models propose that neocortical inputs conveying spatial and nonspatial information separately arrive separately at the LEC and MEC, respectively. Both regions project to the hippocampus, which combines the two information types into a complete cognitive map^40–42^. However, mounting evidence, including our own findings, indicate that the MEC encodes both spatial and nonspatial information during navigation. In our study, visual landmark identity information could reach the MEC via direct and indirect inputs from visual cortical areas^43–50^. Similarly, spatial and nonspatial information is also jointly represented by the LEC based on several anatomical^51^, behavioral^52–54^, and neural recording studies^21,32,33^. Collectively, we propose that although the MEC and LEC show preferential encoding of spatial and nonspatial information, respectively^3–5,32,33,55^, both regions are also capable of integrating spatial and nonspatial signals before transmitting them to the hippocampus. This architecture supports both specialized local computations within MEC and LEC circuits and the generation of a comprehensive cognitive map across the broader entorhinal-hippocampal network, ultimately enabling robust and flexible navigation.

## Methods

### Animals

All animal procedures were performed in accordance with animal protocol 1524 approved by the Institutional Animal Care and Use Committee (IACUC) at NIH/NINDS. For two-photon imaging experiments, GP5.3 mice (C57BL/6J-Tg (Thy1-GCaMP6f) GP5.3Dkim/J, JAX stock #028280)^56^ were used, including 8 males and 3 females. Of these, the main experimental (tracks 1-3) mice included 3 males and 2 females aged 4-5 months at the time when imaging began. Track 4 mice (Fig. S4) included the original 5 mice from the main experiment as well as an additional 5 male mice and 1 female mouse aged 6-7 months at the time when imaging began. Track 5 data (Fig. S4) were made up entirely of the additional 5 male and 1 female mice. Mice were maintained on a reverse 12-hr on/off light schedule with all experiments being performed in the light off period. Animals were housed at a temperature of 70-74° and 40-65% humidity. Analyses were not separated by sex, as previous work found navigation performance to be similar across sex^27^; the main experiment was roughly sex-balanced for broad applicability of the findings.

### Surgery

#### Microprism construction

Microprism construction procedures were similar to those described previously^24,26^. A canula (MicroGroup, 304H11XX) was attached to a circular cover glass (3 mm, Warner Instruments, 64-0720). A right angle microprism coated with aluminum on the hypotenuse (1.5 mm, OptoSigma, RBP3-1.5-8-550), was attached to the opposite side of the cover glass. All attachments were performed using UV-curing optical adhesive (ThorLabs, NOA81).

#### Microprism implantation surgery

Microprism implantation procedures were similar to those described previously^24,26^. Mice were anesthetized using a tabletop laboratory animal anesthesia system (induction: 3% isoflurane, 1 L/min oxygen, maintenance: 0.5%-1.5% isoflurane, 0.7 L/min oxygen, VetEquip, 901806) and surgery was performed on a stereotaxic alignment system (Kopf Instruments, 1900). A homeothermic pad and monitoring system (Harvard Apparatus, 50-7220 F) was used to maintain a body temperature of 37 °C. After anesthesia induction, dexamethasone (2 mg/kg, VetOne, 13985-533-03) and saline (500 µL, 0.9% NaCl, McKesson, 0409-4888-50) were administered by intraperitoneal (IP) injection, and slow-release buprenorphine (1 mg/kg, ZooPharm, Buprenorphine SR-LAB) was administered subcutaneously. Enroflox 100 (10 mg/mL, VetOne, 13985-948-10) was used as an antimicrobial wash just after the skull was exposed and just prior to sealing the skull. All insertions were performed on the left hemisphere, aligning with previous observations of more favorable vasculature on the left side^24^. A 3 mm craniotomy was performed centered at 3.4 mm lateral to the midline and 0.75 mm posterior to the center of the transverse sinus (approximately 5.4 mm posterior to the bregma). A durotomy was then performed over the cerebellum. Mannitol (3 g/kg, Millipore Sigma, 63559) was administered by IP prior to the durotomy. A microprism implant, which was assembled as described above, was inserted into the transverse sinus and sealed to the skull with Vetbond (3 M, 1469SB). A headplate was then mounted on the skull opposite the craniotomy. Finally, the prism and headplate were adhered to the skull with Metabond (Parkell).

### Virtual reality

For all visual-behavioral experiments, a customized virtual reality (VR) setup was used, which projects a one-dimensional (1D) virtual environment based on the running of a mouse, similar to that described previously^26^. Mice were head-fixed onto an air-supported polystyrene ball (8” diameter, Smoothfoam) using the mounted headplate. The ball rotated on an axle, allowing only forward and backward rotation. The virtual environment was projected onto a dome screen filling the visual field of mice (270° projection). An optical flow sensor (Paialu, paiModule_10210) with infrared LEDs (DigiKey, 365-1056-ND) was used to measure the rotation of the ball and thereby control the motion of the virtual environment. The optical flow sensor output to an Arduino board (Newark, A000062), which transduced the motion signal to the computer controlling the virtual reality. An approximately 4 μl water reward was provided via a lick tube at a fixed location in a given environment using a solenoid. A lick sensor connected to both the lick tube and headplate holder was used to detect mouse licking. A mouse licking the lick tube created a closed circuit between the lick sensor, the lick tube, the mouse (from the tongue to the skull), the headplate (which directly contacts the skull), and the headplate holder. The solenoid and lick sensor were controlled using a Multifunction I/O DAQ (National Instruments, PCI-6229). The virtual environments were generated and projected using ViRMEn software (Princeton, version 2016-02-12) within MATLAB R2015aSP1^57^. Imaging and behavior data were synchronized by recording a voltage signal of behavioral parameters from the VR system using the DAQ. ViRMEn environments were updated at 60 Hz. The DAQ input/output rate was 1 kHz. The synchronization voltage signal was updated at 20 kHz. Final behavioral outputs were matched to the imaging frame rate (30 Hz, see Two-photon Imaging section of Methods) for synchronization.

Environments were projected through a Wratten filter (Kodak, 53-700) to reduce contamination of the imaging path with projected light. Virtual environments were 1D linear tracks with patterned walls and patterned visual cues at fixed locations. At the end of the track, mice were immediately teleported to the start of the track.

### Behavior

#### Training in VR

Mice were allowed to recover for 5 days after prism implantation surgery and were then placed on water restriction, receiving 1 mL water per day. After approximately 3 days of water restriction, mice were trained daily in VR. The mice were first trained on a 1-meter track to encourage running. They were further trained on a 10-meter track with two water rewards until familiarization, as measured by consistent running (>40 trials per hour) and stable significant anticipation of reward locations for 3 days (less than 5% change in predictive licking, see Behavior – Predictive licking in Methods for quantification). The mice were then switched to a novel environment (track 1, 875 cm) for 7 days of navigation. On day 7 in track 1, the mice conducted a second session to explore the next novel environment (track 2, 875 cm). The mice navigated in track 2 for 7 days and on the last day, they conducted a final session (one day) to explore the third novel environment (track 3, 1000 cm). The mice were imaged daily from the first day in track 1 to the first day in track 3 (a total of 13 days). Each training or imaging session lasted around 45 mins, during which the mice traversed at least 12 runs on the track.

#### Predictive licking

Predictive licking was the percentage of licks that occurred within 20 cm prior to the reward location relative to all other locations (excluding 30 cm after reward)^27^.

### Two-photon imaging

Imaging was performed using an Ultima 2Pplus microscope (Bruker) configured with the above VR setup based on an existing method^27^. A tunable laser (Coherent, Chameleon Discovery NX) set to a 920 nm excitation wavelength was used. Laser scanning was performed using a resonant-galvo scanner (Cambridge Technology, CRS8K). GCaMP fluorescence was isolated using a bandpass emission filter (525/25 nm) and detected using GaAsP photomultiplier tubes (Hamamatsu, H10770PB). A 16x water-immersion objective (Nikon, MRP07220) was used with ultrasound transmission gel (Sonigel, refractive index: 1.335989; Mettler Electronics, 1844) as the immersion media.

The anterior-posterior (AP) and the medial-lateral (ML) angle of the prism (i.e., the angle of the surface of the prism along to the AP or ML direction of the mouse) relative to the head-fixed position of the mouse were measured prior to the first imaging session. The headplate holder and rotatable objective angles were set before each imaging session to align the objective with the prism in the AP and ML direction, respectively, such that the objective was in parallel with the prism surface. A black rubber tubing was wrapped around the objective and imaging window to prevent light leakage into the objective.

Microscope control and image acquisition were performed using Prairie View software (Bruker, version 5.7). Raw data was converted to images using the Bruker Image-Block Ripping Utility. Dual-plane imaging was performed through a Z-Piezo, which enabled the objective to travel between two planes (30-µm apart) within layer 2. Imaging data in each plane were collected at 7.4 Hz and at 512 × 512 resolution (1.116 µm/pixel). Average beam power at the front of the objective was typically 70-115 mW. Imaging and behavior data were synchronized as described above in the virtual reality section of Methods.

### Image processing

#### General processing

Motion correction was performed using cross-correlation based rigid motion correction^27^. Identification of regions of interest (ROIs) and the extraction of their fluorescence time course were performed using Suite2p v0.11.1^58^. The fractional change in fluorescence with respect to baseline (ΔF/F) was calculated as (F(t) – F0(t)) / F0(t)^24^. For each cell, significant calcium transients were identified using amplitude and duration thresholds, such that the false-positive rate of significant transient identification was 1%^31^. A final ΔF/F including only the significant calcium transients was used for all further analysis. The mean ΔF/F (significant transients only) for a cell was calculated as a function of position along the track in 5 cm bins. Data points when the mouse was moving below a speed threshold were excluded from this analysis. The speed threshold was calculated by generating a 100-point histogram of all instantaneous velocities greater than 0 and taking the value twice the center of the first bin (approximately 1% of max positive speed).

#### Cell alignment

All imaging sessions for a given FOV were aligned pairwise using the alignment toolbox developed previously^59^ to identify common cell pairs between each pair of sessions (e.g., common cells of the sessions on days 1 and 7 in track 1). Pairwise alignments were combined to generate the full set of possible cell alignments for more than two imaging sessions (e.g., common cells on the first days in tracks 1, 2, and 3). All alignments were manually validated for accuracy.

### Data analysis

#### General data handling, analysis, and visualization

All data handling, analysis, and visualization post-image processing was done using MATLAB version R2023-2024.

#### Cue cells in one session

Cue scores were calculated as previously described^23^. In each track, since visual landmarks were symmetrically arranged on both sides of the track, the calculation used a single landmark template, in which spatial bins (5 cm bin) within and outside of landmark areas (between the front and back edge of each landmark) were set to 1 and 0, respectively. The cross correlation between the landmark template and the spatially binned mean ΔF/F (described in Image Processing - General processing) was first calculated (relative shift ≤ 300 cm). The peak in the cross correlation with the smallest absolute shift from zero was chosen as the best correlation of the mean ΔF/F to the landmark template. The spatial shift at which this peak occurred was then used to displace the landmark template to best align with the mean ΔF/F. Spatial shifts were restricted to 5 cm increments, as the mean ΔF/F was calculated by every 5 cm bins along the track. The correlation was then calculated locally between mean ΔF/F and each landmark within the landmark zone, which included the landmark region and the region on either side extending by half of the landmark width. Note that the landmark zone of the same landmark could occupy different spatial bins on the mean ΔF/F of individual cells, because the landmark template could have different spatial shifts relative to the mean ΔF/F. The mean of local correlation values across all landmarks was calculated and defined as ‘cue score’ of the cell.

To identify cue cells in a particular track, a cue score threshold was calculated as the 95^th^ percentile of shuffled cue scores, which combined 200 shuffles of each cell from all FOVs of all mice and in all imaging sessions. The cells with cue scores above the threshold were identified as cue cells. Each cue cell was characterized by its spatial shift and a set of landmark zones for individual landmarks, as described above.

#### Cue cells across multiple sessions

To identify common cue cells across 2 imaging sessions (day 1 in a pair of tracks or days 1 and 7 in the same track), the same cue score calculation was conducted as described above, but the 80^th^ percentile of shuffles was used as a threshold for cue cells. All cells that passed this threshold and were also present in multiple sessions were identified as cue cells across those sessions. To further validate the results, additional analyses were conducted on common cue cells classified using the 95^th^ percentile of shuffles (Figs. S12,16 and 17).

#### Before-cue, at-cue, and after-cue cells in one session

Before-cue and after-cue cells were determined as the cue cells that were active before and after individual landmarks (spatial shift <0 and > 0), respectively. At-cue cells were the cue cells with zero spatial shift.

#### Before-cue and after-cue cells across two sessions

Before-cue and after-cue cells on days 1 and 7 in the same track were the ones identified as those cells in both sessions based on the above criteria.

#### Putative grid cells in one session

Grid cells were identified by an existing method^26,27^ based their activity features on linear tracks. For the activity of each cell, we first identified its spatial fields, which were defined by comparing the mean ΔF/F value in each 5 cm spatial bin to that of a random distribution created by 1000 bootstrapped shuffled responses^26,29^. For each 5 cm bin, a *p*value equaled the percent of shuffled mean ΔF/F that were higher than the real mean ΔF/F. Therefore, 1-*p*value equaled the percent of shuffled mean ΔF/F lower than the real mean ΔF/F. In-field-periods were defined as three or more adjacent bins (except at the beginning and end of the track where two adjacent bins were sufficient) whose 1-*p*value ≥ 0.8 and for which at least 20% of the runs contained significant calcium transients within the period. Out-of-field periods were defined as two or more bins whose 1-*p*value ≤ 0.25. Bins with intermediate mean ΔF/F remained unassigned.

Grid cells were further identified based on a classifier with the following criteria^26,29,30^. (1) A grid cell must have at least two spatial fields on a track. (2) The response of grid cells must have a number of transitions between an in-field and out-of-field period for a track of length L larger than L/(5w), where w is the mean field width of the response. (3) The widest field of the response must be smaller than 5w. (4) At least 30% of the bins must be assigned to either in-field or out-of-field periods. (5) The mean ΔF/F of in-field periods divided by the mean ΔF/F of out-of-field periods must be larger than 2.

Finally, we removed cue cells, which were identified in the same session, from grid cell population. To minimize the effect of cue-cell-like activity on grid cell activity in landmark encoding, we removed cue cells with cue scores above 80^th^ percentile of shuffles (the same threshold used Data Analysis - Cue cells across multiple sessions).

#### Grid cells across two sessions

Grid cells on days 1 and 7 in the same track must be the ones identified as grid cells in both sessions based on the above criteria.

#### Activity variation for a pair of landmarks

For the activity variation of a cue cell at a pair of landmarks, we first averaged the mean spatially binned ΔF/F of the cell within the landmark zones for the two landmarks as A1 and A2, and the activity variation was calculated as the absolute difference of A1 and A2 normalized by their sum.

#### Averaged activity variation per cell

Averaged activity variation was calculated per cell by averaging its activity variations for a certain landmark type (i.e., identical or disparate landmark pairs) at all matched distances.

#### Activity variation difference

Activity variation difference was calculated as the activity variation for disparate landmark pairs subtracted by that for identical landmark pairs. The difference is calculated for landmark pairs at individual matched distances.

#### Activity variation difference per cell

Activity variation difference was calculated per cell by subtracting its averaged activity variation at identical landmarks from that at disparate landmarks.

#### Absolute reward distance

Absolute reward distance was the average of the magnitudes of the displacements between each of the two landmarks and the water reward for any landmark pair.

#### Rank correlation for a pair of landmarks

We calculated rank correlation of simultaneously imaged cue cells and grid cells in the same FOV. Since the variations in the numbers of the cells in individual FOVs could complicate the results, our calculation always included a fixed number (*N*) of cells randomly selected from each FOV. Only the FOVs with at least *N* + 1 cells were involved in the calculation. Based on the numbers of cells in individual FOVs in each condition, we used a specific *N* for each condition so that at least half of the imaging FOVs were included in the analysis. *N* = 15 for cue cells on day 1 of a track (Fig. 3), for common cue cells on pairs of tracks (Fig. 5), for common cue cells on days 1 and 7 of a track (Fig. 6). *N* = 5 for before-cue or after-cue cells on days 1 and 7 in a track (Fig. S21). To further demonstrate that our conclusions were independent of the size of cue cell groups, for the last panels in Figs. S6 and S15, the size of cue cell groups varied from 2 to 20, and for the last panel of Fig. S22, the size of cue cell groups varied from 2 to 22 (23 could not be used in order to maintain at least half of the FOVs in the calculation for both tracks).

Individual cell groups were selected randomly, and the number of groups equaled the number of total cells (M) in the FOV. To make this calculation, for each cell A in the FOV, we make one random selection of *N* number of cells in the remaining cells (the population excluding A). We repeated this step M times.

For a pair of landmarks (landmarks 1 and 2), the rankings of the *N* cells, R1 and R2, were calculated based on their averaged mean ΔF/F within the landmark zone of landmark 1 and landmark 2, respectively. We further calculated the correlation of C1 and C2 as the rank correlation.

The same calculation was applied to grid cells based on their spatially binned ΔF/F in landmark zones, which were generated during the cue score calculation (see Data Analysis - Cue cells in one session).

#### Averaged rank correlation per cell group

Averaged rank correlation was calculated per cell group by averaging its rank correlations for a certain landmark type (i.e., identical or disparate landmark pairs) at all matched distances.

#### Rank correlation difference

Rank correlation difference was calculated as the rank correlation for disparate landmark pairs subtracted by that for identical landmark pairs. The difference is calculated for landmark pairs at individual matched distances.

#### Rank correlation difference per cell group

Rank correlation difference was calculated per cell group by subtracting its averaged activity variation at identical landmarks from that at disparate landmarks.

#### Track change in activity variation and rank correlation for landmark pairs at different distances

For the track change of activity variations at identical landmark pairs, we used the activity variation (per cell) on one track subtracted by that on another track at individual matched distances. The track change of activity variation at disparate landmark pairs was similarly calculated. The track changes in rank correlation at different distances for identical and disparate landmark pairs were similarly calculated.

#### Learning change in activity variation and rank correlation for landmark pairs at different distances

For the learning change of activity variations at identical landmark pairs, we used the activity variation (per cell) on day 7 subtracted by that on day 1 at individual matched distances. The learning change of activity variation at disparate landmark pairs was similarly calculated. The learning changes in rank correlation at different distances for identical and disparate landmark pairs were similarly calculated.

#### Integrated activity

Integrated activity is the mean activity for each cell within a range (activity range) spanning both sides of each landmark’s midline.

#### Integrated activity variation

Integrated activity variation was calculated by taking the absolute value of the difference in integrated activity at two landmarks and dividing by their sum.

#### Integrated activity variation difference

Integrated activity variation difference was the integrated activity variation of disparate landmark pairs subtracted by the integrated activity variation of identical landmark pairs. The difference was calculated for landmark pairs at individual matched distances.

#### Mean integrated activity variation difference

The mean integrated activity variation difference was the average of the integrated activity variation difference for a sample aggregated from all cells and all matched distances.

### Visualization

#### Spatial activity of many cells as a function of track bin

Activity matrices have each cell’s activity normalized by its mean activity across all track bins.

### General analysis statistics

Image processing was performed using previously published MATLAB codes as cited above. The difference between two curves representing activity parameters at matched distances was calculated using two-way ANOVA. Bonferroni-Holm p-value correction was conducted as specified. The comparison between two pairs of conditions was conducted using two-tailed Student’s t test. Linear correlations were calculated using a two-tailed Pearson’s linear correlation coefficient. *P*-values less than 0.05 were considered significant (* < 0.05, ** <0.01, *** <0.001). All figures show mean and standard error, except where noted.

### Reporting summary

Further information on research design is available in the Nature Portfolio Reporting Summary linked to this article.

## Supplementary information

Supplementary Information

Reporting Summary

Transparent Peer Review file

## Source data

Source data

## Data Availability

Data generated in this study have been deposited in Zenodo [10.5281/zenodo.19115394]. Processed data underlying all figures are available in the Source Data file. Further information and requests for resources and reagents should be directed to the lead contact, Y.G. (yi.gu@nih.gov). Source data are provided with this paper.

## Code availability

Custom code used for the analysis of data is available on GitHub [https://github.com/GuLab-NIH/Wang-and-Shahid-2026].
