# Supplementary material for: The entorhinal spatial map integrates visual identity information of landmarks
Source: Nat Commun. 2026 May 7;17:6164. doi: 10.1038/s41467-026-72453-1 (PMC13365227; doi:10.1038/s41467-026-72453-1)
Supplement: Supplementary file 1 — Supplementary Information [file 41467_2026_72453_MOESM1_ESM.pdf]

Supplementary Information for

The entorhinal spatial map integrates visual identity information of landmarks

Garret Wang[1, 2, *], Farid Shahid[1, *], Taylor J. Malone[1], Jean Tyan[1, 3], Kyle Cekada[1, 4], Lujia Chen[1], Yi Gu[1, †]

[†]Corresponding author: yi.gu@nih.gov

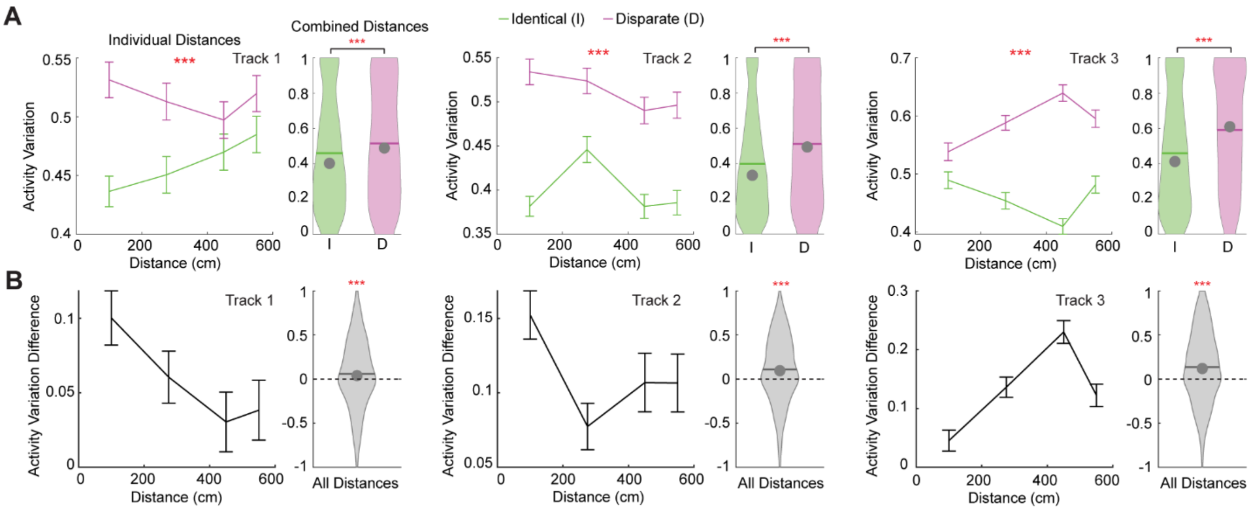

Figure S1. Individual cue cells from all three tracks encode landmark identity.

A. Activity variation for identical (I, green) and disparate (D, magenta) landmark pairs from tracks 1-3 (left to right) at individual (left) and combined (right) matched distances. The asterisks in the left panels indicate the results of two-way ANOVA comparisons for I and D curves. The asterisks in the right panels indicate the results of two-tailed paired t-tests.

B. Activity variation difference in tracks 1-3 at individual (left) and combined (right) distances. The asterisks in the right panels indicate the results of two-tailed paired t-test comparisons of activity variation difference with zero.

*p < 0.05, **p < 0.01, ***p < 0.001, n.s. p ≥ 0.05. Same for all figures. Statistical information including exact p-values are available in Supplementary Table 1. Violin plots have dot and horizontal bar representing median and mean, respectively. Other data are presented as mean ± SEM. Data were from 5 mice, with 434, 465, and 508 cells from tracks 1, 2, and 3, respectively.

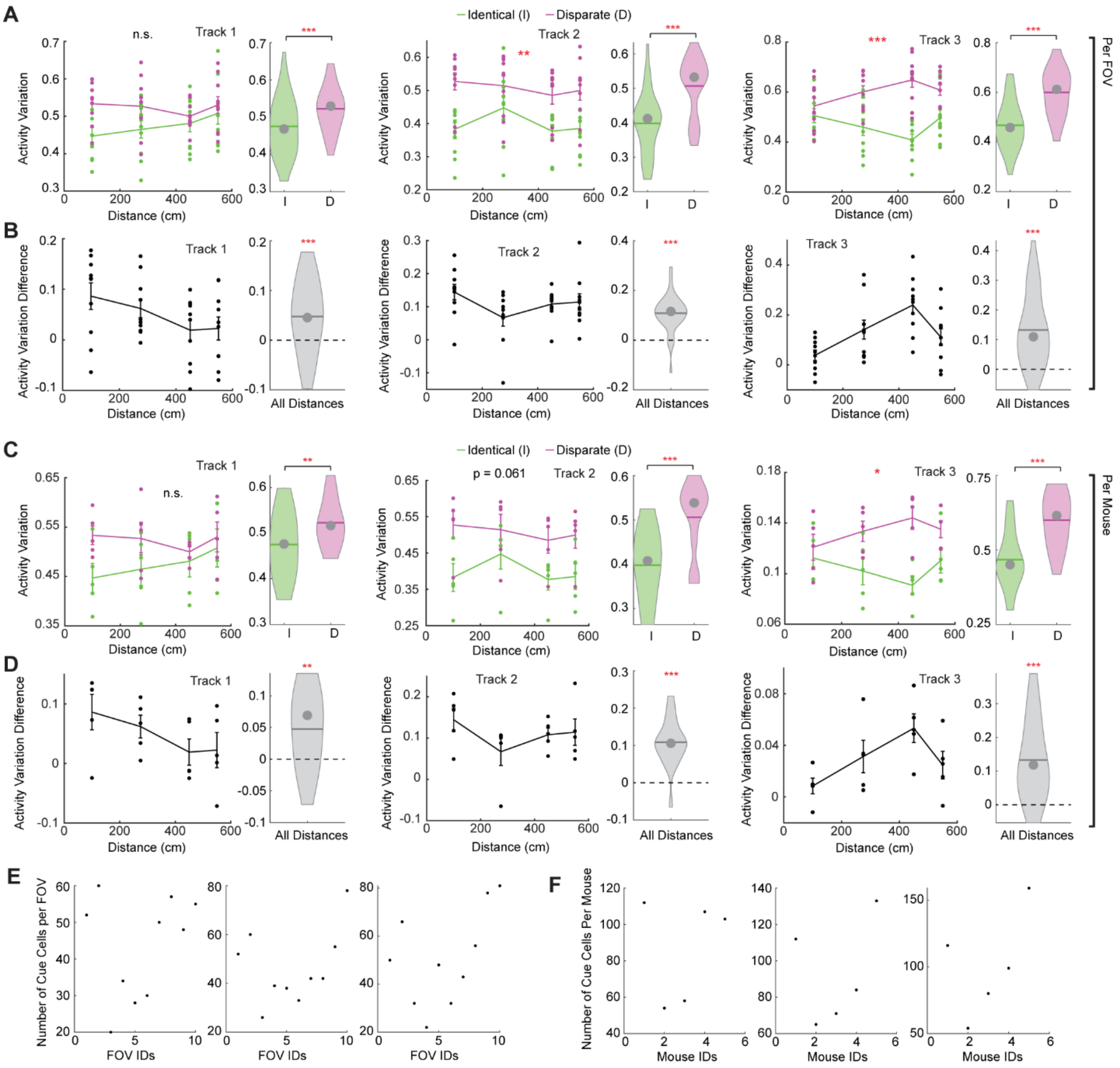

Figure S2. Individual cue cells grouped by imaging field of view (FOV) and by mouse encode landmark identity.

A. Per FOV: activity variation for identical (I, green) and disparate (D, magenta) landmark pairs in tracks 1 to 3 (left to right). These data were generated by grouping those in Fig. S1 by FOV. The asterisks in the left panels indicate the results of two-way ANOVA comparisons for I and D curves. Individual dots represent each FOV at each distance. The asterisks in the right panels indicate the results of two-tailed paired t-tests.

B. Per FOV: activity variation difference in the three tracks. Individual dots represent each FOV at each distance. The asterisks in the right panels indicate the results of two-tailed paired t-test comparisons of activity variation difference with zero.

C-D. Similar to A and B but per mouse. Individual dots represent each mouse at each distance. Statistical tests are the same as those of A and B.

E. Number of cue cells per FOV.

F. Number of cue cells per mouse.

Data were from 434, 465, and 508 cells from tracks 1, 2, and 3, respectively, which were averaged on a per FOV or per mouse basis. Violin plots have dot and horizontal bar representing median and mean, respectively. Other data are presented as mean ± SEM. Statistical information is available in Supplementary Table 1.

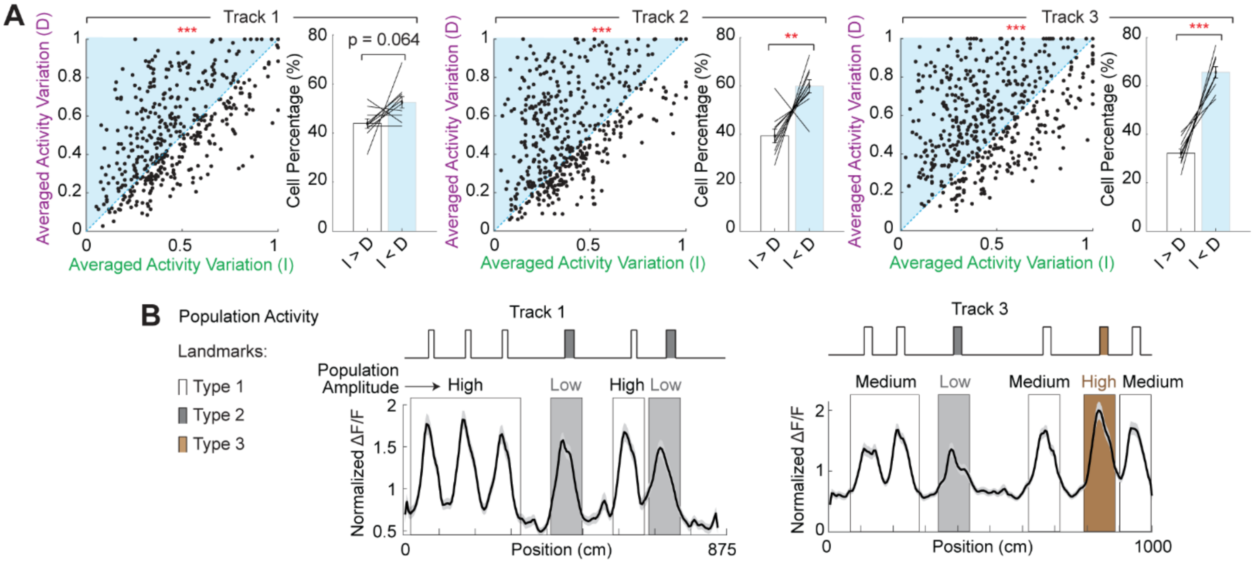

Figure S3. Activity variation of cue cells in individual tracks better discriminate disparate landmarks than identical ones, leading to differential averaged activity for landmarks of different identity.

A. For each track, Left: averaged activity variations for identical landmark pairs (I) versus those for disparate pairs (D) of individual cells. Asterisks represent p-values of the two-tailed Pearson's linear correlations of the averaged activity variation coordinates for all cells. Blue and white zones include cells with larger variations for disparate and identical landmark pairs, respectively. Right: the percentage of cells per FOV with larger activity variations for identical (white) or disparate (blue) landmark pairs. Asterisks represent results of two-tailed paired t-tests.

B. Cue cell population activity on day 1 of tracks 1 and 3. The activity for each landmark is highlighted by a box with its landmark color; its relative amplitude is indicated above. Population activity is high, low, or medium for landmarks of different types.

Data were from 5 mice, with 434, 465, and 508 cells from tracks 1, 2, and 3, respectively. Violin plots have dot and horizontal bar representing median and mean, respectively. Other data are presented as mean ± SEM. Statistical information is available in Supplementary Table 1.

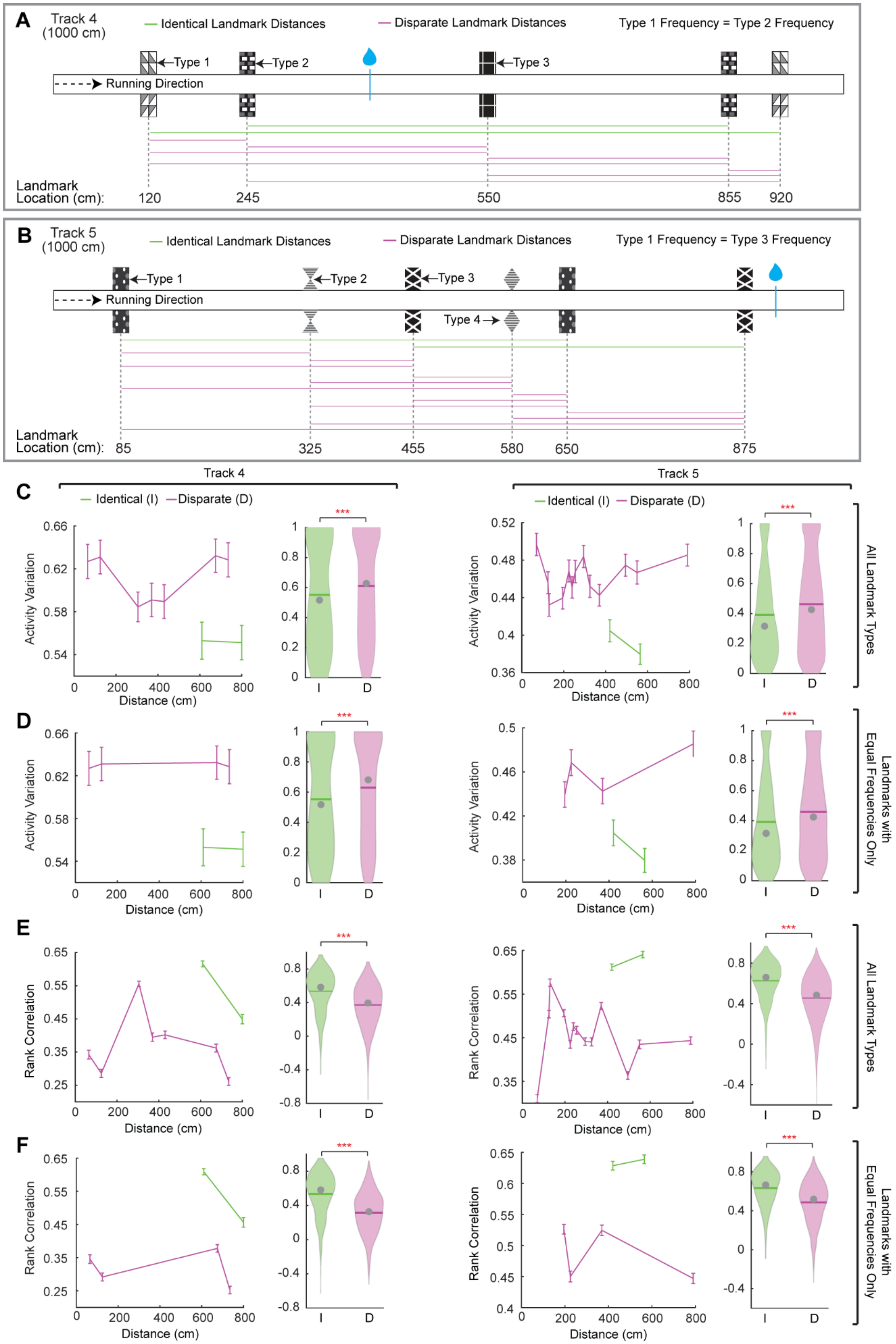

A
Track 4 (1000 cm)
Identical Landmark Distances
Disparate Landmark Distances
Type 1 Frequency = Type 2 Frequency
Type 1
Type 2
Type 3
Running Direction
Landmark Location (cm): 120 245 550 855 920
B
Track 5 (1000 cm)
Identical Landmark Distances
Disparate Landmark Distances
Type 1 Frequency = Type 3 Frequency
Type 1
Type 2
Type 3
Type 4
Running Direction
Landmark Location (cm): 85 325 455 580 650 875
Track 4
Track 5
C
D
E
F
Identical (I)
Disparate (D)
Activity Variation
Rank Correlation
Distance (cm)
I
D
***
All Landmark Types
Landmarks with Equal Frequencies Only

Figure S4. Differential activity patterns between disparate and identical landmark pairs remain for landmarks with equal frequency-based salience.

A. The design of track 4 with landmark types 1 and 2 having equal frequency.

B. The design of track 5 with landmark types 1 and 3 having equal frequency.

C. Activity variation for all identical (I, green) and disparate (D, magenta) landmark pairs at individual and combined distances for tracks 4 and 5. Asterisks indicate results of two-tailed non-paired t-tests.

D. Similar to C, except that the analysis was restricted to landmarks of equal frequency. Statistical tests are the same as those in C.

E-F. Similar to C and D but for rank correlation. Statistical tests are the same as those of C and D. Each cue cell group contains 15 cells.

Track 4 activity variation data were from 11 mice and 498 cells. Track 4 rank correlation data were from 9 mice and 404 cell groups. Track 5 data were from 6 mice and 670 cells and cell groups. Violin plots have dot and horizontal bar representing median and mean, respectively. Other data are presented as mean ± SEM. Statistical information is available in Supplementary Table 1.

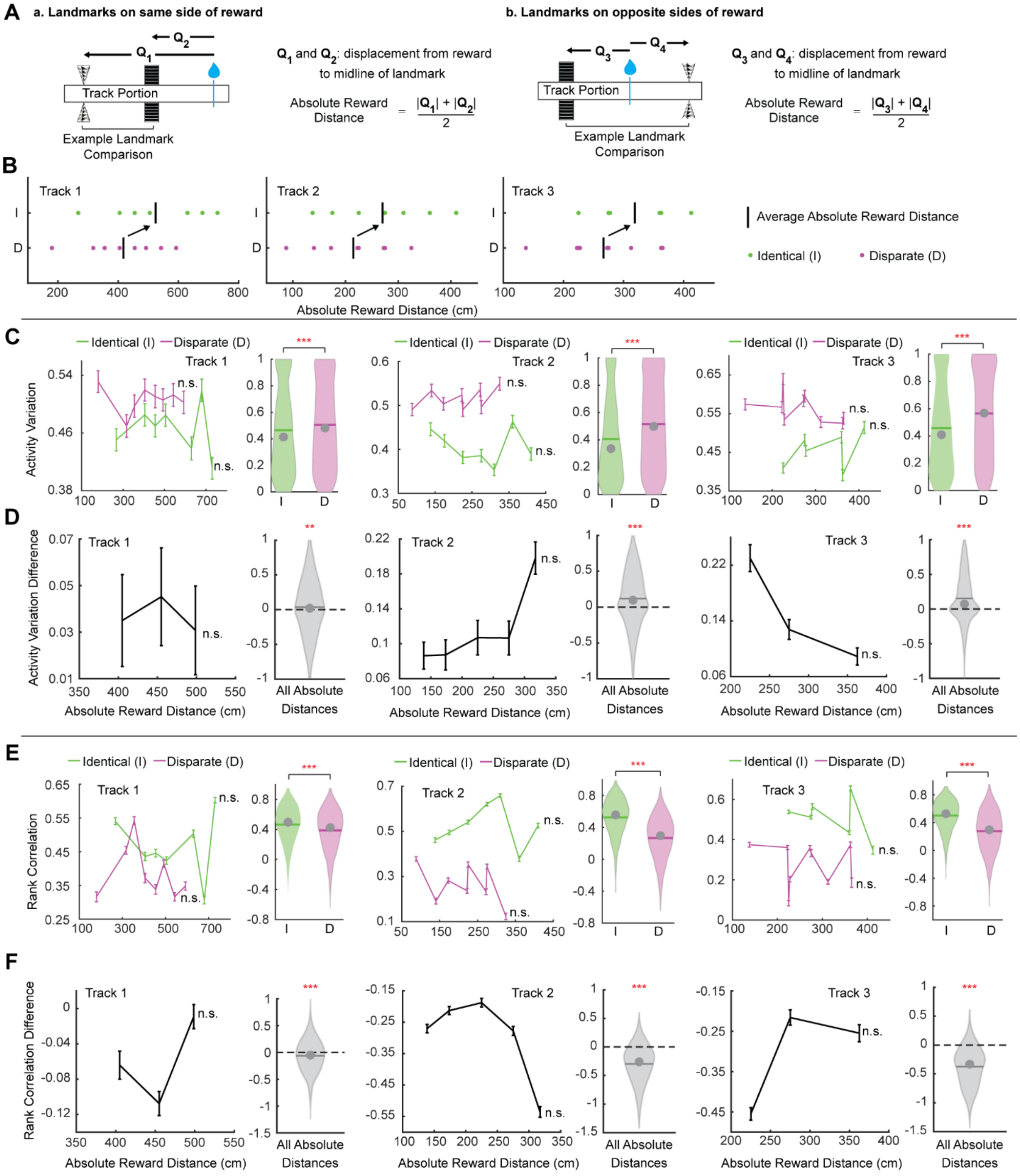

Figure S5. Landmark identity encoding is not modulated by distance to the reward.

A. Schematic for absolute reward distance of two landmarks on the same (**a**) or opposite sides (**b**) of the reward. Q1-Q4 represent displacements from the reward to the midlines of individual landmarks.

B. Absolute reward distance for identical (I, green) and disparate (D, magenta) landmark pairs, with identical landmark pairs being slightly further from the reward than disparate ones.

C. Activity variation for identical and disparate landmark pairs at individual (left) and combined (right) absolute reward distances in tracks 1 to 3. Non-significant results next to I and D curves indicate the two-tailed Pearson's linear correlation of averaged activity variations at individual distances with the absolute reward distances, demonstrating no relationship between landmark encoding and distance from the reward. Asterisks indicate the results of two-tailed non-paired t-tests.

D. Similar to C, but for activity variation difference. For those absolute reward distances of identical landmark pairs with no matching absolute reward distances for disparate landmarks, we included the closest data point within 15 cm, if available, to conduct the difference calculation. When the distances did not exactly match, we used the average of the two distances. Non-significant results next to activity variation difference curves indicate the two-tailed Pearson's linear correlation of averaged activity variation differences at individual distances with the absolute reward distances, demonstrating no relationship between landmark identity encoding and distance from the reward. Insignificant trends are inconsistent across tracks. Asterisks indicate the results of two-tailed paired t-test comparisons with zero.

E-F. Similar to C and D, but for rank correlation and rank correlation difference. Statistical tests are the same as those in C and D. Each cue cell group contains 15 cells.

Data were from 5 mice, with 434, 465, and 508 cells and cell groups from tracks 1, 2, and 3, respectively. Violin plots have dot and horizontal bar representing median and mean, respectively. Other data are presented as mean ± SEM. Statistical information is available in Supplementary Table 1.

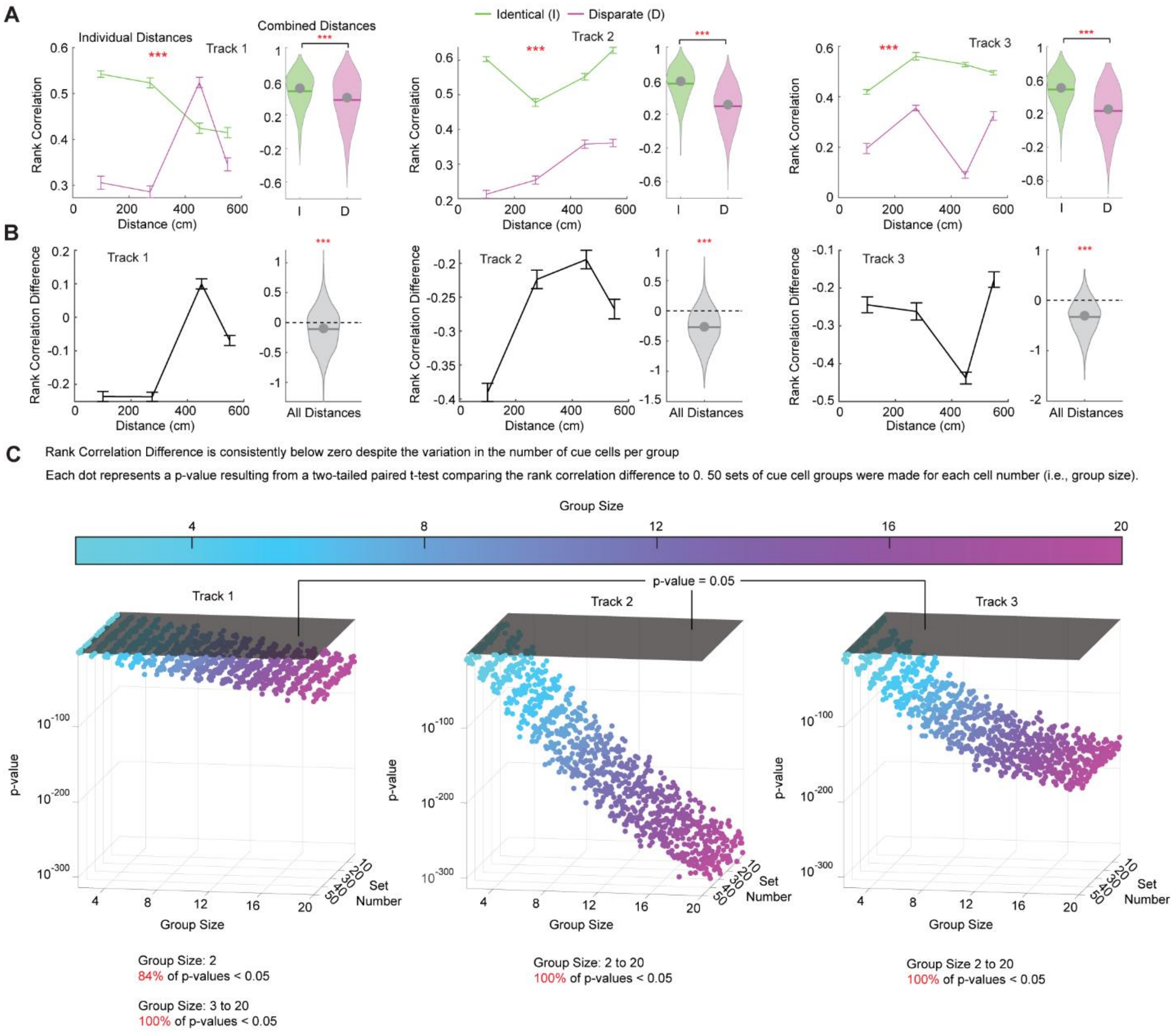

Figure S6. Populations of cue cells from all three tracks robustly encode landmark identity.

A. Rank correlation for identical (I, green) and disparate (D, magenta) landmark pairs in tracks 1-3 at individual (left) and combined (right) distances. Each cue cell group contains 15 cells. The asterisks in the left panels indicate the results of two-way ANOVA comparisons between the I and D curves. The asterisks in the right panels indicate the results of two-tailed paired t-tests.

B. Rank correlation difference in the three tracks. The asterisks in the right panels indicate the results of two-tailed paired t-test comparisons of rank correlation difference with zero.

C. The comparison between rank correlation differences and zero calculated using cue cell groups of different numbers of cells (group size is from 2 to 20). Each dot indicates the p-value for the comparison between rank correlation difference and zero of a set of cue cell groups for each track. Since each calculation was based on cue cell groups randomly chosen from individual FOVs, 50 calculations were made based on 50 sets of cue cell groups for each group size. The individual p-values were color coded according to the number of cue cells in the group and shown on a logarithmic scale in comparison to a significance level of 0.05. With the exception

of a group size of 2 in track 1, all p-values across tracks and group sizes were below the significance level.

Data in panels A and B were from 5 mice, with 434, 465, and 508 cell groups from tracks 1, 2, and 3, respectively. In panel C, the number of FOVs from all 5 mice that were included in the analysis varied based on the number of cells per group (see Data Analysis - Rank correlation for a pair of landmarks in Methods). Violin plots have dot and horizontal bar representing median and mean, respectively. Other data are presented as mean ± SEM. Statistical information is available in Supplementary Table 1.

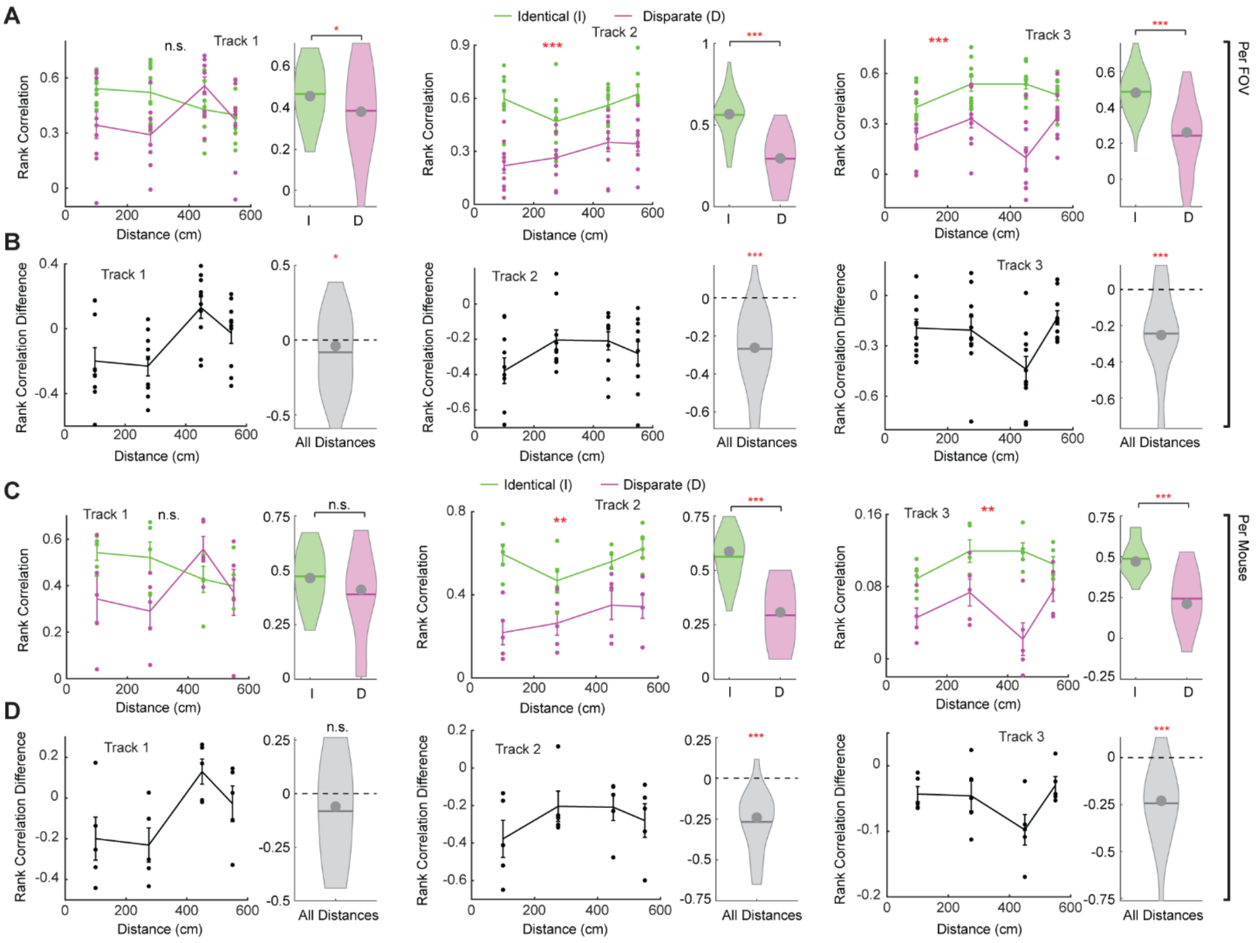

Figure S7. Populations of cue cells grouped by imaging FOV and by mouse encode landmark identity.

A. Per FOV: rank correlation for identical (I, green) and disparate (D, magenta) landmark pairs in tracks 1-3 (left to right). These data were generated by grouping those in Fig. S6A by FOV. The asterisks in the left panels indicate the results of two-way ANOVA comparisons between the I and D curves. Individual dots represent each FOV at each distance. The asterisks in the right panels indicate the results of two-tailed paired t-tests.

B. Per FOV: rank correlation difference in the three tracks. Individual dots represent each FOV at each distance. The asterisks in the right panels indicate the results of two-tailed paired t-test comparisons of rank correlation difference with zero.

C-D. Similar to A and B, but per mouse. Individual dots represent each mouse at each distance. Statistical tests are the same as those in A and B.

Data were from 434, 465, and 508 cell groups from tracks 1, 2, and 3, respectively, which were averaged on a per FOV or per mouse basis. Violin plots have dot and horizontal bar representing median and mean, respectively. Other data are presented as mean ± SEM. Statistical information is available in Supplementary Table 1.

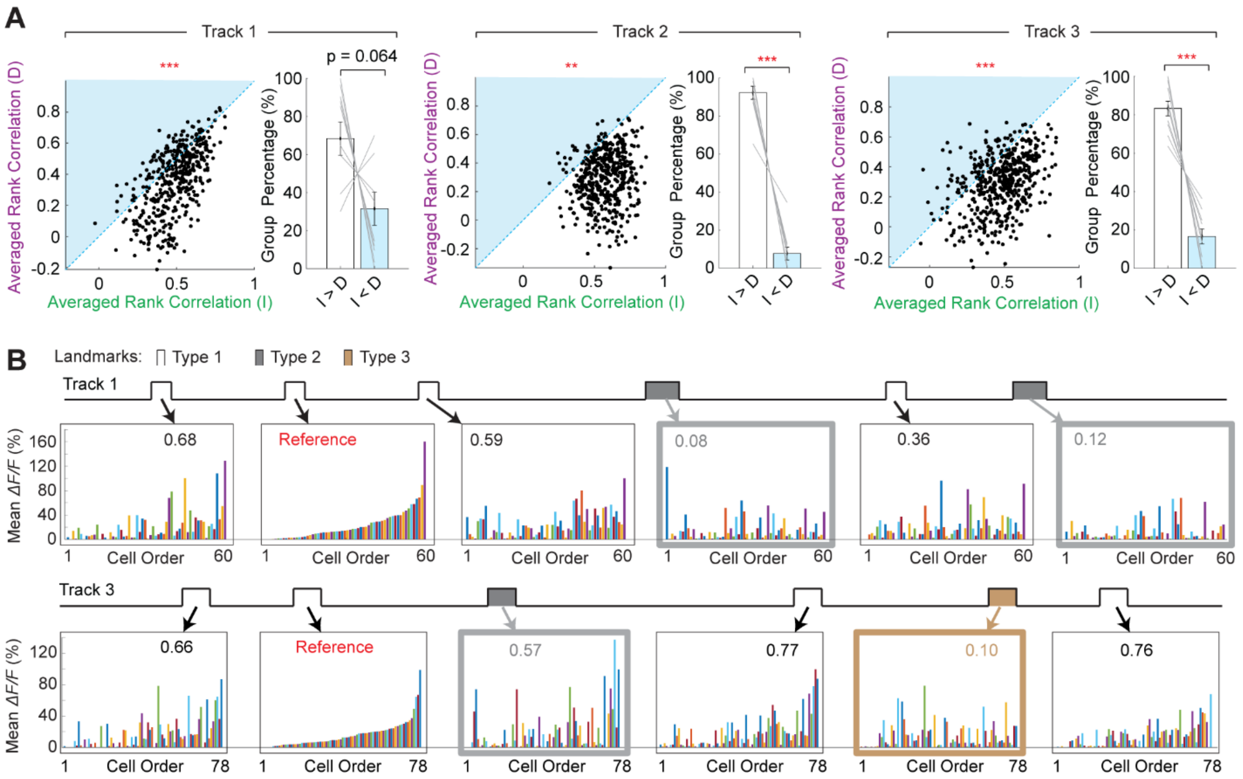

Figure S8. Rank correlations of cue cell populations in individual tracks tend to better discriminate disparate compared to identical landmarks.

A. For each track: left: averaged rank correlations for identical landmark pairs (I) vs those for disparate pairs (D) for individual cell groups. Asterisks represent p-values of two-tailed Pearson's linear correlations of averaged rank correlation coordinates for all cell groups within each track. Blue and white zones include cell groups with larger rank correlations for disparate and identical landmark pairs, respectively. Right: the percentage of cell groups per FOV with larger rank correlations for identical (white) or disparate (blue) landmark pairs. Asterisks represent results of two-tailed paired t-tests.

B. Visualization of the activity of simultaneously imaged cue cells in the same FOV from track 1 and 3 at individual landmarks. Cue cells were ranked ascendingly based on their landmark activity at the reference landmark and their activity at other landmarks was plotted with the same ranking. Rank correlations for these cells between each landmark and the reference are shown at the top of each landmark box. A higher rank correlation corresponds to a more consistent trend of activity ascent at a landmark compared to the reference, indicating that the relative activities of the cells between the two landmarks are more similar.

Data in panel A were from 5 mice, with 434, 465, and 508 cell groups from tracks 1, 2, and 3, respectively. Data in panel B were from 60 and 78 cells obtained from single FOVs in different mice for track 1 and 3. Data are presented as mean ± SEM. Statistical information is available in Supplementary Table 1.

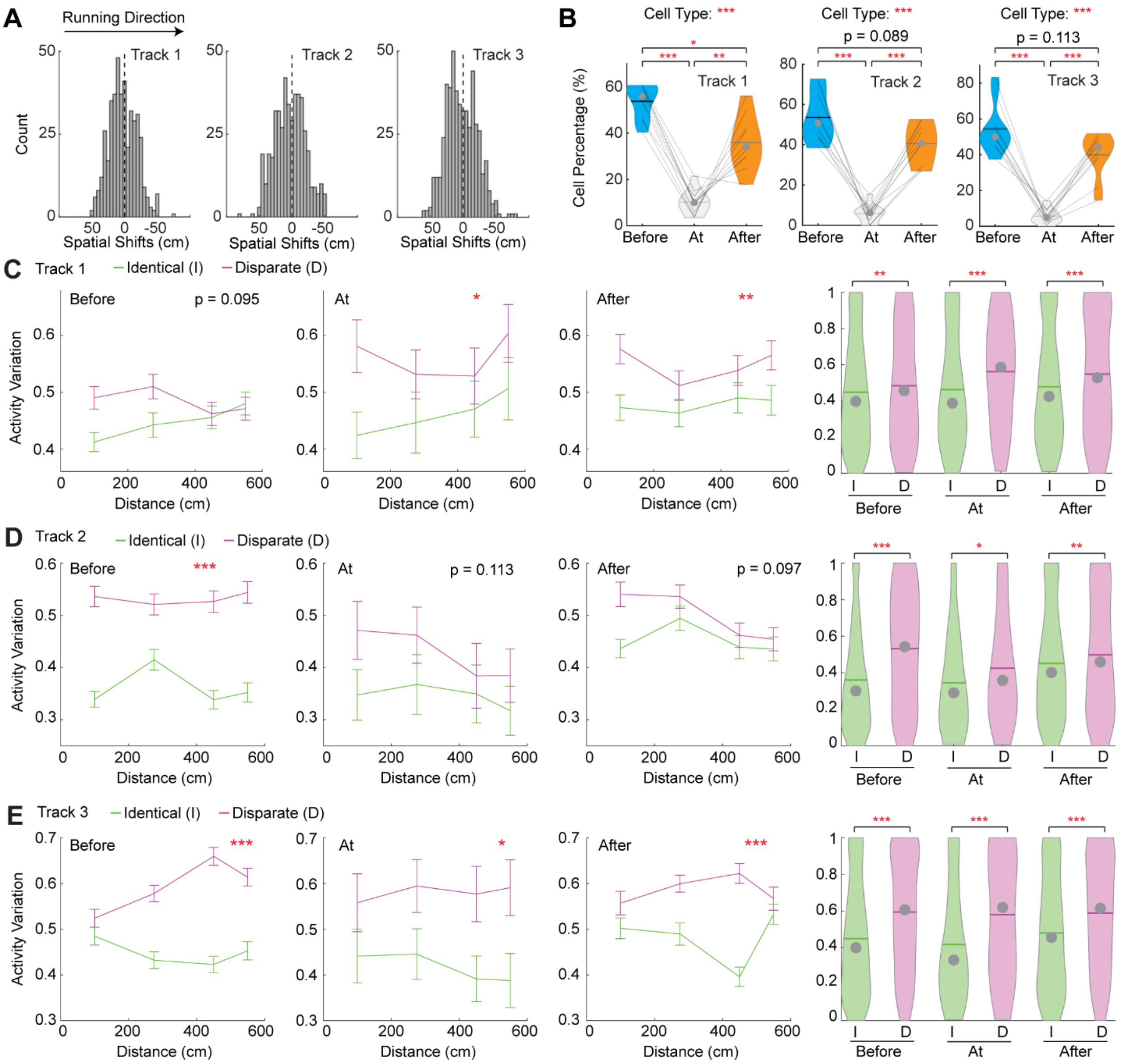

Figure S9. Landmark identity encoding of cue cells in individual tracks is modulated by spatial shifts.

A. Spatial shifts of cue cells in each track largely fell within ± 50 cm.

B. The percentages of before-cue (Before), at-cue (At), and after-cue (After) cells in each FOV for tracks 1-3. The same FOVs are connected with gray lines. Asterisks for Cell Type represent p-values of one-way ANOVA comparisons of before, at, and after-cue cells' percentages within each FOV. Comparisons between samples are two-way paired t-tests with Bonferroni-Holm p-value correction within each track.

C. Track 1: activity variation of before-cue, at-cue, and after-cue cells for identical (I, green) and disparate (D, magenta) landmark pairs at individual (panels 1-3 from left to right) and combined (panel 4) distances showing consistent landmark identity encoding or a trend to this effect regardless of spatial shift region. First three panels' statistics are two-way ANOVA

comparisons between I and D curves with Bonferroni-Holm correction. Panel 4 shows two-tailed paired t-tests with Bonferroni-Holm correction.

D. Similar to C, but for track 2. Statistical tests are the same as those of C.

E. Similar to C, but for track 3. Statistical tests are the same as those of C.

Data were from 5 mice, with 434, 465, and 508 cells from tracks 1, 2, and 3, respectively. Violin plots have dot and horizontal bar representing median and mean, respectively. Other data are presented as mean ± SEM. Statistical information is available in Supplementary Table 1.

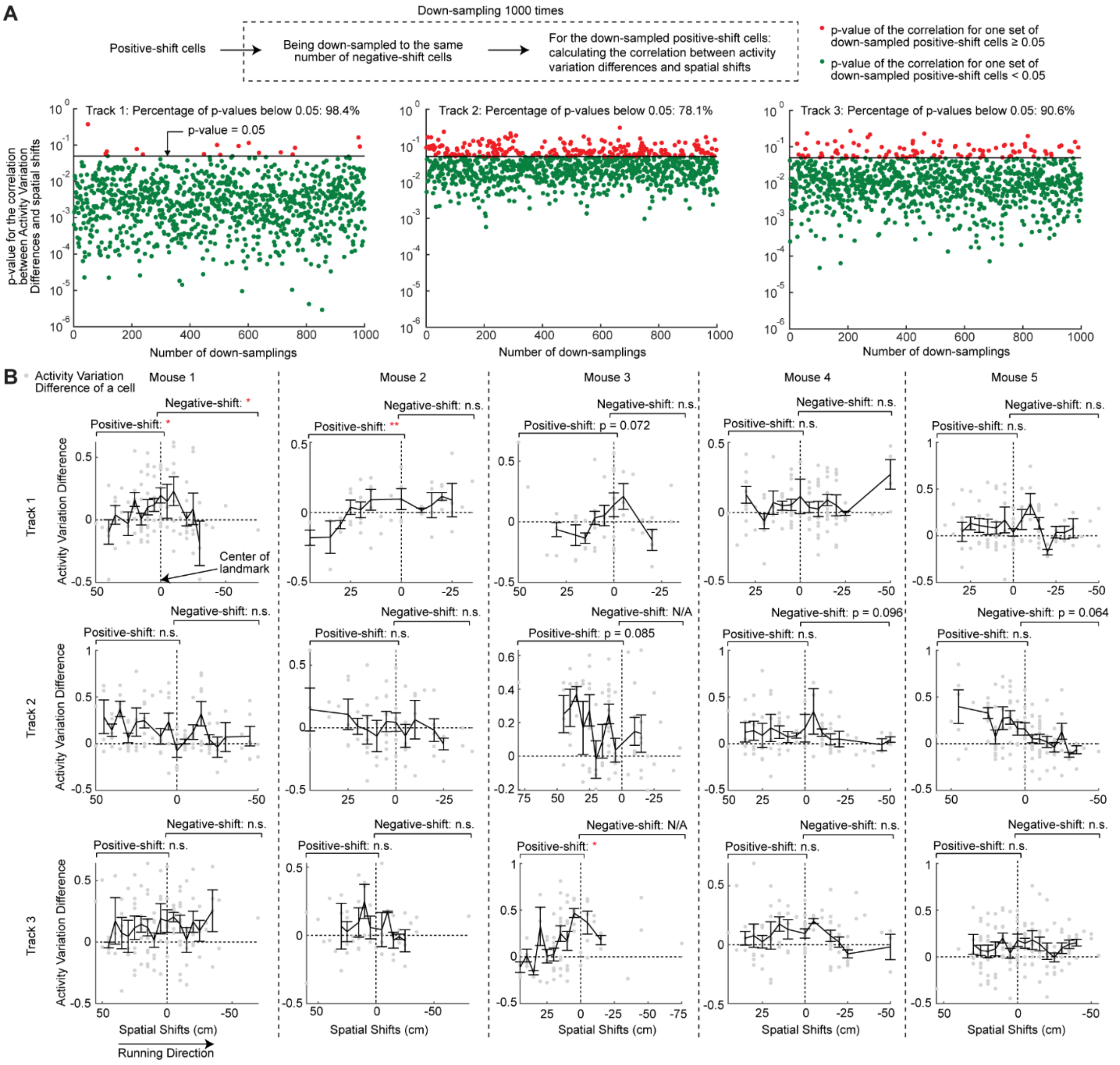

Figure S10. Activity variation difference is robustly modulated by positive spatial shifts.

A. The significant correlation between activity variation differences and absolute spatial shifts of positive-shift cells is unlikely due to the larger number of positive-shift cells compared to negative-shift cells. Each dot represents the p-value of the correlation for one calculation out of 1000 for each track in which positive-shift cells were down-sampled to match the number of negative-shift cells. This calculation was a two-tailed Pearson's linear correlation between the averaged activity variation difference and the absolute spatial shifts that have at least 5 cells. Red dots indicate insignificant p-values, while green dots indicate significant p-values relative to a significance level of 0.05. For all three tracks, a majority of p-values are below 0.05.

B. For individual mice: activity variation differences of individual cue cells (gray dots) as functions of their spatial shifts. Two-tailed Pearson's linear correlation p-values are shown for

positive-shift and negative-shift cells' averaged activity variation difference relative to absolute spatial shifts with at least 3 cells. The threshold for including a spatial shift in the analysis is reduced due to the fewer number of cells in each mouse compared to all mice together as is the case in A.

Data were from 5 mice, with 434, 465, and 508 cells from tracks 1, 2, and 3, respectively. Data are presented as mean ± SEM. Statistical information is available in Supplementary Table 1.

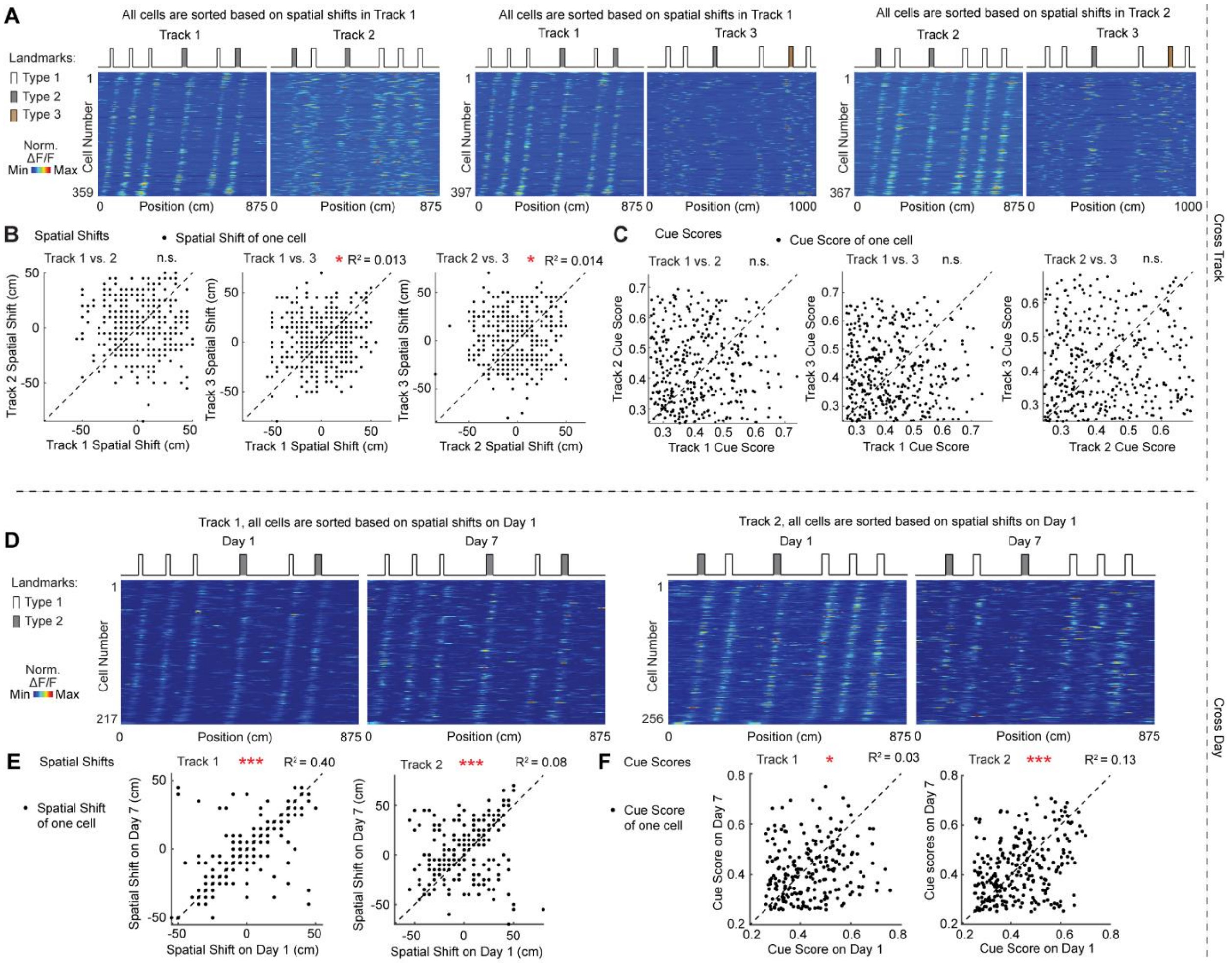

Figure S11. Cue cells common to different tracks show limited correlation in their spatial shifts and cue scores, whereas spatial shifts and cue scores for cue cells common to days 1 and 7 show strong correlations.

A. Activity of common cue cells in tracks 1 and 2, 1 and 3, as well as 2 and 3. All cells are sorted by their spatial shifts from the former track in each track-pair comparison, with weak or no obvious recreation of the characteristic pattern in the latter track. Hotter colors indicate higher normalized activity; same for all analogous visualizations.

B. The two-tailed Pearson's linear correlation between spatial shifts of individual common cue cells in different tracks is low. R^2 represents the fits of the data to the diagonal dashed lines, indicating weak similarity in shifts between tracks. Statistical tests are two-tailed Pearson's linear correlations between spatial shifts in different tracks.

C. Similar to B but showing no significant Pearson correlation of cue scores in track-pair comparisons. Statistical tests are two-tailed Pearson's linear correlations between cue scores of the same cells in different tracks.

D. Activity of common cue cells on days 1 and 7 of tracks 1 and 2. All cells are sorted by their spatial shifts from day 1.

E. Spatial shifts of cue cells on different days are well correlated. Statistical information comes from two-tailed Pearson's linear correlations between spatial shifts on different days.

F. Similar to E but showing strong correlation of cue scores between days 1 and 7 in both tracks. Asterisks represent the p-values of the Pearson correlations between cue scores of the same cells on day 1 and day 7.

Data were from 5 mice with day 1 track pairs 1 and 2, 1 and 3, as well as 2 and 3 each having 359, 397, and 367 cells, respectively. Track 1 data included 217 cells common to day 1 and day 7. Track 2 data included 256 cells common to day 1 and day 7. Statistical information is available in Supplementary Table 1.

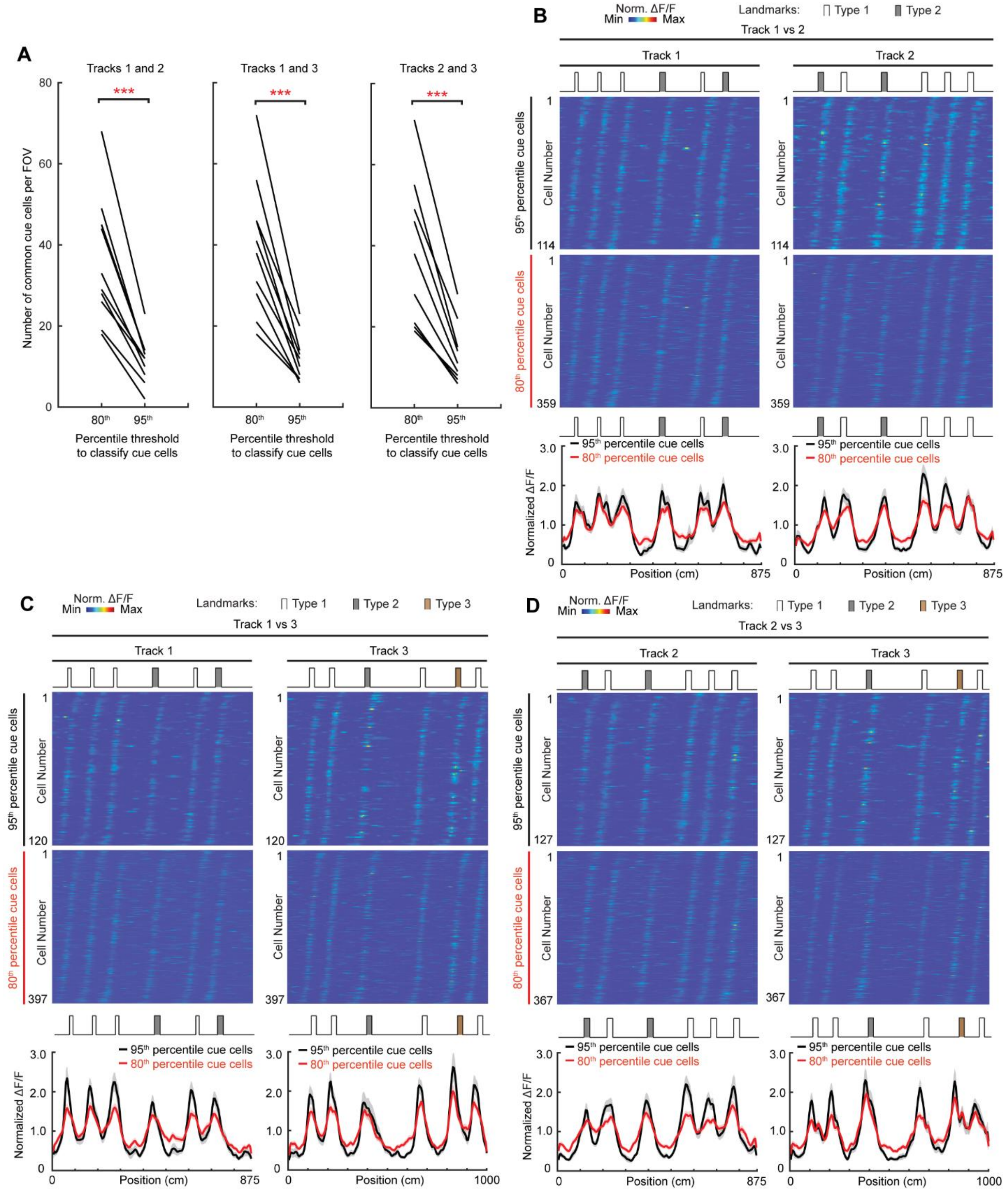

Figure S12. Comparison of common cue cells across different tracks, classified using the 80th and 95th percentile thresholds of the shuffled distribution.

A. Number of common cue cells across track pairs under the different thresholds. Asterisks indicate the results of two-tailed paired t-tests. Note that the 80th percentile threshold produces more cells.

B. The activity of common cue cells on tracks 1 and 2 classified using the two thresholds. Top heat plots: cue cell activity patterns sorted by their spatial shifts in each track. Bottom: calcium activity of cue cell populations under the two thresholds. Note that both thresholds produce cue cells with strongly landmark-associated activity. Hotter colors indicate higher normalized activity; same for all analogous visualizations.

C. and **D.** Similar to B, but for tracks 1 and 3 (C), as well as 2 and 3 (D).

80th percentile cue threshold data were from 5 mice, including 359, 397, and 367 cells in track comparisons 1 and 2, 1 and 3, as well as 2 and 3, respectively. 95th percentile cue threshold data were from 5 mice, including 114, 120, and 127 cells in track comparisons 1 and 2, 1 and 3, as well as 2 and 3, respectively. Data are presented as mean ± SEM. Statistical information is available in Supplementary Table 1.

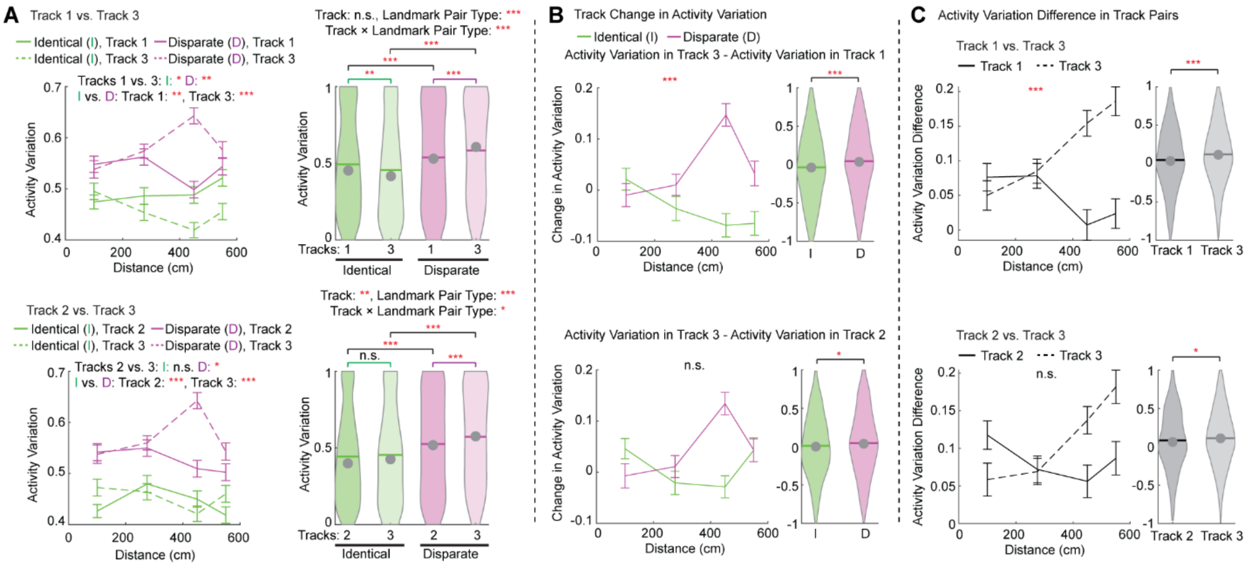

Figure S13. Cue cells encode landmark identity differently in different tracks, as determined by activity variation calculations.

A. Left: Activity variation of common cue cells in track 1 (solid line) vs 3 (dashed line) as well as track 2 (solid line) vs 3 (dashed line) for identical (I, green) and disparate (D, magenta) landmark pairs across distances. Statistics are two-way ANOVA comparisons with Bonferroni-Holm correction conducted separately for each track pair. Right: activity variation calculations for combined distances. Above statistics are within-track comparison two-way ANOVAs showing that track and landmark pair type jointly modulate activity variation. Post-hoc comparisons are two-tailed paired t-tests with Bonferroni-Holm correction conducted separately for each pairwise track comparison.

B. Track changes in activity variation for identical (green) and disparate (magenta) landmark pairs in tracks 1 and 3 as well as tracks 2 and 3 at individual (left) and combined (right) distances. Statistical tests are two-way ANOVA comparisons (left) and two-tailed paired t-tests (right).

C. Similar to B, but for activity variation difference in tracks 1 and 3 as well as tracks 2 and 3. Statistical tests are the same as those in B.

Data were from 5 mice, with 397 cells common to day 1 of track 1 and 3 as well as 367 cells common to day 1 of track 2 and 3. Violin plots have dot and horizontal bar representing median and mean, respectively. Other data are presented as mean ± SEM. Statistical information is available in Supplementary Table 1.

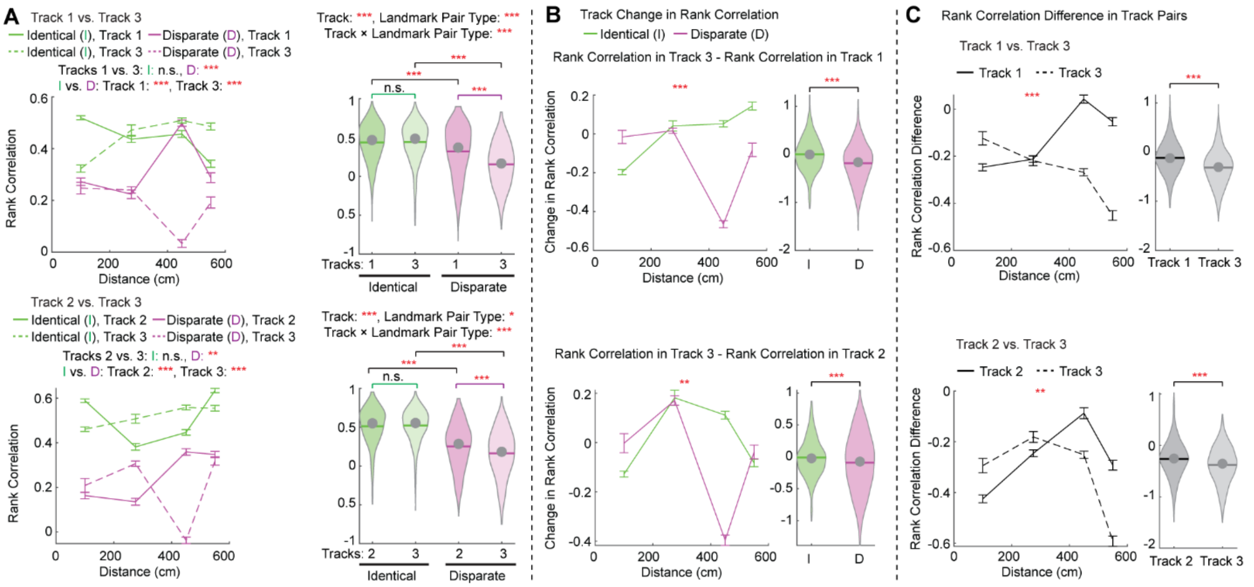

Figure S14. Populations of cue cells encode landmark identity differently in different tracks, as determined by rank correlation calculations.

A. Left: Rank correlation of common cue cells in track 1 (solid line) vs 3 (dashed line) as well as track 2 (solid line) vs 3 (dashed line) for identical (I, green) and disparate (D, magenta) landmark pairs across distances. Statistics are two-way ANOVA comparisons with Bonferroni-Holm correction conducted separately for each track pair. Right: rank correlation calculations for combined distances. Above statistics are within-track comparison Bonferroni-Holm-corrected two-way ANOVAs showing that track and landmark pair type jointly modulate rank correlation. Post-hoc comparisons were two-tailed paired t-tests with Bonferroni-Holm correction conducted separately for each pairwise track comparison. Each cue cell group contains 15 cells.

B. Track changes in rank correlation for identical (green) and disparate (magenta) landmark pairs in tracks 1 and 3 as well as tracks 2 and 3 at individual (left) and combined (right) distances. Statistical tests are two-way ANOVA comparisons (left) and two-tailed paired t-tests (right).

C. Similar to B, but for rank correlation difference in tracks 1 and 3 as well as tracks 2 and 3. Statistical tests are the same as those in B.

Data were from 5 mice, with 397 cell groups common to day 1 of track 1 and 3 as well as 367 cell groups common to day 1 of track 2 and 3. Violin plots have dot and horizontal bar representing median and mean, respectively. Other data are presented as mean ± SEM. Statistical information is available in Supplementary Table 1.

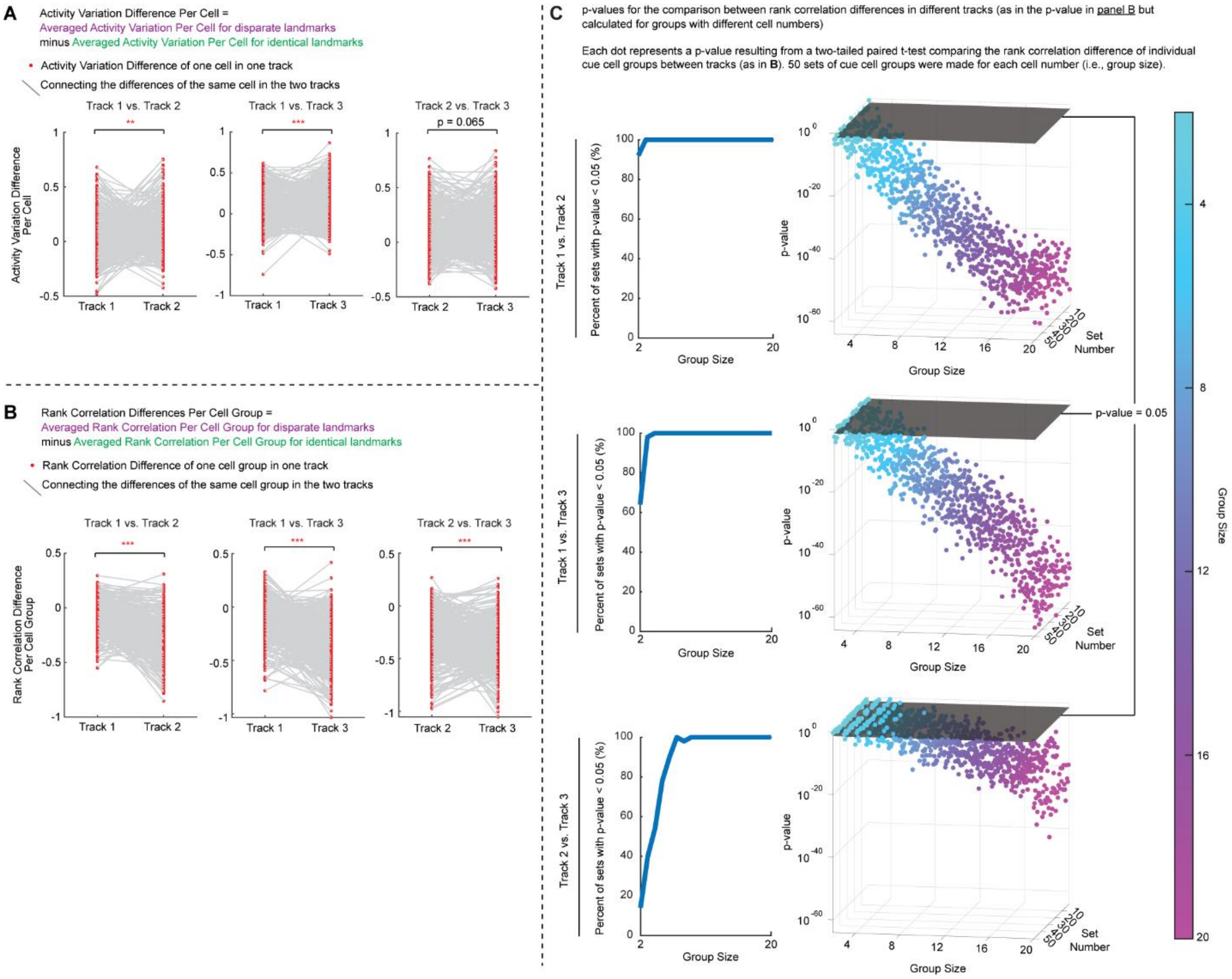

Figure. S15. Individual and groups of cue cells encode landmark identity differently in different tracks.

A. Activity variation difference in individual cells (i.e. averaged across all four matched distances for each cell) in each track pair (track 1 vs 2, track 1 vs 3, and track 2 vs 3). Individual cells generally show differential landmark identity encoding in different tracks. Statistical tests are two-tailed paired t-tests.

B. Rank correlation difference in individual cell groups (i.e. averaged across all four matched distances for each cell group) in each track pair (track 1 vs 2, track 1 vs 3, and track 2 vs 3). Individual cue cell groups show strong differential landmark identity encoding in different tracks. Statistical tests are two-tailed paired t-tests.

C. p-values for the comparisons between rank correlation differences of the same groups of common cells in different tracks when varying the group size. Each dot is the p-value for one calculation using one set of common cell groups, as in B. Since the cell groups were randomly sampled within each FOV, 50 calculations were made using 50 sets of common cell groups for each group size. The individual p-values were color coded according to the number of cue cells in the group and shown on a logarithmic scale relative to a significance level of 0.05. In all three track comparisons, most of the choices for group size have all 50 calculations showing significant differences in rank correlation difference between tracks.

Data in panels A and B were from 5 mice with day 1 track pairs 1 and 2, 1 and 3, as well as 2 and 3 each having 359, 397, and 367 cells and cell groups, respectively. In panel C, the number of FOVs from all 5 mice that were included in the analysis varied based on the number of cells per group (see Rank correlation for a pair of landmarks in Methods). Statistical information is available in Supplementary Table 1.

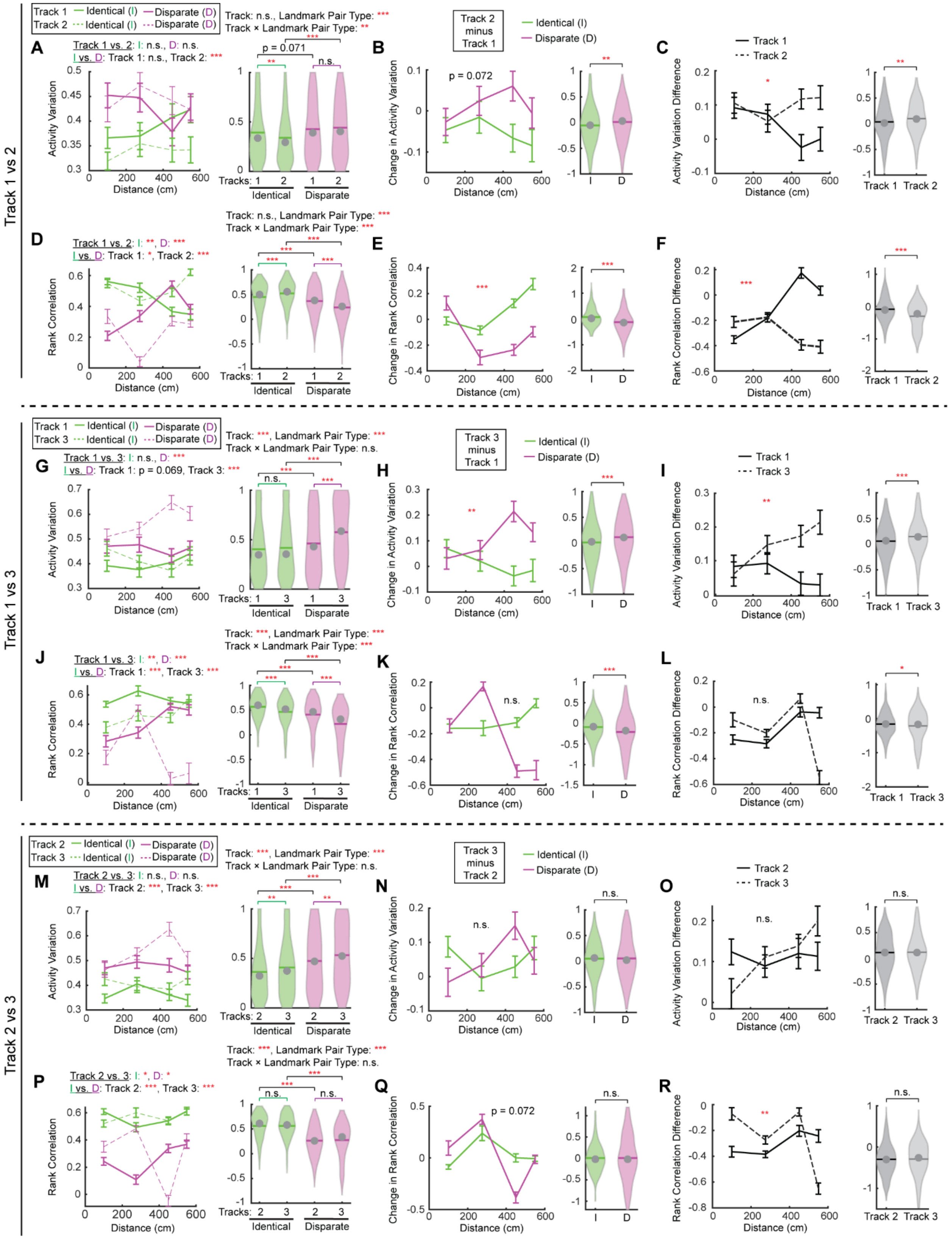

Fig. S16. 95th percentile cue cells largely show differential landmark identity encoding in different tracks.

A. Left: Activity variation of 95th percentile common cue cells in track 1 (solid line) versus track 2 (dashed line) for identical (I, green) and disparate (D, magenta) landmark pairs across distances. Statistics are two-way ANOVA comparisons with Bonferroni-Holm correction. Right: activity variation calculations for combined distances. Above statistics are two-way ANOVA comparisons. Post-hoc comparisons are Bonferroni-Holm-corrected two-tailed paired t-tests.

B. Track change in activity variation for identical (green) and disparate (magenta) landmark pairs between tracks 1 and 2 at individual (left) and combined (right) distances. Statistical tests are two-way ANOVA (left) and two-tailed paired t-test (right).

C. Similar to B, but for activity variation difference for tracks 1 and 2. Statistical tests are the same as those in B.

D-F. Similar to A-C for rank correlations in tracks 1 and 2. Statistical tests are the same as those in A-C. Each cue cell group contains 10 cells.

G-L. Similar to A-F for tracks 1 and 3.

M-R. Similar to A-F for tracks 2 and 3.

Activity variation data were from 5 mice, with 114, 120, and 127 cells for track comparisons 1 and 2, 1 and 3, as well as 2 and 3, respectively. Rank correlation data were from 4 mice (88 cell groups), 3 mice (82 cell groups), and 3 mice (90 cell groups), respectively, for track comparisons 1 and 2, 1 and 3, as well as 2 and 3. Violin plots have dot and horizontal bar representing median and mean, respectively. Other data are presented as mean ± SEM. Statistical information is available in Supplementary Table 1.

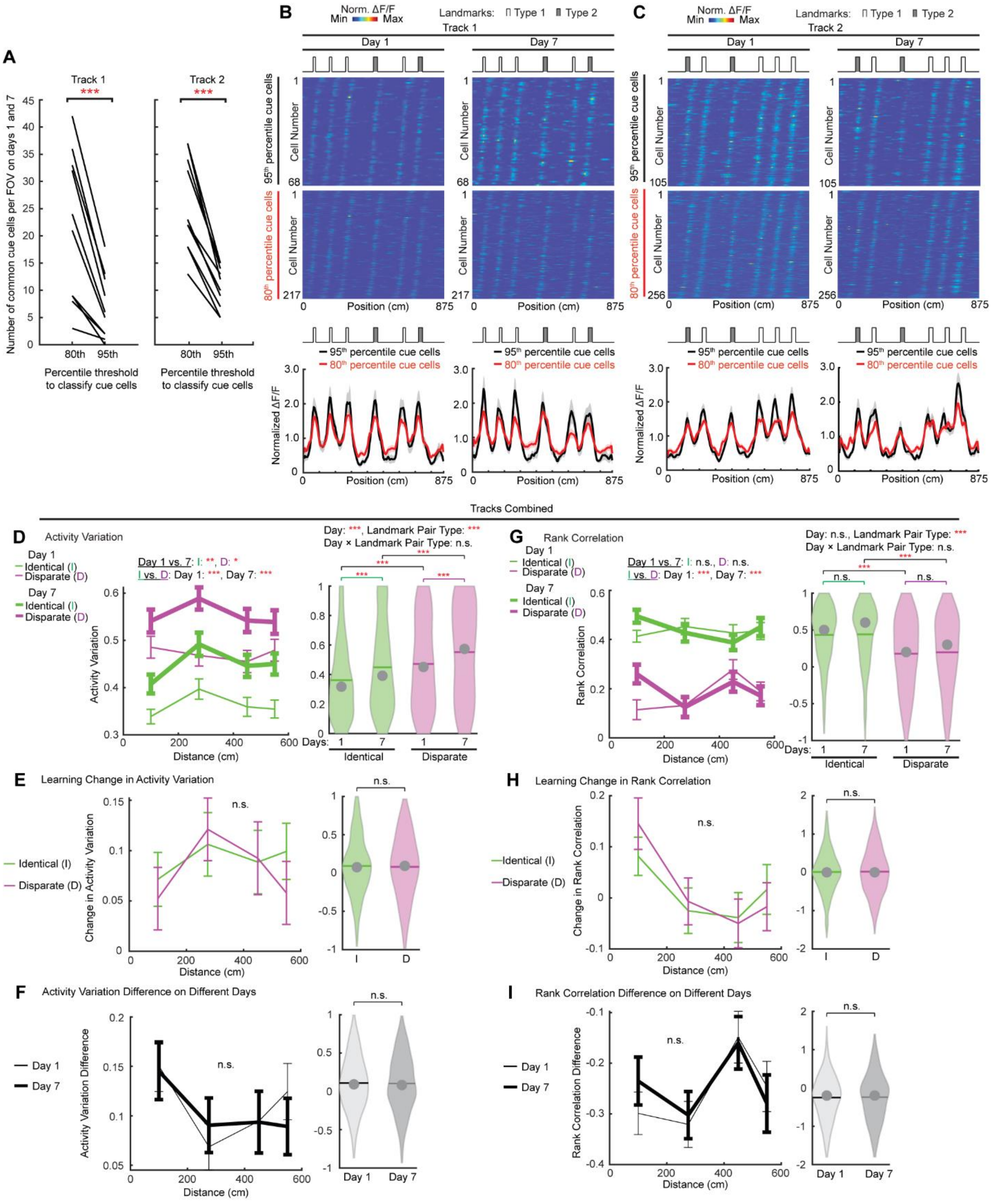

Fig. S17. The features of cue cells classified using the 80th and 95th percentile thresholds of the shuffled distribution.

A. Number of common cue cells across days 1 and 7 of the same tracks under different thresholds. Asterisks indicate the results of two-tailed paired t-tests.

B and C. The activity of common cue cells on days 1 and 7 of tracks 1 (B) and 2 (C), classified using the two thresholds. Top: heat plots, cue cell activity patterns sorted by their spatial shifts in each track. Hotter colors indicate higher normalized activity. Bottom: calcium activity of cue cell populations under the two thresholds. Note that both thresholds produce cue cells with strongly landmark-associated activity.

D. Left: activity variation of common cue cells on day 1 (dashed lines) vs day 7 (dashed solid lines) for identical (I, green) and disparate (D, magenta) landmark pairs across distances, with data pooled from tracks 1 and 2. Statistics are two-way ANOVA comparisons with Bonferroni-Holm correction. Right: activity variation for combined distances pooled from both tracks. Above statistics are two-way ANOVA comparisons. Post-hoc comparisons are two-tailed paired t-tests with Bonferroni-Holm correction.

E. Learning change in activity variation for identical (I, green) and disparate (D, magenta) landmark pairs on days 1 and 7 for both tracks together at individual (left) as well as combined (right) distances. Statistics are two-way ANOVA (left) and two-tailed paired t-test (right).

F. Similar to **E**, but for activity variation difference on days 1 and 7.

G-I. Similar to **D-F** for rank correlations on day 1 and day 7. Statistical tests are the same as those in **D-F**. Each cue cell group contains 5 cells.

80th percentile cue threshold data were from 5 mice, including 217 and 256 cells in tracks 1 and 2, respectively. 95th percentile cue threshold data were from 5 mice, including 68 and 105 cells in tracks 1 and 2, respectively. Track 1 95th percentile rank correlation data were from 4 mice. Track 2 95th percentile rank correlation data were from 5 mice, together producing 173 cue cell groups. Violin plots have dot and horizontal bar representing median and mean, respectively. Other data are presented as mean ± SEM. Statistical information is available in Supplementary Table 1.

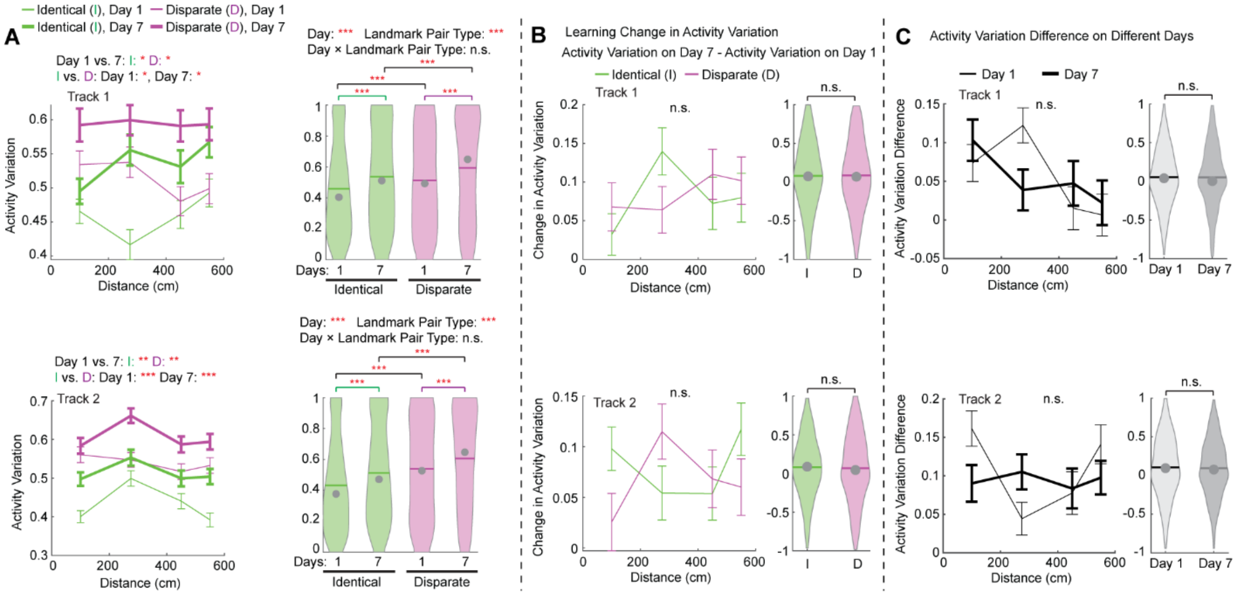

Figure S18. Cue cells improve their discrimination of landmarks with experience, but not their encoding of landmark identity, as determined by activity variation calculations in each track.

A. Left: Activity variation of common cue cells in track 1 (top) and track 2 (bottom) for identical (I, green) and disparate (D, magenta) landmark pairs across distances on day 1 (thin lines) and day 7 (thick lines). Statistics are two-way ANOVA comparisons with Bonferroni-Holm correction conducted separately for each track. Right: activity variation calculations for combined distances. Above statistics are two-way ANOVA comparisons showing that day and landmark pair type modulate activity variation separately, but not jointly. Post-hoc comparisons are two-tailed paired t-tests with Bonferroni-Holm correction conducted separately for each track.

B. Track changes in activity variation for identical (green) and disparate (magenta) landmark pairs in track 1 (top) and track 2 (bottom) at individual (left) and combined (right) distances. Statistical tests are two-way ANOVA comparisons (left) and two-tailed paired t-tests (right).

C. Similar to B, but for activity variation difference on day 1 and day 7 for track 1 (top) and track 2 (bottom). Statistical tests are the same as those in B.

Track 1 data included 217 cells common to day 1 and day 7. Track 2 data included 256 cells common to day 1 and day 7. Violin plots have dot and horizontal bar representing median and mean, respectively. Other data are presented as mean ± SEM. Statistical information is available in Supplementary Table 1.

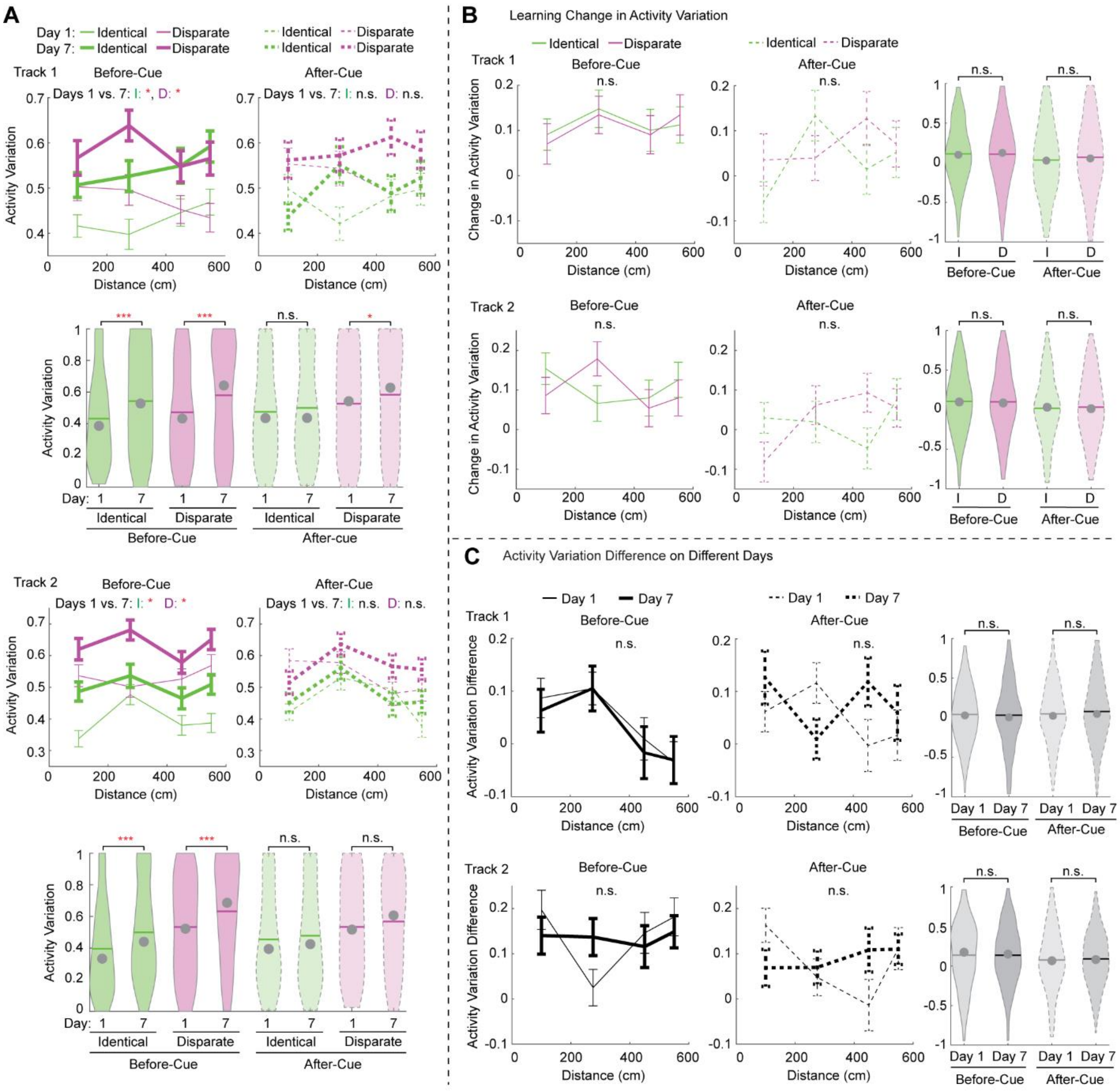

Figure S19. Cue cells show stronger improvements in prospective compared to retrospective discrimination of landmarks with experience, as demonstrated by activity variation calculations.

A. Activity variation for before-cue (solid lines) and after-cue (dashed lines) cells in tracks 1 and 2 at individual and combined distances on day 1 and day 7 for identical (I, green) and disparate (D, magenta) landmark pairs. Line plots include two-way ANOVA comparisons between day 1 and 7 curves for identical and disparate landmarks. Bonferroni-Holm correction conducted across both cell types for each track separately. Violin plots include two-tailed paired t-test comparisons between day 1 and day 7 activity variation for all conditions. Bonferroni-Holm correction conducted separately for each track.

B. Learning change in activity variation for before-cue (solid lines) and after-cue (dashed lines) cells at individual and combined distances for identical and disparate landmark pairs in tracks

1 and 2. Statistical tests for line plots are two-way ANOVA comparisons between identical and disparate curves. Bonferroni-Holm p-value correction conducted across both cell types for each track separately. Statistical tests for violin plots are two-tailed paired t-tests which are Bonferroni-Holm corrected separately for each track.

C. Activity variation difference for before-cue (solid lines) and after-cue (dashed lines) cells at individual and combined distances on day 1 and day 7 in tracks 1 and 2. Statistical tests for line plots are two-way ANOVA comparisons between day 1 and day 7 curves. Bonferroni-Holm p-value correction conducted across both cell types for each track separately. Statistical tests for violin plots are two-tailed paired t-tests which are Bonferroni-Holm corrected separately for each track.

Track 1 data were from 5 mice and 168 cells. Track 2 data were from 5 mice and 160 cells. Violin plots have dot and horizontal bar representing median and mean, respectively. Other data are presented as mean ± SEM. Statistical information is available in Supplementary Table 1.

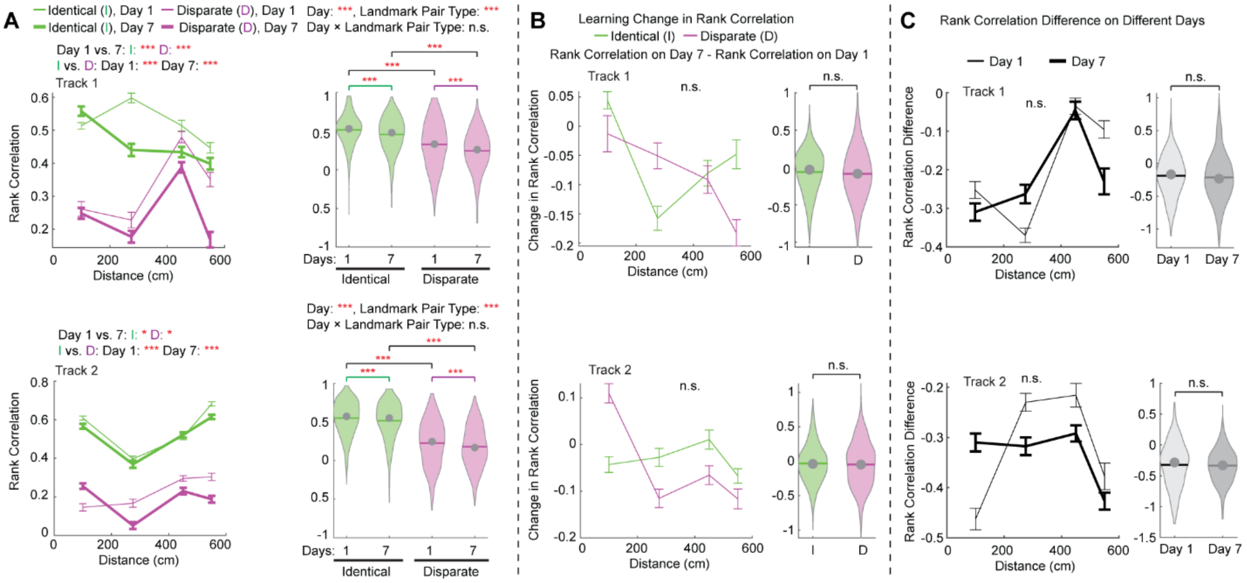

Figure S20. Populations of cue cells improve their discrimination of landmarks with experience, but not their encoding of landmark identity, as determined by rank correlation calculations in each track.

A. Left: rank correlation of common cue cells in track 1 (top) and track 2 (bottom) for identical (I, green) and disparate (D, magenta) landmark pairs across distances on day 1 (thin lines) and day 7 (thick lines). Statistics are two-way ANOVA comparisons with Bonferroni-Holm correction conducted separately for each track. Right: rank correlation calculations for combined distances. Above statistics are two-way ANOVA comparisons showing that day and landmark pair type modulate rank correlation separately, but not jointly. Post-hoc comparisons are two-tailed paired t-tests with Bonferroni-Holm correction conducted separately for each track. Each cue cell group contains 15 cells.

B. Track changes in rank correlation for identical (green) and disparate (magenta) landmark pairs in track 1 (top) and track 2 (bottom) at individual (left) and combined (right) distances. Statistical tests are two-way ANOVA comparisons (left) and two-tailed paired t-tests (right).

C. Similar to B, but for rank correlation difference on day 1 and day 7 for track 1 (top) and track 2 (bottom). Statistical tests are the same as those in B.

Track 1 rank correlation data were from 3 mice and 188 cell groups common to day 1 and day 7. Track 2 rank correlation data were from 5 mice and 243 cell groups common to day 1 and day 7. Violin plots have dot and horizontal bar representing median and mean, respectively. Other data are presented as mean ± SEM. Statistical information is available in Supplementary Table 1.

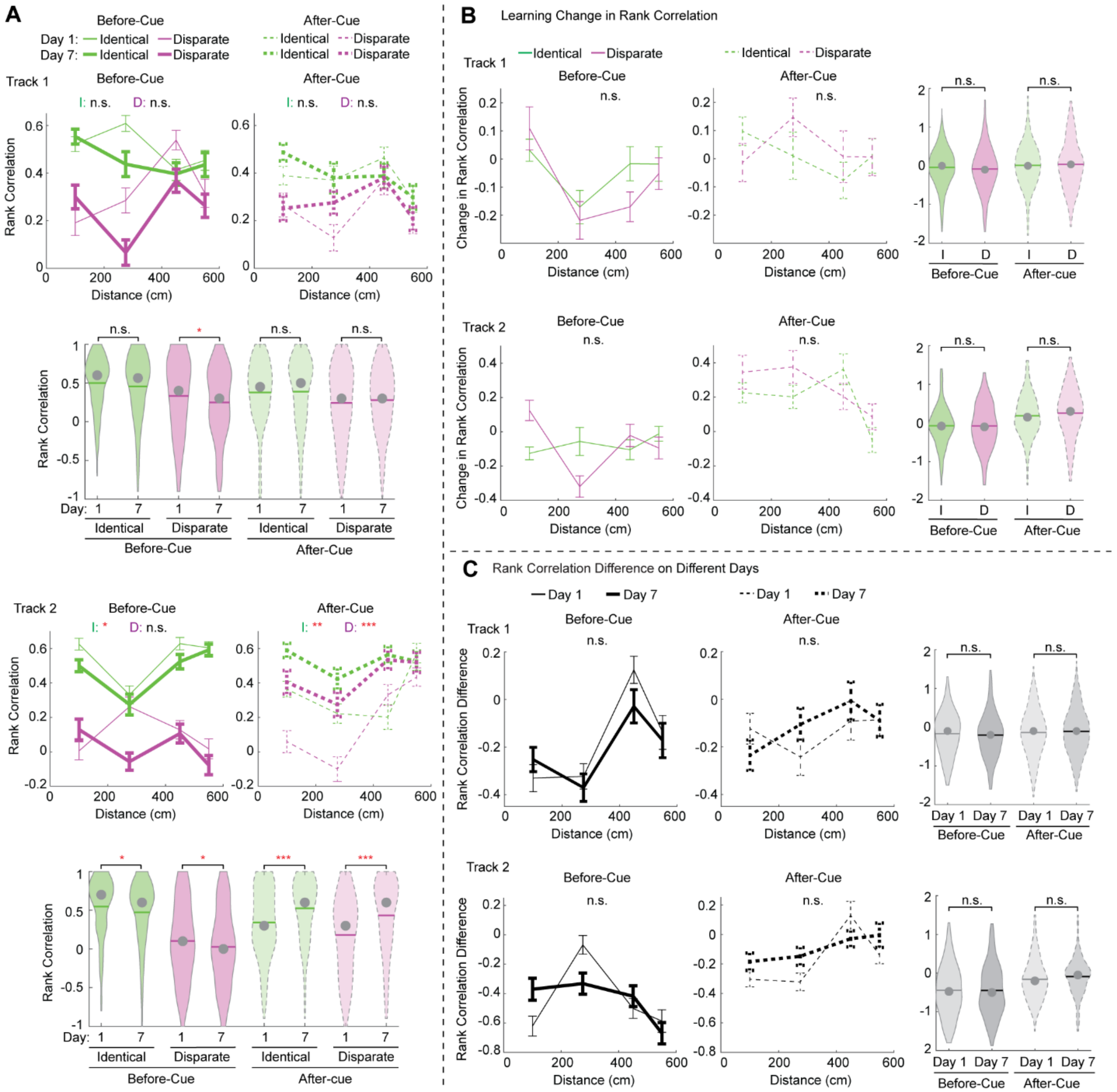

Figure S21. Population of cue cells show stronger improvements in prospective compared to retrospective discrimination of landmarks with experience, as demonstrated by rank correlation calculations.

A. Rank correlation for before-cue (solid lines) and after-cue (dashed lines) cells in tracks 1 and 2 at individual and combined distances on day 1 and day 7 for identical (I, green) and disparate (D, magenta) landmark pairs. Line plots include two-way ANOVA comparisons between day 1 and 7 curves for identical and disparate landmarks. Bonferroni-Holm correction conducted across both cell types for each track separately. Violin plots include two-tailed paired t-test comparisons between day 1 and day 7 rank correlation for all conditions. Bonferroni-Holm correction conducted separately for each track. Each cue cell group contains 5 cells.

B. Learning change in rank correlation for before-cue (solid lines) and after-cue (dashed lines) cells at individual and combined distances for identical and disparate landmark pairs in tracks

1 and 2. Statistical tests for line plots are two-way ANOVA comparisons between identical and disparate curves. Bonferroni-Holm p-value correction conducted across both cell types for each track separately. Statistical tests for violin plots are two-tailed paired t-tests which are Bonferroni-Holm corrected separately for each track.

C. Rank correlation difference for before-cue (solid lines) and after-cue (dashed lines) cells at individual and combined distances on day 1 and day 7 in tracks 1 and 2. Statistical tests for line plots are two-way ANOVA comparisons between day 1 and day 7 curves. Bonferroni-Holm p-value correction conducted across both cell types for each track separately. Statistical tests for violin plots are two-tailed paired t-tests which are Bonferroni-Holm corrected separately for each track.

Track 1 data were from 3 mice and 152 cell groups. Track 2 data were from 5 mice and 135 cell groups. Violin plots have dot and horizontal bar representing median and mean, respectively. Other data are presented as mean ± SEM. Statistical information is available in Supplementary Table 1.

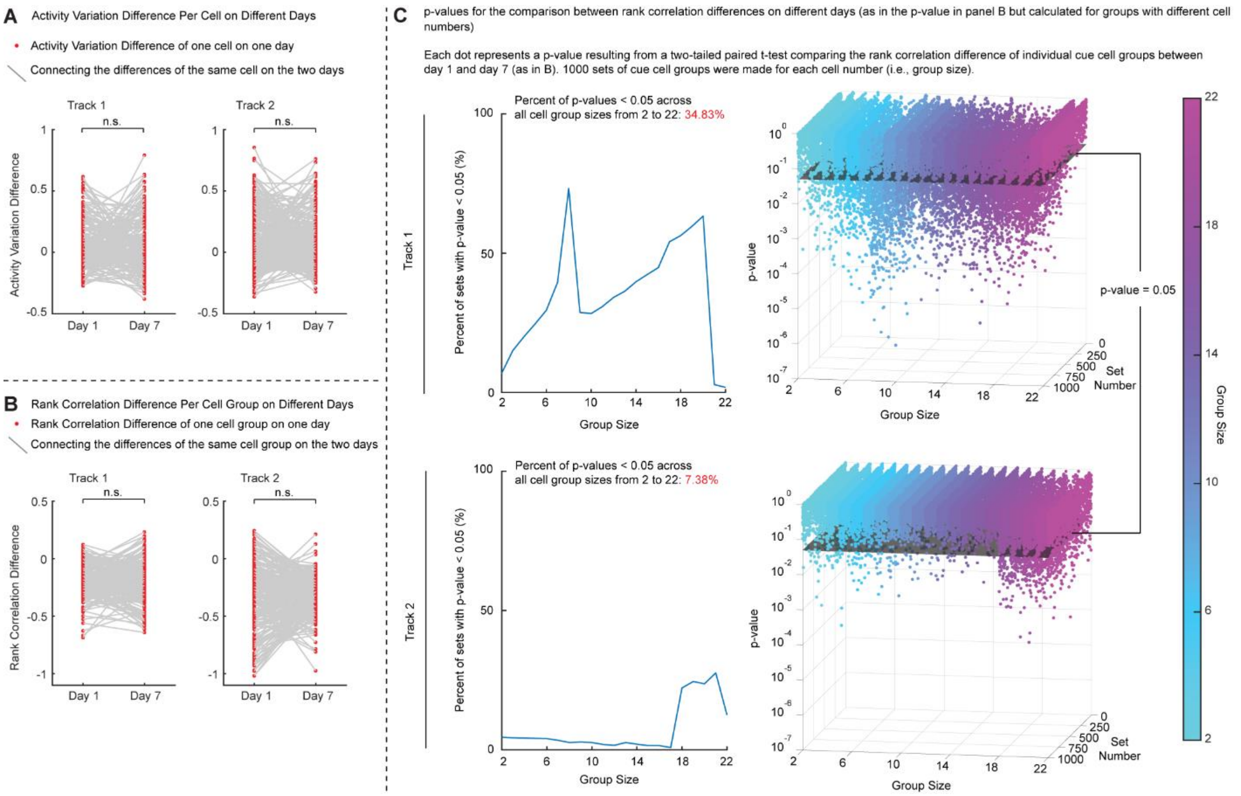

Figure S22. Individual and groups of cue cells show no change in their encoding of landmark identity with experience.

A. Activity variation difference of individual cells on day 1 and day 7 for track 1 and 2. Statistical tests are two-tailed paired t-tests.

B. Rank correlation difference of individual cell groups on day 1 and day 7 for tracks 1 and 2. Statistical tests are two-tailed paired t-tests. Each cue cell group contains 15 cells, just as in the analysis from Fig. 6D-F and Fig. S17.

C. p-values for the comparisons between rank correlation differences of the same groups of common cells on different days when varying the size of cell groups. Each dot is the p-value for one calculation using one set of common cell groups, as in B. Since the cell groups were randomly sampled within each FOV, 1000 calculations were made using 1000 sets of common cell groups for each group size. The individual p-values were color coded according to the number of cue cells in the group and shown on a logarithmic scale in comparison to a significance level of 0.05. Despite some cue cell group sizes having large percentages of calculations with significant p-values, in both tracks, a majority of calculations across all choices for the group size do not show significant p-values.

Activity variation data in panel A were from 5 mice, with 217 and 256 cells common to day 1 and day 7 for track 1 and track 2, respectively. Track 1 rank correlation data in panel B were from 3 mice and 188 cell groups common to day 1 and day 7. Track 2 rank correlation data in panel B were from 5 mice and 243 cell groups common to day 1 and day 7. In panel C, the number of FOVs from all 5 mice that were included in the analysis varied based on the number of cells per

group (see Data Analysis - Rank correlation for a pair of landmarks in Methods). Statistical information is available in Supplementary Table 1.

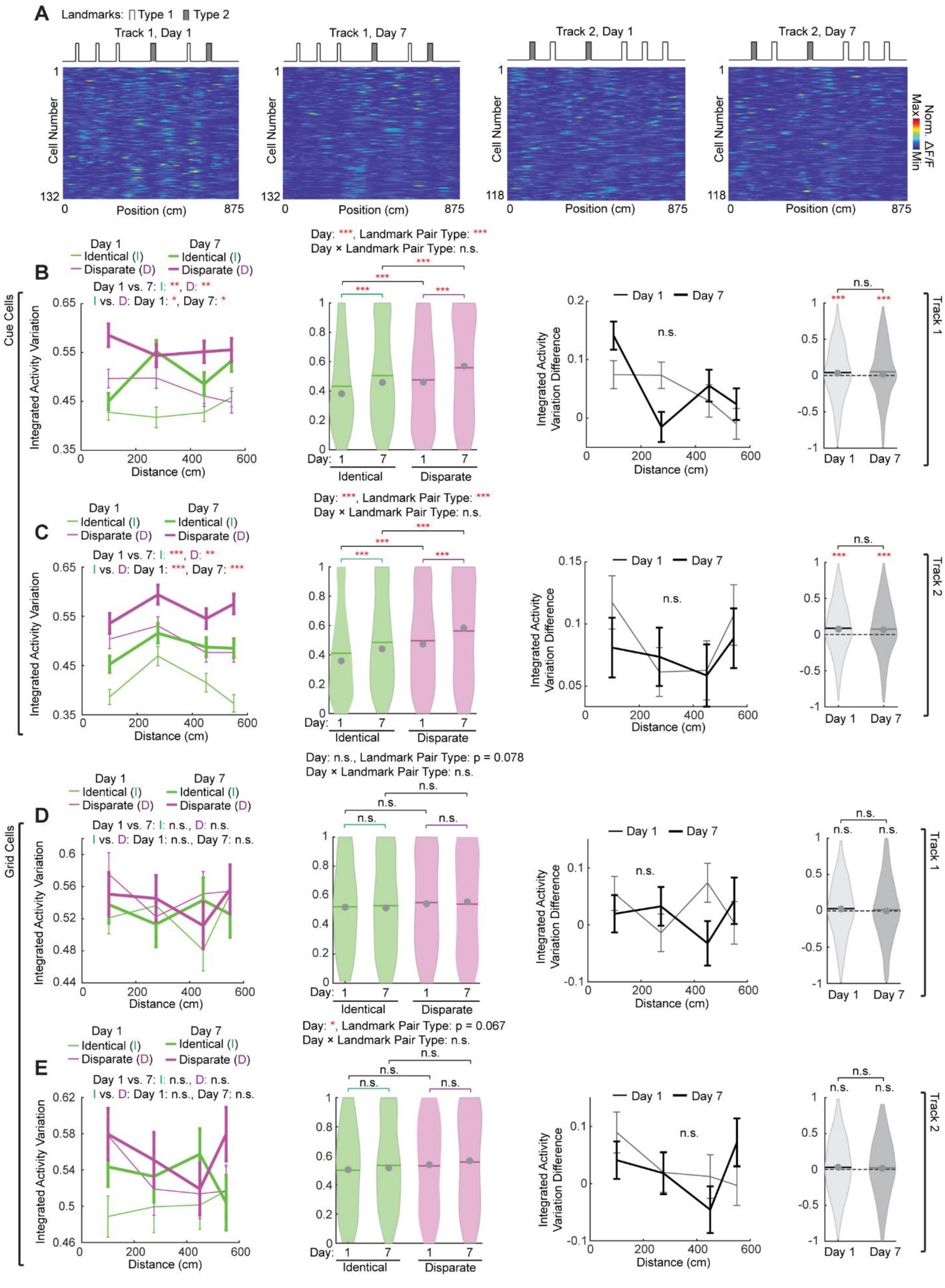
A
Landmarks: Type 1 Type 2
Track 1, Day 1
Track 1, Day 7
Track 2, Day 1
Track 2, Day 7
Cell Number
Position (cm)
Norm. ΔF/F
Max
Min
B
C
D
E
Cue Cells
Grid Cells
Day 1
Day 7
Identical (I)
Disparate (D)
Day 1 vs. 7: I: **, D: **
I vs. D: Day 1: *, Day 7: *
Day 1 vs. 7: I: ***, D: **
I vs. D: Day 1: ***, Day 7: ***
Day 1 vs. 7: I: n.s., D: n.s.
I vs. D: Day 1: n.s., Day 7: n.s.
Integrated Activity Variation
Distance (cm)
Day: ***, Landmark Pair Type: ***
Day × Landmark Pair Type: n.s.
Day: n.s., Landmark Pair Type: p = 0.078
Day: *, Landmark Pair Type: p = 0.067
Identical
Disparate
Integrated Activity Variation Difference
n.s.
Track 1
Track 2

Figure S23. Comparing MEC cells by functional class shows that grid cells demonstrate a consistent trend of worsening landmark identity encoding with experience for tracks 1 and 2, while cue cells do not.

A. Activity of common putative grid cells on day 1 (left) and day 7 (right) for track 1 (top) and track 2 (bottom). Hotter colors indicate higher normalized activity.

B. Left two panels: Integrated activity variation for identical (I, green) and disparate (D, magenta) landmark pairs on day 1 (thin lines) and day 7 (thick lines) at individual (left) and combined (right) distances for track 1 cue cells. Line plot shows Bonferroni-Holm corrected two-way ANOVA comparisons. Violin plot includes Bonferroni-Holm-corrected two-way ANOVA comparison showing that day and landmark pair type modulate activity variation separately, but not jointly. Post-hoc tests are two-tailed paired t-tests with Bonferroni-Holm correction. Right two panels: Integrated Activity Variation Difference on day 1 and day 7 for track 1 cue cells. Line plot statistical test is two-way ANOVA between day 1 and day 7 curves. Violin plot statistical test is two-tailed paired t-test.

C. Similar to B for track 2 cue cells. Statistical tests are the same as in B.

D-E. Similar to B and C for putative grid cells in track 1 and track 2. Statistical tests and error correction procedures are the same as in B and C. Putative grid cells show nonsignificant trends of decreasing integrated activity variation difference between days 1 and 7.

Cue cell data were from 5 mice, with 217 and 256 cells in tracks 1 and 2, respectively. Putative grid cell data were from 5 mice, with 132 and 118 cells in tracks 1 and 2, respectively. Violin plots have dot and horizontal bar representing median and mean, respectively. Other data are presented as mean ± SEM. Statistical information is available in Supplementary Table 1.

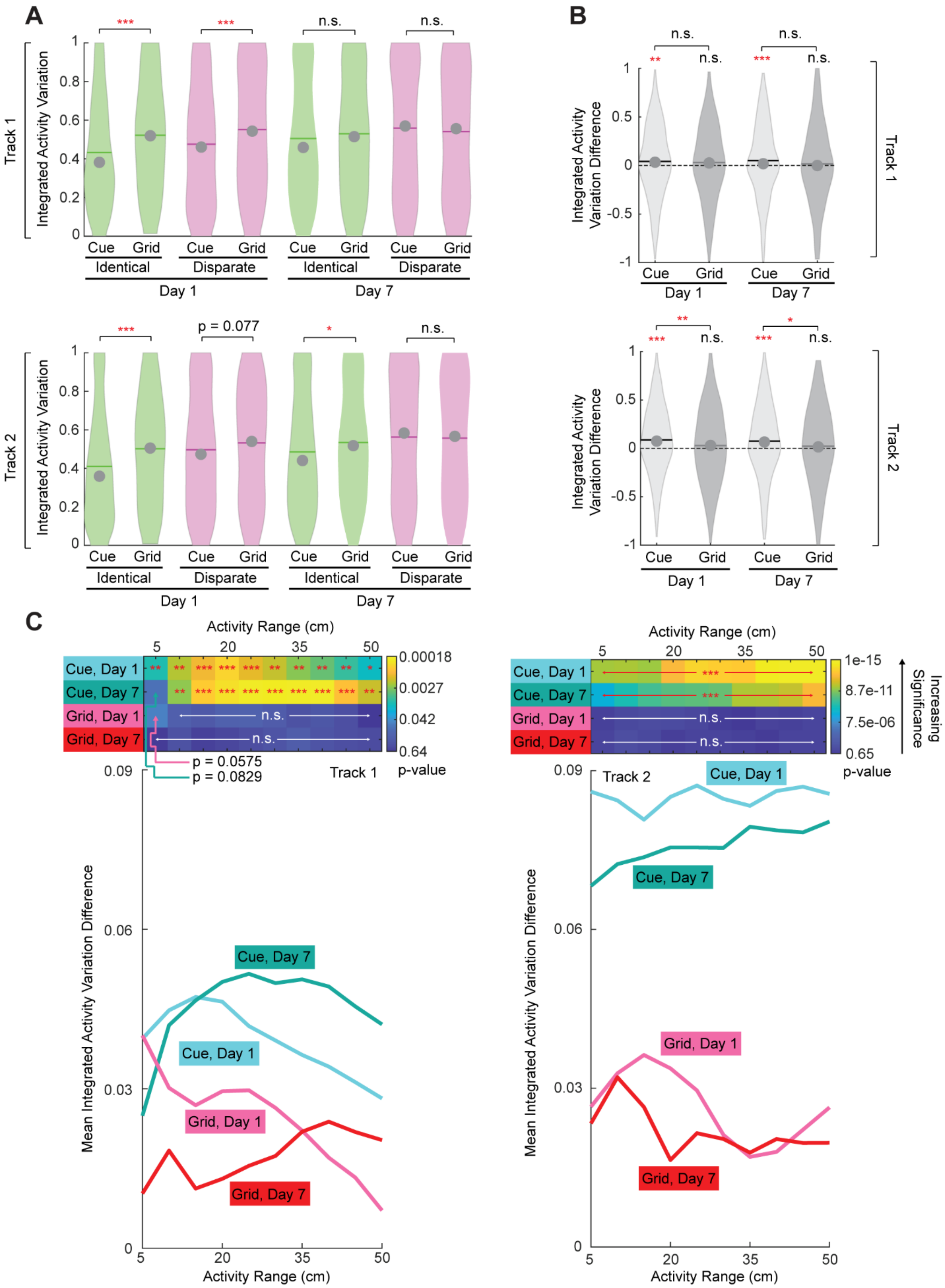
A
***
***
n.s.
n.s.
Track 1
Integrated Activity Variation
Cue
Grid
Identical
Disparate
Day 1
Day 7
***
p = 0.077
*
n.s.
Track 2
B
n.s.
**
n.s.
***
Integrated Activity Variation Difference
C
Activity Range (cm)
Cue, Day 1
Cue, Day 7
Grid, Day 1
Grid, Day 7
0.00018
0.0027
0.042
0.64
p-value
p = 0.0575
p = 0.0829
1e-15
8.7e-11
7.5e-06
0.65
Increasing Significance
Mean Integrated Activity Variation Difference

Figure S24. Putative grid cells in tracks 1 and 2 show trends of decreasing landmark identity encoding with experience, contrasting with cue cells, and exhibit trends of decreasing mean integrated activity variation difference as functions of the studied activity range on day 1.

A. Integrated activity variations for identical (green) and disparate (magenta) landmark pairs in cue and putative grid cells across days 1 and 7 and tracks 1 and 2 for all distances. Statistical tests are two-tailed nonpaired t-tests Bonferroni-Holm-corrected separately for each track.

B. Integrated activity variation differences on days 1 and 7 for cue and putative grid cells in tracks 1 and 2. Statistical tests include two-tailed paired t-tests for each sample's comparison with zero, which are Bonferroni-Holm corrected separately for each track. The results of two-tailed nonpaired t-tests are shown comparing the integrated activity variation difference of cue and putative grid cells on the same day, with Bonferroni-Holm correction conducted separately for each track.

C. Mean integrated activity variation differences for cue and putative grid cells on days 1 and 7 as functions of activity range. p-value heatmap shows hotter colors for more significant two-tailed paired t-tests relative to zero, obtained as in Fig. 7C and E, with Bonferroni-Holm correction conducted together for each functional class's day 1 and 7 p-values. Putative grid cells on day 1 show trends of decreasing mean integrated activity variation difference with increasing activity range.

Data were from 5 mice with 217 cue cells and 132 putative grid cells in track 1. Track 2 data were from 5 mice with 256 cue cells and 118 putative grid cells. Violin plots have dot and horizontal bar representing median and mean, respectively. Statistical information is available in Supplementary Table 1.

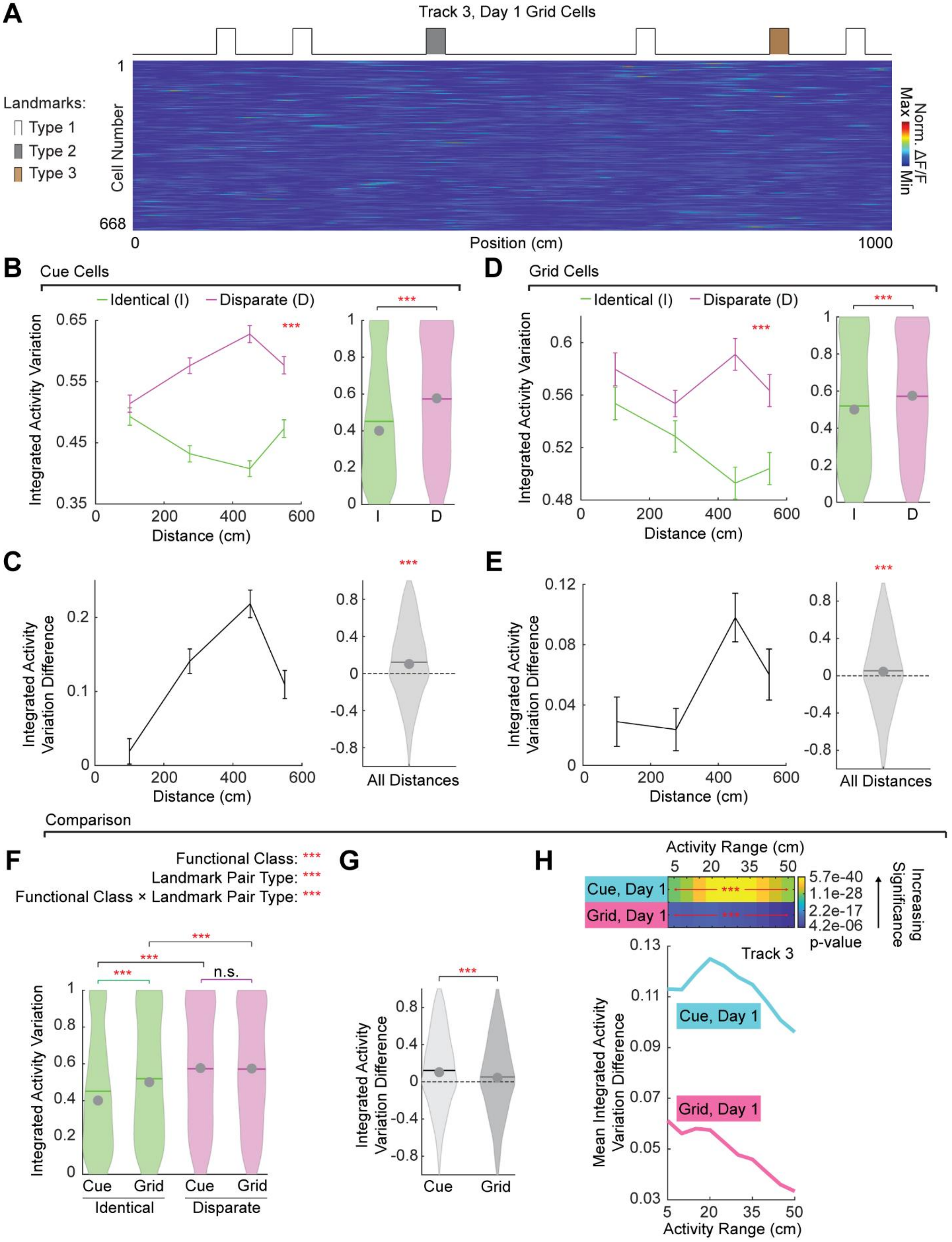
A
Track 3, Day 1 Grid Cells
Landmarks:
Type 1
Type 2
Type 3
Cell Number
1
668
Norm. ΔF/F
Max
Min
0
Position (cm)
1000
B
Cue Cells
Identical (I)
Disparate (D)
Integrated Activity Variation
Distance (cm)
I
D
***
C
Integrated Activity Variation Difference
All Distances
D
Grid Cells
E
Comparison
F
Functional Class: ***
Landmark Pair Type: ***
Functional Class × Landmark Pair Type: ***
n.s.
Cue
Grid
Identical
Disparate
G
H
Activity Range (cm)
Cue, Day 1
Grid, Day 1
5.7e-40
1.1e-28
2.2e-17
4.2e-06
p-value
Increasing Significance
Track 3
Mean Integrated Activity Variation Difference

Figure S25. Comparing MEC cells by functional class on day 1 of track 3 shows landmark identity encoding in both functional cell types, but with weaker identity encoding in putative grid cells, as well as sensitivity to the size of the studied activity range.

A. Activity of day 1 putative grid cells in track 3. Hotter colors indicate higher normalized activity.

B. Integrated activity variation for identical (I, green) and disparate (D, magenta) landmark pairs in track 3 cue cells at individual (left) and combined (right) distances. Left panel statistical test is a two-way ANOVA comparing I and D curves. Right panel statistical test is a two-tailed paired t-test.

C. Integrated activity variation difference for track 3 cue cells at individual (left) and combined (right) distances. Right panel statistical test is a two-tailed paired t-test comparison with zero.

D-E. Similar to A and B for track 3, for day 1 putative grid cells. Statistical tests are the same as those in A and B.

F. Integrated activity variation for cue and putative grid cells at identical and disparate landmark pairs. Two-way ANOVA results (above) show that functional class and landmark pair type modulate integrated activity variation both separately and jointly. Post-hoc tests are two-tailed paired t-tests with Bonferroni-Holm p-value correction.

G. Integrated activity variation difference for cue and putative grid cells in track 3. Cue cells show stronger encoding of landmark identity. Asterisks indicate the result of a two-tailed nonpaired t-test.

H. Mean integrated activity variation difference for cue and putative grid cells on day 1 in track 3 as functions of activity range. Heatmap shows hotter colors for more significant p-values obtained from paired t-tests of integrated activity variation difference for various activity ranges from 5 to 50 cm, similar to those in C and E.

Data were from 5 mice, with 508 cue cells and 668 putative grid cells. Violin plots have dot and horizontal bar representing median and mean, respectively. Other data are presented as mean ± SEM. Statistical information is available in Supplementary Table 1.

Supplementary Table 1: Statistical Information

**Figure 2**	
2D (left)	Two-way ANOVA: p = 3.53e-31, F = 138.46, I: 1407 cells x 4 matched distances; D: 1407 cells x 4 matched distances
2D (right)	Two-tailed paired t-test: p = 3.8069e-79, n = 5628 activity variations across all cells and matched distances for I and D
2E	Two-tailed paired t-test with 0: p = 3.8069e-79, n = 5628 activity variation differences across all cells and matched distances
2F (left)	Two-tailed Pearson's linear correlation: r = 0.4986, p = 5.5011e-89, n = 1407 cells
2F (right)	Two-tailed paired t-test: p = 2.987e-07, n = 30 FOVs

**Figure 3**	
3B (left)	Two-way ANOVA: p = 4.605e-144, F = 763.65, I: 1407 cell groups x 4 matched distances; D: 1407 cell groups x 4 matched distances
3B (right)	Two-tailed paired t-test: reported as p = 0 by MATLAB ttest function, n = 5628 rank correlations across all cell groups and matched distances for I and D
3C	Two-tailed paired t-test: reported as p = 0 by MATLAB ttest function, n = 5628 rank correlation differences across all cell groups and matched distances
3D (left)	Two-tailed Pearson's linear correlation: r = 0.30666, p = 5.0939e-32, n = 1407 cell groups
3D (right)	Two-tailed paired t-test: p = 1.7827e-09, n = 30 FOVs
3E	Rank correlations listed at each landmark are the two-tailed Pearson's linear correlations of each landmark's ranking of the same 38 cells compared to the reference landmark.

**Figure 4**	
4C	One-way ANOVA considering effect of Spatial Shift Cell Type (Before / At / After): p = 1.1508e-21, F = 123.91, n = 30 FOVs (3 tracks, 10 FOVs each) for each Cell Type<br><br>Bonferroni-Holm-corrected post-hoc two-tailed paired t-tests:<br>$p_{Before\text{-}At}$ = 1.7112e-17<br>$p_{At\text{-}After}$ = 1.8471e-13<br>$p_{Before\text{-}After}$ = 0.00065547
4D (panels 1-3)	Before cue (panel 1): I: 734 cells x 4 matched distances; D: 734 cells x 4 matched distances, Bonferroni-Holm-corrected Two-way ANOVA: p = 1.2888e-23, F = 106.39

	At cue (panel 2): I: 102 cells x 4 matched distances; D: 102 cells x 4 matched distances, Bonferroni-Holm-corrected Two-way ANOVA: $p = 0.00021977$, $F = 14.201$<br><br>After cue (panel 3): I: 571 cells x 4 matched distances; D: 571 cells x 4 matched distances, Bonferroni-Holm-corrected Two-way ANOVA: $p = 5.4761e-07$, $F = 26.791$
4D (panel 4)	Bonferroni-Holm-corrected two-tailed paired t-tests comparing identical to disparate activity variation:<br>$p_{Before} = 3.8783e-56$, $n = 2936$ for both I and D<br>$p_{At} = 3.3212e-10$, $n = 408$ for both I and D<br>$p_{After} = 1.496e-18$, $n = 2284$ for both I and D<br><br>Unit for all n is activity variations across all specified cells and all four matched distances.
4E	Unit for all n is activity variation differences across all specified cells and all four matched distances.<br><br>Two-way ANOVA considering effect of Track ($p = 0.0178$, $F = 4.0420$), Cell Type ($p = 0.0072$, $F = 4.9488$) and the join contribution of Track and Cell Type ($p = 3.0354e-05$, $F = 6.5791$) on activity variation difference.<br><br>Track 1: Grouped By Bonferroni-Holm correction family of tests<br><br>$n_{Before} = 228$, $n_{At} = 41$, $n_{After} = 165$<br><br>Two-tailed non-paired t-tests:<br>$P_{Before-At} = 0.11989$<br>$P_{At-After} = 0.22285$<br>$P_{Before-After} = 0.40023$<br><br>Two-tailed paired t-tests:<br>$P_{Before-0} = 0.0012928$<br>$P_{At-0} = 0.00093041$<br>$P_{After-0} = 3.7949e-05$<br><br>Track 2: Grouped By Bonferroni-Holm correction family of tests<br><br>$n_{Before} = 238$, $n_{At} = 29$, $n_{After} = 198$<br><br>Two-tailed non-paired t-tests:<br>$P_{Before-At} = 0.071278$<br>$P_{At-After} = 0.63894$

	$P_{Before\text{-}After}$ = 2.1555e-07<br><br>Two-tailed paired t-tests:<br>$P_{Before\text{-}0}$ = 1.687e-21<br>$P_{At\text{-}0}$ = 0.096283<br>$P_{After\text{-}0}$ = 0.0013774<br><br>Track 3: Grouped By Bonferroni-Holm correction family of tests<br>n_{Before} = 268, n_{At} = 32, n_{After} = 208<br><br>Two-tailed non-paired t-tests:<br>$P_{Before\text{-}At}$ = 0.53416<br>$P_{At\text{-}After}$ = 0.70437<br>$P_{Before\text{-}After}$ = 0.67829<br><br>Two-tailed paired t-tests:<br>$P_{Before\text{-}0}$ = 2.3521e-14<br>$P_{At\text{-}0}$ = 6.6792e-05<br>$P_{After\text{-}0}$ = 1.9429e-10
4F	All correlations below are two-tailed Pearson's linear correlations of spatial shifts which have at least 5 cells along with their associated averaged activity variation difference. The analysis was conducted separately for positive-shift and negative-shift cells.<br><br>Track 1:<br>Positive-shift cells' correlation: r = -0.913586, p = 0.000219557<br>Negative-shift cells' correlation: r = -0.0531606, p = 0.891957<br><br>Track 2:<br>Positive-shift cells' correlation: r = 0.715781, p = 0.0199183<br>Negative-shift cells' correlation: r = 0.631553, p = 0.0371404<br><br>Track 3:<br>Positive-shift cells' correlation: r = -0.792388, p = 0.00627307<br>Negative-shift cells' correlation: r = 0.363952, p = 0.301196<br><br>Two-tailed paired t-test comparisons with zero: The following p-values are Bonferroni-Holm corrected for all lags within each track; the number of cells n is also listed. N/A noted for spatial shifts without enough cells (≥ 5 required) for a two-tailed paired t-test calculation.

Spatial shifts (cm)	Track 1	Track 2	Track 3

	45	p = 1, n = 6	p = 0.0315, n = 19	p = 1, n = 12	
	40	p = 1, n = 8	p = 0.2119, n =10	p = 1, n = 12	
	35	p =1, n = 12	p = 0.0003, n = 19	p = 1, n = 12	
	30	p =1, n = 26	p = 0.3512, n = 18	p = 0.6649, n = 24	
	25	p =1, n = 17	p = 0.0026, n = 32	p = 0.0629, n = 40	
	20	p =1, n = 35	p = 0.2091, n = 32	p = 0.4362, n = 39	
	15	p =1, n = 37	p = 0.2088, n = 27	p = 0.0014, n = 50	
	10	p = 0.4966, n = 48	p = 0.0006, n = 42	p = 0.0074, n = 38	
	5	p = 0.1909, n = 37	p = 0.0373, n = 34	p = 0.0011, n = 34	
	0	p = 0.0195, n = 41	p = 0.7703, n = 29	p = 0.0014, n = 32	
	-5	p = 0.1943, n = 21	p = 0.7711, n = 37	p = 0.0340, n = 32	
	-10	p = 0.0992, n = 32	p = 0.0704, n = 37	p = 0.0016, n = 27	
	-15	p = 0.1840, n = 29	p = 0.1609, n = 36	p = 0.0245, n = 44	
	-20	p = 0.9206, n = 31	p = 1, n = 26	p = 1, n = 28	
	-25	p = 1, n = 24	p = 1, n = 20	p = 1, n = 26	
	-30	p = 1, n = 9	p = 0.9957, n = 9	p = 0.1712, n = 13	
	-35	p = 1, n = 6	p = 0.5331, n = 9	p = 1, n = 17	
	-40	N/A	p = 0.7230, n = 7	p = 0.7911, n = 8	
	-45	N/A	p = 1, n = 10	N/A	
	-50	p = 0.7158, n = 7	p = 1, n = 7	p = 1, n = 5	

Figure 5	
5A (left)	Two-way ANOVA: Bonferroni-Holm Corrected<br>All curves: 359 cells x 4 distances

	I Track 1 vs I track 2: p = 0.010927, F = 8.5116<br>D Track 1 vs D track 2: p = 0.54296, F = 0.37045<br>Track 1 I vs D: p = 0.052488, F = 4.9611<br>Track 2 I vs D: p = 7.2428e-08, F = 32.436
5A (right)	Two-way ANOVA:<br>Track: p = 0.039879, F = 4.231<br>Landmark Pair Type: p = 1.1847e-18, F = 79.961<br>Track x Landmark Pair Type: p = 0.00069307, F = 11.56<br><br>Two-tailed paired t-tests: Bonferroni-Holm Corrected<br><br>Each violin contains n = 1436 activity variations across all cells and all four matched distances.<br><br>I Track 1 vs I track 2: p = 0.00032698<br>D Track 1 vs D track 2: p = 0.66718<br>Track 1 I vs D: p = 0.00040966<br>Track 2 I vs D: p = 5.0896e-18
5B (left)	Two-way ANOVA:<br>Both curves: 359 cells x 4 distances<br>p = 0.022869, F = 5.202
5B (right)	Two-tailed paired t-test: Each violin contains n = 1436 changes in activity variation across all cells and all four matched distances.<br>p = 0.00069307
5C (left)	Two-way ANOVA:<br>Both curves: 359 cells x 4 distances<br>Track 1 vs Track 2: p = 0.00049468, F = 12.252
5C (right)	Two-tailed paired t-tests: p = 0.00027278, n = 1436 activity variation differences across all cells and all four matched distances.
5D (left)	Two-way ANOVA: Bonferroni-Holm Corrected<br>All curves: 359 cell groups x 4 distances<br><br>I Track 1 vs I track 2: p = 2.5456e-08, F = 33.161<br>D Track 1 vs D track 2: p = 3.393e-28, F = 134.79<br>Track 1 I vs D: p = 0.00031613, F = 13.1<br>Track 2 I vs D: p = 2.0038e-74, F = 432.09
5D (right)	Two-way ANOVA<br>Track: p = 5.7164e-15, F = 62.353<br>Landmark Pair Type: p = 3.853e-100, F = 531.72<br>Track x Landmark Pair Type: p = 3.5179e-81, F = 415.46<br><br>Two-tailed paired t-tests: Bonferroni-Holm Corrected

	Each violin contains n = 1436 rank correlations across all cell groups and all four matched distances<br><br>I Track 1 vs I track 2: p = 1.0321e-11<br>D Track 1 vs D track 2: p = 9.3624e-61<br>Track 1 I vs D: p = 2.6816e-07<br>Track 2 I vs D: p = 7.2649e-164
5E (left)	Two-way ANOVA:<br>Both curves: 359 cell groups x 4 distances<br>p = 8.6251e-39, F = 192.32
5E (right)	Two-tailed paired t-test: Each violin contains n = 1436 changes in rank correlation across all cell groups and all four matched distances.<br><br>p = 3.5179e-81
5F (left)	Two-way ANOVA:<br>Both curves: 359 cell groups x 4 distances<br>Track 1 vs Track 2: p = 7.1857e-49, F = 253.31
5F (right)	Two-tailed paired t-tests: Each violin contains n = 1436 rank correlation differences across all cell groups and all four matched distances.<br>p = 5.9823e-62

**Figure 6**	
6A (left)	Two-way ANOVA: Bonferroni-Holm Corrected<br><br>All curves: 473 cells x 4 distances<br><br>I Day 1 vs I Day 7: p = 2.6206e-05, F = 19.212<br>D Day 1 vs D Day 7: p = 2.7557e-05, F = 17.762<br>Day 1 I vs D: p = 1.9856e-07, F = 29.66<br>Day 7 I vs D: p = 1.6621e-07, F = 30.622
6A (right)	Two-way ANOVA<br>Day: p = 4.3039e-21, F = 91.128<br>Landmark Pair Type: p = 2.1852e-31, F = 141.22<br>Day x Landmark Pair Type: p = 0.68028, F = 0.16987<br><br>Two-tailed paired t-tests: Bonferroni-Holm Corrected<br><br>Each violin contains n = 1892 activity variations across all cells and all four matched distances.<br><br>I Day 1 vs I Day 7: p = 6.034e-16<br>D Day 1 vs D Day 7: p = 4.832e-13

	Day 1 I vs D: p = 1.6329e-19<br>Day 7 I vs D: p = 3.7694e-16
6B (left)	Two-way ANOVA:<br>Both curves: 473 cells x 4 distances<br>p = 0.788174, F = 0.072236
6B (right)	Two-tailed paired t-test: p = 0.68028, n = 1892 changes in activity variation across all cells and all four matched distances for both violins
6C (left)	Two-way ANOVA:<br>Both curves: 473 cells x 4 distances<br>Day 1 vs Day 7: p = 0.8041, F = 0.061566
6C (right)	Two-tailed paired t-tests: p = 0.68028, n = 1892 activity variation differences across all cells and all four matched distances for both violins
6D (left)	Two-way ANOVA: Bonferroni-Holm Corrected<br>All curves: 431 cell groups x 4 distances<br><br>I Day 1 1 vs I day 7: p = 5.7242e-93, F = 19.345<br>D Day 1 vs D day 7: p = 2.3216e-05, F = 18.104<br>Day 1 I vs D: p = 2.0698e-72, F = 395.58<br>Day 7 I vs D: p = 4.2931e-93, F = 543.63
6D (right)	Two-way ANOVA:<br>Day: p = 5.2373e-22, F = 95.581<br>Landmark Pair Type: p = 1.9309e-281, F = 1910.3<br>Day x Landmark Pair Type: p = 0.070357, F = 3.2787<br><br>Two-tailed paired t-tests: Bonferroni-Holm Corrected<br><br>Each violin contains n = 1724 rank correlations across all cell groups and all four matched distances.<br><br>I Day 1 vs I day 7: p = 2.5023e-10<br>D Day 1 vs D day 7: p = 3.7074e-14<br>Day 1 I vs D: p = 5.9273e-169<br>Day 7 I vs D: p = 6.6365e-212
6E (left)	Two-way ANOVA:<br>Both curves: 431 cell groups x 4 distances<br>p = 0.190403, F = 1.7172
6E (right)	Two-tailed paired t-test: Each violin contains n = 1724 changes in rank correlation across all cell groups and all four matched distances.<br><br>p = 0.070357
6F (left)	Two-way ANOVA:<br>Both curves: 431 cell groups x 4 distances<br>Day 1 vs day 7: p = 0.203307, F = 1.6209

6F (right)	Two-tailed paired t-tests: $p = 0.070357$, $n = 1724$ rank correlation differences across all cell groups and all four matched distances for both violins

**Figure 7**	
7B (left)	Two-way ANOVA: Bonferroni-Holm Corrected<br><br>All curves: 473 cells x 4 distances<br><br>I Day 1 vs I Day 7: $p = 7.44497e\text{-}06$, $F = 21.668$<br>D Day 1 vs D Day 7: $p = 1.50895e\text{-}05$, $F = 18.928$<br>Day 1 I vs D: $p = 9.26712e\text{-}06$, $F = 22.594$<br>Day 7 I vs D: $p = 7.99049e\text{-}06$, $F = 22.33$
7B (right)	Two-way ANOVA<br>Day: $p = 2.2793e\text{-}26$, $F = 116.49$<br>Landmark Pair Type: $p = 1.8139e\text{-}23$, $F = 102.46$<br>Day x Landmark Pair Type: $p = 0.88478$, $F = 0.021004$<br><br>Two-tailed paired t-tests: Bonferroni-Holm Corrected<br><br>Each violin contains $n = 1892$ integrated activity variations across all cells and all four matched distances<br><br>I Day 1 vs I Day 7: $p = 4.6109e\text{-}17$<br>D Track 1 vs D Day 7: $p = 1.6069e\text{-}15$<br>Day 1 I vs D: $p = 7.84076e\text{-}15$<br>Day 7 I vs D: $p = 5.35323e\text{-}13$
7C (left)	Two-way ANOVA:<br>Both curves: 437 cells x 4 distances<br>$p = 0.94423$, $F = 0.0048962$
7C (right)	Two-tailed paired t-test comparing integrated activity variation difference on day 1 vs day 7: $p_{day1, day7} = 0.884784$<br><br>Two-tailed paired t-test comparing integrated activity variation difference with 0 for both day 1 and day 7 condition, Bonferroni-Holm corrected:<br><br>$p_{day1, 0} = 7.84076e\text{-}15$<br>$p_{day7, 0} = 5.35323e\text{-}13$<br><br>Each violin contains $n = 1892$ integrated activity variation differences across all cells and all four matched distances.
7D (left)	Two-way ANOVA: Bonferroni-Holm Corrected

	All curves: 250 cells x 4 distances<br><br>I Day 1 vs I Day 7: $p = 0.773099$, $F = 0.75112$<br>D Day 1 vs D Day 7: $p = 0.890309$, $F = 0.01904$<br>Day 1 I vs D: $p = 0.234035$, $F = 3.5958$<br>Day 7 I vs D: $p = 0.812703$, $F = 1.215$
7D (right)	Two-way ANOVA<br>Day: $p = 0.17029$, $F = 1.8832$<br>Landmark Pair Type: $p = 0.011076$, $F = 6.4777$<br>Day x Landmark Pair Type: $p = 0.5177$, $F = 0.41878$<br><br>Two-tailed paired t-tests: Bonferroni-Holm Corrected<br><br>Each violin contains n = 1000 integrated activity variations across all cells and all four matched distances<br><br>I Day 1 vs I Day 7: $p = 0.372148$<br>D Track 1 vs D Day 7: $p = 0.539898$<br>Day 1 I vs D: $p = 0.0648758$<br>Day 7 I vs D: $p = 0.332728$
7E (left)	Two-way ANOVA:<br>Both curves: 250 cells x 4 distances<br>$p = 0.45657$, $F = 0.55518$
7E (right)	Two-tailed paired t-test comparing integrated activity variation difference on day 1 vs day 7: $p_{day1, day7} = 0.517697$<br><br>Two-tailed paired t-test comparing integrated activity variation difference with 0 for both day 1 and day 7 condition, Bonferroni-Holm corrected:<br><br>$p_{day1, 0} = 0.0324379$<br>$p_{day7, 0} = 0.166364$<br><br>Each violin contains n = 1000 integrated activity variation differences across all cells and all four matched distances.
7F	Each violin for cue cells contains n = 1892. Each violin for putative grid cells contains n = 1000. Units for both are integral activity variations across all appropriate cells and all four matched distances.<br><br>Two-tailed non-paired t-tests: Bonferroni-Holm corrected<br><br>Day 1 Identical Cue vs Putative Grid: $p = 1.04762e-15$<br>Day 1 Disparate Cue vs Putative Grid: $p = 1.77083e-05$<br>Day 7 Identical Cue vs Putative Grid: $p = 0.00333082$

	Day 7 Disparate Cue vs Putative Grid: p = 0.338633
7G	Each violin for cue cells contains n = 1892. Each violin for putative grid cells contains n = 1000. Units for both are integral activity variation differences across all appropriate cells and all four matched distances.<br><br>Two-tailed non-paired t-tests comparing cue and putative grid cells: Bonferroni-Holm corrected<br><br>Day 1 Cue vs Putative Grid: p = 0.0117133<br>Day 7 Cue vs Putative Grid: 0.00588861
7H	Two-tailed paired t-test comparing integrated activity variation difference with 0 for both day 1 and day 7 condition, independently Bonferroni-Holm corrected for each activity range and functional class (i.e. as in Fig. 7C (right) and Fig. 7E (right)).

Cue:

Activity range (cm)	p-value, day 1	p-value, day 7
5	1.1684e-11	7.3127e-07
10	7.5601e-13	5.5165e-10
15	4.8072e-13	1.2703e-11
20	1.6466e-14	1.4316e-12
25	7.8408e-15	5.3532e-13
30	2.1574e-14	4.8219e-13
35	3.0084e-14	5.5234e-14
40	1.4262e-14	3.2451e-14
45	1.1919e-14	7.2625e-14
50	3.4445e-14	3.0239e-14

Putative Grid:

Activity range (cm)	p-value, day 1	p-value, day 7
5	0.0254	0.2499
10	0.0337	0.0766
15	0.0308	0.1787
20	0.0253	0.2790
25	0.0324	0.1664
30	0.0915	0.1443
35	0.1927	0.1133
40	0.1349	0.1490
45	0.1255	0.1818
50	0.1489	0.1853

Figure S1	
S1A	Track 1:<br>Two-way ANOVA, both curves: 434 cells x 4 distances<br>p = 0.00013977, F = 14.645<br><br>Two-tailed paired t-test: p = 1.8311e-09, n = 1736 activity variations across all cells and matched distances for both violins<br><br>Track 2:<br>Two-way ANOVA, both curves: 465 cells x 4 distances<br>p = 1.4375e-14, F = 61.218<br><br>Two-tailed paired t-test: p = 4.2448e-34, n = 1860 activity variations across all cells and matched distances for both violins<br><br>Track 3:<br>Two-way ANOVA, both curves: 508 cells x 4 distances<br>p = 6.65e-18, F = 77.58<br><br>Two-tailed paired t-test: p = 3.5323e-45, n= 2032 activity variations across all cells and matched distances for both violins
S1B (right panels)	Two-tailed paired t-tests comparing activity variation difference to 0:<br><br>Track 1: p = 1.8311e-09, n = 1736<br>Track 2: p = 4.2448e-34, n = 1860<br>Track 3: p = 3.5323e-45, n = 2032<br><br>Units for all n are activity variation differences across all cells and matched distances.

Figure S2	
S2A	Track 1:<br><br>Two-way ANOVA, both curves: 10 FOVs x 4 distances<br>p = 0.090107, F = 3.2081<br><br>Two-tailed paired t-test, both violins have n = 40 activity variations across all FOVs and four matched distances<br>p = 0.00021335<br><br><br>Track 2:

	Two-way ANOVA, both curves: 10 FOVs x 4 distances<br>p = 0.0069763, F = 9.2687<br><br>Two-tailed paired t-test, both violins have n = 40 activity variations across all FOVs and four matched distances<br>p = 1.7882e-11<br><br><br>Track 3:<br><br>Two-way ANOVA, both curves: 10 FOVs x 4 distances<br>p = 0.00077302, F = 16.299<br><br>Two-tailed paired t-test, both violins have n = 40 activity variations across all FOVs and four matched distances<br>p = 4.1084e-08
S2B	Two-tailed paired t-tests comparing activity variation difference to 0:<br><br>Track 1: p = 0.00021335, n = 40<br>Track 2: p = 1.7882e-11, n = 40<br>Track 3: p = 4.1084e-08, n = 40<br><br>Units for all n are activity variation differences across all FOVs and all four matched distances.
S2C	Track 1:<br>Two-way ANOVA, both curves: 5 mice x 4 distances<br>p = 0.23804, F = 1.626<br><br>Two-tailed paired t-test, both violins have n = 20 activity variations across all mice and four matched distances<br>p = 0.0021079<br><br><br>Track 2:<br>Two-way ANOVA, both curves: 5 mice x 4 distances<br>p = 0.061144, F = 4.7395<br><br>Two-tailed paired t-test, both violins have n = 20 activity variations across all mice and four matched distances<br>p = 3.9388e-07

	Track 3:<br>Two-way ANOVA, both curves: 5 mice x 4 distances<br>$p = 0.024459$, $F = 7.649$<br><br>Two-tailed paired t-test, both violins have $n = 20$ activity variations across all mice and four matched distances<br>$p = 0.0001024$
S2D	Two-tailed paired t-tests comparing activity variation difference to 0:<br><br>Track 1: $p = 0.0021079$, $n = 20$<br>Track 2: $p = 3.9388e\text{-}07$, $n = 20$<br>Track 3: $p = 0.0001024$, $n = 20$<br><br>Units for all n are activity variation differences across all mice and all four matched distances.

**Figure S3**	
S3A	Track 1: Left, Two-tailed Pearson's linear correlation<br>$r = 0.62797$, $p = 7.1494e\text{-}49$, $n = 434$ cells with identical and disparate coordinates<br><br>Track 1: Right, Two-tailed paired t-test<br>$p = 0.064064$, $n = 10$ FOV in both conditions<br><br>Track 2: Left, Two-tailed Pearson's linear correlation<br>$r = 0.45081$, $p = 1.1742e\text{-}24$, $n = 465$ cells with identical and disparate coordinates<br><br>Track 2: Right, Two-tailed paired t-test<br>$p = 0.0044211$, $n = 10$ FOV in both conditions<br><br>Track 3: Left, Two-tailed Pearson's linear correlation<br>$r = 0.42264$, $p = 2.9614e\text{-}23$, $n = 508$ cells with identical and disparate coordinates<br><br>Track 3: Right, Two-tailed paired t-test<br>$p = 1.3803e\text{-}05$, $n = 10$ FOV in both conditions

Figure S4	
S4C	Two-tailed non-paired t-tests comparing Identical (I) and Disparate (D) conditions (violin plots):<br><br>Track 4: $p = 2.9962e\text{-}06$, $n_I = 996$, $n_D = 3486$<br>Track 5: $p = 2.9578e\text{-}15$, $n_I = 1340$, $n_D = 8710$<br><br>Units for all n are activity variations across all cells and all appropriate distances.
S4D	Two-tailed non-paired t-tests comparing Identical (I) and Disparate (D) conditions (violin plots):<br><br>Track 4: $p = 3.0577e\text{-}08$, $n_I = 996$, $n_D = 1992$<br>Track 5: $p = 2.5645e\text{-}11$, $n_I = 1340$, $n_D = 2680$<br><br>Units for all n are activity variations across all cells and all appropriate distances.
S4E	Two-tailed non-paired t-tests comparing Identical (I) and Disparate (D) conditions (violin plots):<br><br>Track 4: $p = 2.9482e\text{-}58$, $n_I = 808$, $n_D = 2828$<br>Track 5: $p = 3.8888e\text{-}138$, $n_I = 1340$, $n_D = 8710$<br><br>Units for all n are rank correlations across all cell groups and all appropriate distances.
S4F	Two-tailed non-paired t-tests comparing Identical (I) and Disparate (D) conditions (violin plots):<br><br>Track 4: $p = 1.2003e\text{-}87$, $n_I = 808$, $n_D = 1616$<br>Track 5: $p = 3.2027e\text{-}95$, $n_I = 1340$, $n_D = 2680$<br><br>Units for all n are rank correlations across all cell groups and all appropriate distances.

Figure S5	
S5C	Units for all n are activity variations across all appropriate cells and distances.<br><br>Track 1:<br>Two-tailed Pearson's linear correlation between distance and mean activity variation:<br><br>Identical: $r = -0.13751$, $p = 0.76875$, 7 distances and associated mean activity variations

	Disparate: r = -0.093998, p = 0.82479, 8 distances and associated mean activity variations<br><br>Two-tailed non-paired t-test (violin plot):<br>p = 5.098e-07, n_I = 3038, n_D = 3472<br><br><br>Track 2:<br>Two-tailed Pearson's linear correlation between distance and mean activity variation:<br>Identical: r = -0.21033, p = 0.65078, 7 distances and associated mean activity variations<br>Disparate: r = 0.46009, p = 0.25134, 8 distances and associated mean activity variations<br><br>Two-tailed non-paired t-test (violin plot):<br>p = 9.2716e-49, n_I = 3255, n_D = 3720<br><br><br>Track 3:<br>Two-tailed Pearson's linear correlation between distance and mean activity variation:<br>Identical: r = 0.42929, p = 0.39563, 6 distances and associated mean activity variations<br>Disparate: r = -0.49236, p = 0.17815, 9 distances and associated mean activity variations<br><br>Two-tailed non-paired t-test (violin plot):<br>p = 1.2275e-44, n_I = 3048, n_D = 4572
S5D	Units for all n are activity variation differences across all cells and all appropriate distances.<br><br>Track 1:<br>Two-tailed Pearson's linear correlation between distance and mean activity variation difference:<br>r = -0.24862, p = 0.84005, 3 distances and associated mean activity variation difference values<br><br>Two-tailed paired t-test compared to 0 (violin plot):<br>p = 0.0013558, n = 1302

	Track 2:<br>Two-tailed Pearson's linear correlation between distance and mean activity variation difference:<br>r = 0.82684, p = 0.084218, 5 distances and associated mean activity variation difference values<br><br>Two-tailed paired t-test compared to 0 (violin plot):<br>p = 7.51e-45, n = 2325<br><br><br>Track 3:<br>Two-tailed Pearson's linear correlation between distance and mean activity variation difference:<br>r = -0.91641, p = 0.26215, 3 distances and associated mean activity variation difference values<br><br>Two-tailed paired t-test compared to 0 (violin plot):<br>p = 6.2312e-55, n = 1524
S5E	Units for all n are rank correlations across all cell groups and all appropriate distances.<br><br>Track 1:<br>Two-tailed Pearson's linear correlation between distance and mean rank correlation:<br><br>Identical: r = -0.0634, p = 0.89259, 7 distances and associated mean rank correlations<br>Disparate: r = -0.2012, p = 0.6328, 8 distances and associated mean rank correlations<br><br>Two-tailed non-paired t-test (violin plot):<br>p = 5.457e-33, n_I = 3038, n_D = 3472<br><br><br>Track 2:<br>Two-tailed Pearson's linear correlation between distance and mean rank correlation:<br>Identical: r = 0.060136, p = 0.89809, 7 distances and associated mean rank correlations<br>Disparate: r = -0.44872, p = 0.26476, 8 distances and associated mean rank correlations<br><br>Two-tailed non-paired t-test (violin plot):

	p: reported as 0 by MATLAB ttest2 function, $n_I = 3255$, $n_D = 3720$<br><br>Track 3:<br>Two-tailed Pearson's linear correlation between distance and mean rank correlation:<br>Identical: r = -0.45101, p = 0.36936, 6 distances and associated mean rank correlations<br>Disparate: r = -0.12656, p = 0.74558, 9 distances and associated mean rank correlations<br><br>Two-tailed non-paired t-test (violin plot):<br>p = 6.2653e-187, $n_I = 3048$, $n_D = 4572$
S5F	Units for all n are rank correlation differences across all cell groups and all appropriate distances.<br><br>Track 1:<br>Two-tailed Pearson's linear correlation between distance and mean rank correlation difference:<br>r = 0.52438, p = 0.6486, 3 distances and associated mean rank correlation difference values<br><br>Two-tailed paired t-test compared to 0 (violin plot):<br>p = 1.2426e-12, n = 1302<br><br>Track 2:<br>Two-tailed Pearson's linear correlation between distance and mean rank correlation difference:<br>r = -0.68708, p = 0.19997, 5 distances and associated mean rank correlation difference values<br><br>Two-tailed paired t-test compared to 0 (violin plot):<br>p = 2.3941e-300, n = 2325<br><br>Track 3:<br>Two-tailed Pearson's linear correlation between distance and mean rank correlation difference:<br>r = 0.67403, p = 0.52913, 3 distances and associated mean rank correlation difference values

	Two-tailed paired t-test compared to 0 (violin plot):<br>p = 2.2025e-146, n = 1524

**Figure S6**	
S6A	All dots represent p-values from two-tailed paired t-tests comparing rank correlation difference to 0. Units for all n are rank correlations across all cell groups and all four matched distances.<br><br>Track 1:<br><br>Two-way ANOVA, both curves: 434 cell groups x 4 distances<br>p = 2.1847e-18, F = 80.046<br><br>Two-tailed paired t-test, both violins have n = 1736<br>p = 1.7061e-40<br><br>Track 2:<br><br>Two-way ANOVA, both curves: 465 cell groups x 4 distances<br>p = 1.0181e-107, F = 639.27<br><br>Two-tailed paired t-test, both violins have n = 1860<br>p = 3.3871e-233<br><br>Track 3:<br><br>Two-way ANOVA, both curves: 508 cell groups x 4 distances<br>p = 1.1864e-47, F = 289.35<br><br>Two-tailed paired t-test, both violins have n = 2032<br>p = 4.3454e-155
S6B	Two-tailed paired t-test comparing rank correlation difference with 0:<br><br>Track 1: p = 1.7061e-40, n = 1736<br>Track 2: p = 3.3871e-233, n = 1860<br>Track 3: p = 4.3454e-155, n = 2032<br><br>Units for all n are rank correlation differences across all cell groups and all four matched distances.

**Figure S7**	
S7A	Units for all n are rank correlations across all FOVs and all four matched distances.<br><br>Track 1:<br><br>Two-way ANOVA, both curves: 10 FOVs x 4 distances<br>p = 0.16247, F = 2.1214<br><br>Two-tailed paired t-test, both violins have n = 40<br>p = 0.048566<br><br><br>Track 2:<br><br>Two-way ANOVA, both curves: 10 FOVs x 4 distances<br>p = 1.418e-05, F = 34.671<br><br>Two-tailed paired t-test, both violins have n = 40<br>p = 3.1942e-10<br><br>Track 3:<br><br>Two-way ANOVA, both curves: 10 FOVs x 4 distances<br>p = 1.6111e-05, F = 33.948<br><br>Two-tailed paired t-test, both violins have n = 40<br>p = 4.7137e-08
S7B	Units for all n are rank correlation differences across all FOVs and all four matched distances. Two-tailed paired t-tests comparing rank correlation difference to 0:<br><br>Track 1: p = 0.048566, n = 40<br>Track 2: p = 3.1942e-10, n = 40<br>Track 3: p = 4.7137e-08, n = 40
S7C	Units for all n are rank correlation across all mice and all four matched distances.<br><br>Track 1:<br>Two-way ANOVA, both curves: 5 mice x 4 distances<br>p = 0.34362, F = 1.0131

	Two-tailed paired t-test, both violins have n = 20<br>p = 0.12978<br><br><br>Track 2:<br>Two-way ANOVA, both curves: 5 mice x 4 distances<br>p = 0.0022754, F = 19.393<br><br>Two-tailed paired t-test, both violins have n = 20<br>p = 4.734e-06<br><br><br>Track 3:<br>Two-way ANOVA, both curves: 5 mice x 4 distances<br>p = 0.0012794, F = 23.478<br><br>Two-tailed paired t-test, both violins have n = 20<br>p = 5.0255e-05
S7D	Two-tailed paired t-tests comparing activity variation difference to 0:<br><br>Track 1: p = 0.12978, n = 20<br>Track 2: p = 4.734e-06, n = 20<br>Track 3: p = 5.0255e-05, n = 20<br><br>Units for all n are rank correlation differences across all mice and all four matched distances.

**Figure S8**	
S8A	Track 1: Left, Two-tailed Pearson's linear correlation<br>r = 0.59735, p = 2.5435e-43, n = 434 cell groups with identical and disparate coordinates<br><br>Track 1: Right, Two-tailed paired t-test<br>p = 0.063877, n = 10 FOV in both conditions<br><br><br>Track 2: Left, Two-tailed Pearson's linear correlation<br>r = 0.12273, p = 0.0080632, n = 465 cell groups with identical and disparate coordinates<br><br><br>Track 2: Right, Two-tailed paired t-test

	p = 5.7445e-07, n = 10 FOV in both conditions<br><br><br>Track 3: Left, Two-tailed Pearson's linear correlation<br>r = 0.37114, p = 4.905e-18, n = 508 cell groups with identical and disparate coordinates<br><br><br>Track 3: Right, Two-tailed paired t-test<br>p = 1.1363e-05, n = 10 FOV in both conditions
S8B	Rank correlations listed at each landmark are the two-tailed Pearson's linear correlations of each landmark's ranking of the same 60 (track 1) or 78 (track 3) cells compared to the reference landmarks.

**Figure S9**	
S9B	Track 1:<br>One-way ANOVA considering effect of Spatial Shift Cell Type (Before / At / After): p = 1.1258e-07, F = 44.248, n = 10 FOVs for each Cell Type<br><br>Bonferroni-Holm-corrected post-hoc two-tailed paired t-tests:<br>$p_{Before-At}$ = 5.8895e-09<br>$p_{At-After}$ = 0.0015403<br>$p_{Before-After}$ = 0.014656<br><br><br>Track 2:<br>One-way ANOVA considering effect of Spatial Shift Cell Type (Before / At / After): p = 9.3813e-08, F = 45.338, n = 10 FOVs for each Cell Type<br><br>Bonferroni-Holm-corrected post-hoc two-tailed paired t-tests:<br>$p_{Before-At}$ = 9.0601e-06<br>$p_{At-After}$ = 3.2162e-06<br>$p_{Before-After}$ = 0.088505<br><br><br>Track 3:<br>One-way ANOVA considering effect of Spatial Shift Cell Type (Before / At / After): p = 8.1217e-07, F = 33.751, n = 10 FOVs for each Cell Type<br><br>Bonferroni-Holm-corrected post-hoc two-tailed paired t-tests:<br>$p_{Before-At}$ = 2.2564e-05<br>$p_{At-After}$ = 1.5099e-05

	$p_{Before\text{-}After} = 0.11324$
S9C	Bonferroni-Holm p-value correction conducted between before, at, and after-cue conditions:<br><br>Before cue line plot:<br>Two-way ANOVA, both curves: 228 cells x 4 distances<br>$p = 0.095348$, $F = 2.7939$<br><br>At cue line plot:<br>Two-way ANOVA, both curves: 41 cells x 4 distances<br>$p = 0.029738$, $F = 6.2277$<br><br>After cue line plot:<br>Two-way ANOVA, both curves: 165 cells x 4 distances<br>$p = 0.0095121$, $F = 8.8471$<br><br><br>Bonferroni-Holm p-value correction conducted between before, at, and after-cue conditions:<br><br>Two-tailed paired t-test comparing identical and disparate landmarks:<br>Before: $p = 0.0019071$, $n_I = n_D = 912$<br>At: $p = 0.00071997$, $n_I = n_D = 164$<br>After: $p = 6.4302e-05$, $n_I = n_D = 660$<br><br>Units for above n are activity variations across all cells and all four matched distances.
S9D	Bonferroni-Holm p-value correction conducted between before, at, and after-cue conditions:<br><br>Before cue line plot:<br>Two-way ANOVA, both curves: 238 cells x 4 distances<br>$p = 9.3434e-17$, $F = 77.164$<br><br>At cue line plot:<br>Two-way ANOVA, both curves: 29 cells x 4 distances<br>$p = 0.11305$, $F = 2.5948$<br><br>After cue line plot:<br>Two-way ANOVA, both curves: 198 cells x 4 distances<br>$p = 0.097388$, $F = 3.9112$

	Bonferroni-Holm p-value correction conducted between before, at, and after-cue conditions:<br><br>Two-tailed paired t-test comparing identical and disparate landmarks:<br>Before: $p = 1.0419e-38$, $n_I = n_D = 952$<br>At: $p = 0.035507$, $n_I = n_D = 116$<br>After: $p = 0.001591$, $n_I = n_D = 792$<br><br>Units for above n are activity variations across all cells and all four matched distances.
S9E	Bonferroni-Holm p-value correction conducted between before, at, and after-cue conditions:<br><br>Before cue line plot:<br>Two-way ANOVA, both curves: 268 cells x 4 distances<br>$p = 1.2584e-12$, $F = 55.451$<br><br>At cue line plot:<br>Two-way ANOVA, both curves: 32 cells x 4 distances<br>$p = 0.01338$, $F = 6.5109$<br><br>After cue line plot:<br>Two-way ANOVA, both curves: 208 cells x 4 distances<br>$p = 7.0638e-05$, $F = 17.605$<br><br>Bonferroni-Holm p-value correction conducted between before, at, and after-cue conditions:<br><br>Two-tailed paired t-test comparing identical and disparate landmarks:<br>Before: $p = 1.1005e-26$, $n_I = n_D = 1072$<br>At: $p = 2.5322e-07$, $n_I = n_D = 128$<br>After: $p = 1.8895e-14$, $n_I = n_D = 832$<br><br>Units for above n are activity variations across all cells and all four matched distances.

**Figure S10**	
S10A	All dots represent p-values from two-tailed Pearson's linear correlations of spatial shifts ≥ 0 which have at least 5 cells along with their associated averaged activity variation difference.

S10B	All correlations below are two-tailed Pearson's linear correlations of spatial shifts which have at least 3 cells along with their associated averaged activity variation difference. The analysis was conducted separately for positive-shift and negative-shift cells for each mouse and thus involves a lower cell number threshold due to the reduced number of cells.

	Mouse 1	Mouse 2	Mouse 3	Mouse 4	Mouse 5
Track 1	Positive shift: r = -0.77141 p = 0.024975<br><br>Negative shift: r = 0.82341 p = 0.044024	Positive shift: r = -0.90083 p = 0.0056356<br><br>Negative shift: r = -0.20972 p = 0.73495	Positive shift: r = -0.77163 p = 0.072275<br><br>Negative shift: r = 0.91099 p = 0.27065	Positive shift: r = -0.090727 p = 0.86428<br><br>Negative shift: r = -0.63661 p = 0.12421	Positive shift: r = -0.045769 p = 0.92238<br><br>Negative shift: r = 0.31923 p = 0.44087
Track 2	Positive shift: r = 0.53869 p = 0.13455<br><br>Negative shift: r = -0.12604 p = 0.76617	Positive shift: r = 0.63095 p = 0.12866<br><br>Negative shift: r = 0.62028 p = 0.2643	Positive shift: r = 0.6037773 p = 0.0851236<br><br>Negative shift: r =1 p = N/A	Positive shift: r = 0.04851 p = 0.90919<br><br>Negative shift: r = 0.67587 p = 0.095579	Positive shift: r = 0.65011 p = 0.11393<br><br>Negative shift: r = 0.67974 p = 0.063659
Track 3	Positive shift: r = -0.46566 p = 0.17499<br><br>Negative shift: r =	Positive shift: r = -0.42432 p = 0.47642<br><br>Negative shift: r = 0.62166 p = 0.26293	Positive shift: r = -0.7220554 p = 0.02804256<br><br>Negative shift: r = 1	Positive shift: r = -0.5363 p = 0.21462<br><br>Negative shift: r =	Positive shift: r = -0.26068 p = 0.57233<br><br>Negative shift: r = 0.34159 p = 0.36828

		-0.13778<br>p =<br>0.76832		p = N/A	0.69717<br>p =<br>0.12368	

**Figure S11**	
S11B	Two-tailed Pearson's linear correlation of spatial shifts between tracks; coefficient of determination (R^2) for linear fit as well as p-value shown below.<br><br>Track 1 vs 2: R^2 = 0.00015496, p = 0.81417, n = 359 cells<br><br>Track 1 vs 3: R^2 = 0.01339, p = 0.021106, n = 397 cells<br><br>Track 2 vs 3: R^2 = 0.013953, p =0.023629, n = 367 cells
S11C	Two-tailed Pearson's linear correlation of cue scores between tracks; coefficient of determination (R^2) for linear fit as well as p-value shown below.<br><br>Track 1 vs 2: R^2 = 0.00042704, p = 0.69637, n = 359 cells<br><br>Track 1 vs 3: R^2 = 0.0010879, p = 0.51228, n = 397 cells<br><br>Track 2 vs 3: R^2 = 0.0037712, p = 0.24058, n = 367 cells
S11E	Two-tailed Pearson's linear correlation of spatial shifts between days 1 and 7; coefficient of determination (R^2) for linear fit as well as p-value shown below.<br><br>Track 1: R^2 = 0.40078, p = 1.0505e-25, n = 217 cells<br><br>Track 2: R^2 = 0.080259, p =4.1185e-06, n = 256 cells
S11F	Two-tailed Pearson's linear correlation of cue scores between days 1 and 7; coefficient of determination (R^2) for linear fit as well as p-value shown below.<br><br>Track 1: R^2 = 0.026202, p = 0.017011, n = 217 cells<br><br>Track 2: R^2 = 0.12891, p = 3.3212e-09, n = 256 cells

**Figure S12**	
S12A	Track 1 and 2: Two-tailed paired t-test: p = 4.6488e-05, n = 10 FOVs

	Track 1 and 3: Two-tailed paired t-test: p = 4.7637e-05, n = 10 FOVs<br>Track 2 and 3: Two-tailed paired t-test: p = 1.0772e-04, n = 10 FOVs

**Figure S13**	
S13A (left)	Track 1 vs Track 3:<br><br>Two-way ANOVA: Bonferroni-Holm Corrected<br><br>All curves: 397 cells x 4 distances<br><br>I Track 1 vs I track 3: p = 0.02265, F = 5.217<br>D Track 1 vs D track 3: p = 0.0089406, F = 8.1319<br>Track 1 I vs D: p = 0.0050429, F = 9.94<br>Track 3 I vs D: p = 1.2495e-15, F = 69.945<br><br>Track 2 vs Track 3:<br><br>Two-way ANOVA: Bonferroni-Holm Corrected<br><br>All curves: 367 cells x 4 distances<br><br>I Track 2 vs I track 3: p = 0.62957, F = 0.23285<br>D Track 2 vs D track 3: p = 0.013575, F = 7.3729<br>Track 2 I vs D: p = 2.2796e-06, F = 24.898<br>Track 3 I vs D: p = 4.0895e-12, F = 52.855
S13A (right)	Track 1 vs Track 3:<br><br>Two-way ANOVA<br>Track: p = 0.51604, F = 0.422<br>Landmark Pair Type: p = 1.7168e-31, F = 142.99<br>Track x Landmark Pair Type: p = 1.1043e-08, F = 33.032<br><br>Two-tailed paired t-tests: Bonferroni-Holm Corrected<br><br>Each violin contains n = 1588 activity variations across all cells and all four matched distances.<br><br>I Track 1 vs I track 3: p = 0.0012395<br>D Track 1 vs D track 3: p = 9.1173e-05<br>Track 1 I vs D: p = 1.888e-05

	Track 3 I vs D: p = 4.1946e-34<br><br><br><br>Track 2 vs Track 3:<br><br>Two-way ANOVA<br>Track: p = 0.0024311, F = 9.2268<br>Landmark Pair Type: p = 1.6959e-39, F = 184.64<br>Track x Landmark Pair Type: p = 0.029563, F = 4.7446<br><br>Two-tailed paired t-tests: Bonferroni-Holm Corrected<br><br>Each violin contains n = 1468 activity variations across all cells and all four matched distances.<br><br>I Track 2 vs I track 3: p = 0.34098<br>D Track 2 vs D track 3: p = 8.969e-05<br>Track 2 I vs D: p = 1.168e-15<br>Track 3 I vs D: p = 7.6838e-26
S13B	Track 1 vs Track 3:<br><br>Each curve contains 397 cells x 4 distances.<br><br>p = 0.00046226, F = 12.38<br>Two-tailed paired t-test: p = 1.1043e-08, n = 1588 changes in activity variation across all cells and all four matched distances for both violins<br><br><br>Track 2 vs Track 3:<br><br>Each curve contains 367 cells x 4 distances.<br><br>p = 0.095837, F = 2.7816<br><br>Two-tailed paired t-test: p = 0.029563, n = 1468 changes in activity variation across all cells and all four matched distances for both violins
S13C	Track 1 vs Track 3:<br><br>Two-way ANOVA:<br>Each curve contains 397 cells x 4 distances.

	p = 1.6707e-05, F = 18.783<br><br>Two-tailed paired t-test:<br>p = 1.1573e-07, n = 1588 activity variation differences across all cells and all four matched distances for both violins<br><br>Track 2 vs Track 3:<br><br>Two-way ANOVA:<br>Each curve contains 367 cells x 4 distances.<br><br>p = 0.070139, F = 3.2901<br><br>Two-tailed paired t-test:<br>p = 0.045044, n = 1468 activity variation differences across all cells and all four matched distances for both violins

**Figure S14**	
S14A (left)	Track 1 vs Track 3:<br><br>Two-way ANOVA: Bonferroni-Holm Corrected<br><br>All curves: 397 cell groups x 4 distances<br><br>I Track 1 vs I track 3: p = 0.11852, F = 2.4449<br>D Track 1 vs D track 3: p = 2.8478e-08, F = 33.217<br>Track 1 I vs D: p = 1.2067e-13, F = 59.309<br>Track 3 I vs D: p = 1.5441e-30, F = 182.58<br><br>Track 2 vs Track 3:<br><br>Two-way ANOVA: Bonferroni-Holm Corrected<br><br>All curves: 367 cell groups x 4 distances<br><br>I Track 2 vs I track 3: p = 0.45688, F = 0.55447

	D Track 2 vs D track 3: p = 0.0075801, F = 8.4655<br>Track 2 I vs D: p = 7.0523e-70, F = 395.9<br>Track 3 I vs D: p = 5.0033e-35, F = 227.53
S14A (right)	Track 1 vs Track 3:<br><br>Two-way ANOVA<br>Track: p = 9.4017e-23, F = 102.94<br>Landmark Pair Type: p = 8.1029e-104, F = 650.61<br>Track x Landmark Pair Type: p = 3.9908e-52, F = 270.46<br><br>Two-tailed paired t-tests: Bonferroni-Holm Corrected<br><br>Each violin has n = 1588 rank correlations across all cell groups and all four matched distances.<br><br>I Track 1 vs I track 3: p = 0.7133<br>D Track 1 vs D track 3: p = 1.6814e-44<br>Track 1 I vs D: p = 3.9648e-42<br>Track 3 I vs D: p = 1.2247e-131<br><br>Track 2 vs Track 3:<br><br>Two-way ANOVA<br>Track: p = 8.4111e-10, F = 41.265<br>Landmark Pair Type: p = 0.026566, F = 4.9872<br>Track x Landmark Pair Type: p = 1.8923e-09, F = 39.384<br><br>Two-tailed paired t-tests: Bonferroni-Holm Corrected<br><br>Each violin has n = 1468 rank correlations across all cell groups and all four matched distances.<br><br>I Track 2 vs I track 3: p = 0.13748<br>D Track 2 vs D track 3: p = 1.285e-09<br>Track 2 I vs D: p = 1.5817e-137<br>Track 3 I vs D: p = 8.8501e-144
S14B	Track 1 vs Track 3:<br><br>Each curve contains 397 cell groups x 4 distances.<br>p = 7.1018e-11, F = 46.7

	Two-tailed paired t-test:<br>p = 3.9908e-52, n = 1588 changes in rank correlation across all cell groups and all four matched distances for both violins<br><br><br>Track 2 vs Track 3:<br><br>Each curve contains 367 cell groups x 4 distances.<br>p = 0.0031712, F = 8.9069<br><br>Two-tailed paired t-test:<br>p = 1.9651e-31, n = 1468 changes in rank correlation across all cell groups and all four matched distances for both violins
S14C	Track 1 vs Track 3:<br><br>Two-way ANOVA:<br>Each curve contains 397 cell groups x 4 distances.<br>p = 1.7817e-15, F = 67.432<br><br>Two-tailed paired t-test: p = 3.124e-50, n = 1588 rank correlation differences across all cell groups and all four matched distances for both violins<br><br><br>Track 2 vs Track 3:<br><br>Two-way ANOVA:<br>Each curve contains 367 cell groups x 4 distances.<br>p = 0.0024614, F = 9.2717<br><br>Two-tailed paired t-test: p = 1.9983e-23, n = 1468 rank correlation differences across all cell groups and all four matched distances for both violins

**Figure S15**	
S15A	Two-tailed paired t-test comparing activity variation difference per cell for all track pairs:<br><br>Track 1 vs Track 2: p = 0.0021016, n = 359 cells<br>Track 1 vs Track 3: p = 1.1055e-05, n = 397 cells<br>Track 2 vs Track 3: p = 0.065155, n = 367 cells

S15B	Two-tailed paired t-test comparing rank correlation difference per cell group for all track pairs:<br><br>Track 1 vs Track 2: p = 3.075e-51, n = 359 cell groups<br>Track 1 vs Track 3: p = 4.2209e-44, n = 397 cell groups<br>Track 2 vs Track 3: p = 1.2942e-15, n = 367 cell groups
S15C	Each dot represents the p-value resulting from a two-tailed paired t-test comparing the rank correlation difference per cell group in two tracks for each track pair.

**Figure S16**	
S16A (left)	Two-way ANOVA: Bonferroni-Holm Corrected<br><br>All curves: 114 cells x 4 distances<br><br>I Track 1 vs I Track 2: p = 0.13603, F = 4.0508<br>D Track 1 vs D Track 2: p = 0.57032, F = 0.3231<br>Track 1 I vs D: p = 0.40444, F = 1.6358<br>Track 2 I vs D: p = 0.00055003, F = 15.048
S16A (right)	Two-way ANOVA<br>Track: p = 0.16745, F = 1.9117<br>Landmark Pair Type: p = 2.0393e-08, F = 32.617<br>Track x Landmark Pair Type: p = 0.0066157, F = 7.4434<br><br>Two-tailed paired t-tests: Bonferroni-Holm Corrected<br><br>Each violin contains n = 456 activity variations across all cells and all four matched distances.<br><br>I Track 1 vs I Track 2: p = 0.0087618<br>D Track 1 vs D Track 2: p = 0.46051<br>Track 1 I vs D: p = 0.0711<br>Track 2 I vs D: p = 1.0935e-08
S16B (left)	Two-way ANOVA:<br>Both curves: 114 cells x 4 distances<br>p = 0.072363, F = 3.2593
S16B (right)	Two-tailed paired t-test: p = 0.0066157, n = 456 changes in activity variation across all cells and all four matched distances for both violins
S16C (left)	Two-way ANOVA:<br>Both curves: 114 x 4 distances<br>Track 1 vs Track 2: p = 0.020681, F = 5.4302

S16C (right)	Two-tailed paired t-tests: p = 0.0079011, n = 456 activity variation differences across all cells and all four matched distances for both violins
S16D (left)	Two-way ANOVA: Bonferroni-Holm Corrected<br>All curves: 88 cell groups x 4 distances<br><br>I Track 1 vs I Track 2: p = 0.0062256, F = 7.6695<br>D Track 1 vs D Track 2: p = 0.00050052, F = 14.809<br>Track 1 I vs D: p = 0.010601, F = 7.9735<br>Track 2 I vs D: p = 6.4903e-16, F = 83.504
S16D (right)	Two-way ANOVA:<br>Track: p = 0.15954, F = 1.987<br>Landmark Pair Type: p = 5.5591e-30, F = 156.73<br>Track x Landmark Pair Type: p = 1.7066e-12, F = 53.581<br><br>Two-tailed paired t-tests: Bonferroni-Holm Corrected<br><br>Each violin contains n = 352 rank correlations across all cell groups and all four matched distances.<br><br>I Track 1 vs I Track 2: p = 0.00011414<br>D Track 1 vs D Track 2: p = 3.9786e-06<br>Track 1 I vs D: p = 0.00013132<br>Track 2 I vs D: p = 1.8638e-36
S16E (left)	Two-way ANOVA:<br>Both curves: 88 cell groups x 4 distances<br>p = 8.4896e-06, F = 21.061
S16E (right)	Two-tailed paired t-test: Each violin contains n = 352 changes in rank correlation across all cell groups and all four matched distances.<br><br>p = 1.7066e-12
S16F (left)	Two-way ANOVA:<br>Both curves: 88 cell groups x 4 distances<br>Track 1 vs Track 7: p = 1.6713e-08, F = 35.052
S16F (right)	Two-tailed paired t-tests: p = 2.9258e-11, n = 352 rank correlation differences across all cell groups and all four matched distances for both violins
S16G (left)	Two-way ANOVA: Bonferroni-Holm Corrected<br><br>All curves: 120 cells x 4 distances<br><br>I Track 1 vs I Track 3: p = 0.94837, F = 0.0042029<br>D Track 1 vs D Track 3: p = 0.00093355, F = 13.419<br>Track 1 I vs D: p = 0.069126, F = 4.5202<br>Track 3 I vs D: p = 1.2007e-07, F = 33.089

S16G (right)	Two-way ANOVA<br>Track: p = 0.0005448, F = 12.136<br>Landmark Pair Type: p = 5.7382e-17, F = 76.146<br>Track x Landmark Pair Type: p = 8.1014e-06, F = 20.403<br><br>Two-tailed paired t-tests: Bonferroni-Holm Corrected<br><br>Each violin contains n = 480 activity variations across all cells and all four matched distances.<br><br>I Track 1 vs I Track 3: p = 0.63945<br>D Track 1 vs D Track 3: p = 9.2666e-08<br>Track 1 I vs D: p = 0.00075923<br>Track 3 I vs D: p = 6.5324e-18
S16H (left)	Two-way ANOVA:<br>Both curves: 120 cells x 4 distances<br>p = 0.0041934, F = 8.3832
S16H (right)	Two-tailed paired t-test: p = 8.1014e-06, n = 480 changes in activity variation across all cells and all four matched distances for both violins
SR2I (left)	Two-way ANOVA:<br>Both curves: 120 cells x 4 distances<br>Track 1 vs Track 3: p = 0.0027233, F = 9.1913
S16I (right)	Two-tailed paired t-test: p = 3.5808e-05, n = 480 activity variation differences across all cells and all four matched distances for both violins
S16J (left)	Two-way ANOVA: Bonferroni-Holm Corrected<br>All curves: 82 cell groups x 4 distances<br><br>I Track 1 vs I Track 3: p = 0.00010366, F = 17.65<br>D Track 1 vs D Track 3: p = 7.1303e-05, F = 19.37<br>Track 1 I vs D: p = 5.0011e-07, F = 30.597<br>Track 3 I vs D: p = 0.00078599, F = 12.266
S16J (right)	Two-way ANOVA:<br>Track: p = 1.139e-15, F = 76.217<br>Landmark Pair Type: p = 3.8216e-22, F = 120.54<br>Track x Landmark Pair Type: p = 0.00073642, F = 11.765<br><br>Two-tailed paired t-tests: Bonferroni-Holm Corrected<br><br>Each violin contains n = 328 rank correlations across all cell groups and all four matched distances.<br><br>I Track 1 vs I Track 3: p = 6.8517e-06<br>D Track 1 vs D Track 3: p = 3.0666e-10

	Track 1 I vs D: p = 7.317e-14<br>Track 3 I vs D: p = 1.2215e-14
S16K (left)	Two-way ANOVA:<br>Both curves: 82 cell groups x 4 distances<br>p = 0.82227, F = 0.050819
S16K (right)	Two-tailed paired t-test: Each violin contains n = 328 changes in rank correlation across all cell groups and all four matched distances.<br><br>p = 0.00073642
S16L (left)	Two-way ANOVA:<br>Both curves: 82 cell groups x 4 distances<br>Track 1 vs Track 3: p = 0.3625, F = 0.83567
S16L (right)	Two-tailed paired t-tests: p = 0.011287, n = 328 rank correlation differences across all cell groups and all four matched distances for both violins
S16M (left)	Two-way ANOVA: Bonferroni-Holm Corrected<br><br>All curves: 127 cells x 4 distances<br><br>I Track 1 vs I Track 3: p = 0.11086, F = 2.5608<br>D Track 1 vs D Track 3: p = 0.16245, F = 3.0658<br>Track 1 I vs D: p = 4.8544e-05, F = 19.339<br>Track 3 I vs D: p = 1.4284e-05, F = 22.564
S16M (right)	Two-way ANOVA<br>Track: p = 0.00055922, F = 12.07<br>Landmark Pair Type: p = 4.8657e-23, F = 108.6<br>Track x Landmark Pair Type: p = 0.92115, F = 0.0098078<br><br>Two-tailed paired t-tests: Bonferroni-Holm Corrected<br><br>Each violin contains n = 508 activity variations across all cells and all four matched distances.<br><br>I Track 2 vs I Track 3: p = 0.0063519<br>D Track 2 vs D Track 3: p = 0.0055586<br>Track 2 I vs D: p = 7.6963e-11<br>Track 3 I vs D: p = 3.1026e-12
S16N (left)	Two-way ANOVA:<br>Both curves: 127 cells x 4 distances<br>p = 0.99872, F = 2.56e-06
S16N (right)	Two-tailed paired t-test: p = 0.92115, n = 508 changes in activity variation across all cells and all four matched distances for both violins
S16O (left)	Two-way ANOVA:<br>Both curves: 127 cells x 4 distances

	Track 2 vs Track 3: p = 0.69622, F = 0.1528
S16O (right)	Two-tailed paired t-tests: p = 0.9987, n = 508 activity variation differences across all cells and all four matched distances for both violins
S16P (left)	Two-way ANOVA: Bonferroni-Holm Corrected<br>All curves: 90 cell groups x 4 distances<br><br>I Track 2 vs I Track 3: p = 0.01748, F = 7.0785<br>D Track 2 vs D Track 3: p = 0.02974, F = 4.8252<br>Track 2 I vs D: p = 7.6264e-21, F = 118.26<br>Track 3 I vs D: p = 8.3284e-06, F = 24.889
S16P (right)	Two-way ANOVA:<br>Track: p = 0.00055922, F = 12.07<br>Landmark Pair Type: p = 4.8657e-23, F = 108.6<br>Track x Landmark Pair Type: p = 0.92115, F = 0.0098078<br><br>Two-tailed paired t-tests: Bonferroni-Holm Corrected<br><br>Each violin contains n = 360 rank correlations across all cell groups and all four matched distances.<br><br>I Track 2 vs I Track 3: p = 1<br>D Track 2 vs D Track 3: p = 0.72845<br>Track 2 I vs D: p = 1.6597e-37<br>Track 3 I vs D: p = 5.8143e-18
S16Q (left)	Two-way ANOVA:<br>Both curves: 90 cell groups x 4 distances<br>p = 0.072363, F = 3.3025
S16Q (right)	Two-tailed paired t-test: Each violin contains n = 360 changes in rank correlation across all cell groups and all four matched distances.<br><br>p = 0.49479
S16R (left)	Two-way ANOVA:<br>Both curves: 90 cell groups x 4 distances<br>Track 1 vs Track 7: p = 0.0053847, F = 8.0004
S16R (right)	Two-tailed paired t-tests: p = 0.4226, n = 360 rank correlation differences across all cell groups and all four matched distances for both violins

**Figure S17**	
S17A	Track 1: Two-tailed paired t-test: p = 2.8731e-04, n = 10 FOVs

	Track 2: Two-tailed paired t-test: p = 1.5393e-05, n = 10 FOVs
S17D (left)	Two-way ANOVA: Bonferroni-Holm Corrected<br><br>All curves: 173 cells x 4 distances<br><br>I Day 1 vs I Day 7: p = 0.0042486, F = 9.5913<br>D Day 1 vs D Day 7: p = 0.01162, F = 6.4399<br>Day 1 I vs D: p = 1. 1.5562e-05, F = 22.038<br>Day 7 I vs D: p = 1.3424e-05, F = 21.797
S17D (right)	Two-way ANOVA<br>Day: p = 1.6665e-12, F = 51.834<br>Landmark Pair Type: p = 5.9026e-24, F = 110.35<br>Day x Landmark Pair Type: p = 0.72544, F = 0.12344<br><br>Two-tailed paired t-tests: Bonferroni-Holm Corrected<br><br>Each violin contains n = 692 activity variations across all cells and all four matched distances.<br><br>I Day 1 vs I Day 7: p = 2.1084e-09<br>D Day 1 vs D Day 7: p = 7.7034e-07<br>Day 1 I vs D: p = 2.6593e-14<br>Day 7 I vs D: p = 5.3802e-12
S17E (left)	Two-way ANOVA:<br>Both curves: 173 cells x 4 distances<br>p = 0.87276, F = 0.025692
S17E (right)	Two-tailed paired t-test: p = 0.72544, n = 692 changes in activity variation across all cells and all four matched distances for both violins
S17F (left)	Two-way ANOVA:<br>Both curves: 173 cells x 4 distances<br>Day 1 vs Day 7: p = 0.7696, F = 0.085931
S17F (right)	Two-tailed paired t-tests: p = 0.72544, n = 692 activity variation differences across all cells and all four matched distances for both violins
S17G (left)	Two-way ANOVA: Bonferroni-Holm Corrected<br>All curves: 153 cell groups x 4 distances<br><br>I Day 1 vs I Day 2: p = 0.75717, F = 0.095776<br>D Day 1 vs D Day 2: p = 1, F = 0.19312<br>Day 1 I vs D: p = 8.8641e-12, F = 53.596<br>Day 7 I vs D: p = 2.7563e-11, F = 50.326
S17G (right)	Two-way ANOVA:<br>Day: p = 0.4329, F = 0.61586<br>Landmark Pair Type: p = 1.0574e-36, F = 183.25

	Day x Landmark Pair Type: p = 0.77457, F = 0.082099<br><br>Two-tailed paired t-tests: Bonferroni-Holm Corrected<br><br>Each violin contains n = 612 rank correlations across all cell groups and all four matched distances.<br><br>I Day 1 vs I Day 7: p = 0.70781<br>D Day 1 vs D Day 7: p = 0.91955<br>Day 1 I vs D: p = 4.2229e-24<br>Day 7 I vs D: p = 5.3678e-20
S17H (left)	Two-way ANOVA:<br>Both curves: 153 cell groups x 4 distances<br>p = 0.83261, F = 0.044747
S17H (right)	Two-tailed paired t-test: Each violin contains n = 612 changes in rank correlation across all cell groups and all four matched distances.<br><br>p = 0.77457
S17I (left)	Two-way ANOVA:<br>Both curves: 153 cell groups x 4 distances<br>Day 1 vs Day 7: p = 0.83356, F = 0.044235
S17I (right)	Two-tailed paired t-tests: p = 0.77457, n = 612 rank correlation differences across all cell groups and all four matched distances for both violins

**Figure S18**	
S18A (left)	Track 1:<br>Two-way ANOVA: Bonferroni-Holm Corrected<br>All curves: 217 cells x 4 distances<br><br>I Day 1 vs I Day 7: p = 0.011069, F = 8.5316<br>D Day 1 vs D Day 7: p = 0.011815, F = 8.9441<br>Day 1 I vs D: p = 0.011683, F = 6.4152<br>Day 7 I vs D: p = 0.012626, F = 7.5407<br><br><br>Track 2:<br>Two-way ANOVA: Bonferroni-Holm Corrected<br>All curves: 256 cells x 4 distances<br><br>I Day 1 vs I Day 7: p = 0.0022633, F = 10.727<br>D Day 1 vs D Day 7: p = 0.0031258, F = 8.8197<br>Day 1 I vs D: p = 2.8765e-06, F = 25.205

	Day 7 I vs D: p = 2.3687e-06, F = 25.045
S18A (right)	Track 1:<br><br>Two-way ANOVA<br>Day: p = 3.1146e-11, F = 45.377<br>Landmark Pair Type: p = 1.3853e-07, F = 28.25<br>Day x Landmark Pair Type: p = 0.95845, F = 0.0027166<br><br>Two-tailed paired t-tests: Bonferroni-Holm Corrected<br><br>Each violin contains n = 868 activity variations across all cells and all four matched distances.<br><br>I Day 1 vs I Day 7: p = 5.75e-07<br>D Track 1 vs D Day 7: p = 1.5883e-07<br>Day 1 I vs D: p = 4.3903e-05<br>Day 7 I vs D: p = 0.00015466<br><br>Track 2:<br><br>Two-way ANOVA<br>Day: p = 2.3318e-11, F = 45.74<br>Landmark pair type: p = 5.9054e-28, F = 127.97<br>Day x Landmark pair type: p = 0.54307, F = 0.37015<br><br>Two-tailed paired t-tests: Bonferroni-Holm Corrected<br><br>Each violin contains n = 1024 activity variations across all cells and all four matched distances.<br><br>I Day 1 vs I Day 7: p = 4.2811e-10<br>D Track 1 vs D Day 7: p = 1.8567e-06<br>Day 1 I vs D: p = 9.5307e-17<br>Day 7 I vs D: p = 1.0792e-14
S18B (left)	Track 1:<br>Two-way ANOVA: Both curves: 217 cells x 4 distances<br>p = 0.67262, F = 0.17884<br><br>Track 2:<br>Two-way ANOVA: Both curves: 256 cells x 4 distances<br>p = 0.97414, F = 0.0010519

S18B (right)	Track 1:<br>Two-tailed paired t-test: Each violin contains n = 868 changes in activity variation across all cells and all four matched distances.<br>p = 0.95845<br><br>Track 2:<br>Two-tailed paired t-test: Each violin contains n = 1024 changes in activity variation across all cells and all four matched distances.<br>p = 0.54307
S18C (left)	Track 1: Two-way ANOVA<br>Both curves: 217 cells x 4 distances<br>Day 1 vs Day 7: p = 0.989753, F = 0.00016517<br><br>Track 2: Two-way ANOVA:<br>Both curves: 256 cells x 4 distances<br>Day 1 vs Day 7: p = 0.727186, F = 0.12186
S18C (right)	Track 1: Two-tailed paired t-tests<br>p = 0.95845, n = 868 activity variation differences across all cells and all four matched distances for both violins<br><br>Track 2: Two-tailed paired t-tests:<br>p = 0.54307, n = 1024 activity variation differences across all cells and all four matched distances for both violins

**Figure S19**	
S19A (track 1, top)	Bonferroni-Holm corrected two-way ANOVA:<br><br>Before-cue curves: 91 cells x 4 distances<br>After-cue curves: 77 cells x 4 distances<br><br>Before-cue Identical Day 1 vs 7: p = 0.024607, F = 7.1568<br>Before-cue Disparate Day 1 vs 7: p = 0.01637, F = 8.4667<br>After-cue Identical Day 1 vs 7: p = 0.56698, F = 0.32933<br>After-cue Disparate Day 1 vs 7: p = 0.58373, F = 0.75918
S19A (track 2, top)	Bonferroni-Holm corrected two-way ANOVA:<br><br>Before-cue curves: 87 cells x 4 distances<br>After-cue curves: 73 cells x 4 distances<br><br>Before-cue Identical Day 1 vs 7: p = 0.018648, F = 8.2317<br>Before-cue Disparate Day 1 vs 7: p = 0.039405, F = 6.2838<br>After-cue Identical Day 1 vs 7: p = 0.65579, F = 0.19952

	After-cue Disparate Day 1 vs 7: p = 0.86068, F = 0.62552
S19A (track 1, bottom)	Bonferroni-Holm corrected two-tailed paired t-tests:<br><br>Before-cue Identical Day 1 vs 7: p = 2.2324e-07, n = 364 for both violins<br>Before-cue Disparate Day 1 vs 7: p = 3.0114e-06, n = 364 for both violins<br>After-cue Identical Day 1 vs 7: p = 0.20557, n = 308 for both violins<br>After-cue Disparate Day 1 vs 7: p = 0.025482, n = 308 for both violins<br><br>Units for all n are activity variations across all cells and all four matched distances.
S19A (track 2, bottom)	Bonferroni-Holm corrected two-tailed paired t-tests:<br><br>Before-cue Identical Day 1 vs 7: p = 6.3724e-06, n = 348 for both violins<br>Before-cue Disparate Day 1 vs 7: p = 4.1164e-05, n = 348 for both violins<br>After-cue Identical Day 1 vs 7: p = 0.42274, n = 292 for both violins<br>After-cue Disparate Day 1 vs 7: p = 0.38929, n = 292 for both violins<br><br>Units for all n are activity variations across all cells and all four matched distances.
S19B (track 1, line plots)	Bonferroni-Holm corrected two-way ANOVA:<br><br>Before-cue curves: 91 cells x 4 distances<br>After-cue curves: 77 cells x 4 distances<br><br>Before-cue Identical vs Disparate: p = 0.67973, F = 0.17105<br>After-cue Identical vs Disparate: p = 1, F = 0.4478
S19B (track 2, line plots)	Bonferroni-Holm corrected two-way ANOVA:<br><br>Before-cue curves: 87 cells x 4 distances<br>After-cue curves: 73 cells x 4 distances<br><br>Before-cue Identical vs Disparate: p = 0.93348, F = 0.006989<br>After-cue Identical vs Disparate: p = 1, F = 0.10253
S19B (track 1, violin plot)	Bonferroni-Holm corrected two-tailed paired t-tests:<br><br>Before-cue I vs Before-cue D: p = 0.74735, n = 364 for both violins<br>After-cue I vs After-cue D: p = 0.90033, n = 308 for both violins<br><br>Units for both n are changes in activity variation across all cells and all four matched distances.
S19B (track 2,	Bonferroni-Holm Corrected two-tailed paired t-tests<br><br>Before-cue I vs Before-cue D: p = 0.94809, n = 348 for both violins

violin plot)	After-cue I vs After-cue D: p = 1, n = 292 for both violins<br><br>Units for both n are changes in activity variation across all cells and all four matched distances.
S19C (track 1, line plots)	Bonferroni-Holm corrected two-way ANOVA:<br><br>Before-cue curves: 91 cells x 4 distances<br>After-cue curves: 77 cells x 4 distances<br><br>Before-cue Day 1 vs Day 7: p = 0.68244, F = 0.16798<br>After-cue Day 1 vs Day 7: p = 0.78146, F = 0.74149
S19C (track 2, line plots)	Bonferroni-Holm corrected two-way ANOVA:<br><br>Before-cue curves: 87 cells x 4 distances<br>After-cue curves: 73 cells x 4 distances<br><br>Before-cue Day 1 vs Day 7: p = 0.91209, F = 0.012228<br>After-cue Day 1 vs Day 7: p = 1, F = 0.1635
S19C (track 1, violin plot)	Bonferroni-Holm Corrected two-tailed paired t-tests.<br><br>Before-cue Day 1 vs Before-cue Day 7: p = 0.74735, n = 364 for both violins<br>After-cue Day 1 vs After-cue Day 7: p = 0.90033, n = 308 for both violins<br><br>Units for both n are activity variation differences across all cells and all four matched distances.
S19C (track 2, violin plot)	Bonferroni-Holm Corrected two-tailed paired t-tests.<br><br>Before-cue Day 1 vs Before-cue Day 7: p = 0.94809, n = 348 for both violins<br>After-cue Day 1 vs After-cue Day 7: p = 1, n = 292 for both violins<br><br>Units for both n are activity variation differences across all cells and all four matched distances.

**Figure S20**	
S20A (left)	Track 1: Two-way ANOVA: Bonferroni-Holm Corrected<br>All curves: 188 cell groups x 4 distances<br><br>I Day 1 vs I Day 7: p = 5.0071e-05, F = 18.215<br>D Day 1 vs D Day 7: p = 0.00010784, F = 15.32<br>Day 1 I vs D: p = 8.2472e-19, F = 90.088<br>Day 7 I vs D: p = 4.2625e-31, F = 166.07

	Track 2: Two-way ANOVA: Bonferroni-Holm Corrected<br>All curves: 243 cell groups x 4 distances<br><br>I Day 1 vs I Day 7: p = 0.022059, F = 5.275<br>D Day 1 vs D Day 7: p = 0.036691, F = 5.6008<br>Day 1 I vs D: p = 1.8537e-58, F = 348.15<br>Day 7 I vs D: p = 3.1449e-64, F = 396.03
S20A (right)	Track 1: Two-way ANOVA<br>Day: p = 2.7097e-18, F = 80.134<br>Landmark Pair Type: p = 2.1232e-81, F = 470.63<br>Day x Landmark Pair Type: p = 0.16057, F = 1.9727<br><br>Two-tailed paired t-tests: Bonferroni-Holm Corrected<br>Each violin contains n = 752 rank correlations across all cell groups and all four matched distances.<br><br>I Day 1 vs I Day 7: p = 2.4165e-08<br>D Track 1 vs D Day 7: p = 1.2261e-10<br>Day 1 I vs D: p = 5.3845e-51<br>Day 7 I vs D: p = 1.141e-47<br><br>Track 2: Two-way ANOVA<br>Day: p = 1.6223e-07, F = 27.844<br>Landmark pair type: p = 1.9623e-214, F = 1685.6<br>Day x Landmark pair type: p = 0.24862, F = 1.3326<br><br>Two-tailed paired t-tests: Bonferroni-Holm Corrected<br>Each violin contains n = 972 rank correlations across all cell groups and all four matched distances.<br><br>I Day 1 vs I Day 7: p = 0.00051921<br>D Track 1 vs D Day 7: p = 2.3988e-05<br>Day 1 I vs D: p = 1.5272e-122<br>Day 7 I vs D: p = 6.1016e-197
S20B (left)	Track 1: Two-way ANOVA<br>Both curves: 188 cell groups x 4 distances<br>p = 0.26395, F = 1.2517<br><br>Track 2: Two-way ANOVA<br>Both curves: 243 cell groups x 4 distances<br>p = 0.44015, F = 0.59687

S20B (right)	Two-tailed paired t-test:<br>Track 1: p = 0.16057, n = 752 for both violins<br>Track 2: p = 0.24862, n = 972 for both violins<br><br>Units for both n are changes in rank correlation across all cell groups and all four matched distances.
S20C (left)	Track 1: Two-way ANOVA<br>Both curves: 188 cell groups x 4 distances<br>Day 1 vs Day 7: p = 0.17234, F = 1.8696<br><br>Track 2: Two-way ANOVA:<br>Both curves: 243 cell groups x 4 distances<br>Day 1 vs Day 7: p = 0.479989, F = 0.49966
S20C (right)	Two-tailed paired t-tests<br>Track 1: p = 0.16057, n = 752 for both violins<br>Track 2: p = 0.24862, n = 972 for both violins<br><br>Units for both n are rank correlation differences across all cell groups and all four matched distances.

**Figure S21**	
S21A (track 1, top)	Bonferroni-Holm corrected two-way ANOVA:<br><br>Before-cue curves: 80 cell groups x 4 distances<br>After-cue curves: 72 cell groups x 4 distances<br><br>Before-cue Identical Day 1 vs 7: p = 0.68997, F = 1.4521<br>Before-cue Disparate Day 1 vs 7: p = 0.43111, F = 2.6161<br>After-cue Identical Day 1 vs 7: p = 0.81269, F = 0.056358<br>After-cue Disparate Day 1 vs 7: p = 0.93715, F = 0.52817
S21A (track 2, top)	Bonferroni-Holm corrected two-way ANOVA:<br><br>Before-cue curves: 80 cell groups x 4 distances<br>After-cue curves: 55 cell groups x 4 distances<br><br>Before-cue Identical Day 1 vs 7: p = 0.048919, F = 5.1602<br>Before-cue Disparate Day 1 vs 7: p = 0.17696, F = 1.8393<br>After-cue Identical Day 1 vs 7: p = 0.0041463, F = 10.781<br>After-cue Disparate Day 1 vs 7: p = 0.00071856, F = 15.059

S21A (track 1, bottom)	Bonferroni-Holm corrected two-tailed paired t-tests:<br><br>Before-cue Identical Day 1 vs 7: p = 0.36392, n = 320 for both violins<br>Before-cue Disparate Day 1 vs 7: p = 0.044518, n = 320 for both violins<br>After-cue Identical Day 1 vs 7: p = 0.75757, n = 288 for both violins<br>After-cue Disparate Day 1 vs 7: p = 0.6468, n = 288 for both violins<br><br>Units for all n are rank correlations across all cell groups and all four matched distances.
S21A (track 2, bottom)	Bonferroni-Holm corrected two-tailed paired t-tests:<br><br>Before-cue Identical Day 1 vs 7: p = 0.020016, n = 320 for both violins<br>Before-cue Disparate Day 1 vs 7: p = 0.017166, n = 320 for both violins<br>After-cue Identical Day 1 vs 7: p = 4.8656e-06, n = 220 for both violins<br>After-cue Disparate Day 1 vs 7: p = 1.2582e-07, n = 220 for both violins<br><br>Units for all n are rank correlations across all cell groups and all four matched distances.
S21B (track 1, line plots)	Bonferroni-Holm corrected two-way ANOVA:<br><br>Before-cue curves: 80 cell groups x 4 distances<br>After-cue curves: 72 cell groups x 4 distances<br><br>Before-cue Identical vs Disparate: p = 0.96625, F = 0.49413<br>After-cue Identical vs Disparate: p = 0.66817, F = 0.18452
S21B (track 2, line plots)	Bonferroni-Holm corrected two-way ANOVA:<br><br>Before-cue curves: 80 cell groups x 4 distances<br>After-cue curves: 55 cell groups x 4 distances<br><br>Before-cue Identical vs Disparate: p = 0.95702, F = 0.0029134<br>After-cue Identical vs Disparate: p = 0.76447, F = 0.38223
S21B (track 1, violin plot)	Bonferroni-Holm Corrected two-tailed paired t-tests:<br><br>Before-cue I vs Before-cue D: p = 0.61066, n = 320 for both violins<br>After-cue I vs After-cue D: p = 0.5951, n = 288 for both violins<br><br>Units for both n are changes in rank correlation across all cell groups and all four matched distances.
S21B (track 2,	Bonferroni-Holm Corrected two-tailed t-tests:<br><br>Before-cue I vs Before-cue D: p = 0.94479, n = 320 for both violins, paired

violin plot)	After-cue I vs After-cue D: p = 0.32708, n = 220 for both violins, paired<br><br>Units for both n are changes in rank correlation across all cell groups and all four matched distances.
S21C (track 1, line plots)	Bonferroni-Holm corrected two-way ANOVA:<br><br>Before-cue curves: 80 cell groups x 4 distances<br>After-cue curves: 72 cell groups x 4 distances<br><br>Before-cue Day 1 vs Day 7: p = 0.82041, F = 0.68183<br>After-cue Day 1 vs Day 7: p = 0.61146, F = 0.2592
S21C (track 2, line plots)	Bonferroni-Holm corrected two-way ANOVA:<br><br>Before-cue curves: 80 cell groups x 4 distances<br>After-cue curves: 55 cell groups x 4 distances<br><br>Before-cue Day 1 vs Day 7: p = 0.96511, F = 0.0019195<br>After-cue Day 1 vs Day 7: p = 0.41789, F = 1.5978
S21C (track 1, violin plot)	Bonferroni-Holm Corrected two-tailed t-tests:<br><br>Before-cue Day 1 vs Before-cue Day 7: p = 0.61066, n = 320 for both violins<br>After-cue Day 1 vs After-cue Day 7: p = 0.5951, n = 288 for both violins<br><br>Units for both n are rank correlation differences across all cell groups and all four matched distances.
S21C (track 2, violin plot)	Bonferroni-Holm Corrected two-tailed t-tests:<br><br>Before-cue Day 1 vs Before-cue Day 7: p = 0.94479, n = 320 for both violins<br>After-cue Day 1 vs After-cue Day 7: p = 0.32708, n = 220 for both violins<br><br>Units for both n are rank correlation differences across all cell groups and all four matched distances.

**Figure S22**	
S22A	Two-tailed paired t-tests:<br>Track 1: p = 0.95677, n = 217 cells<br>Track 2: p = 0.48646, n = 256 cells
S22B	Two-tailed paired t-tests:<br>Track 1: p = 0.13995, n = 188 cell groups<br>Track 2: p = 0.39883, n = 243 cell groups

S22C	Each dot represents the p-value resulting from a two-tailed paired t-test comparing the rank correlation difference per cell group on day 1 to that of day 7 for both track 1 and track 2.

**Figure S23**	
S23B (integrated activity variation)	Line Plot:<br>Two-way ANOVA: Bonferroni-Holm Corrected<br><br>All curves: 217 cell groups x 4 distances<br><br>I Day 1 vs I Day 7: p = 0.00956625, F = 8.7999<br>D Day 1 vs D Day 7: p = 0.0034422, F = 11.267<br>Day 1 I vs D: p = 0.0250484, F = 5.0564<br>Day 7 I vs D: p = 0.011437, F = 7.7186<br><br><br>Violin Plot:<br>Two-way ANOVA<br>Day: p = 1.1144e-13, F = 57.066<br>Landmark Pair Type: p = 6.6752e-07, F = 25.094<br>Day x Landmark Pair Type: p = 0.5517, F = 0.35457<br><br>Two-tailed paired t-tests: Bonferroni-Holm Corrected<br><br>Each violin has n = 868 integrated activity variations across all cells and all four matched distances.<br><br>I Day 1 vs I Day 7: p = 8.07545e-08<br>D Day 1 vs D Day 7: p = 8.71284e-09<br>Day 1 I vs D: p = 0.000971204<br>Day 7 I vs D: p = 0.000184809
S23B (integrated activity variation difference)	Line Plot:<br>Two-way ANOVA:<br>Both curves: 217 cells x 4 distances<br>Day 1 vs Day 7: p = 0.61027, F = 0.26019<br><br>Violin Plot: Each violin contains n = 868 integrated activity variation differences across all cells and all four matched distances.<br>Two-tailed paired t-test comparing day 1 to day 7: p = 0.5517<br><br>Bonferroni-Holm corrected two-tailed paired t-test comparing both samples to 0:<br>p_{Day1-0} = 0.000971204, p_{Day7-0} = 0.000184809

S23C (integrated activity variation)	Line Plot:<br>Two-way ANOVA: Bonferroni-Holm Corrected<br><br>All curves: 256 cell groups x 4 distances<br><br>I Day 1 vs I Day 7: p = 0.000717835, F = 12.912<br>D Day 1 vs D Day 7: p = 0.00508463, F = 7.9194<br>Day 1 I vs D: p = 7.33884e-05, F = 18.709<br>Day 7 I vs D: p = 0.000360621, F = 15.031<br><br>Violin Plot:<br>Two-way ANOVA<br>Day: p = 3.0164e-14, F = 59.463<br>Landmark Pair Type: p = 4.0543e-19, F = 83.18<br>Day x Landmark Pair Type: p = 0.43399, F = 0.61262<br><br>Two-tailed paired t-tests: Bonferroni-Holm Corrected<br><br>Each violin has n = 1024 integrated activity variations across all cells and all four matched distances.<br><br>I Day 1 vs I Day 7: p = 2.23852e-10<br>D Day 1 vs D Day 7: p = 4.20952e-08<br>Day 1 I vs D: p = 5.93184e-14<br>Day 7 I vs D: p = 1.13449e-09
S23C (integrated activity variation difference)	Line Plot:<br>Two-way ANOVA:<br>Both curves: 256 cells x 4 distances<br>Day 1 vs Day 7: p = 0.60059, F = 0.27446<br><br>Violin Plot: Each violin contains n = 1024 integrated activity variation differences across all cells and all four matched distances.<br><br>Two-tailed paired t-test comparing day 1 to day 7: p = 0.433991<br><br>Bonferroni-Holm corrected two-tailed paired t-test comparing both samples to 0: p_{Day1-0} = 2.96592e-14, p_{Day7-0} = 5.67245e-10
S23D (integrated activity variation)	Line Plot:<br>Two-way ANOVA: Bonferroni-Holm Corrected<br><br>All curves: 132 cell groups x 4 distances<br><br>I Day 1 vs I Day 7: p = 0.920099, F = 0.010082

	D Day 1 vs D Day 7: p = 1, F = 0.32136<br>Day 1 I vs D: p = 0.780883, F = 1.6865<br>Day 7 I vs D: p = 1, F = 0.30205<br><br>Violin Plot:<br>Two-way ANOVA<br>Day: p = 0.8919, F = 0.018488<br>Landmark Pair Type: p = 0.078222, F = 3.1139<br>Track x Landmark Pair Type: p = 0.53533, F = 0.38478<br><br>Two-tailed paired t-tests: Bonferroni-Holm Corrected<br><br>Each violin has n = 528 integrated activity variations across all cells and all four matched distances.<br><br>I Day 1 vs I Day 7: p = 1<br>D Day 1 vs D Day 7: p = 0.676959<br>Day 1 I vs D: p = 0.314925<br>Day 7 I vs D: p = 1
S23D (integrated activity variation difference)	Line Plot:<br>Two-way ANOVA:<br>Both curves: 132 cells x 4 distances<br>Day 1 vs Day 7: p = 0.51414, F = 0.42684<br><br>Violin Plot: Each violin contains n = 528 integrated activity variation differences across all cells and all four matched distances.<br><br>Two-tailed paired t-test comparing day 1 to day 7: p = 0.535331<br><br>Bonferroni-Holm corrected two-tailed paired t-test comparing both samples to 0: p_{Day1-0} = 0.157462, p_{Day7-0} = 0.398145
S23E (integrated activity variation)	Line Plot:<br>Two-way ANOVA: Bonferroni-Holm Corrected<br><br>All curves: 118 cell groups x 4 distances<br><br>I Day 1 vs I Day 7: p = 0.703346, F = 1.4211<br>D Day 1 vs D Day 7: p = 0.399909, F = 0.71122<br>Day 1 I vs D: p = 0.653367, F = 1.9553<br>Day 7 I vs D: p = 0.607882, F = 1.0616<br><br>Violin Plot:

	Two-way ANOVA<br>Day: p = 0.017629, F = 5.6732<br>Landmark Pair Type: p = 0.066821, F = 3.3753<br>Day x Landmark Pair Type: p = 0.77264, F = 0.083573<br><br>Two-tailed paired t-tests: Bonferroni-Holm Corrected<br><br>Each violin has n = 472 integrated activity variations across all cells and all four matched distances.<br><br>I Day 1 vs I Day 7: p = 0.284812<br>D Day 1 vs D Day 7: p = 0.308253<br>Day 1 I vs D: p = 0.304716<br>Day 7 I vs D: p = 0.262601
S23E (integrated activity variation difference)	Line Plot:<br>Two-way ANOVA:<br>Both curves: 118 cells x 4 distances<br>Day 1 vs Day 7: p = 0.69271, F = 0.15656<br><br>Violin Plot: Each violin contains n = 472 integrated activity variation differences across all cells and all four matched distances.<br><br>Two-tailed paired t-test comparing day 1 to day 7: p = 0.772642<br><br>Bonferroni-Holm corrected two-tailed paired t-test comparing both samples to 0: p_{Day1-0} = 0.203144, p_{Day7-0} = 0.262601

**Figure S24**	
S24A	Track 1:<br>Bonferroni-Holm corrected two-tailed nonpaired t-test comparing cue and putative grid cell integrated activity variation:<br><br>Identical Day 1: p = 3.38204e-08, n_{cue} = 868, n_{grid} = 528<br>Disparate Day 1: p = 4.2159e-05, n_{cue} = 868, n_{grid} = 528<br>Identical Day 7: p = 0.299738, n_{cue} = 868, n_{grid} = 528<br>Disparate Day 7: p = 0.330219, n_{cue} = 868, n_{grid} = 528<br><br><br>Track 2:<br>Bonferroni-Holm corrected two-tailed nonpaired t-test comparing cue and putative grid cell integrated activity variation:

	Identical Day 1: p = 7.61253e-08, n_{cue} = 1024, n_{grid} = 472<br>Disparate Day 1: p = 0.0774822, n_{cue} = 1024, n_{grid} = 472<br>Identical Day 7: p = 0.0101405, n_{cue} = 1024, n_{grid} = 472<br>Disparate Day 7: p = 0.765074, n_{cue} = 1024, n_{grid} = 472<br><br>Units for all n are integrated activity variations across all cells and all four matched distances.
S24B	Track 1:<br>Bonferroni-Holm corrected two-tailed non-paired t-tests:<br>Day 1 Cue vs Grid: p = 0.562364, n_{cue} = 868, n_{grid} = 528<br>Day 7 Cue vs Grid: p = 0.204154, n_{cue} = 868, n_{grid} = 528<br><br>Bonferroni-Holm corrected two-tailed paired t-tests comparing each sample with 0:<br>Day 1 Cue: p = 0.00291361, n = 868<br>Day 1 Grid: p = 0.157462, n = 528<br>Day 7 Cue: p = 0.000369618, n = 868<br>Day Grid: p = 0.398145, n = 528<br><br>Track 2:<br>Bonferroni-Holm corrected two-tailed non-paired t-tests:<br>Day 1 Cue vs Grid: p = 0.00999223, n_{cue} = 1024, n_{grid} = 472<br>Day 7 Cue vs Grid: p = 0.0141315, n_{cue} = 1024, n_{grid} = 472<br><br>Bonferroni-Holm corrected two-tailed paired t-tests comparing each sample with 0:<br>Day 1 Cue: p = 5.93184e-14, n = 1024<br>Day 1 Grid: p = 0.203144, n = 472<br>Day 7 Cue: p = 1.70173e-09, n = 1024<br>Day Grid: p = 0.262601, n = 472<br><br>Units for all n are integrated activity variation differences across all cells and all four matched distances.
S24C	Track 1:<br><br>Two-tailed paired t-test comparing integrated activity variation difference with 0 for both day 1 and day 7 condition, independently Bonferroni-Holm corrected for each activity range and functional class (i.e. as in Fig. 7C (right) and Fig. 7E (right)).<br><br>Cue:

Activity range (cm)	p-value, day 1	p-value, day 7
5	0.0092	0.0829
10	0.0017	0.0025
15	8.4482e-04	4.9930e-04
20	4.1396e-04	3.3167e-04
25	9.7120e-04	1.8481e-04
30	0.0015	2.4905e-04
35	0.0025	1.8006e-04
40	0.0038	2.3188e-04
45	0.0069	6.2461e-04
50	0.0127	0.0014

Putative Grid:

Activity range (cm)	p-value, day 1	p-value, day 7
5	0.0575	0.5990
10	0.1789	0.3504
15	0.2504	0.5587
20	0.1694	0.4834
25	0.1575	0.3981
30	0.2205	0.3243
35	0.3525	0.2021
40	0.2883	0.3153
45	0.3909	0.3864
50	0.6378	0.4237

Track 2:

Two-tailed paired t-test comparing integrated activity variation difference with 0 for both day 1 and day 7 condition, independently Bonferroni-Holm corrected for each activity range and functional class (i.e. as in Fig. 7C (right) and Fig. 7E (right)).

Cue:

Activity range (cm)	p-value, day 1	p-value, day 7
5	2.4183e-11	3.2009e-07
10	1.9071e-11	1.7894e-08
15	3.0256e-11	2.1211e-09
20	3.2833e-13	7.8230e-10
25	2.9659e-14	5.6724e-10
30	5.7784e-14	3.2286e-10
35	7.8683e-14	1.5742e-11

40	4.9286e-15	1.3034e-11
45	1.0239e-15	6.3900e-12
50	1.0127e-15	4.1812e-13

Putative Grid:

Activity range (cm)	p-value, day 1	p-value, day 7
5	0.3601	0.2597
10	0.1861	0.1081
15	0.1177	0.1758
20	0.1434	0.4049
25	0.2031	0.2626
30	0.4476	0.2801
35	0.6468	0.3385
40	0.2932	0.5388
45	0.3779	0.2781
50	0.2301	0.2602

**Figure S25**	
S25B	Line plot:<br>Two-way ANOVA: Both curves have 508 cells x 4 matched distances<br>p = 7.2746e-17, F = 72.179<br><br>Violin plot:<br>Two-tailed paired t-test: p = 8.33614e-40, n = 2032 integrated activity variations across all cells and all four matched distances for both violins
S25C	Two-tailed paired t-test with 0: p = 8.33614e-40, n = 2032 integrated activity variation differences s across all cells and all four matched distances for both violins
S25D	Line plot:<br>Two-way ANOVA: Both curves have 668 cells x 4 matched distances<br>p = 2.95443e-05, F = 17.571<br><br>Violin plot:<br>Two-tailed paired t-test: p = 4.67585e-11, n = 2672 integrated activity variations across all cells and all four matched distances for both violins
S25E	Two-tailed paired t-test with 0: p = 4.67585e-11, n = 2672 integrated activity variation differences s across all cells and all four matched distances for both violins
S25F	Two-way ANOVA with mixed-effects model and Sattherthwaite-adjusted denominator degrees of freedom (df)<br>Functional Class: p = 1.838e-06, F = 22.816<br>Landmark Pair Type: p = 2.6237e-46, F = 208.71

	Functional Class x Landmark Pair Type: $p = 6.8032e-09$, $F = 33.716$<br><br>Bonferroni-Holm Corrected two-tailed t-tests. Comparisons of the same cells (cue or putative grid cells) are paired, while comparisons of different cells are nonpaired.<br><br>Identical Cue vs Grid: $p = 5.52207e-13$, $n_{cue} = 2032$, $n_{grid} = 2672$<br>Disparate Cue vs Grid: $p = 0.836079$, $n_{cue} = 2032$, $n_{grid} = 2672$<br>Cue Identical vs Disparate: $p = 3.33446e-39$, $n_{cue} = 2032$<br>Grid Identical vs Disparate: $p = 9.3517e-11$, $n_{grid} = 2672$<br><br>Units for all n are integrated activity variations across all cells and all four matched distances.
S25G	Two-tailed nonpaired t-test: $p = 9.23962e-09$, $n_{cue} = 2032$, $n_{grid} = 2672$<br><br>Units for both n are integrated activity variation differences across all cells and all four matched distances.
S25H	Two-tailed paired t-test comparing integrated activity variation difference with 0 for both cue and putative grid cells.

Activity range (cm)	p-value, cue	p-value, grid
5	7.4913e-28	4.1815e-12
10	6.5428e-30	4.3986e-11
15	4.7114e-35	3.3912e-12
20	5.6827e-40	2.2373e-12
25	8.3361e-40	4.6759e-11
30	6.9960e-39	1.0081e-09
35	1.3272e-38	1.9482e-09
40	1.3088e-35	5.3765e-08
45	8.1620e-32	1.0729e-06
50	2.0554e-29	4.2066e-06
