# Supplementary material for: The entorhinal spatial map integrates visual identity information of landmarks
Source: Nat Commun. 2026 May 7;17:6164. doi: 10.1038/s41467-026-72453-1 (PMC13365227; doi:10.1038/s41467-026-72453-1)
Supplement: Supplementary file 2 — Reporting Summary [file 41467_2026_72453_MOESM2_ESM.pdf]

nature portfolio

Corresponding author(s): Yi Gu

Last updated by author(s): 03/20/2026

Reporting Summary

Nature Portfolio wishes to improve the reproducibility of the work that we publish. This form provides structure for consistency and transparency in reporting. For further information on Nature Portfolio policies, see our Editorial Policies and the Editorial Policy Checklist.

Statistics

For all statistical analyses, confirm that the following items are present in the figure legend, table legend, main text, or Methods section.

n/a	Confirmed	
☐	☒	The exact sample size (*n*) for each experimental group/condition, given as a discrete number and unit of measurement
☐	☒	A statement on whether measurements were taken from distinct samples or whether the same sample was measured repeatedly
☐	☒	The statistical test(s) used AND whether they are one- or two-sided<br>*Only common tests should be described solely by name; describe more complex techniques in the Methods section.*
☐	☒	A description of all covariates tested
☐	☒	A description of any assumptions or corrections, such as tests of normality and adjustment for multiple comparisons
☐	☒	A full description of the statistical parameters including central tendency (e.g. means) or other basic estimates (e.g. regression coefficient) AND variation (e.g. standard deviation) or associated estimates of uncertainty (e.g. confidence intervals)
☐	☒	For null hypothesis testing, the test statistic (e.g. *F*, *t*, *r*) with confidence intervals, effect sizes, degrees of freedom and *P* value noted<br>*Give P values as exact values whenever suitable.*
☒	☐	For Bayesian analysis, information on the choice of priors and Markov chain Monte Carlo settings
☒	☐	For hierarchical and complex designs, identification of the appropriate level for tests and full reporting of outcomes
☐	☒	Estimates of effect sizes (e.g. Cohen's *d*, Pearson's *r*), indicating how they were calculated

Our web collection on statistics for biologists contains articles on many of the points above.

Software and code

Policy information about availability of computer code

Data collection	Two-photon imaging was performed using Prairie View (Bruker, version 5.7). Mouse behavior was recorded using ViRMEn (Princeton, version 2016-02-12) and Prairie View.
Data analysis	Image processing was performed using previously published MATLAB (Mathworks, version R2015aSP1) codes and Suite2p (University College London, v0.11.1) as cited in the methods section. Data analysis was performed using Matlab (Mathworks, versions R2015aSP1 for ViRMEn and R2023-2024 for analyses). Custom Matlab scripts used for data analysis are available on GitHub: https://github.com/GuLab-NIH/Wang-and-Shahid-2026

For manuscripts utilizing custom algorithms or software that are central to the research but not yet described in published literature, software must be made available to editors and reviewers. We strongly encourage code deposition in a community repository (e.g. GitHub). See the Nature Portfolio guidelines for submitting code & software for further information.

Data

Policy information about availability of data

All manuscripts must include a data availability statement. This statement should provide the following information, where applicable:

- Accession codes, unique identifiers, or web links for publicly available datasets
- A description of any restrictions on data availability
- For clinical datasets or third party data, please ensure that the statement adheres to our policy

Data generated in this study has been deposited to Zenodo [https://doi.org/10.5281/zenodo.19115394]. Processed data underlying all figures are available in the

Source Data file. Further information and requests for resources and reagents should be directed to the lead contact, Dr. Yi Gu (yi.gu@nih.gov).

Research involving human participants, their data, or biological material

Policy information about studies with human participants or human data. See also policy information about sex, gender (identity/presentation), and sexual orientation and race, ethnicity and racism.

Reporting on sex and gender	Human research participants were not used.
Reporting on race, ethnicity, or other socially relevant groupings	Human research participants were not used.
Population characteristics	Human research participants were not used.
Recruitment	Human research participants were not used.
Ethics oversight	Human research participants were not used.

Note that full information on the approval of the study protocol must also be provided in the manuscript.

Field-specific reporting

Please select the one below that is the best fit for your research. If you are not sure, read the appropriate sections before making your selection.

☒ Life sciences ☐ Behavioural & social sciences ☐ Ecological, evolutionary & environmental sciences

For a reference copy of the document with all sections, see nature.com/documents/nr-reporting-summary-flat.pdf

Life sciences study design

All studies must disclose on these points even when the disclosure is negative.

Sample size	No statistical methods were used to predetermine sample sizes. Sample sizes in terms of mice and neurons are similar to other contemporary studies in the field (For example, see Pettit et. al. Nature 2022, Kinkhabwala et. al. eLife 2020, Robinson et. al. Cell 2020, Gu et. al. Cell 2018, Danielson et. al. Neuron 2016).
Data exclusions	No data was excluded.
Replication	All experiments were replicated at least once, and similar results were obtained.
Randomization	Animals were not separated into different experimental groups.
Blinding	Animals were not separated into groups, but comparisons of various groups within the manuscript were done after data collection when group allocation was already set. The complexity of neural data as well as the fact that much analysis was conducted via automated code post-data collection makes the experimenters blinded to group allocation.

Reporting for specific materials, systems and methods

We require information from authors about some types of materials, experimental systems and methods used in many studies. Here, indicate whether each material, system or method listed is relevant to your study. If you are not sure if a list item applies to your research, read the appropriate section before selecting a response.

Materials & experimental systems

n/a	Involved in the study	
☒	☐	Antibodies
☒	☐	Eukaryotic cell lines
☒	☐	Palaeontology and archaeology
☐	☒	Animals and other organisms
☒	☐	Clinical data
☒	☐	Dual use research of concern
☒	☐	Plants

Methods

n/a	Involved in the study	
☒	☐	ChIP-seq
☒	☐	Flow cytometry
☒	☐	MRI-based neuroimaging

Animals and other research organisms

Policy information about studies involving animals; ARRIVE guidelines recommended for reporting animal research, and Sex and Gender in Research

Laboratory animals	GP5.3 (C57BL/6J-Tg(Thy1-GCaMP6f)GP5.3Dkim/J, JAX stock #028280) mice : 4-7 months old. Mice were maintained on a reverse 12-hr on/off light schedule with all experiments being performed in the light off period. Animals were housed at a temperature of 70–74°F and 40–65% humidity.
Wild animals	No wild animals were used.
Reporting on sex	Both male and female mice were used for all experiments based on availability at the time of experiment. GP5.3 mice: 3 males and 2 females (4-5 months old) for most all analyses, which is roughly sex-balanced. Track 4 mice (Fig. S4) included the original 5 mice from the main experiment as well as an additional 5 male mice and 1 female mouse aged 6-7 months at the time when imaging began. Track 5 data (Fig. S4) are made up entirely of the additional 5 male and 1 female mice. Analyses were not separated by sex, as previous work found navigation performance to be similar across sex (Malone et. al. Nature Comm 2024).
Field-collected samples	Study did not involve field-collected samples.
Ethics oversight	All animal procedures were performed in accordance with animal protocol 1524 approved by the Institutional Animal Care and Use Committee (IACUC) at NIH/NINDS.

Note that full information on the approval of the study protocol must also be provided in the manuscript.

Plants

Seed stocks	Plants were not used.
Novel plant genotypes	Plants were not used.
Authentication	Plants were not used.
