# Supplementary material for: The entorhinal spatial map integrates visual identity information of landmarks
Source: Nat Commun. 2026 May 7;17:6164. doi: 10.1038/s41467-026-72453-1 (PMC13365227; doi:10.1038/s41467-026-72453-1)
Supplement: Supplementary file 3 — Transparent Peer Review file [file 41467_2026_72453_MOESM3_ESM.pdf]

nature portfolio

Peer Review File

The entorhinal spatial map integrates visual identity information of landmarks

Corresponding Author: Dr Yi Gu

This file contains all reviewer reports in order by version, followed by all author rebuttals in order by version.

Version 0:

Reviewer comments:

Reviewer #1

(Remarks to the Author)
The study by Wang et al. investigates the medial entorhinal cortex's (MEC) role in encoding both spatial and non-spatial information, specifically the visual identity of landmarks, during navigation. Using two-photon calcium imaging in mice navigating virtual environments, the researchers discovered that MEC, traditionally associated with spatial information processing, also encodes landmark identity through cue cells—neurons that respond to individual landmarks. These cue cells exhibited distinct activity patterns for visually disparate landmarks compared to identical ones, indicating a role in landmark identity encoding. The study further revealed that this encoding is modulated by the spatial relationship between cue cell activity and landmark location and is subject to change across different environments but remains consistent within the same environment despite increased experience. In contrast, grid cells, known for their role in spatial navigation, were found to weakly encode landmark identity but robustly encode landmark location. The authors consider these results to be evidence for the idea that MEC integrates spatial and non-spatial information.

Although landmark coding is an important topic in spatial navigation, I find the advances in this work are incremental. More importantly, many claims are either too strong or inaccurate. Here are my major comments:
(1) The authors claimed that previous studies suggest landmark identity is only coded in LEC and hippocampus in the discussion, and frame this integration of spatial and non-spatial information as one of their major discussion points. I have an issue with this interpretation. Landmarks, as the name suggests, carry spatial information, and even identity information can be useful in inferring spatial relationships. The authors seem to equate the landmark with objects placed inside the arena, and the latter has been used to discern spatial and non-spatial coding of MTL regions. However, as these objects do not provide enough polarizing information, they are not the same as landmarks.
(2) The novelty of the results on cue cells is limited. Previous studies have shown the coding of landmarks by MEC neurons (see Casali et al., 2018, Frontiers in Cellular Neuroscience; and Nguyen et al., 2024, Cell Report by the author's group). Here, the authors performed detailed quantification of the rate modulation, which is apparent in the above citations. This is not a conceptual advance.
(3) In the discussion (Line 569-573), the authors consider the differed activity level (rate remapping) by cue cells to be a novel finding. Quite the opposite, this is a very popular mechanism employed by the hippocampus and associated region, as seen in the numerous cases summarized in a previous review (O'Keefe and Krupic, 2021, Physiol Rev). Even MEC grid cells could use this rate remapping mechanism to code environmental features (Diehl et al., 2017, Neuron).

Reviewer #2

(Remarks to the Author)
Ever since discovery of grid cells in medial entorhinal cortex (MEC), they have been thought to play a key role in path integration. Path integration, however, is noisy on its own and prone to error accumulation over time. The errors must be corrected for the system to maintain an accurate spatial code. A critical complementary mechanism to this purpose is error correction, which relies on positional feedback from landmarks. Although our knowledge on grid cells has advanced, the neural mechanisms underlying spatial error correction are still largely unknown. This paper studies a group of MEC cells, termed "cue cells", that may play a role in error correction. Specifically, the paper addresses an intriguing question of whether "cue cells" encode landmark identity. Although the findings are interesting and the experimental design is clever, I have several major concerns regarding the methodology and presentation of results. I believe these concerns must be

addressed before the paper can be considered for publication.
Major comments
• A potential major confound arises from the unequal distribution of landmarks. Across all tracks, one type of landmark (e.g., rectangles) is more frequent than others (e.g., cones), leading to disparate comparisons that are more likely to involve the less frequent landmark type, while identical comparisons are dominated by the more frequent landmark type. For instance, in track 3, the imbalance is even more pronounced, with four dotted rectangles compared to just one instance of each of the other two landmark types. Interestingly, the largest activity variation difference across tracks is observed on track 3, raising concerns that this effect might be driven by the unequal representation of landmarks rather than true landmark identity encoding. If the less frequent landmarks intrinsically exhibit greater variability in neural activity (e.g., due to differences in salience), this imbalance could artificially inflate activity variation in disparate comparisons. To address this, I recommend analyzing the different landmark types separately and/or subsampling to balance the comparisons. Additionally, testing whether the less frequent landmark types exhibit higher intrinsic variability could provide further clarity.
On a related note, it would strengthen the results if the authors accounted for the location of landmarks relative to the reward. Given the animal's stereotyped behavior, presumably becoming more attentive as it approaches the reward, and prior literature showing the influence of attention on spatial coding, a possible confound might arise from imbalances in the proximity of landmarks to the reward between disparate and identical comparisons. If landmarks in disparate comparisons tend to be closer to the reward on average, this could bias the results by amplifying neural variability in those conditions. Including a control analysis to address this potential confound would further solidify the findings.
• Fig. 5 and the corresponding Results section feel disconnected from the rest of the paper and appear to serve more as a supplement to earlier figures (e.g., Figs. 2 and 4) rather than adding new insights. Specifically, Fig. 5D-K (which occupies the most space) largely replicates analyses from Figs. 2 and 4, focusing on common cells across tracks 1 and 2. This analysis could instead be mentioned in the Results sections for Figs. 2 and 4, with a reference to a supplementary figure. Additionally, it is unclear why this analysis only includes tracks 1 and 2 while excluding track 3, and this should be clarified. The other panels of Fig. 5 (A-C) also seem less critical and could be presented as supplementary analyses linked to the Results section for Fig. 2 or Fig 6.
Overall, I recommend moving Fig. 5 to the supplementary figures and referring to it in the relevant Results sections to streamline the main text and maintain focus on the primary findings.
• The difference in activity variations between identical and disparate landmarks in Fig. 6D appears considerably smaller than in Fig. 2, and it must be clarified why this is the case. It seems that the difference may reduce over days, suggesting that activity variations for identical and disparate landmarks might converge with experience. If this is the case, it would challenge the authors' claim that cue cells encode landmark identity, or at least suggest that this encoding diminishes or becomes less distinct in familiar environments. Similarly, Fig. 6E shows a potential trend of decreasing rank correlations over time. Have the authors analyzed whether the activity variation difference settles within 7 days or whether the trend suggests further convergence with more days of experience? Additional analysis is needed to address these questions and clarify the implications for landmark identity encoding over time.
Moreover, it is unclear why the longitudinal analysis in Fig. 6 is presented only for track 1. The result for track 2 seems to be presented in Fig 7, which is strange. Results of the same analysis should be provided for track 2 within Fig 6 to ensure the robustness of the findings across tracks and for a less convoluted description of results.
• The authors should report the percentage of overlap between "putative" grid cells and cue cells and clarify why cue cells were removed from the grid cells not the other way around. This decision appears arbitrary unless a strong and convincing rationale is provided. It is critical to demonstrate the robustness of the findings by showing that the results in Figs. 1 to 6 still hold when conjunctive grid + cue cells are removed from the cue cell population. Otherwise, it raises the concern that the reported results might not generalize to "pure" cue cells.
For the sake of transparency, the authors should also explicitly indicate that the identified grid cells are "putative," as it is possible that they may not be true grid cells.
There is a lot of redundancy between Fig 7D-E and Fig 6. Authors may just refer to Fig 6 and remove these repeated panels in Fig 7.
• I found the decoding analysis (lines 502–514) challenging to follow, and it would benefit from clearer explanation. In particular, it is unclear how the analysis distinguishes between landmark identity encoding and temporal instability (i.e., changes in activity patterns from one lap to another). If the decoding accuracy difference is primarily driven by consistency in activity patterns across laps rather than distinct representations of landmark identity, the results might reflect stability rather than true identity encoding. I suggest revising this section to improve clarity and providing more details on how the analysis accounts for potential confounds, and demonstrating whether the observed decoding accuracy difference is robust against temporal instability in the neural activity.

Other comments
• Number of animals and number of cells used for the analyses must be reported.
• What is the justification for use of 80th percentile as the cut-off for cue cells across multiple sessions as opposed to 95th or a larger percentile?
• What is 1-pvalue?
• Landmark template of track 1 in Fig 2C does not match to the template in Fig 1D.
• It is unclear whether the authors chose a single random subset of cells for a given N in analysis of Figure 3 or they subsample all possible subsets (or as many possible)? If a single random subset is used from each FOV, the authors must perform additional analyses to show that their results are not by chance and they hold true for other subsets to.
• Why is the second landmark used as a reference in the second and third rows of Fig 3E whereas the first landmark is used as the reference in the first row?
• Given that cue cells become active at all cues and given that the minimum inter-landmark distance is 100 cm in all three tracks, the spatial shifts of cue cells are expected to be within +- 100/2 centimeters. Fig 4B can be simply explained by this

expectation?

Reviewer #3

(Remarks to the Author)
The manuscript presents a compelling exploration of whether the medial entorhinal cortex (mEC) encodes landmark identity, providing a pathway in addition to the lateral entorhinal cortex for non-spatial information to reach the hippocampus. The experimental design and use of virtual environments are innovative and well-controlled for the research question. I also appreciate the effort to test landmark identity encoding across multiple contexts, as well as across days, adding depth to the investigation beyond what is currently known. Overall, the manuscript convincingly demonstrates that cue (or landmark) responsive cells in the mEC differentiate between landmarks in a manner that evolves over days and across environments.

However, I have major concerns with the interpretation of analyses and statistical methodology, as well as some conceptual oversights, as outlined below.

Major Comments:

1. My biggest issue with the current version of the manuscript is its narrative, partially in the introduction and definitely in the discussion. The authors describe what used to once be the prevailing view of entorhinal functional segregation – 'mEC provides spatial information, while lEC provides non-spatial information' as a core assumption that their manuscript refutes, but this view is no longer the basic assumption. There have been numerous studies describing abundant non-spatial information coding in the mEC (Hardcastle et al., 2017, for a review; Diehl et al., 2017; many others cited in this manuscript and previous work from Dr. Gu's lab). I suggest the authors rewrite the narrative to acknowledge that information in the EC is not as segregated as was previously hypothesized. What this manuscript provides, quite nicely, is a thorough description of the landmark identity coding abilities of mEC neurons. The authors should focus on this aspect rather than the broader non-spatial and spatial tuning.

2. My other conceptual issue with the manuscript is the claim that they have uncovered 'distinct circuit mechanisms supporting non-spatial and spatial encoding' in mEC. There is no data to truly suggest the existence of distinct mechanisms. I have several concerns with the analysis of grid cells (elaborated in a later point). But even barring those, effectively what the authors have done is subselect two groups of neurons – one, the 'cue cells,' which respond to landmarks, and the other, 'grid cells,' which fire in fixed spatial locations. Analyzing these cell types will obviously yield differential results on landmark responses and location responses respectively, and it is unclear how these differences reflect underlying circuit mechanisms versus a result of the subselection.

3. Statistical analyses throughout the manuscript lack details (e.g., F, Z statistics, corrections for multiple comparisons, number of samples). Also, the main statistics and type of test performed should be reported in the figure legends rather than a supplementary table (which also provides no details). In several plots, the choice of statistical tests is also questionable, e.g., plots like Figure 8B, E should use two-way ANOVAs with posthoc tests rather than multiple t-tests.

4. I appreciate the schematics provided by the authors to explain their analytical approaches. Despite this, the manuscript is quite confusing, and often, the main point of the figure becomes hidden or unclear. Part of the problem is that there are so many conditions and comparisons (disparate versus identical cues, types of tracks, day 1 vs day 7), it is hard for the reader. One suggestion would be to combine or condense results across tracks and perhaps show individual track results in the supplement. Only the figure comparing across tracks (Figure 5) can be retained. Reducing the overall number of plots in a figure would greatly help the readability of the manuscript.

5. Line 502: "However, since the above analyses were designed for landmark-specific activity of cue cells, they may not properly reveal landmark identity encoding by grid cells." This is a major issue of the grid cell analysis. Grid cells may or may not have a field near each landmark, so simply correlating activity around same and different landmarks is misleading. The authors should perform the analysis around defined grid fields, perhaps comparing fields near distinct landmarks to see if the rate in each node is differentially modulated as reported in previous studies.

Moreover, the main result in Figure 8 is the comparison between grid and cue cells (Fig. 8H). But the differences in decoding accuracy are quite minimal, and not surprising, especially because one would expect grid cells to very effectively differentiate between landmarks. Overall, I am unconvinced that the grid cell analysis adds to this manuscript, especially because the ability to decode location is obvious. Perhaps the authors should focus more on how cue identity and experience alter individual grid nodes, following up on past work in open fields (Diehl et al., 2017; Ismakov et al., 2017; Boccara et al., 2019).

Minor Comments:

1. Both the main and supplemental figure legends could benefit from clearer descriptions and explicit connections to the main text findings.

2. One thing unclear from the current analysis is, do the cue cells not reflect the landmark location at all? For example, in Figure 3B, right, the correlation of ~0.6 for identical landmarks is quite high, suggesting similar firing irrespective of location.

But, based on Fig. 8E, even identical landmarks can be correctly decoded 80% of the time. Some estimate of the floor or ceiling levels of these decoding accuracies and rank correlations using shuffles would help clarify these points.

3. Perhaps the population analysis in Figure 3 can be moved to be closer to Figure 8, as they are more similar conceptually.

4. The spatial shift of landmark cells, reflecting more prospective tuning, is extremely interesting. Do grid cell fields also demonstrate any shift in their fields over experience on the track?

5. I do not understand the results in Figure 2G. Cue cells were selected for their response across all landmarks, not a certain type of landmark. Why then, at the population level, is there a preferential response for landmark type 1? Surely that would average out across the population with distinct cells preferring different landmarks?

6. Why show the p-value in Supplementary Figure 2E, instead of showing the results themselves?

7. I am also confused by Figure 5D. "Across tracks 1 and 2, the cells changed their activity variations at identical landmarks (Fig. 5D, the two green groups), but not at disparate ones (Fig. 5D, the two magenta groups)." Why would this be the case?

8. Lines 437-439: "Thus, the encodings of landmark identity and location by cue cells are differentially regulated by experience, suggesting different circuit mechanisms underlying these encodings," seems out of context and an overclaim given the results.

Version 1:

Reviewer comments:

Reviewer #1

(Remarks to the Author)
I'm overall satisfied with the authors' response. However, I strongly suggest that the authors add additional discussion regarding the difference and relationship between the coding of landmark identity and the previously reported rate remapping in MEC.

Reviewer #3

(Remarks to the Author)
I am satisfied with the authors' thorough and careful rebuttal, which addresses the previous concerns in detail. The additional analyses, as well as the companion manuscript, greatly strengthen the study. I have no major concerns.

One minor point: the authors state that their new grid cell analyses show grid cells are "spatial and not landmark-driven." Strictly speaking, the analyses demonstrate that grid cells are not landmark-driven; the claim that they are "spatial" is inferred from their known properties, rather than directly shown in these experiments. This is not a major issue but worth noting for precision.

Overall, I find the revisions convincing and the manuscript much improved.

Version 2:

Reviewer comments:

Reviewer #1

(Remarks to the Author)
I thank the authors for the additional discussion. I have no further comments.

Open Access This Peer Review File is licensed under a Creative Commons Attribution 4.0 International License, which permits use, sharing, adaptation, distribution and reproduction in any medium or format, as long as you give appropriate credit to the original author(s) and the source, provide a link to the Creative Commons license, and indicate if changes were made.
In cases where reviewers are anonymous, credit should be given to 'Anonymous Referee' and the source.
The images or other third party material in this Peer Review File are included in the article's Creative Commons license, unless indicated otherwise in a credit line to the material. If material is not included in the article's Creative Commons license and your intended use is not permitted by statutory regulation or exceeds the permitted use, you will need to obtain permission directly from the copyright holder.
To view a copy of this license, visit https://creativecommons.org/licenses/by/4.0/

We would like to thank the reviewers for their insights and suggestions, and the editor for giving us the opportunity to revise the manuscript.

To address reviewers' comments, we have conducted additional experiments and data analyses. We have also reorganized figures and modified text in the manuscript based on reviewers' requests. All changes were tracked in the revised manuscript.

Our new experiments involve imaging of MEC cells when mice navigated two new tracks, which contained landmarks with different identities, but appeared with equal frequencies. This design tested whether landmark identity encoding is driven by frequency-based salience between different landmarks (Reviewer 2).

In addition, we conduct new analyses related to:
(1) The activity variation and rank correlation between identical landmarks of either high or low frequency within tracks 1 and 2 (Reviewer 2).
(2) The relationship between activity variation and rank correlation as functions of the absolute distance to the water reward in tracks 1-3 (Reviewer 2).
(3) The encoding of landmarks and landmark identity over seven days of learning in track 1 and 2 (Reviewer 2).
(4) Data from the Figs. 1-6 in the original manuscript, except now applied to cue cells after removal of conjunctive cue-grid cells (Reviewer 2).
(5) A comparison of cue and putative grid cells based on a new calculation which is agnostic to the exact activity pattern of either functional class of cell, thereby allowing a fair comparison of the two (Reviewer 3).
(6) A modified study of landmark identity encoding by grid cells with experience (Reviewer 3). We were unable to implement the grid field–based analysis suggested by Reviewer 3, due to the complex and dynamic nature of grid field behavior during learning. We addressed these complexities in detail in this letter and in a separate manuscript under review (attached with this submission: "grid_field_dynamics_manuscript.pdf"), which focuses specifically on grid field dynamics during learning.
(7) Activity shuffles to assess landmark encoding by activity with temporal instability (Reviewer 2).
(8) Activity shuffles to determine the robustness of location and identity encoding by cue cells based on decoding accuracy and rank correlation metrics (Reviewer 3).

The modifications of original figures and texts include:
(1) Largely aggregating data from multiple tracks in the main figures (Reviewer 3).
(2) Increasing the rigor of the statistical analyses for all figures (Reviewer 3).
(3) Text changes to the narrative based on reviewer feedback (Reviewers 1, 2, and 3).
(4) Replacing the original Figs. 7 (cue cell analysis applied to putative grid cells) and 8 (landmark decoding using temporal activity) based on comments by reviewers 2 and 3, and replacing them with a new analysis in Fig. 7.
(5) We now include the statistical information table as a separate document, Supplementary Table 1, due to its length.

Below are our responses (in black text) to all reviewer comments (in blue).

Reviewer #1 (Remarks to the Author):

The study by Wang et al. investigates the medial entorhinal cortex's (MEC) role in encoding both spatial and non-spatial information, specifically the visual identity of landmarks, during navigation. Using two-photon calcium imaging in mice navigating virtual environments, the researchers discovered that MEC, traditionally associated with spatial information processing, also encodes landmark identity through cue cells—neurons that respond to individual landmarks. These cue cells exhibited distinct activity patterns for visually disparate landmarks compared to identical ones, indicating a role in landmark identity encoding. The study further revealed that this encoding is modulated by the spatial relationship between cue cell activity and landmark location and is subject to change across different environments but remains consistent within the same environment despite increased experience. In contrast, grid cells, known for their role in spatial navigation, were found to weakly encode landmark identity but robustly encode landmark location. The authors consider these results to be evidence for the idea that MEC integrates spatial and non-spatial information.

Although landmark coding is an important topic in spatial navigation, I find the advances in this work are incremental. More importantly, many claims are either too strong or inaccurate. Here are my major comments:

(1) The authors claimed that previous studies suggest landmark identity is only coded in LEC and hippocampus in the discussion, and frame this integration of spatial and non-spatial information as one of their major discussion points. I have an issue with this interpretation. Landmarks, as the name suggests, carry spatial information, and even identity information can be useful in inferring spatial relationships. The authors seem to equate the landmark with objects placed inside the arena, and the latter has been used to discern spatial and non-spatial coding of MTL regions. However, as these objects do not provide enough polarizing information, they are not the same as landmarks.

We thank the reviewer for raising this important question. Current literature indicates that objects within an arena can serve as landmarks if they possess recognizable features and provide a stable reference point during navigation[1]. Previous studies have used various types of objects as landmarks, such as cylinders[2] and flowers[3]. Spatial arrangement of objects provides critical spatial information about the environment. For example, the "object location task"—in which the same objects are relocated—has been widely used to assess spatial memory[4-9]. In contrast, nonspatial memory is often tested using the "novel object recognition task," where familiar objects are replaced with new ones without changing their locations[4-6,8,9]. However, objects may fail to function as landmarks and influence neural activity if their locations are unstable within the environment[10,11].

Furthermore, objects within real open environments are encoded by "landmark vector cells" in the hippocampus[12] and "object vector cells" in the MEC[13]. These cells represent distances and directions from objects in open arenas, thus providing vector information relative to landmarks/objects for navigation. Their activity features are very similar to cue cells in our study, which are active at certain distance before and after virtual landmarks. These studies mentioned that "object can play an important role in shaping spatial representation by acting as landmarks"[12], and "in environments that contain discrete objects, animals are known to store information about

distance and direction to those objects and to use this vector information to guide navigation"[13]. Therefore, "objects in an environment have to be considered both in terms of the nonspatial information (such as their identity, shape, color, texture, taste, odor, size, and utility) as well as in terms of their contribution towards defining the space in which the rat moves"[12].

In summary, we believe that objects in an open environment can serve as landmarks, providing spatial information much like the virtual landmarks used in our task. Notably, while the visual appearance of objects and landmarks constitutes nonspatial information, its encoding in the MEC has not yet been reported.

(2) The novelty of the results on cue cells is limited. Previous studies have shown the coding of landmarks by MEC neurons (see Casali et al., 2018, Frontiers in Cellular Neuroscience; and Nguyen et al., 2024, Cell Report by the author's group). Here, the authors performed detailed quantification of the rate modulation, which is apparent in the above citations. This is not a conceptual advance.

We appreciate the reviewer for raising this important question. We believe that the encoding of landmark "identity" by MEC cells represents a major conceptual advance of our study. While the encoding of landmark "locations" in the MEC has been reported in several studies, as indicated by the reviewer[13-16], none have examined the encoding of landmark identity, despite observed variations in neuronal firing rates of cue cells across different landmarks[14]. In contrast, our study not only demonstrates the association between cue cell activity and landmark identity, but also investigates the encoding of landmark identity by grid cells, although the encoding is weaker than that of cue cells. Thus, we provide compelling evidence for landmark identity encoding in the MEC. To our knowledge, such encoding has not been specifically demonstrated in previous studies.

We would also like to clarify that in Nguyen et al., 2024, although we replicated our previous finding that cue cells encode visual landmark locations, we did not address whether these cells encode landmark identity. Furthermore, we did not identify any cue cells responsive to auditory landmarks[16].

(3) In the discussion (Line 569-573), the authors consider the differed activity level (rate remapping) by cue cells to be a novel finding. Quite the opposite, this is a very popular mechanism employed by the hippocampus and associated region, as seen in the numerous cases summarized in a previous review (O'Keefe and Krupic, 2021, Physiol Rev). Even MEC grid cells could use this rate remapping mechanism to code environmental features (Diehl et al., 2017, Neuron).

We agree with the reviewer that it is not surprising for neurons to alter their activity levels, and rate remapping across different environments has been widely documented, including in the study cited by the reviewer (Diehl et al., 2017). However, our key finding is that cue cells in the MEC exhibit activity changes within the same environment, and that this variation corresponds specifically to landmark identity. To our knowledge, this is a novel observation for the MEC, which has traditionally been regarded as encoding primarily spatial, rather than nonspatial, information. In contrast, the combined encoding of spatial and nonspatial information is well established in the hippocampus, as discussed in the review by O'Keefe and Krupic (2021), likely due to the hippocampus integrating inputs from both the MEC and the LEC.

In the revised manuscript, we replaced the previous sentence in discussion (lines 569-573 in the previous manuscript, and lines 590-591 in the revised manuscript) with the following to clarify our finding: “However, cue cells respond to all landmarks but vary their activity at individual ones according to their identity.”

Reviewer #2 (Remarks to the Author):

Ever since discovery of grid cells in medial entorhinal cortex (MEC), they have been thought to play a key role in path integration. Path integration, however, is noisy on its own and prone to error accumulation over time. The errors must be corrected for the system to maintain an accurate spatial code. A critical complementary mechanism to this purpose is error correction, which relies on positional feedback from landmarks. Although our knowledge on grid cells has advanced, the neural mechanisms underlying spatial error correction are still largely unknown. This paper studies a group of MEC cells, termed “cue cells”, that may play a role in error correction. Specifically, the paper addresses an intriguing question of whether “cue cells” encode landmark identity. Although the findings are interesting and the experimental design is clever, I have several major concerns regarding the methodology and presentation of results. I believe these concerns must be addressed before the paper can be considered for publication.

Major comments

• A potential major confound arises from the unequal distribution of landmarks. Across all tracks, one type of landmark (e.g., rectangles) is more frequent than others (e.g., cones), leading to disparate comparisons that are more likely to involve the less frequent landmark type, while identical comparisons are dominated by the more frequent landmark type. For instance, in track 3, the imbalance is even more pronounced, with four dotted rectangles compared to just one instance of each of the other two landmark types. Interestingly, the largest activity variation difference across tracks is observed on track 3, raising concerns that this effect might be driven by the unequal representation of landmarks rather than true landmark identity encoding. If the less frequent landmarks intrinsically exhibit greater variability in neural activity (e.g., due to differences in salience), this imbalance could artificially inflate activity variation in disparate comparisons. To address this, I recommend analyzing the different landmark types separately and/or subsampling to balance the comparisons. Additionally, testing whether the less frequent landmark types exhibit higher intrinsic variability could provide further clarity.

We thank the reviewer for raising these concerns. To address them, we conducted two analyses/experiments:

(1) We compared the activities between identical landmark types that were more (black) or less (red) frequent, testing whether less frequent landmarks exhibit greater intrinsic variability among their own types (Fig. R1). Overall, we did not observe consistent differences in activity variation or rank correlation between frequent and infrequent landmarks. In track 1, infrequent landmarks tended to show lower activity variation and higher rank correlation than frequent ones (Fig. R1A). However, the trends were reversed in track 2 (Fig. R1B). When data from both tracks were pooled, the differences between frequent and infrequent landmarks were not significant (Fig. R1C), indicating that landmark frequency does not affect the intrinsic variability of neural activity.

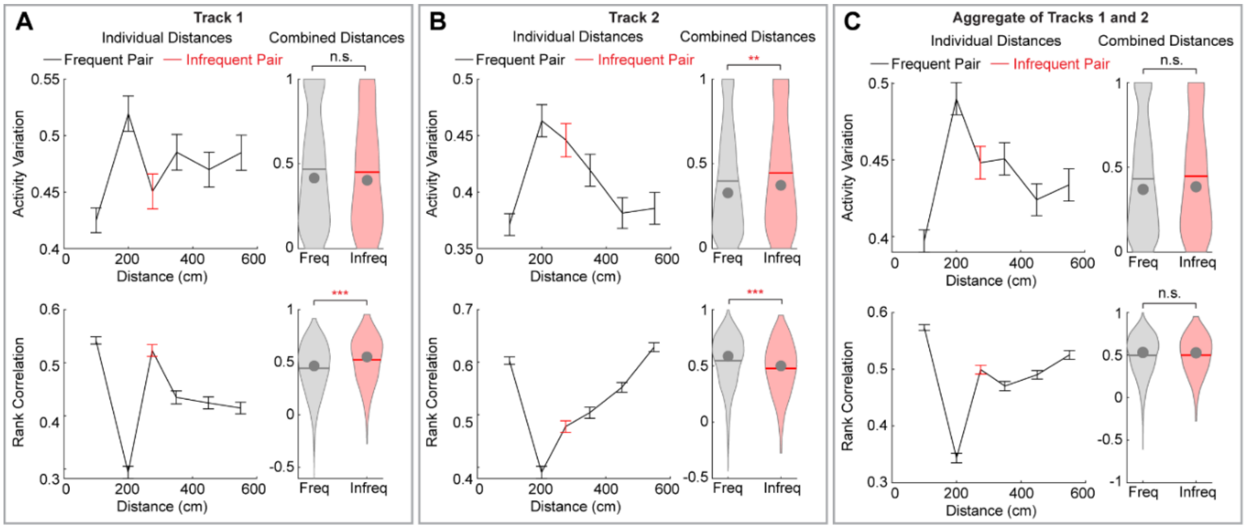

Figure R1. A. Activity variation (top) and rank correlation (bottom) of frequent and infrequent identical landmarks in track 1, at individual (left) and combined (right) distances, evaluated by two-tailed non-paired t-test. **B.** Similar to A, but in track 2. **C.** Similar to A, but with data aggregated from tracks 1 and 2. Each cue cell group contains 15 cells. Statistical information is available in Revision Table 1. Same for all other figures. Data were from 5 mice. Track 1 data were from 434 cells and cell groups. Track 2 data were from 465 cells and cell groups.

(2) We measured calcium dynamics of cue cells in two new tracks (Fig. R2A and B), which contained two types of landmarks with equal frequency. In both tracks, our previous conclusions remained consistent: disparate landmarks were associated with greater activity variation regardless of including all landmarks (Fig. R2C) or only including the two landmark types with equal frequency (Fig. R2D). Consistently, rank correlation was lower between disparate landmark pairs in the two conditions (Fig. R2E and F).

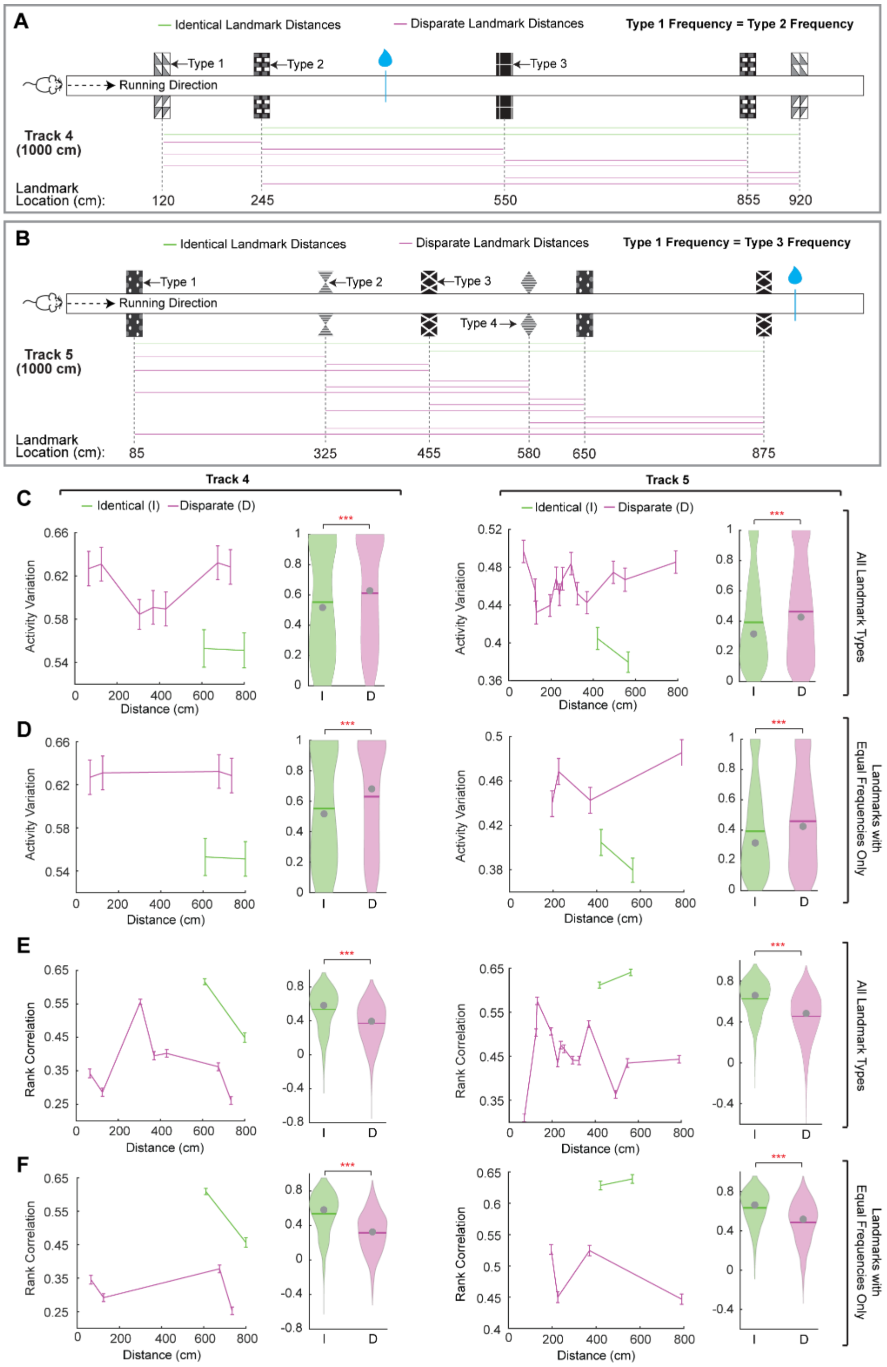

Figure R2. A. The design of track 4 with landmark types 1 and 2 having equal frequency. **B.** The design of track 5 with landmark types 1 and 3 having equal frequency. **C.** Activity variation for all identical (I, green) and disparate (D, magenta) landmark pairs at individual (left) and combined distances (right) for tracks 4 and 5. Asterisks indicate results of two-tailed non-paired t-tests, same in other panels. **D.** Similar to C, except analysis restricted to landmarks with equal frequency. **E-F.** Similar to C and D but for rank correlation. Each cue cell group contains 15 cells.

Track 4 activity variation data were from 11 mice and 498 cells. Track 4 rank correlation data were from 9 mice and 404 cell groups. Track 5 data were from 6 mice and 670 cells and cell groups.

These results together demonstrate that cue cells' encoding of landmark identity is independent of landmark frequency. We have included Fig. R2 as Fig. S4 in the revised manuscript.

On a related note, it would strengthen the results if the authors accounted for the location of landmarks relative to the reward. Given the animal's stereotyped behavior, presumably becoming more attentive as it approaches the reward, and prior literature showing the influence of attention on spatial coding, a possible confound might arise from imbalances in the proximity of landmarks to the reward between disparate and identical comparisons. If landmarks in disparate comparisons tend to be closer to the reward on average, this could bias the results by amplifying

neural variability in those conditions. Including a control analysis to address this potential confound would further solidify the findings.

To address this concern, we calculated activity variation and rank correlation for landmark pairs as functions of their absolute distances from the water reward in tracks 1-3. Since the two landmarks in each pair were positioned at different locations, and thus were located at different distances from the reward, we calculate a metric termed "absolute reward distance," which is the average of the absolute distances (i.e. strictly non-negative) of each landmark from the reward (Fig. R3A). This metric, which is independent of whether landmarks were positioned on the same of the reward (Fig. R3A, a) or on opposite sides of the reward (Fig. R3A, b), allowed us to calculate the absolute reward distance of all landmark pairs in all tracks. This revealed that identical landmark pairs were slightly further away from the water reward on average compared to disparate landmark pairs (Fig. R3B).

Across various absolute reward distances, we replicated our original findings: activity variation was consistently greater for disparate landmark pairs compared to identical pairs in all three tracks (Fig. R3C). Pearson correlations between the absolute reward distances and the averaged activity variations at each such distance were not significant for either landmark pair type (i.e., identical or disparate), indicating that proximity to the reward does not explain differences in landmark discrimination (Fig. R3C). Although absolute reward distances for identical and disparate landmark pairs did not always perfectly match, we calculated activity variation difference by subtracting identical activity variations from the disparate activity variation values with the closest absolute reward distance, within a 15-cm tolerance. When the absolute reward distances did not exactly match, we used the average of the two for plotting the activity variation difference versus distance curve (as in Fig. R3D, track 1). Using this procedure, we show that activity variation difference was not significantly correlated with absolute reward distance and that the trends varied across tracks (Fig. R3D). Similar findings obtained through comparable procedures are evident for populations of cue cells, including strong landmark identity encoding as well as a lack of correlation between absolute reward distance and rank correlation or rank correlation difference (Fig. R3E-F).

These results indicate that the discrimination of identical and disparate landmarks is not influenced by the distance between landmarks and reward. We have included Fig. R3 as Fig. S5 in the revised manuscript.

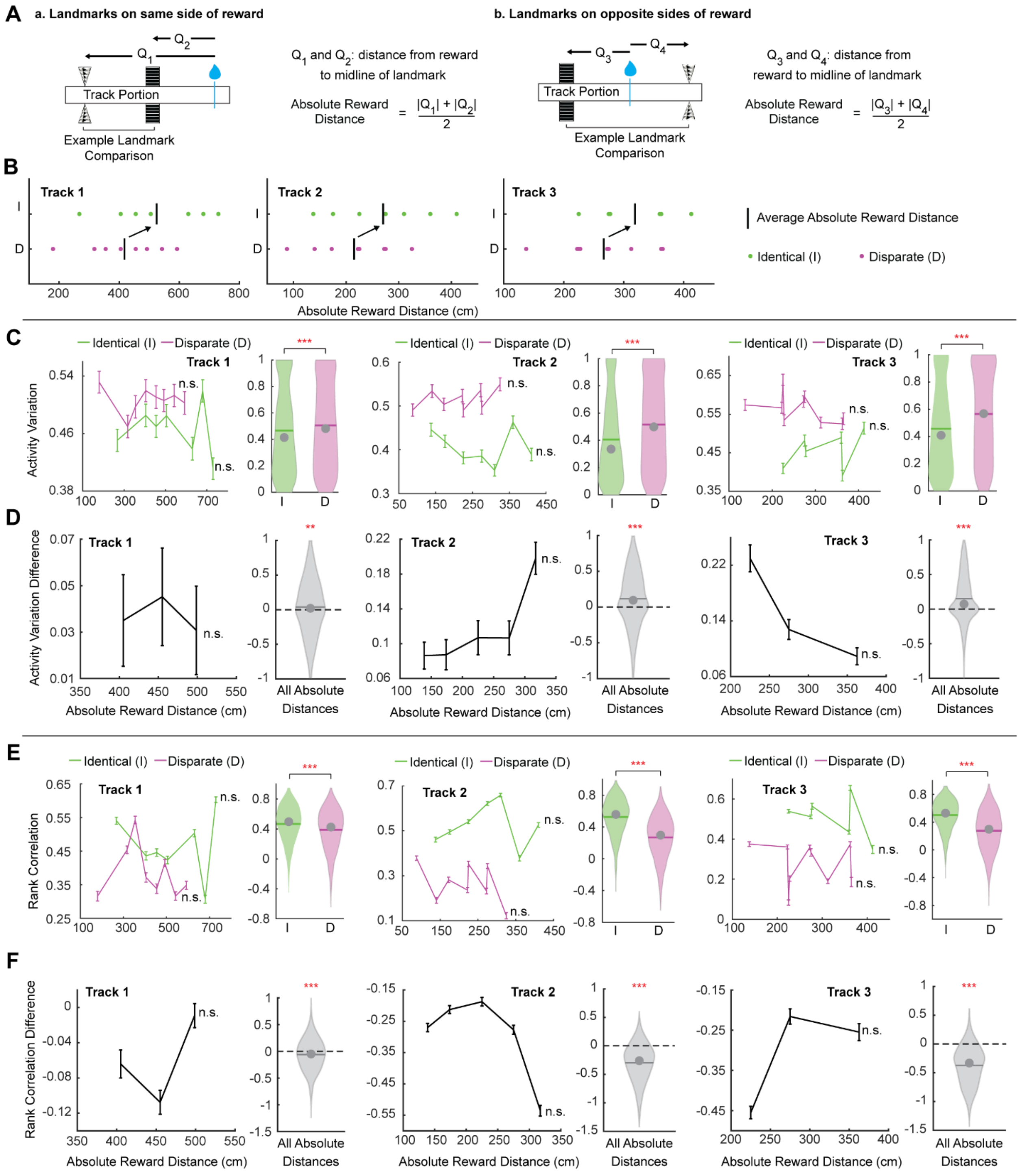

Figure R3. A. Schematic for absolute reward distance of two landmarks on the same (a) and opposite sides (b) of the reward. Q1-Q4 represent distances from the reward to the midlines of individual landmarks. **B.** Absolute reward distances for identical (I, green) and disparate (D, magenta) landmark pairs, with identical landmark pairs being slightly further from the reward than disparate ones. **C.** Activity variation for identical and disparate landmark pairs at individual (left) and combined (right) absolute reward distances in tracks 1 to 3. Non-significant results next to I and D curves indicate the Pearson correlation of averaged activity variations at individual distances with the absolute reward distances, demonstrating no relationship between landmark encoding and distance from the reward. Asterisks indicate the results of two-tailed non-

paired t-tests. **D.** Similar to C, but for activity variation difference. For those absolute reward distances of identical landmark pairs with no matching absolute reward distance for disparate landmarks, we included the closest data point within 15 cm, if available, to conduct the difference calculation. When the distances did not exactly match, we used the average of the two distances for the curve. Non-significant results next to activity variation difference curves indicate the Pearson correlation of averaged activity variation differences at individual distances with the absolute reward distances, demonstrating no relationship between landmark identity encoding and distance from the reward. Insignificant trends are inconsistent across tracks. Asterisks indicate the results of two-tailed paired t-test comparisons with zero. **E-F.** Similar to C and D, but for rank correlation and rank correlation difference. Statistical tests are the same as those in C and D. Each cue cell group contains 15 cells.
Data were from 5 mice, with 434, 465, and 508 cells and cell groups from tracks 1, 2, and 3, respectively.

• Fig. 5 and the corresponding Results section feel disconnected from the rest of the paper and appear to serve more as a supplement to earlier figures (e.g., Figs. 2 and 4) rather than adding new insights. Specifically, Fig. 5D-K (which occupies the most space) largely replicates analyses from Figs. 2 and 4, focusing on common cells across tracks 1 and 2. This analysis could instead be mentioned in the Results sections for Figs. 2 and 4, with a reference to a supplementary figure. Additionally, it is unclear why this analysis only includes tracks 1 and 2 while excluding track 3, and this should be clarified. The other panels of Fig. 5 (A-C) also seem less critical and could be presented as supplementary analyses linked to the Results section for Fig. 2 or Fig 6. Overall, I recommend moving Fig. 5 to the supplementary figures and referring to it in the relevant Results sections to streamline the main text and maintain focus on the primary findings.

We thank the reviewer for raising this concern, but we feel that Figure 5 addresses an important question: whether landmark identity encoding by the same cell or cell group remains consistent across different environments. Figure 6, in contrast, examines whether the encoding remains consistent on different days within the same environment. Since the results of these two figures offer complementary but contrasting insights, we prefer to retain Figure 5 as a main figure.

We would like to clarify that this analysis was conducted between all pairs of tracks. The results for track 1 versus track 3 and track 2 versus track 3 were shown in Figs. S4 and S5 of the original manuscript (now Figs. S12-14 in the revised manuscript).

To focus on the main results, we have moved other cue cell information in panels Fig. 5A-C (cue cell activity plots, cue cell shifts, cue scores) in the original manuscript as well as similar panels from the original Fig. 6 to Fig. S11 of the revised manuscript.

• The difference in activity variations between identical and disparate landmarks in Fig. 6D appears considerably smaller than in Fig. 2, and it must be clarified why this is the case. It seems that the difference may reduce over days, suggesting that activity variations for identical and disparate landmarks might converge with experience. If this is the case, it would challenge the authors' claim that cue cells encode landmark identity, or at least suggest that this encoding diminishes or becomes less distinct in familiar environments. Similarly, Fig. 6E shows a potential trend of decreasing rank correlations over time. Have the authors analyzed whether the activity variation difference settles within 7 days or whether the trend suggests further convergence with more days of experience? Additional analysis is needed to address these questions and clarify the implications for landmark identity encoding over time.

We confirmed that the differences in activity variation between identical and disparate conditions of the same day shown in Fig. 6D of the original manuscript are comparable to that observed in the original Fig. 2 (Fig. R4A and B).

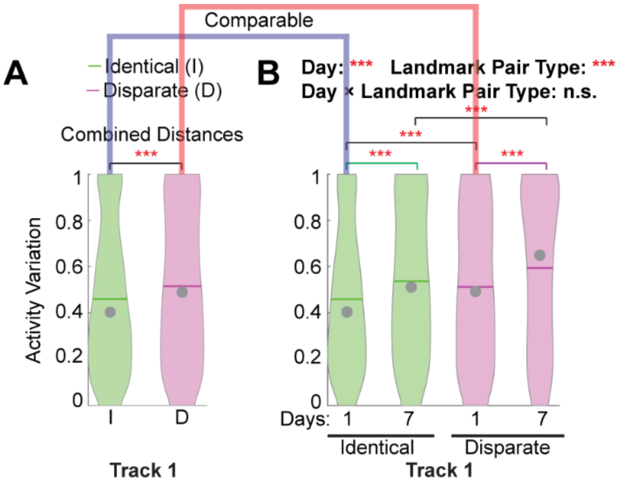

Figure R4. A. Data in original Fig. 2D (track 1, now Fig. S1A). Activity variation for identical (I) and disparate (D) landmark pairs with all distances combined in track 1. Statistical test is a two-tailed paired t-test. **B.** Data in original Fig. 6D (track 1, now Fig. S15A). Activity variation for identical (I) and disparate (D) landmark pairs with all distances combined in track 1 for day 1 and 7. Two-way ANOVA statistics are shown above. Post-hoc statistical tests are two-tailed paired t-tests with Bonferroni-Holm p-value correction. Activity variations for identical and disparate landmarks on day 1 are comparable to those of panel A, as indicated by the purple and red curves connecting the same data across both panels.
Data were from 5 mice, including 434 cells in panel A and 217 cells in panel B.

To better understand landmark identity encoding across days, we investigated calcium dynamics of cue cells across 7 days in each track (tracks 1 and 2). Cue cells were identified as those exceeding the 80th percentile of shuffled cue scores for at least 4 days (i.e., more than half of the 7 days), as conducted previously[17]. We calculated activity variation and rank correlation between identical and disparate landmarks and their differences across days. Consistent with our findings from days 1 and 7, activity variations for identical and disparate landmarks increased in parallel, resulting in largely stable activity variation differences during learning, without any significant upward or downward trend (Fig. R5A and B). Likewise, rank correlation did not exhibit any consistent trend across days (Fig. R5C and D). These results support our conclusion that cue cells' ability to discriminate between landmark identities remains stable with experience.

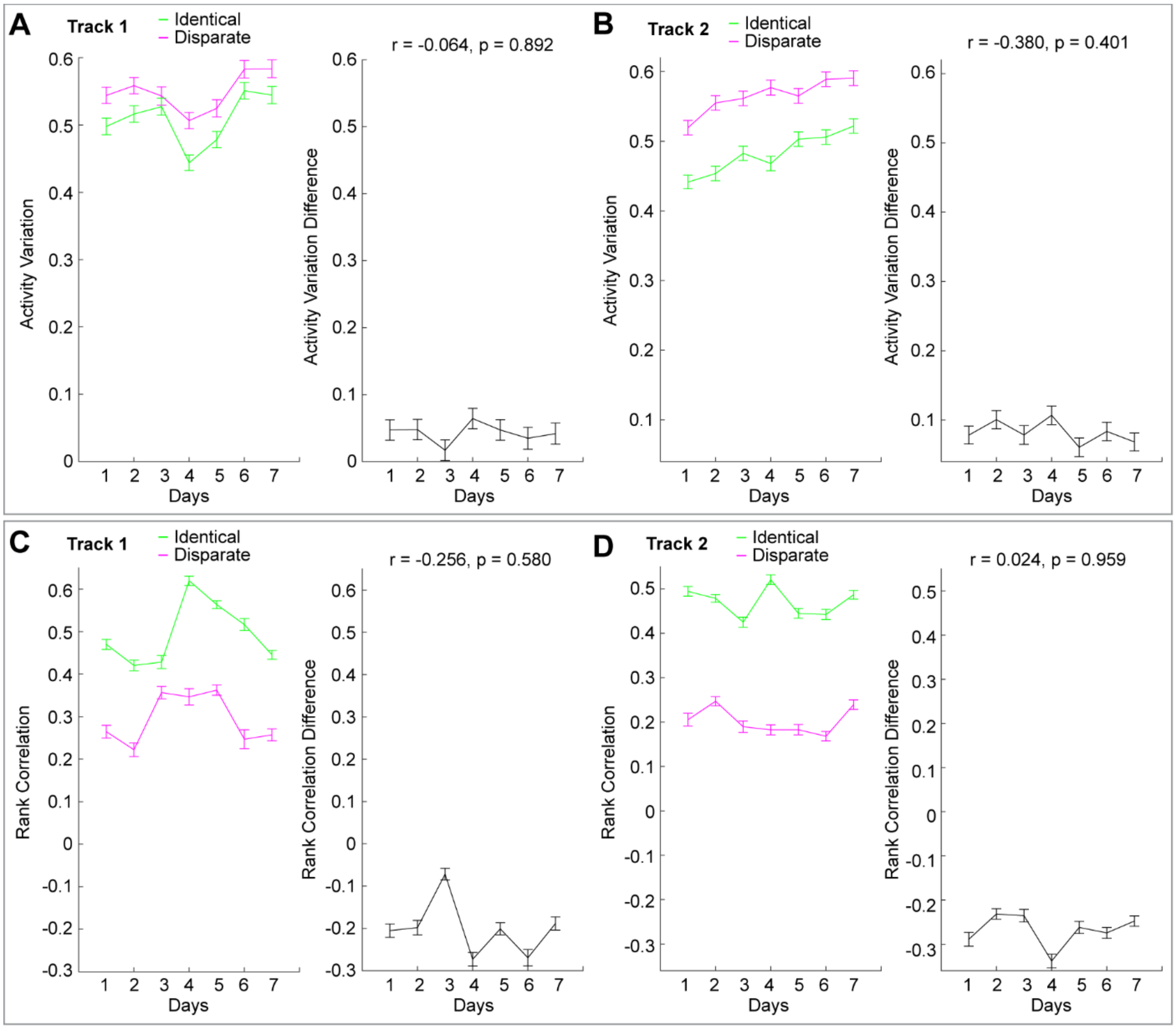

Figure R5. Activity variation (**A and B**) and rank correlation (**C and D**) over 7 days of learning in tracks 1 (left) and 2 (right) for identical (I, green) and disparate (D, magenta) landmarks. The parameters and their differences between identical and disparate landmarks are plotted on the same axis scale to reveal the magnitude of the difference change across days. For the differences across days, "r" and "p" are the coefficient and p-value of the Pearson correlation between the difference and the number of days, respectively. Each cue cell group contains 15 cells.
Activity variation data were from 5 mice, with 159 and 228 cells in tracks 1 and 2, respectively. Rank correlation data were from 3 mice (114 cell groups) as well as 4 mice (188 cell groups) in tracks 1 and 2, respectively.

Moreover, it is unclear why the longitudinal analysis in Fig. 6 is presented only for track 1. The result for track 2 seems to be presented in Fig 7, which is strange. Results of the same analysis should be provided for track 2 within Fig 6 to ensure the robustness of the findings across tracks and for a less convoluted description of results.

The corresponding results for track 2 were shown in Fig. S7 of the original manuscript (Figs. S15-17 of the revised manuscript). We now pool data from tracks 1 and 2 in Fig. 6 for the cross-day analysis and show corresponding individual track data in Figs. S15 and S17.

• The authors should report the percentage of overlap between "putative" grid cells and cue cells and clarify why cue cells were removed from the grid cells not the other way around. This decision appears arbitrary unless a strong and convincing rationale is provided. **It is critical to demonstrate the robustness of the findings by showing that the results in Figs. 1 to 6 still hold when conjunctive grid + cue cells are removed from the cue cell population.** Otherwise, it raises the concern that the reported results might not generalize to "pure" cue cells.
For the sake of transparency, the authors should also explicitly indicate that the identified grid cells are "putative," as it is possible that they may not be true grid cells. There is a lot of redundancy between Fig 7D-E and Fig 6. Authors may just refer to Fig 6 and remove these repeated panels in Fig 7.

We thank the reviewer for raising these important issues. We addressed these comments in three aspects.

(1) As suggested, we analyzed pure cue cells, the activity of which in tracks 1-3 passed cue cell but not grid cell criteria. This procedure removed of 65.52% + 2.98%, 73.46% + 1.64%, and 69.22% + 3.10% (mean ± SEM) of the original cue cell population in each field of view for tracks 1, 2, and 3, respectively.

Figures 2 and 3 of the original manuscript
Consistent with our previous results, pure cue cells showed greater activity variation between disparate landmarks (Fig. R6A), resulting in activity variation differences greater than zero (Fig. R6B). Similarly, pure cue cells showed lower rank correlation for disparate landmarks compared to identical ones (Fig. R6C). Thus, the rank correlation difference was significantly lower than zero (Fig. R6D).

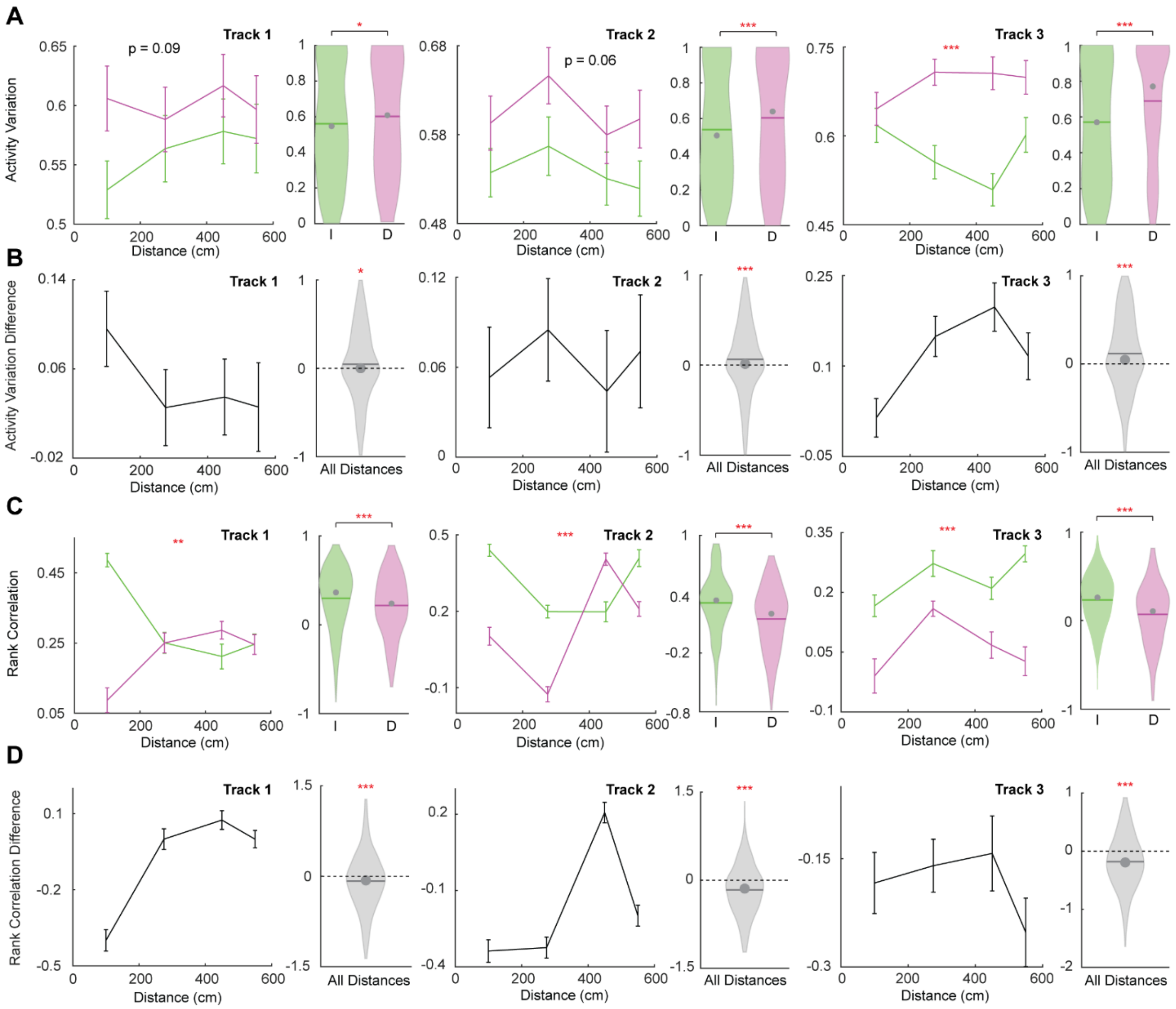

Figure R6. A. Activity variation for identical (I, green) and disparate (D, magenta) landmark pairs at individual and combined distances for tracks 1, 2, and 3. Statistical tests for left panels are two-way ANOVAs while right panels show the result of two-tailed paired t-tests. **B.** Activity variation difference at individual and combined distances for tracks 1, 2, and 3. Statistical test for right panels are two-tailed paired t-tests with 0. **C-D**. Similar to A and B but for rank correlation and rank correlation difference. Each cue cell group contains 10 cells. Data were from 5 mice. Activity variation calculation included 148, 124, and 151 cells for tracks 1, 2, and 3, respectively. Rank correlation calculation included 132, 81, and 127 cell groups for tracks 1, 2, and 3, respectively.

Figure 4 of the original manuscript

Using pure cue cells, we further replicated our findings on landmark identity encoding of cue cells with various spatial shifts (Fig. R7). Pure cue cells active before, at, and after landmarks consistently showed trends, though often insignificant, of higher activity variation for disparate landmarks compared to identical ones at individual distances (Fig. R7A-C), likely due to the reduced sample size mentioned above. When distances were combined, the trends of higher activity variation for disparate landmarks were more often significant (Fig. R7D). The comparisons of activity variation differences for the three cell categories (Fig. R7E), and for cells with various shift directions and distances (Fig. R7F), revealed track-dependent effects. Notably, the difference

in activity variation correlated significantly with absolute spatial shift only in Track 3 for the positive-shift cells (Fig. R7F).

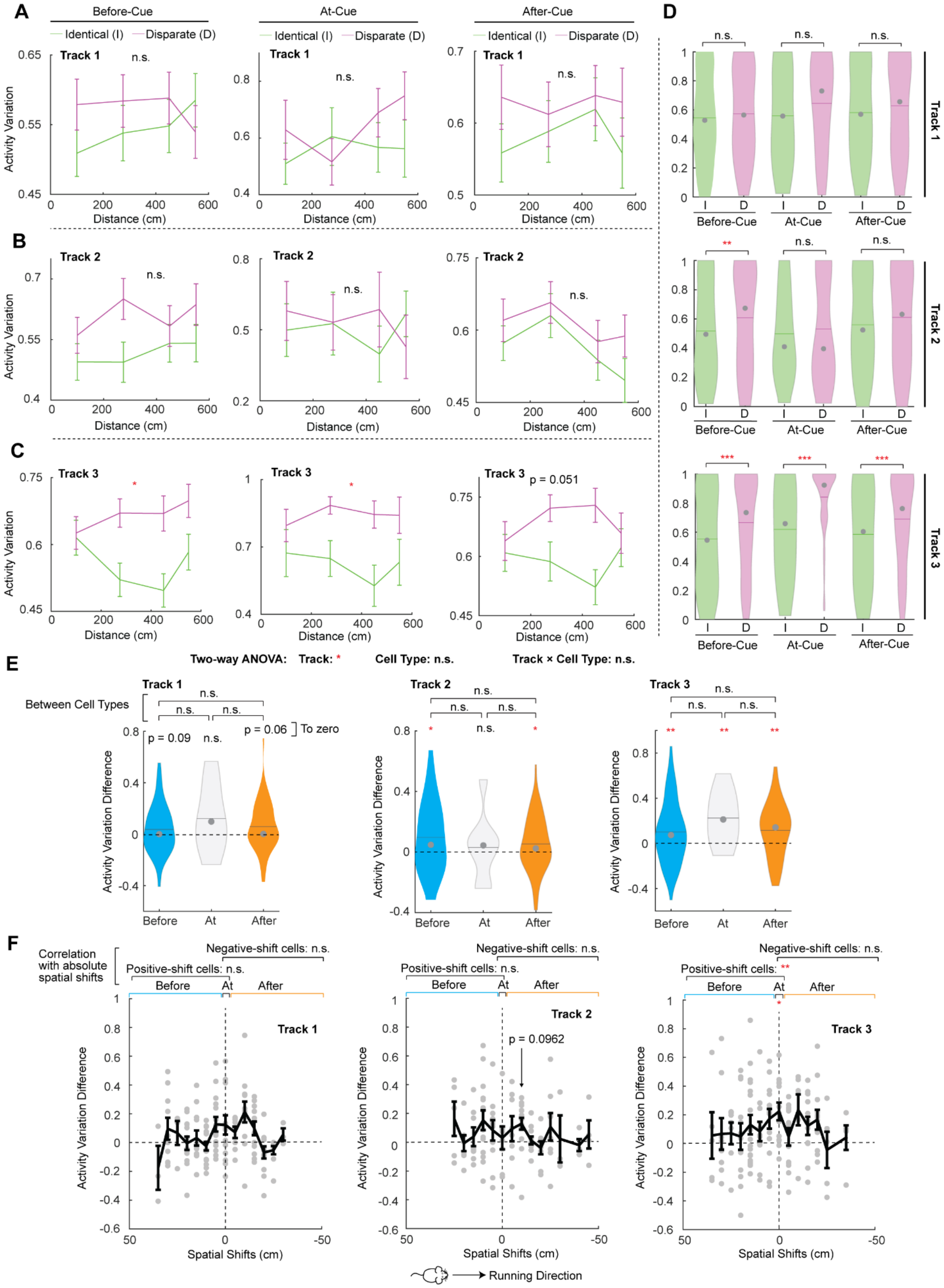

Figure R7. A. Activity variation of pure before-cue, at-cue, and after-cue cells for identical (I, green) and disparate (D, magenta) landmark pairs at individual distances. Statistical tests are two-way ANOVA comparisons of I and D curves with Bonferroni-Holm correction across cell types. **B.** Similar to A, but for track 2. **C.** Similar to A, but for track 3. **D.** Activity variation of pure before-cue, at-cue, and after-cue cells for identical and disparate landmarks at combined distances. Statistical tests are two-tailed paired t-tests with Bonferroni-Holm p-value correction conducted for each track separately. **E.** Activity variation differences of before-cue, at-cue, and after-cue cells compared with zero and between these cell types. Two-way ANOVA results shown above demonstrate differential activity variation differences between tracks. Comparisons with zero are two-tailed paired t-tests (Bonferroni-Holm corrected within each track), while comparisons between cell types are two-tailed nonpaired t-tests (Bonferroni-Holm-corrected within each track). **F.** For each track: activity variation differences of individual cue cells (gray dots) as a function of their spatial shifts. The dashed horizontal line indicates zero difference. The black curve represents the mean and standard errors of the difference at spatial shifts with at least 3 cells. The asterisks next to “positive-shift” or “negative-shift” cells indicate significant correlations between the averaged differences at each spatial shift and the absolute spatial shifts that have at least 3 cells. Paired t-tests for comparisons with zero were Bonferroni-Holm corrected within each track.
Data were from 5 mice, including 148, 124, and 151 cells from tracks 1, 2, and 3, respectively.

Figure 5 of the original manuscript

We further examined common pure cue cells across different tracks. To ensure a sufficient number of cells for each track pair, we included cue cells that were present on day 1 of both tracks and were not classified as grid cells in at least one track. This approach yielded 144, 164, and 154 pure cue cells in track pairs 1 and 2, 1 and 3, as well as 2 and 3, respectively. This represented only ~40%, ~41%, and ~42% of the original common cue cell population. As a result, the statistical significance of some analyses below may be affected by this reduced sample size.

For all track comparisons, our analysis revealed significantly different or a strong trend of different activity variation levels between either identical or disparate landmark pairs between tracks (Fig. R8A). This resulted in largely significant differences in the change in activity variation for identical and disparate landmarks between tracks in two out of three comparisons (Fig. R8B). Moreover, strong trends in differential activity variation difference levels between tracks were evident (Fig. R8C). Activity variation difference for individual cells showed no significant difference for each track comparison (Fig. R8D), likely due to the reduced sample size noted earlier.

We conducted a similar analysis for rank correlation (Fig. R9), demonstrating largely significant results matching those in Fig. R8. Taken together, these results replicated our original finding of differential encoding of landmark identity between different tracks.

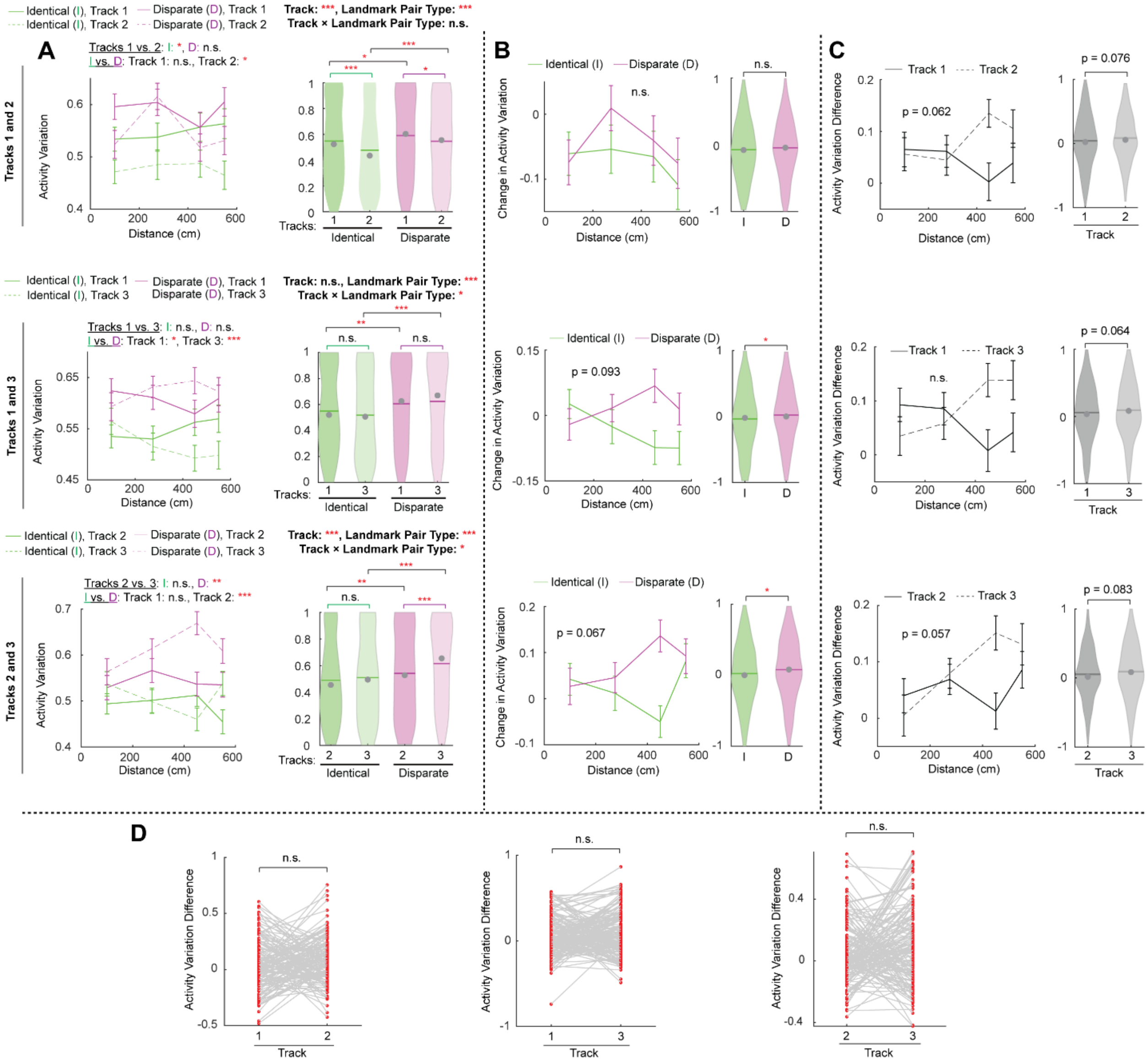

Figure R8. A. Activity variation of pure cue cells for identical (I, green) and disparate (D, magenta) landmarks at individual and combined distances in track 1 vs 2, track 1 vs 3, and track 2 vs 3. Statistical tests in left panels are within-track comparison Bonferroni-Holm-corrected two-way ANOVAs. Statistical tests in right panels are within-track comparison Bonferroni-Holm-corrected two-tailed paired t-tests. Above statistics are two-way ANOVAs for each track comparison showing the role of track and landmark pair type in separately and jointly modulating activity variation. **B.** Change in activity variation for identical (I, green) and disparate (D, magenta) landmarks at individual and combined distances between tracks 1 and 2, 1 and 3, as well as 2 and 3, from top to bottom. Statistical tests in left panels are two-way ANOVAs. Statistical tests for right panels are two-tailed paired t-tests. **C.** Similar to B, but for activity variation differences for all three track comparisons. **D.** Similar to right panels of C, but with activity variation differences of all distances averaged for each cell.

Data were from 5 mice, including 144, 164, and 154 cells for track comparisons 1 vs 2, 1 vs 3, and 2 vs 3, respectively.

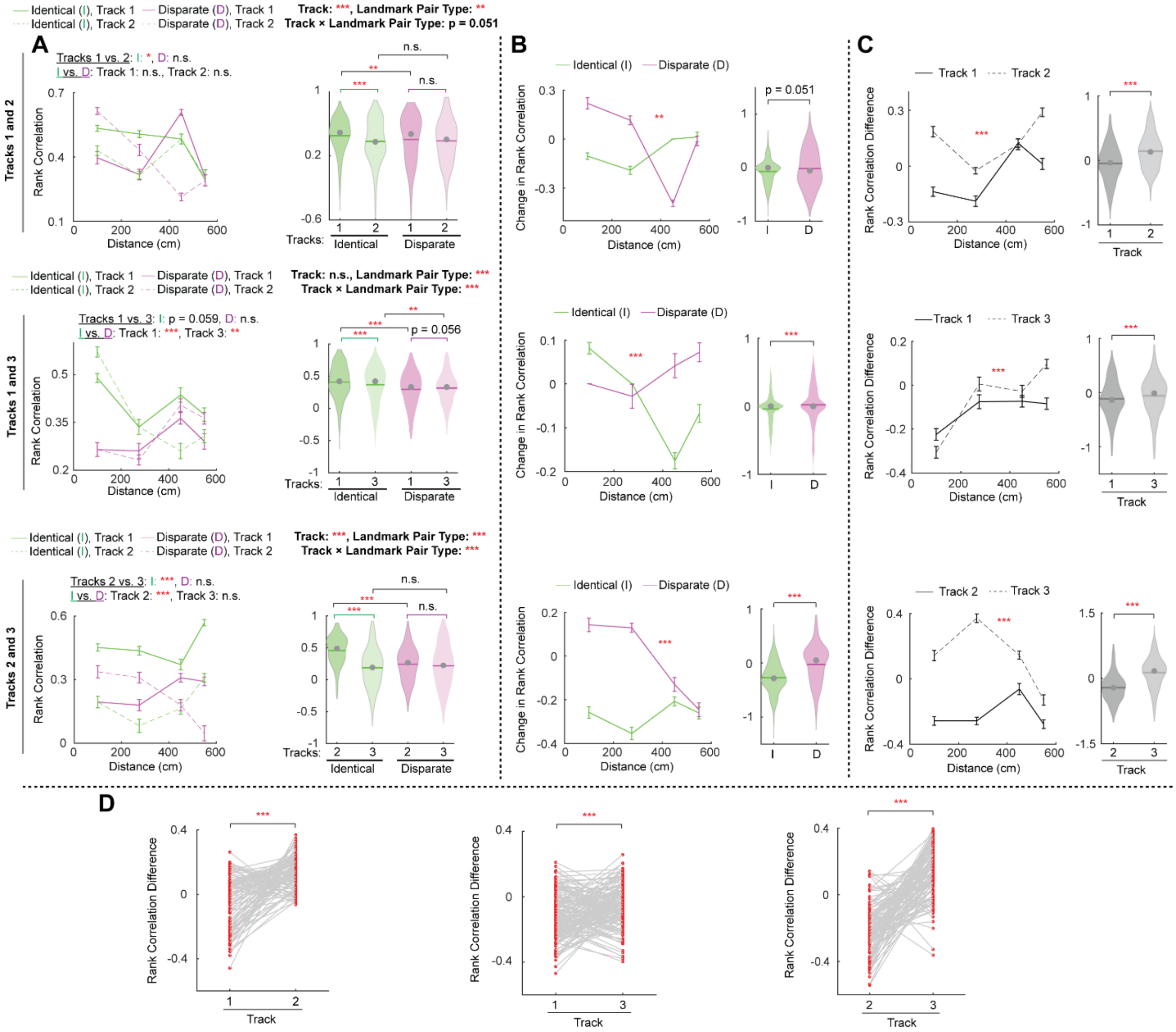

Figure R9. A. Rank correlation of pure cue cell groups for identical (I, green) and disparate (D, magenta) landmarks at individual and combined distances in track 1 vs 2, track 1 vs 3, and track 2 vs 3. Statistical tests in left panels are within-track comparison Bonferroni-Holm-corrected two-way ANOVAs. Statistical tests in right panels are within-track comparison Bonferroni-Holm-corrected two-tailed paired t-tests. Above statistics are two-way ANOVAs for each track comparison showing the role of track and landmark pair type in separately and jointly modulating rank correlation. **B.** Change in rank correlation for identical (I, green) and disparate (D, magenta) landmarks at individual and combined distances between tracks 1 and 2, 1 and 3, as well as 2 and 3, from top to bottom. Statistical tests in left panels are two-way ANOVAs. Statistical tests for right panels are two-tailed paired t-tests. **C.** Similar to B, but for rank correlation differences for all three track comparisons. **D.** Similar to right panels of C, but with rank correlation differences of all distances averaged for each cell group. In all cases, each cue cell group contains 13 cells.

Data were from 3 mice (99 cell groups), 4 mice (153 cell groups), and 4 mice (121 cell groups) for track comparisons 1 vs 2, 1 vs 3, and 2 vs 3, respectively.

Figure 6 of the original manuscript

Lastly, we examined common pure cue cells on days 1 and 7 of the same track. Following the procedure for Figs. R8 and R9, we included only cue cells that were present on both days and were not classified as grid cells on at least one day. This yielded 105 and 115 common cue cells in tracks 1 and 2, respectively, representing only ~48% and ~45% of the original common cue cell population. As expected, we observed increased activity variation from day 1 to 7 for both identical and disparate landmark pairs in both tracks (Fig. R10A). A two-way ANOVA revealed main effects of both day and landmark pair type on activity variation, but no interaction between them, suggesting an experience-independent effect. When examining the change in activity variation from day 1 to 7 for identical and disparate landmark pairs (Fig. R10B), as well as the activity variation difference on days 1 and 7 (Fig. R10C and D), we observed no significant difference. In a comparable study of rank correlation between days 1 and 7, we generally observed expected decreases in rank correlation from days 1 to 7, representing improved discrimination of landmarks (Fig. R11A). However, the change in rank correlation between identical and disparate landmark pairs (Fig. R11B), as well as the rank correlation differences on days 1 and 7 (Fig. R11C and D), were not significantly different. Two-way ANOVA results (Fig. R11A) mirrored those for activity variation (Fig. R10A), supporting an experience-independent modulation.

Together, these findings in pure cue cells support our previous conclusion that landmark identity encoding by the same cue cells or groups of cue cells do not change during learning in the same track.

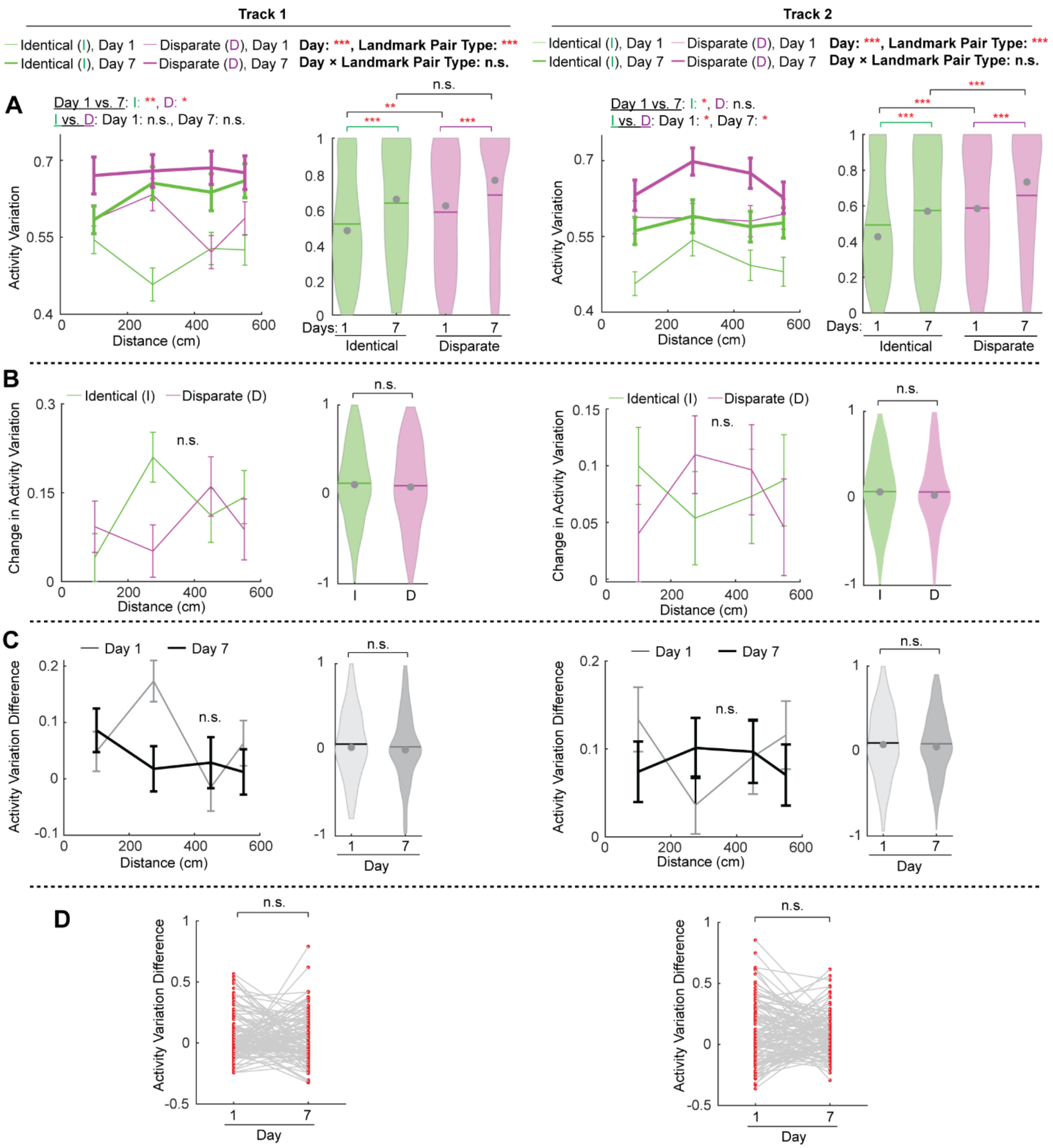

Figure R10. A. Activity variation of pure cue cells for identical (I, green) and disparate (D, magenta) landmarks at individual and combined distances on day 1 (thin lines) and day 7 (thick lines) for tracks 1 and 2. Statistical tests in left panels are within-track Bonferroni-Holm-corrected two-way ANOVAs. Statistical tests in right panels are within-track Bonferroni-Holm-corrected two-tailed paired t-tests. Above statistics are two-way ANOVAs for each track showing the role of day and landmark pair type in separately and jointly modulating activity variation. **B.** Change in activity variation for identical (I, green) and disparate (D, magenta) landmarks at individual and combined distances for track 1 (left) and track 2 (right). Statistical tests in left panels are two-way ANOVAs. Statistical tests for right panels are two-tailed paired t-tests. **C.** Similar to B, but for activity variation differences. **D.** Similar to right panels of C, but with activity variation differences of all distances averaged for each cell.

Data were from 5 mice, including 105 and 115 cells in track 1 and track 2, respectively.

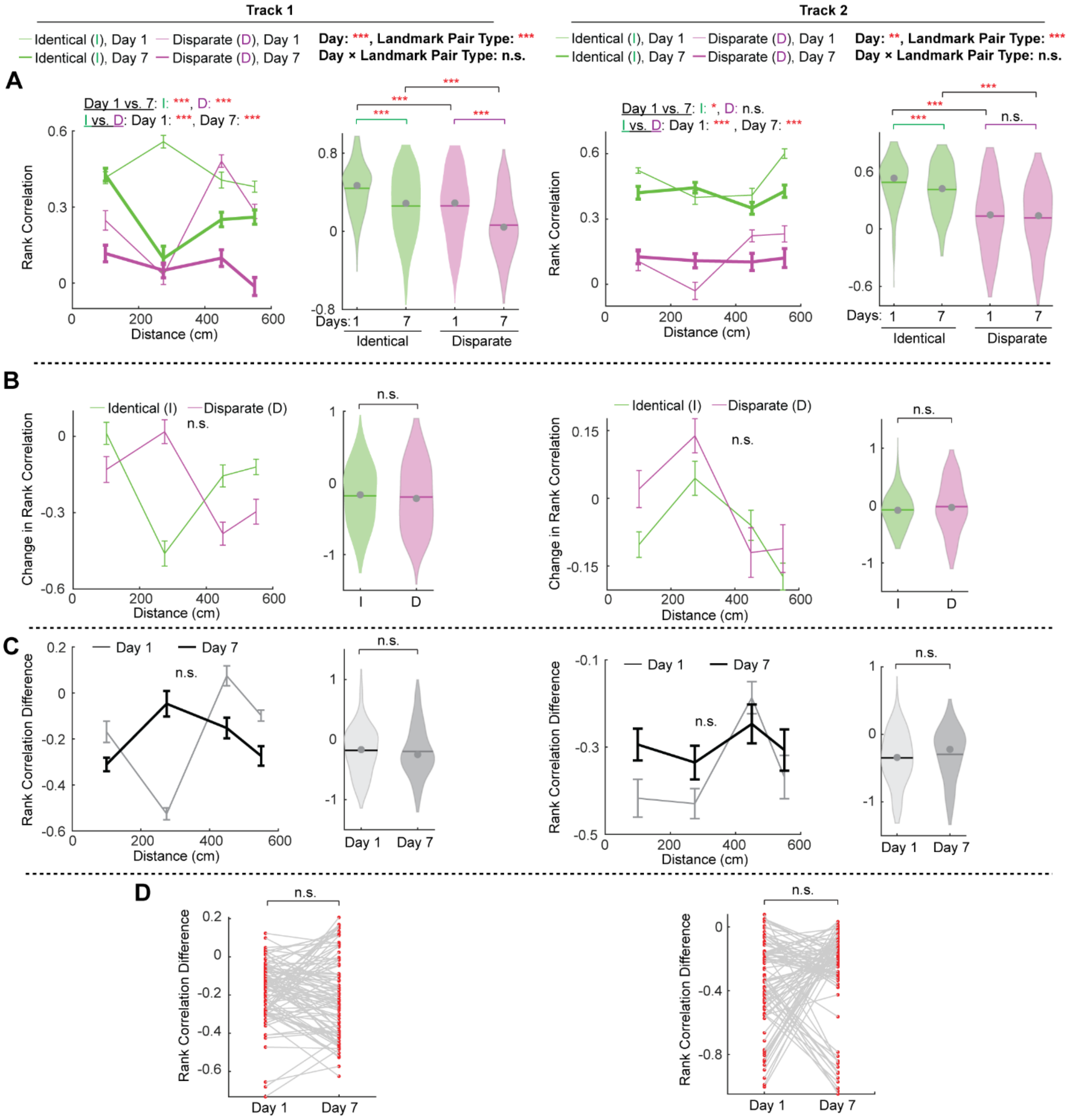

Figure R11. A. Rank correlation of pure cue cell groups for identical (I, green) and disparate (D, magenta) landmarks at individual and combined distances on day 1 (thin lines) and day 7 (thick lines) for tracks 1 and 2. Statistical tests in left panels are within-track Bonferroni-Holm-corrected two-way ANOVAs. Statistical tests in right panels are within-track Bonferroni-Holm-corrected two-tailed paired t-tests. Above statistics are two-way ANOVAs for each track showing the role of day and landmark pair type in separately and jointly modulating rank correlation. **B.** Change in rank correlation for identical (I, green) and disparate (D, magenta) landmarks at individual and combined distances for track 1 (left) and track 2 (right). Statistical tests in left panels are two-way ANOVAs. Statistical tests for right panels are two-tailed paired t-tests. **C.** Similar to B, but for rank correlation differences. **D.** Similar to right panels of C, but with rank correlation differences of all distances averaged for each cell group. In all cases, each cue cell group contains 11 cells.
Track 1 data were from 3 mice and 91 cell groups. Track 2 data were from 4 mice and 87 cell groups.

(2) In the main manuscript, we still used cue cells to refer the cells that passed criteria of both cue and grid cells, based on the definition in the initial paper that characterized cue cells[14]. We indicated identified grid cells as putative grid cells, as suggested by the reviewer.

(3) Regarding Figure 7, we have removed all duplicated figure panels and used a different analysis to compare grid and cue cells, based on the suggestion of reviewer 3.

• I found the decoding analysis (lines 502–514) challenging to follow, and it would benefit from clearer explanation. In particular, it is unclear how the analysis distinguishes between landmark identity encoding and temporal instability (i.e., changes in activity patterns from one lap to another). If the decoding accuracy difference is primarily driven by consistency in activity patterns across laps rather than distinct representations of landmark identity, the results might reflect stability rather than true identity encoding. I suggest revising this section to improve clarity and providing more details on how the analysis accounts for potential confounds, **and demonstrating whether the observed decoding accuracy difference is robust against temporal instability in the neural activity.**

We agree with the reviewer and believe that temporal activity consistency is important for successful decoding, but it is not the sole determining factor. The differential firing between a pair of landmarks is also important. For example, if a cell shows temporally consistent activity across all runs, but the activity at each track location is identical, this activity would not be able to distinguish different landmarks. Thus, the final decoding accuracy is affected by (1) temporal activity consistency and (2) differential firing between landmarks. We agree with the reviewer that it could be difficult to evaluate the relative contributions of the two factors. Based on this fact and the comment of reviewer 3, we have decided to remove this analysis from the manuscript. A new analysis, which only relied on differential firing around individual landmarks, is included in the current Figure 7. This new analysis reproduced our previous conclusion that cue cells more robustly encode landmark identity, whereas grid cells more strongly encode landmark location.

Nonetheless, based on the reviewer's request, we demonstrated the robustness of the previous decoding analysis using temporally permuted activity on a run-by-run basis, representing temporal instability and nondifferential activity between landmarks (Fig. R12). Following decoding of landmark identity from the temporally permuted activity, we demonstrate decoding accuracy of roughly 50% (i.e. chance level for a pair of landmarks) for all conditions of cue and putative grid cells for both identical and disparate landmark conditions on days 1 and 7 of learning, as well as for tracks 1 and 2 (Fig. R12A and B).

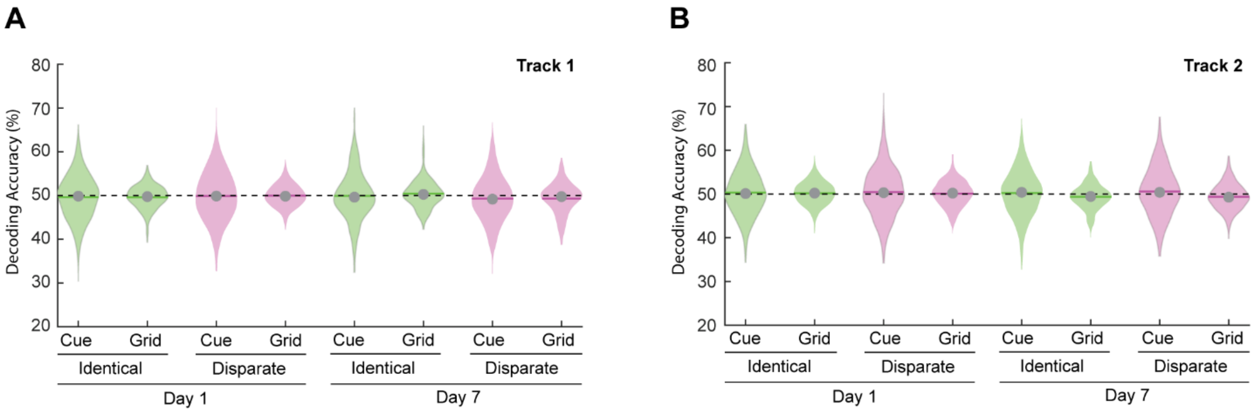

Figure R12. A. Decoding accuracy for temporally permuted activity on a lap-by-lap basis for cue and putative grid cells at identical and disparate landmarks both on day 1 and day 7 of learning in track 1. **B**. Similar to A, but for track 2. Data were from 5 mice. A total of 40 cue cells were randomly selected from each field of view 20 times and the data were aggregated.

Other comments

- Number of animals and number of cells used for the analyses must be reported.

  We have now included this information in all figure legends in the revised manuscript and this letter.

- What is the justification for use of 80th percentile as the cut-off for cue cells across multiple sessions as opposed to 95th or a larger percentile?

  We used an 80th percentile threshold to increase the number of cue cells for the analysis. 95th percentile leads to fewer cue cells, making the statistics difficult.

- What is 1-pvalue?

  This is used to determine the significance of spatial fields and is inherited from a published study[18]. For example, if a track bin's lap-averaged ΔF/F activity is high, a very small percentage of 1000 shuffled mean ΔF/F responses (termed "pvalue") would be higher than the real ΔF/F. If perhaps 2 out of 1000 shuffled mean ΔF/F responses were higher than the true ΔF/F, the pvalue would be 0.002 and 1-pvalue would be 0.998. Having adjacent track bins with 1-pvalue ≥ 0.8 is a condition for in-field categorization. Similarly, out-of-field regions required 1-pvalue ≤ 0.25.

- Landmark template of track 1 in Fig 2C does not match to the template in Fig 1D.

  We thank the reviewer for pointing this out. We have corrected the error in the revised manuscript.

- It is unclear whether the authors chose a single random subset of cells for a given N in analysis of Figure 3 or they subsample all possible subsets (or as many possible)? If a single random subset is used from each FOV, the authors must perform additional analyses to show that their results are not by chance and they hold true for other subsets to.

We chose a single random subset so that the number of samples for the rank correlation analysis was identical to those for activity variation in Fig. 2, ensuring comparable statistical powers of the two analyses. In Fig. S2 of the original manuscript (Fig. S6C of the revised manuscript), we show that for 50 random subsets of cue cells, all p values comparing rank correlation difference to zero were below the 0.05 significance level, except when only 2 cells per group were used. These results indicate that rank correlation difference remains negative—reflecting lower rank correlation for disparate landmark pairs than that for identical ones—regardless of the number of cells per group the specific subset of cells selected.

We now update the panel in Fig. S6C of the revised manuscript by showing the results of the comparisons on a logarithmic scale to clearly demonstrate the robustness of our analysis (Fig. R13). We also improve the rigor of our interpretation of the sets of p-values by determining the percentage of p-values which are significant rather than applying additional statistical tests to, or directly averaging, p-values.

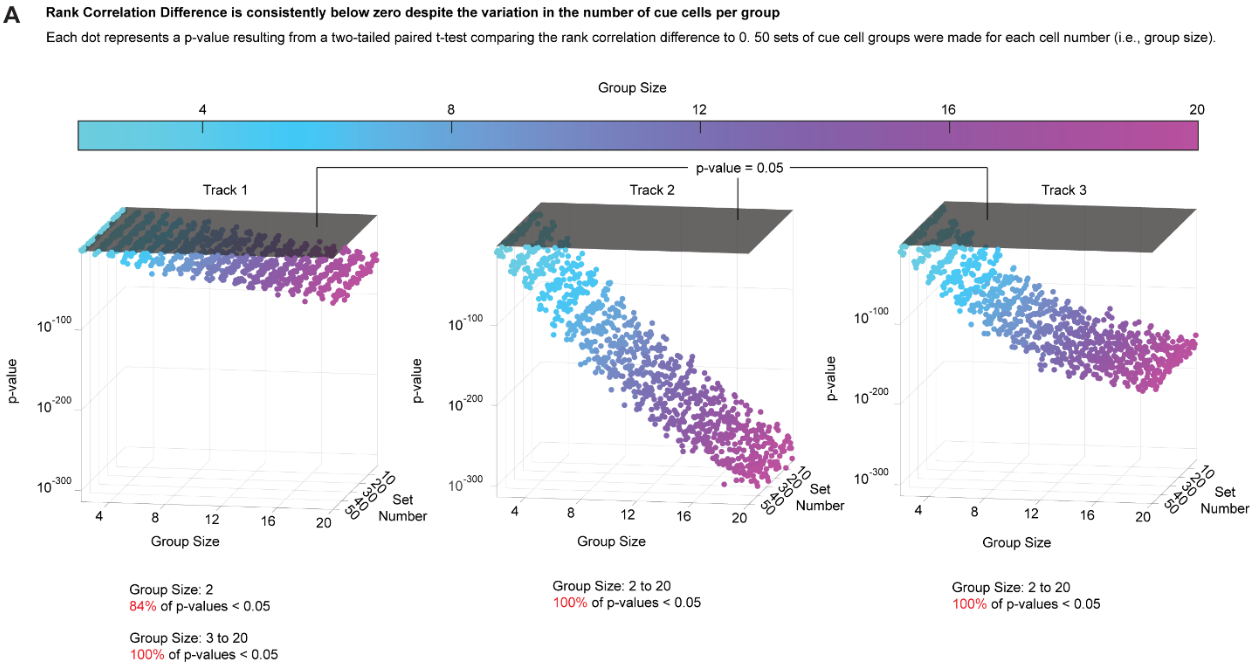

Figure R13. A. The comparison between rank correlation differences and zero calculated using cue cell groups with different numbers of cells (group size). Each dot indicates the p-value for the comparison between rank correlation difference and zero of a set of cue cell groups for each track. Since each calculation was based on cue cell groups randomly chosen from individual FOVs, 50 calculations were made based on 50 sets of cue cell groups for each group size. The individual p-values were color-coded according to the number of cue cells in the group and shown on a log scale in comparison to a significance level of 0.05 (black plane). For various cue cell group sizes, the rank correlation difference compared to zero is significant ($p < 0.05$) for almost all comparisons. The number of FOVs from all 5 mice that were included in the analysis varied based on the number of cells per group. Some FOVs were eliminated when group size increased because the number of cells in the eliminated FOVs were not strictly greater than the group size.

• Why is the second landmark used as a reference in the second and third rows of Fig 3E whereas the first landmark is used as the reference in the first row?

This result is only for a visual demonstration of the rank correlation results. Therefore, we chose the one that are most visually compelling for visualization purpose only. In Fig. R14, we showed the same group of cells in track 1 using the second landmark as a reference. Addressing the reviewer, we replace track 1's visualization of population level encoding of identity with Fig. R14. Additionally, we now only show the visual example of one track in the main figure; we moved the rest (including the one in Fig. R14) to Figure S8 of the revised manuscript.

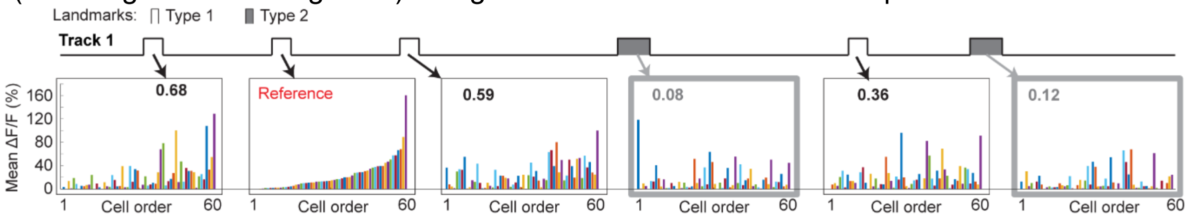

Figure R14. Visualization of the activity of simultaneously imaged cue cells in the same FOV from track 1 at individual landmarks. Cue cells were ranked ascendingly based on their "landmark activity" at the "reference" landmark and their activity at other landmarks was plotted with the same ranking. Rank correlations for these cells between each landmark and the reference are shown at the top of each landmark box. A higher rank correlation corresponds to a more consistent trend of activity ascent at a landmark compared to the reference, indicating that the relative activities of the cells between the two landmarks are more similar. Data were from 60 cells obtained from a single FOV in one mouse.

• Given that cue cells become active at all cues and given that the minimum inter-landmark distance is 100 cm in all three tracks, the spatial shifts of cue cells are expected to be within +-100/2 centimeters. Fig 4B can be simply explained by this expectation?

To calculate the shifts of cue cells, we allow the shift range to be ± 300 cm, following a previous study[14]. Thus, this accumulation of shifts between -50 and +50 cm is not necessarily expected. In the previous study[14], the smallest distance between cues in three tracks ranged from 92 to 130 cm, and the cue shifts were also confined between -50 and +50 cm. This suggests that the shift range is an intrinsic feature of putative cue cells rather than a track-based characteristic. Additionally, the main point of Fig 4B is that the shifts are skewed to the prospective direction.

Reviewer #3 (Remarks to the Author):

The manuscript presents a compelling exploration of whether the medial entorhinal cortex (mEC) encodes landmark identity, providing a pathway in addition to the lateral entorhinal cortex for non-spatial information to reach the hippocampus. The experimental design and use of virtual environments are innovative and well-controlled for the research question. I also appreciate the effort to test landmark identity encoding across multiple contexts, as well as across days, adding depth to the investigation beyond what is currently known. Overall, the manuscript convincingly demonstrates that cue (or landmark) responsive cells in the mEC differentiate between landmarks in a manner that evolves over days and across environments.

However, I have major concerns with the interpretation of analyses and statistical methodology, as well as some conceptual oversights, as outlined below.

Major Comments:

1. My biggest issue with the current version of the manuscript is its narrative, partially in the introduction and definitely in the discussion. The authors describe what used to once be the prevailing view of entorhinal functional segregation – 'mEC provides spatial information, while lEC provides non-spatial information' as a core assumption that their manuscript refutes, but this view is no longer the basic assumption. There have been numerous studies describing abundant non-spatial information coding in the mEC (Hardcastle et al., 2017, for a review; Diehl et al., 2017; many others cited in this manuscript and previous work from Dr. Gu's lab). I suggest the authors rewrite the narrative to acknowledge that information in the EC is not as segregated as was previously hypothesized. What this manuscript provides, quite nicely, is a thorough description of the landmark identity coding abilities of mEC neurons. The authors should focus on this aspect rather than the broader non-spatial and spatial tuning.

We have made the change in the introduction and discussion. In the introduction, we removed the statement about the separate encoding of spatial and nonspatial information in the MEC and the LEC, respectively, and specifically focused on the encoding of both types of information in the MEC. We cited the papers mentioned by the reviewer and also additional studies to support our statement. In the last paragraph of discussion, we proposed that that "although the MEC and LEC show preferential encoding of spatial and nonspatial information, respectively[18-23], both regions are capable of integrating spatial and nonspatial signals before transmitting them to the hippocampus. This architecture supports both specialized local computations within MEC and LEC circuits and the generation of a comprehensive cognitive map across the broader entorhinal-hippocampal network, ultimately enabling robust and flexible navigation".

2. My other conceptual issue with the manuscript is the claim that they have uncovered 'distinct circuit mechanisms supporting non-spatial and spatial encoding' in mEC. There is no data to truly suggest the existence of distinct mechanisms. I have several concerns with the analysis of grid cells (elaborated in a later point). But even barring those, effectively what the authors have done is subselect two groups of neurons – one, the 'cue cells,' which respond to landmarks, and the other, 'grid cells,' which fire in fixed spatial locations. Analyzing these cell types will obviously yield differential results on landmark responses and location responses respectively, and it is unclear how these differences reflect underlying circuit mechanisms versus a result of the subselection.

We agree with the reviewer's comment and have removed the statement referring to a "different circuit mechanisms" or "different mechanisms" based on the differential encoding by putative grid and cue cells, or by cue cells alone.

3. Statistical analyses throughout the manuscript lack details (e.g., F, Z statistics, corrections for multiple comparisons, number of samples). Also, the main statistics and type of test performed should be reported in the figure legends rather than a supplementary table (which also provides no details). In several plots, the choice of statistical tests is also questionable, e.g., plots like Figure 8B, E should use two-way ANOVAs with posthoc tests rather than multiple t-tests.

We have included details of the statistical tests used in the figure legends. However, adding p-values—and especially more extensive statistical details—directly into the legends significantly reduces readability. We therefore retained additional statistical information in a supplementary table for the revised manuscript and as well as for this rebuttal letter.

For panels comparing a metric for multiple distances, we used a two-way ANOVA (metric x distance) for all comparisons and reported F statistics. The p-values reported were between-subjects comparisons (e.g., Identical data compared with disparate data) which were Bonferroni-Holm corrected. For the correlation of any metric over a distance, we used a two-tailed Pearson's linear correlation of the average of each metric with the horizontal axis vector of corresponding values. In the case of plots of individual cells or cell groups with coordinates (e.g., Fig. 2F of the revised manuscript), we conducted two-tailed Pearson's linear correlations including each individual data point. In both of these situations, we reported the r value or R^2 value (in the case of fitted lines) in the revised supplementary table.

In the case of panels such as Fig. 5A (right) (activity variation for identical and disparate landmark pairs across tracks 1 and 2) in the revised manuscript, we included two-way ANOVA results with within-subject factors (track and landmark pair type) above. Post-hoc tests were two-tailed paired t-tests, which were Bonferroni-Holm corrected. Our sample sizes were quite large in this study, allowing use of the t-test for post-hoc comparisons due to the Central Limit Theorem. For comparisons in which the samples differed in distances, number of cells or cell groups (including when the samples were not of the same cells or cell groups), we used two-tailed unpaired t-tests.

Moreover, the two-way ANOVA in Fig. S22F in the revised manuscript was a mixed-effects model with Sattherthwaite-adjusted denominator degrees of freedom since this was a comparison of different cells (cue vs putative grid cells) for some comparisons as well as of the same cells (day 1 vs day 7 for the same functional class of cell) in other comparisons. In addition, in Figs. 4C and S9B of the revised manuscript, which are comparison of the percentages of cells in each FOV by cell types (i.e., before-cue, at-cue, or after-cue), we show one-way ANOVA comparisons with appropriate post-hoc Bonferroni-Holm error correction to ascertain the role of cell type in modulating the percentage of each cell type's occurrence.

Details of Bonferroni-Holm corrections as well as additional information were provided in the revised Supplementary Table 1.

4. I appreciate the schematics provided by the authors to explain their analytical approaches. Despite this, the manuscript is quite confusing, and often, the main point of the figure becomes

hidden or unclear. Part of the problem is that there are so many conditions and comparisons (disparate versus identical cues, types of tracks, day 1 vs day 7), it is hard for the reader. One suggestion would be to combine or condense results across tracks and perhaps show individual track results in the supplement. Only the figure comparing across tracks (Figure 5) can be retained. Reducing the overall number of plots in a figure would greatly help the readability of the manuscript.

We have made the requested changes in the revised manuscript. In addition to Fig. 5, we retain individual track results for some panels in Fig. 4 of the revised manuscript due to our observed track-dependent results.

Below we address three questions together since they are all related to grid cells:

5. Line 502: "However, since the above analyses were designed for landmark-specific activity of cue cells, they may not properly reveal landmark identity encoding by grid cells." This is a major issue of the grid cell analysis. Grid cells may or may not have a field near each landmark, so simply correlating activity around same and different landmarks is misleading. The authors should perform the analysis around defined grid fields, perhaps comparing fields near distinct landmarks to see if the rate in each node is differentially modulated as reported in previous studies.

Moreover, the main result in Figure 8 is the comparison between grid and cue cells (Fig. 8H). But the differences in decoding accuracy are quite minimal, and not surprising, especially because one would expect grid cells to very effectively differentiate between landmarks. Overall, I am unconvinced that the grid cell analysis adds to this manuscript, especially because the ability to decode location is obvious. Perhaps the authors should focus more on how cue identity and experience alter individual grid nodes, following up on past work in open fields (Diehl et al., 2017; Ismakov et al., 2017; Boccara et al., 2019).

Minor Comments:

4. The spatial shift of landmark cells, reflecting more prospective tuning, is extremely interesting. Do grid cell fields also demonstrate any shift in their fields over experience on the track?

Field-based grid cell analysis across learning days is challenging

We agree with the reviewer about the lack of applicability of the cue cell analysis to grid cell activity. Based on the reviewer's suggestion, the proposed grid-field-based analysis would require two steps:

(1) On day 1, calculating the amplitude variation of grid fields near pairs of identical or disparate landmarks, and then deriving the amplitude variation difference between the two types of landmark pairs to evaluate encoding of landmark identity encoding.
(2) On day 7, tracking the same sets of fields in (1) and determining whether landmark identity encoding alters between these days.

However, our recent study[19] showed that grid fields in one-dimensional (1D) linear track are highly dynamic over multiple days of spatial learning, making the above analyses unfeasible, as explained below.

First, on day 1 of a novel track with multiple landmarks (Fig. R15A), we observed that grid fields were generally uniformly distributed between pairs of landmarks, making it difficult to assign specific fields to individual landmarks (Fig. R15B).

Second, tracking the same grid field from day 1 to day 7 is challenging due to their instability across days. Grid cell activity in a 1D linear track can be simulated as a slice through a 2D triangular lattice[20]. Our recent study showed that during multiple days of learning, this slice shifts and rotates, leading to appearance and disappearance of fields on each day (Fig. R15C for two example cells). Although these dynamics decrease over learning while the slice stabilizes, they make it hard to determine the same fields on days 1 and 7. Our results indicate that from day 1 to day 10 in novel environments, most grid fields only last 2-4 days (Fig. R15D).

Grid cell field over experience
We observed that grid fields exhibit backward shifts on a run-by-run basis within and across days (backward fields, Fig. R15C and E). Other cross-day fields shift in forward direction (forward fields) or exhibit no directional shifts (stationary fields) (Fig. R15E). Notably, the percentage of backward fields is significantly higher than that would be expected by chance, whereas the percentages of other field types are lower (Fig. R15F). These backward fields gradually shift toward the nearest landmarks behind them (i.e., the back landmarks), and correspondingly, away from the one in front (i.e., the front landmarks). The fields eventually cluster near the back landmarks with a low likelihood of passing them (Figure R15G), forming a grid map that over-represents salient landmarks. These results are detailed in our current study, which is under review[19]. We have also attached this manuscript with this submission (“grid_field_dynamics_manuscript.pdf”).

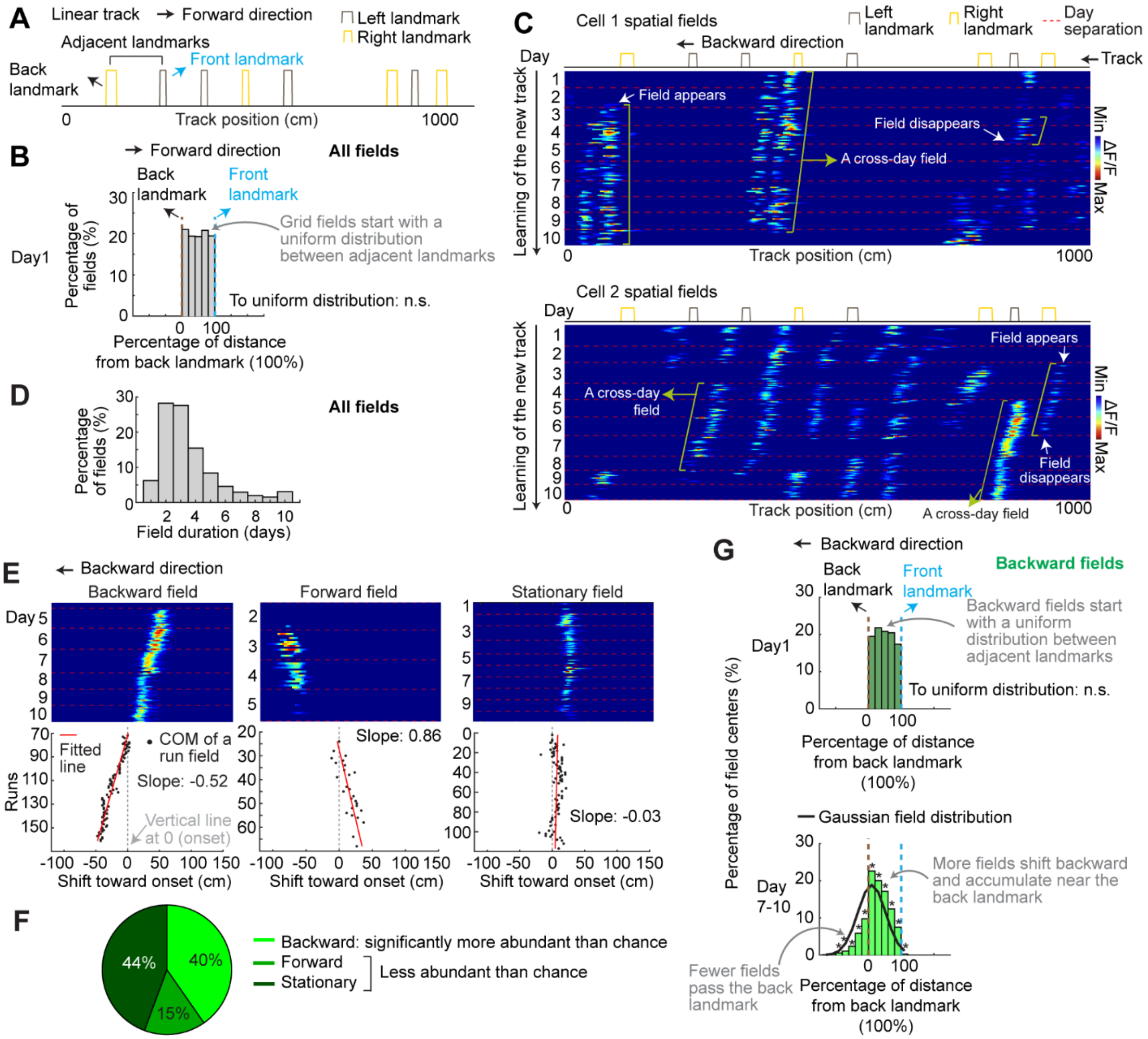

Figure R15. A. A linear track with landmarks on the left and right sides of the track (left and right landmarks, respectively). For an adjacent landmark pair, front and back landmarks are those in the forward and backward running directions along the track, respectively. **B.** Distribution of all cross-day fields between adjacent landmark pairs on day 1 in the track shown in A. The total distance between each adjacent landmark pair is defined as 100% so that the distributions between all adjacent landmark pairs can be pooled together. The distribution of these fields is not significantly different from a uniform distribution, indicating evenly distributed fields between adjacent landmarks. **C.** Example calcium dynamics of cross-day fields (green brackets and arrows) of two putative grid cells. White arrows indicate field appearance and disappearance. **D.** Distribution of all fields' duration. Note that most fields last 2-4 days. **E.** Examples of three types of cross-day fields (from left to right: backward, forward, and stationary fields). For each type, top: calcium dynamics of a field; bottom: centers of mass (COMs) of run fields with their linear regression results. **F.** Percentage of three cross-day field types in behaving mice, calculated by pooling all cross-day fields of the same shifting type across putative grid cells and FOVs together. **G.** Top: distribution of cross-day backward fields with their day 1 fields between landmark pairs. Bottom: distribution of day 7-10 backward fields. The percentages of fields that do or do not cross back landmarks were compared with 20000 simulated percentage values (black), each of which was constructed by the same number of fields that formed a normal distribution around current peak location and covered the same range of track position. Data were from 11 mice, together with 267 cells.

New analysis in the current Figure 7:
Although the grid field dynamics across days are interesting, they would certainly complicate the field-based analyses proposed above—particularly in the final figure of the manuscript. Moreover, our previous analyses of cue cells were all based on spatially-binned activity, rather than on significant spatial fields. Givin these considerations, we conducted a new study of cue and grid cells that does not rely on spatial fields or specific activity patterns around landmarks (Fig. R16). This approach allowed for a fair comparison between cue and grid cells. The analyses in the original Figs. 7 (cue-cell analysis for grid cells) and 8 (decoding) have been removed based on reviewers' comments. Nonetheless, all three analyses had converged on the same conclusion: that landmark identity and location are preferentially encoded by cue cells and grid cells, respectively, demonstrating the robustness of our conclusion.

We focused on the integrated activity of cue and grid cells within a region around the midpoint of the landmark (i.e., integrated activity). The results for a ± 25 cm region are shown in Fig. R16A-G, with additional region sizes tested in Fig. R16H. For each cell, we first averaged integrated activity at each landmark, and then calculated the integrated activity variations between identical and disparate landmark pairs. The difference in these variations, referred to as the integrated activity variation difference, was used to assess each cell's ability to encode landmark identity (Fig. R16A).

This analysis reproduced landmark identity encoding observed in cue cells, as evidenced by greater integrated activity variation between disparate landmark pairs compared to identical ones (Fig. R16B). For both types of landmark pairs, the variation increased from day 1 to day 7, reflecting improved spatial encoding with experience (Fig. R16B). However, two-way ANOVA statistics showed no significant joint effect of day and landmark pair type, reflecting experience-independent landmark identity encoding (Fig. R16B, top). This was confirmed by the comparable integrated activity variation differences on days 1 and 7 (Fig. R16C). Thus, during learning, cue cells enhance their encoding of landmark location, while maintaining stable encoding of landmark identity.

For putative grid cells, we observed that on day 1, the integrated activity variation between disparate landmark pairs tended to be higher than that between identical ones (Fig. R16D), resulting in an integrated activity variation difference significantly greater than zero (Fig. R16E). This suggests a weak encoding of landmark identity on day 1. However, such encoding was not observed on day 7. Moreover, integrated activity variations for both types of landmark pairs did not increase from day 1 to day 7 (Fig. R16D) suggesting that learning-related spatial encoding improvements in grid cells–reported in other studies[17]–may instead be captured by other activity features, such as increased spatial activity consistency[17].

A comparison of integrated activity variation for cue and putative grid cells for identical and disparate landmarks shows that putative grid cells generally exhibited greater variations (Fig. R16F), consistent with their established role in spatial encoding. However, cue cells demonstrated significantly greater integrated activity variation differences on both Day 1 and Day 7 (Fig. R16G), indicating that they more robustly encode landmark identity.

Interestingly, the identity encoding by grid cells is affected by the proximity of their activity to landmarks (i.e., the size of the studied activity region). On day 1, we found that the integrated

activity variation difference for putative grid cells gradually approached zero as the activity range increased (Fig. R16H, Grid Day 1), suggesting that early in learning, grid cells encode landmark identity primarily through activity confined to a small region near the landmarks. On Day 7, however, this identity encoding was no longer present, regardless of region size (Fig. R16H, Grid Day 7). In contrast, cue cells maintained robust identity encoding across all region sizes on both Day 1 and Day 7 (Fig. R16H, Cue Day 1 and 7).

In summary, the above analyses reproduced our previous conclusion that cue cells more strongly encode non-spatial landmark identity, while grid cells more robustly encode the spatial properties of landmarks (Fig. R16I).

Fig. R16 is currently included as Fig. 7 of the revised manuscript, and we also included analyses of individual tracks as Figs. S20-22, which show the same conclusion as the pooled data.

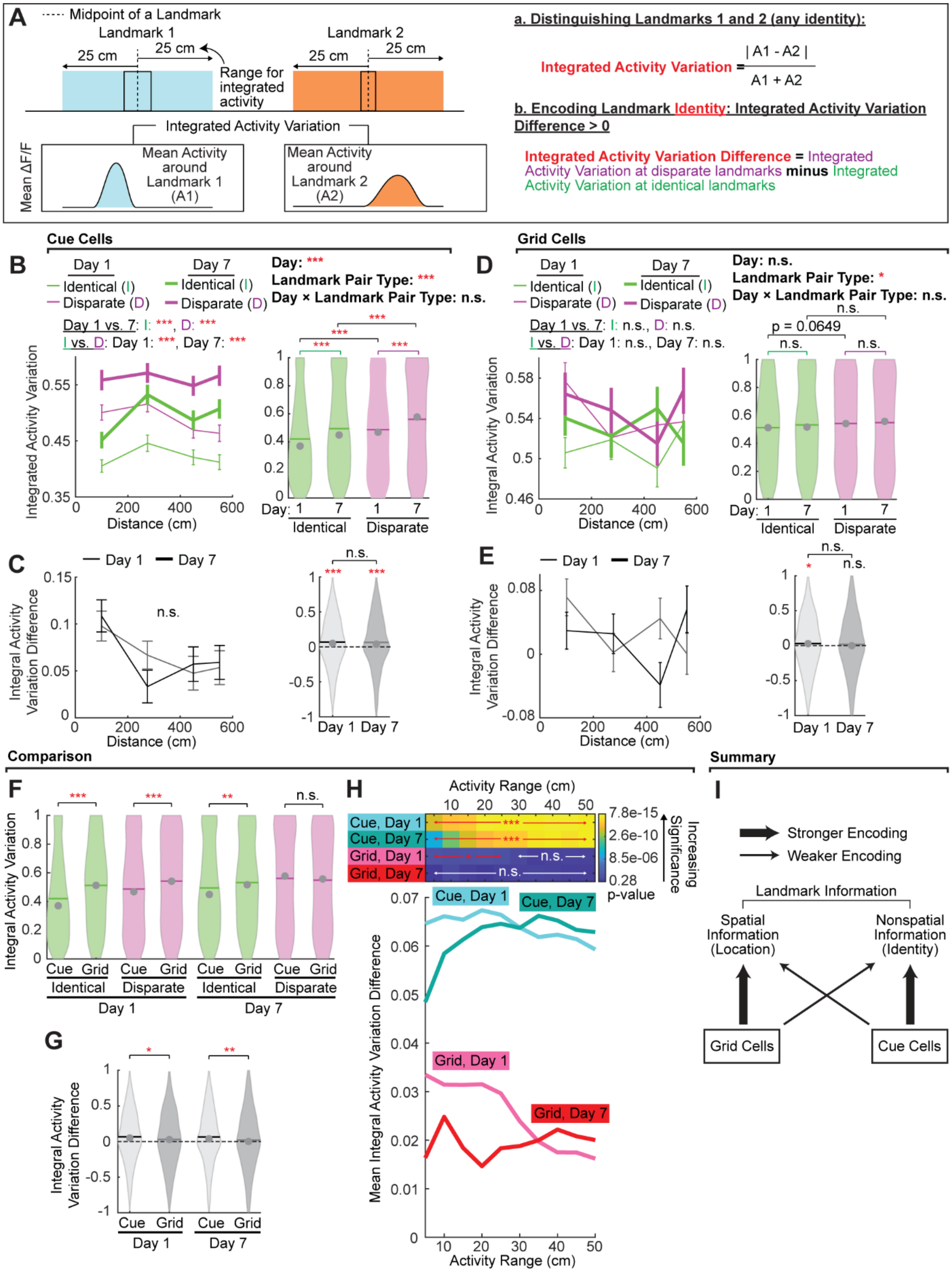
A
Midpoint of a Landmark
Landmark 1
Landmark 2
25 cm
Range for integrated activity
Integrated Activity Variation
Mean ΔF/F
Mean Activity around Landmark 1 (A1)
Mean Activity around Landmark 2 (A2)
a. Distinguishing Landmarks 1 and 2 (any identity):
Integrated Activity Variation = | A1 - A2 | / A1 + A2
b. Encoding Landmark Identity: Integrated Activity Variation Difference > 0
Integrated Activity Variation Difference = Integrated Activity Variation at disparate landmarks minus Integrated Activity Variation at identical landmarks
Cue Cells
Grid Cells
B
Day 1
Day 7
Identical (I)
Disparate (D)
Day: ***
Landmark Pair Type: ***
Day × Landmark Pair Type: n.s.
Day 1 vs. 7: I: ***, D: ***
I vs. D: Day 1: ***, Day 7: ***
Integrated Activity Variation
Distance (cm)
Identical
Disparate
D
Day: n.s.
Landmark Pair Type: *
Day × Landmark Pair Type: n.s.
Day 1 vs. 7: I: n.s., D: n.s.
I vs. D: Day 1: n.s., Day 7: n.s.
p = 0.0649
Integral Activity Variation
C
E
Integral Activity Variation Difference
Comparison
F
Cue
Grid
Integral Activity Variation
G
H
Activity Range (cm)
Cue, Day 1
Cue, Day 7
Grid, Day 1
Grid, Day 7
7.8e-15
2.6e-10
8.5e-06
0.28
p-value
Increasing Significance
Mean Integral Activity Variation Difference
Summary
I
Stronger Encoding
Weaker Encoding
Landmark Information
Spatial Information (Location)
Nonspatial Information (Identity)
Grid Cells
Cue Cells

Figure R16. A. Schematic explaining integrated activity variation calculation. **B.** Integrated activity variation for identical (I, green) and disparate (D, magenta) landmarks at individual (left) and combined (right) distances on day 1 (thin lines) and day 7 (thick lines). Statistical tests in left panel are Bonferroni-corrected two-way ANOVAs. Statistics in right panel (top) are two-way ANOVAs. Post-hoc tests are Bonferroni-Holm-corrected two-tailed paired t-tests. **C.** Integrated activity variation difference for cue cells on day 1 and 7 at individual (left) and combined (right) distances. Statistical test in left panel is a two-way ANOVA. Statistical tests in right panel show Bonferroni-Holm-corrected two-tailed paired t-tests with zero as well as a two-tailed paired t-test comparing the two samples. **D-E.** Similar to B and C for putative grid cells. Statistical tests are the same as those in D-E. **F.** Comparison of Integrated activity variation in cue and putative grid cells for identical and disparate landmarks on day 1 and 7. Statistical tests are Bonferroni-Holm-corrected two-tailed non-paired t-tests. **G.** Integrated activity variation difference in cue and putative grid cells on day 1 and 7. Statistical tests are two-tailed non-paired t-tests. **H.** Mean integrated activity variation differences of cue cells and putative grid cells on days 1 and 7 as functions of activity range. p-value heatmap shows hotter colors for more significant paired t-tests relative to zero, obtained as in C and E with Bonferroni-Holm correction for each functional class's day 1 and 7 p-values. Putative grid cells on day 1 encode landmark identity only within small activity ranges. **I.** Summary schematic showing that both grid and cue cells are involved with the encoding of spatial and nonspatial information in the form of location and landmark identity, respectively, but to different degrees.

Cue cell data were from 5 mice, including 217 and 256 cells common to day 1 and day 7 of track 1 and track 2, respectively. Putative grid cell data were from 5 mice, including 132 and 118 cells common to day 1 and day 7 of track 1 and track 2, respectively.

Minor Comments:

1. Both the main and supplemental figure legends could benefit from clearer descriptions and explicit connections to the main text findings.

We have modified the figure legends for clarity and connection to the main text findings.

2. One thing unclear from the current analysis is, do the cue cells not reflect the landmark location at all? For example, in Figure 3B, right, the correlation of ~0.6 for identical landmarks is quite high, suggesting similar firing irrespective of location. But, based on Fig. 8E, even identical landmarks can be correctly decoded 80% of the time. Some estimate of the floor or ceiling levels of these decoding accuracies and rank correlations using shuffles would help clarify these points.

To estimate the ceiling effects for the rank correlation metric, we compare the true rank correlations of identical and disparate landmark pairs with three types of shuffles. Shuffle 1 randomly permuted the activity within each cell, abolishing activity differences across landmarks for that cell while preserving the relative activity levels between cells. Shuffle 2 permuted spatial activity across all cells within each FOV, abolishing both activity differences across landmarks within each cell and relative activity differences between cells. The last shuffle (Simulated) consisted of activity sampled from a normal distribution of ΔF/F values ranging from 0% to 100% (mean = 50%, standard deviation = 10%) (Fig. R17A).

Across tracks, identical landmarks exhibited higher rank correlations than Shuffle 1, whereas disparate landmarks showed the opposite trend (Fig. R17A). The floor level of shuffle 1 is above zero because, although cue cell activity varied between landmarks, the ranking across different

cue cells remains largely influenced by their intrinsic relative activity levels, resulting in a positive rank correlation (Fig. R17B). Importantly, the contrasting differences observed for identical and disparate landmarks compared to this shuffle strongly supports the encoding of landmark identity. Furthermore, removing the effect of intrinsic differences in activity between cells, as in shuffle 2, resulted in rank correlation close to zero, similar to those obtained from simulated cell activity, which is sampled from the same distribution for all cells (Fig. R17A).

Regarding the decoding result, we applied a run-by-run temporal shuffle of activity and compare the decoding of identical and disparate landmarks for cue cells on day 1 and day 7 for tracks 1 and 2 (Fig. R17C). Fixing a slight bug in our code resulted in decoding accuracies for identical landmark pairs being lower than ~80%. Nevertheless, shuffling activity reduced decoding accuracy to roughly 50% (i.e. chance), lower than the accuracy of both types of landmark pairs. The fact that identical landmarks were more accurately decoded suggests a role for cue cells in encoding landmark location. The higher decoding accuracy for identical compared to disparate landmarks supports cue cells' encoding for landmark identity.

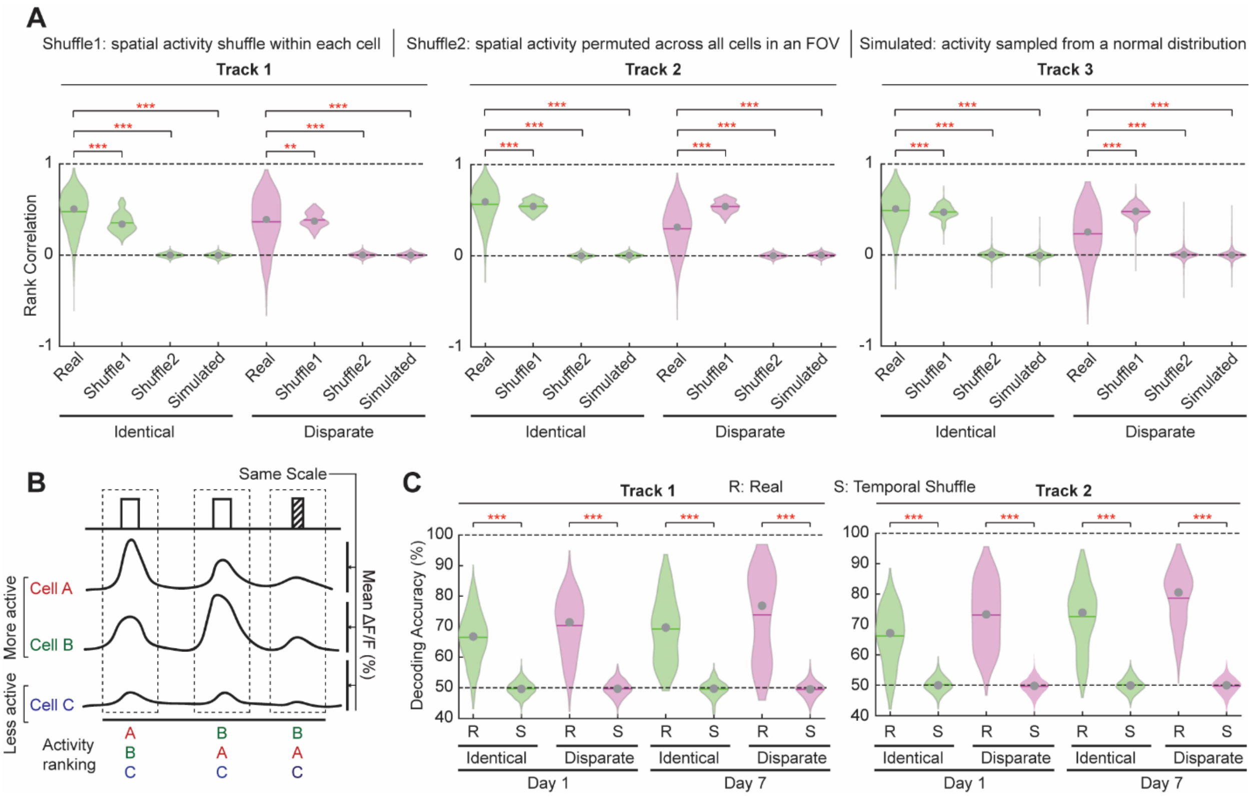

Figure R17 A. Rank correlation for identical and disparate landmark pairs in tracks 1, 2, and 3 for real as well as 100-times shuffled then averaged spatial activity (shuffle1: cells independently shuffled. Shuffle2: cell activity shuffled across all cells in each FOV. Simulated: randomly sampled activity data from a normal distribution). Statistical tests are two-tailed nonpaired t-tests. Bonferroni-Holm correction conducted within each track. **B.** Schematic of three cells' activity at landmarks at three landmarks, two of which have the same identity. Since cells A and B generally show higher activity (mean ΔF/F) than cell C, the ranking always has cell A and cell B in the top two positions. Therefore, the rank correlations between landmark pairs tend to show positive correlations. **C.** Decoding accuracy for identical and disparate landmark pairs of day 1 and 7 of tracks 1 and 2 for real as well as run-by-run temporally permuted activity. Statistical tests are the same

as in A. Data were from 5 mice. Rank correlation data were from 434, 465, and 508 cell groups for tracks 1, 2, and 3, respectively. For decoding accuracy data, 40 cue cells were randomly selected from each field of view 20 times and the data was aggregated.

3. Perhaps the population analysis in Figure 3 can be moved to be closer to Figure 8, as they are more similar conceptually.

Figure 3 presented data on cue cells, whereas Figure 8 focused on putative grid cells. We think Figure 3 is appropriately placed as it serves as a population-level follow-up to Figure 2, which demonstrates that individual cue cells encode landmark identity. In addition, the analysis of Figure 3 was used in figures 5 and 6.

We removed Figure 8 from the revised manuscript, as mentioned above.

5. I do not understand the results in Figure 2G. Cue cells were selected for their response across all landmarks, not a certain type of landmark. Why then, at the population level, is there a preferential response for landmark type 1? Surely that would average out across the population with distinct cells preferring different landmarks?

The response levels of cue cells to individual landmarks did not influence the selection of cue cells. To calculate the cue score, we first computed the correlation between each cue and the corresponding cue cell activity near it, then averaged these correlations across all cues[14]. This method ensures that responses to all individual landmarks contribute equally to the cue score. Therefore, cue cell classification is based solely on the locations of cue cell responses, rather than their relative magnitudes around individual cues.
Given this, the population data, which show differential responses to according to different landmark types, strongly support our finding that cue cells exhibit varying activity levels in response to landmark identity.

6. Why show the p-value in Supplementary Figure 2E, instead of showing the results themselves?

This is because the analysis includes many conditions, with the number of cells per group ranging from 2 to 20, and 50 sets of cue cell groups were created for each cell number condition.

Displaying all the results would result in an overwhelming number of figures. Therefore, we are only presenting the final statistics here.

7. I am also confused by Figure 5D. “Across tracks 1 and 2, the cells changed their activity variations at identical landmarks (Fig. 5D, the two green groups), but not at disparate ones (Fig. 5D, the two magenta groups).” Why would this be the case?

This means that across tracks 1 and 2, the cells did not equally change their responses to identical and disparate landmark pairs. Consequently, the differences in activity variations (i.e. activity variation difference) between these landmark pairs (response to disparate landmarks minus response to identical landmarks) were different across tracks 1 and 2. We clarify this point further in the manuscript (lines 395 to 397): “The different degrees of change in activity variation (Fig.

5B) resulted in distinct activity variation differences across the two tracks (Fig. 5C), reflecting variable levels of landmark identity encoding by the same cue cells in different tracks."

8. Lines 437-439: "Thus, the encodings of landmark identity and location by cue cells are differentially regulated by experience, suggesting different circuit mechanisms underlying these encodings," seems out of context and an overclaim given the results.

We have removed the specific claim about "different circuit mechanisms" or "different mechanisms" throughout the manuscript.

References

1. Chan, E., Baumann, O., Bellgrove, M.A., and Mattingley, J.B. (2012). From objects to landmarks: the function of visual location information in spatial navigation. Front Psychol *3*, 304. 10.3389/fpsyg.2012.00304.
2. Collett, T.S., Cartwright, B.A., and Smith, B.A. (1986). Landmark learning and visuo-spatial memories in gerbils. J Comp Physiol A *158*, 835-851. 10.1007/BF01324825.
3. Hurly, T.A., Fox, T.A., Zwueste, D.M., and Healy, S.D. (2014). Wild hummingbirds rely on landmarks not geometry when learning an array of flowers. Anim Cogn *17*, 1157-1165. 10.1007/s10071-014-0748-x.
4. Mumby, D.G., Gaskin, S., Glenn, M.J., Schramek, T.E., and Lehmann, H. (2002). Hippocampal damage and exploratory preferences in rats: memory for objects, places, and contexts. Learn Mem *9*, 49-57. 10.1101/lm.41302.
5. Oliveira, A.M., Hawk, J.D., Abel, T., and Havekes, R. (2010). Post-training reversible inactivation of the hippocampus enhances novel object recognition memory. Learn Mem *17*, 155-160. 10.1101/lm.1625310.
6. Barker, G.R., and Warburton, E.C. (2011). When is the hippocampus involved in recognition memory? J Neurosci *31*, 10721-10731. 10.1523/JNEUROSCI.6413-10.2011.
7. Assini, F.L., Duzzioni, M., and Takahashi, R.N. (2009). Object location memory in mice: pharmacological validation and further evidence of hippocampal CA1 participation. Behav Brain Res *204*, 206-211. 10.1016/j.bbr.2009.06.005.
8. Vogel-Ciernia, A., and Wood, M.A. (2014). Examining object location and object recognition memory in mice. Curr Protoc Neurosci *69*, 8 31 31-17. 10.1002/0471142301.ns0831s69.
9. Van Cauter, T., Camon, J., Alvernhe, A., Elduayen, C., Sargolini, F., and Save, E. (2013). Distinct roles of medial and lateral entorhinal cortex in spatial cognition. Cereb Cortex *23*, 451-459. 10.1093/cercor/bhs033.
10. Jeffery, K.J., and O'Keefe, J.M. (1999). Learned interaction of visual and idiothetic cues in the control of place field orientation. Exp Brain Res *127*, 151-161. 10.1007/s002210050785.
11. Jeffery, K.J. (1998). Learning of landmark stability and instability by hippocampal place cells. Neuropharmacology *37*, 677-687. 10.1016/s0028-3908(98)00053-7.
12. Deshmukh, S.S., and Knierim, J.J. (2013). Influence of local objects on hippocampal representations: Landmark vectors and memory. Hippocampus *23*, 253-267. 10.1002/hipo.22101.
13. Hoydal, O.A., Skytoen, E.R., Andersson, S.O., Moser, M.B., and Moser, E.I. (2019). Object-vector coding in the medial entorhinal cortex. Nature *568*, 400-404. 10.1038/s41586-019-1077-7.
14. Kinkhabwala, A.A., Gu, Y., Aronov, D., and Tank, D.W. (2020). Visual cue-related activity of cells in the medial entorhinal cortex during navigation in virtual reality. Elife *9*. 10.7554/eLife.43140.
15. Casali, G., Shipley, S., Dowell, C., Hayman, R., and Barry, C. (2018). Entorhinal Neurons Exhibit Cue Locking in Rodent VR. Front Cell Neurosci *12*, 512. 10.3389/fncel.2018.00512.
16. Nguyen, D., Wang, G., Wafa, T., Fitzgerald, T., and Gu, Y. (2024). The medial entorhinal cortex encodes multisensory spatial information. Cell Rep *43*, 114813. 10.1016/j.celrep.2024.114813.
17. Malone, T.J., Tien, N.W., Ma, Y., Cui, L., Lyu, S., Wang, G., Nguyen, D., Zhang, K., Myroshnychenko, M.V., Tyan, J., et al. (2024). A consistent map in the medial entorhinal cortex supports spatial memory. Nat Commun *15*, 1457. 10.1038/s41467-024-45853-4.

18. Deshmukh, S.S., and Knierim, J.J. (2011). Representation of non-spatial and spatial information in the lateral entorhinal cortex. Front Behav Neurosci *5*, 69. 10.3389/fnbeh.2011.00069.
19. Huang, X., Schlesiger, M.I., Barriuso-Ortega, I., Leibold, C., MacLaren, D.A.A., Bieber, N., and Monyer, H. (2023). Distinct spatial maps and multiple object codes in the lateral entorhinal cortex. Neuron *111*, 3068-3083 e3067. 10.1016/j.neuron.2023.06.020.
20. Yoganarasimha, D., Rao, G., and Knierim, J.J. (2011). Lateral entorhinal neurons are not spatially selective in cue-rich environments. Hippocampus *21*, 1363-1374. 10.1002/hipo.20839.
21. Hafting, T., Fyhn, M., Molden, S., Moser, M.B., and Moser, E.I. (2005). Microstructure of a spatial map in the entorhinal cortex. Nature *436*, 801-806. 10.1038/nature03721.
22. Sargolini, F., Fyhn, M., Hafting, T., McNaughton, B.L., Witter, M.P., Moser, M.B., and Moser, E.I. (2006). Conjunctive representation of position, direction, and velocity in entorhinal cortex. Science *312*, 758-762. 10.1126/science.1125572.
23. Solstad, T., Boccara, C.N., Kropff, E., Moser, M.B., and Moser, E.I. (2008). Representation of geometric borders in the entorhinal cortex. Science *322*, 1865-1868. 10.1126/science.1166466.

Revision Table 1: Statistical Information

**Figure R1**	
R1A (track 1)	Activity variation:<br>Two-tailed nonpaired t-test: $p = 0.29063$, $n_{Freq} = 2604$ activity variation values across all frequent-landmark distances and cells, $n_{Infreq} = 434$ activity variation values across all infrequent-landmark distances and cells<br><br>Rank correlation:<br>Two-tailed nonpaired t-test: $p = 9.8702e-11$, $n_{Freq} = 2604$ rank correlation values across all frequent-landmark distances and cell groups, $n_{Infreq} = 434$ rank correlation values across all infrequent-landmark distances and cell groups
R1B (track 2)	Activity variation:<br>Two-tailed nonpaired t-test: $p = 0.0018703$, $n_{Freq} = 2790$ activity variation values across all frequent-landmark distances and cells, $n_{Infreq} = 465$ activity variation values across all infrequent-landmark distances and cells<br><br>Rank correlation:<br>Two-tailed nonpaired t-test: $p = 2.5753e-09$, $n_{Freq} = 2790$ rank correlation values across all frequent-landmark distances and cell groups, $n_{Infreq} = 465$ rank correlation values across all infrequent-landmark distances and cell groups
R1C (aggregate of track 1 and 2)	Activity variation:<br>Two-tailed nonpaired t-test: $p = 0.15681$, $n_{Freq} = 5394$ activity variation values across all frequent-landmark distances and cells, $n_{Infreq} = 899$ activity variation values across all infrequent-landmark distances and cells<br><br>Rank correlation:<br>Two-tailed nonpaired t-test: $p = 0.70767$, $n_{Freq} = 5394$ rank correlation values across all frequent-landmark distances and cell groups, $n_{Infreq} = 899$ rank correlation values across all infrequent-landmark distances and cell groups

**Figure R2**	
R2C	Two-tailed non-paired t-tests comparing Identical (I) and Disparate (D) conditions (violin plots):<br><br>Track 4: $p = 2.9962e-06$, $n_I = 996$, $n_D = 3486$<br>Track 5: $p = 2.9578e-15$, $n_I = 1340$, $n_D = 8710$<br><br>Unit for all n is activity variation values across all appropriate distances and cells for either identical or disparate landmark pairs, as specified.
R2D	Two-tailed non-paired t-tests comparing Identical (I) and Disparate (D) conditions (violin plots):<br><br>Track 4: $p = 3.0577e-08$, $n_I = 996$, $n_D = 1992$<br>Track 5: $p = 2.5645e-11$, $n_I = 1340$, $n_D = 2680$<br><br>Unit for all n is activity variation values across all appropriate distances and cells for either identical or disparate landmark pairs, as specified.

R2E	Two-tailed non-paired t-tests comparing Identical (I) and Disparate (D) conditions (violin plots):<br><br>Track 4: p = 2.9482e-58, n_I = 808, n_D = 2828<br>Track 5: p = 3.8888e-138, n_I = 1340, n_D = 8710<br><br>Unit for all n is rank correlation values across all appropriate distances and cell groups for either identical or disparate landmark pairs, as specified.
R2F	Two-tailed non-paired t-tests comparing Identical (I) and Disparate (D) conditions (violin plots):<br><br>Track 4: p = 1.2003e-87, n_I = 808, n_D = 1616<br>Track 5: p = 3.2027e-95, n_I = 1340, n_D = 2680<br><br>Unit for all n is rank correlation values across all appropriate distances and cell groups for either identical or disparate landmark pairs, as specified.

**Figure R3**	
R3C	Unit for all n is activity variation values across all appropriate distances and cells for either identical or disparate landmark pairs, as specified.<br><br>Track 1:<br>Two-tailed Pearson's linear correlation between distance and mean activity variation:<br><br>Identical: r = -0.13751, p = 0.76875, 7 distances and associated mean activity variations<br>Disparate: r = -0.093998, p = 0.82479, 8 distances and associated mean activity variations<br><br>Two-tailed non-paired t-test (violin plot):<br>p = 5.098e-07, n_I = 3038, n_D = 3472<br><br>Track 2:<br>Two-tailed Pearson's linear correlation between distance and mean activity variation:<br>Identical: r = -0.21033, p = 0.65078, 7 distances and associated mean activity variations<br>Disparate: r = 0.46009, p = 0.25134, 8 distances and associated mean activity variations<br><br>Two-tailed non-paired t-test (violin plot):<br>p = 9.2716e-49, n_I = 3255, n_D = 3720

	Track 3:<br>Two-tailed Pearson's linear correlation between distance and mean activity variation:<br>Identical: r = 0.42929, p = 0.39563, 6 distances and associated mean activity variations<br>Disparate: r = -0.49236, p = 0.17815, 9 distances and associated mean activity variations<br><br>Two-tailed non-paired t-test (violin plot):<br>p = 1.2275e-44, n_I = 3048, n_D = 4572
R3D	Unit for all n is activity variation difference values across all appropriate distances and cells.<br><br>Track 1:<br>Two-tailed Pearson's linear correlation between distance and mean activity variation difference:<br>r = -0.24862, p = 0.84005, 3 distances and associated mean activity variation difference values<br><br>Two-tailed paired t-test compared to 0 (violin plot):<br>p = 0.0013558, n = 1302<br><br>Track 2:<br>Two-tailed Pearson's linear correlation between distance and mean activity variation difference:<br>r = 0.82684, p = 0.084218, 5 distances and associated mean activity variation difference values<br><br>Two-tailed paired t-test compared to 0 (violin plot):<br>p = 7.51e-45, n = 2325<br><br>Track 3:<br>Two-tailed Pearson's linear correlation between distance and mean activity variation difference:<br>r = -0.91641, p = 0.26215, 3 distances and associated mean activity variation difference values<br><br>Two-tailed paired t-test compared to 0 (violin plot):<br>p = 6.2312e-55, n = 1524
R3E	Unit for all n is rank correlation values across all appropriate distances and cell groups for either identical or disparate landmark pairs, as specified.<br><br>Track 1:<br>Two-tailed Pearson's linear correlation between distance and mean rank correlation:

	Identical: r = -0.0634, p = 0.89259, 7 distances and associated mean rank correlations<br>Disparate: r = -0.2012, p = 0.6328, 8 distances and associated mean rank correlations<br><br>Two-tailed non-paired t-test (violin plot):<br>p = 5.457e-33, n_I = 3038, n_D = 3472<br><br><br>Track 2:<br>Two-tailed Pearson's linear correlation between distance and mean rank correlation:<br>Identical: r = 0.060136, p = 0.89809, 7 distances and associated mean rank correlations<br>Disparate: r = -0.44872, p = 0.26476, 8 distances and associated mean rank correlations<br><br>Two-tailed non-paired t-test (violin plot):<br>p: reported as 0 by MATLAB ttest2 function, n_I = 3255, n_D = 3720<br><br><br>Track 3:<br>Two-tailed Pearson's linear correlation between distance and mean rank correlation:<br>Identical: r = -0.45101, p = 0.36936, 6 distances and associated mean rank correlations<br>Disparate: r = -0.12656, p = 0.74558, 9 distances and associated mean rank correlations<br><br>Two-tailed non-paired t-test (violin plot):<br>p = 6.2653e-187, n_I = 3048, n_D = 4572
R3F	Unit for all n is rank correlation difference values across all appropriate distances and cell groups.<br><br><br>Track 1:<br>Two-tailed Pearson's linear correlation between distance and mean rank correlation difference:<br>r = 0.52438, p = 0.6486, 3 distances and associated mean rank correlation difference values<br><br>Two-tailed paired t-test compared to 0 (violin plot):<br>p = 1.2426e-12, n = 1302<br><br><br>Track 2:<br>Two-tailed Pearson's linear correlation between distance and mean rank correlation difference:

	r = -0.68708, p = 0.19997, 5 distances and associated mean rank correlation difference values<br><br>Two-tailed paired t-test compared to 0 (violin plot):<br>p = 2.3941e-300, n = 2325<br><br>Track 3:<br>Two-tailed Pearson's linear correlation between distance and mean rank correlation difference:<br>r = 0.67403, p = 0.52913, 3 distances and associated mean rank correlation difference values<br><br>Two-tailed paired t-test compared to 0 (violin plot):<br>p = 2.2025e-146, n = 1524

**Figure R4**	
R4A	Two-tailed paired t-test: p = 1.8311e-09, n = 1736 activity variation values across four matched distances and all cells (for both violins)
R4B	Two-way ANOVA<br>Day: p = 3.1146e-11, F = 45.377<br>Landmark Pair Type: p = 1.3853e-07, F = 28.25<br>Day x Identity: p = 0.95845, F = 0.0027166<br><br>Two-tailed paired t-tests: Bonferroni-Holm Corrected<br><br>Each violin contains n = 868 activity variation values across four matched distances and all cells<br><br>I Day 1 vs I Day 7: p = 5.75e-07<br>D Track 1 vs D Day 7: p = 1.5883e-07<br>Day 1 I vs D: p = 4.3903e-05<br>Day 7 I vs D: p = 0.00015466

**Figure R5**	
R5A	Two-tailed Pearson's linear correlation of the day with the averaged activity variation difference on each day for all cells across all four matched distances:<br>r = -0.064, p = 0.892<br><br>Each day contained n = 636 activity variations or activity variation differences across 159 cells and 4 matched distances.
R5B	Two-tailed Pearson's linear correlation of the day with the averaged activity variation difference on each day for all cells across all four matched distances:<br>r = -0.380, p = 0.401<br><br>Each day contained n = 912 activity variations or activity variation differences across 228 cells and 4 matched distances.

R5C	Two-tailed Pearson's linear correlation of the day with the averaged rank correlation difference on each day for all cell groups across all four matched distances:<br>r = -0.256, p = 0.580<br><br>Each day contained n = 456 rank correlations or rank correlation differences across 114 cell groups and 4 matched distances.
R5D	Two-tailed Pearson's linear correlation of the day with the averaged rank correlation difference on each day for all cell groups across all four matched distances:<br>r = 0.024, p = 0.959<br><br>Each day contained n = 752 rank correlations or rank correlation differences across 188 cell groups and 4 matched distances.

**Figure R6**	
R6A	Unit for all n is activity variation values across all four matched distances and cells for either identical or disparate landmark pairs, as specified.<br><br><br>Track 1:<br>Line plot:<br>Two-way ANOVA: p = 0.089945, F = 2.8973, I: 148 cells x 4 matched distances; D: 148 cells x 4 matched distances<br><br>Violin plot:<br>Two-tailed paired t-test: p = 0.010993, n = 592 for I and D<br><br><br>Track 2:<br>Line plot:<br>Two-way ANOVA: p = 0.060544, F = 3.5589, I: 124 cells x 4 matched distances; D: 124 cells x 4 matched distances<br><br>Violin plot:<br>Two-tailed paired t-test: p = 0.00057004, n = 496 for I and D<br><br><br>Track 3:<br>Line plot:<br>Two-way ANOVA: p = 0.00015463, F = 14.754, I: 151 cells x 4 matched distances; D: 151 cells x 4 matched distances<br><br>Violin plot:<br>Two-tailed paired t-test: p = 1.3585e-10, n = 604 for I and D
R6B	Unit for all n is activity variation difference values across all four matched distances and cells.

	Track 1:<br>Two-tailed paired t-test with 0: p = 0.010993, n = 592<br><br>Track 2:<br>Two-tailed paired t-test with 0: p = 0.00057004, n = 496<br><br>Track 3:<br>Two-tailed paired t-test with 0: p = 1.3585e-10, n = 604
R6C	Unit for all n is rank correlation values across all four matched distances and cell groups for either identical or disparate landmark pairs, as specified.<br><br>Track 1:<br>Two-way ANOVA: p = 0.0037957, F = 8.531, I: 132 cell groups x 4 matched distances; D: 132 cell groups x 4 matched distances<br><br>Violin plot:<br>Two-tailed paired t-test: p = 0.00011245, n = 528 for I and D<br><br>Track 2:<br>Two-way ANOVA: p = 1.4902e-07, F = 30.227, I: 81 cell groups x 4 matched distances; D: 81 cell groups x 4 matched distances<br><br>Violin plot:<br>Two-tailed paired t-test: p = 5.0789e-11, n = 324 for I and D<br><br>Track 3:<br>Two-way ANOVA: p = 3.3361e-06, F = 23.031, I: 127 cell groups x 4 matched distances; D: 127 cell groups x 4 matched distances<br><br>Violin plot:<br>Two-tailed paired t-test: p = 1.1697e-13, n = 508 for I and D
R6D	Unit for all n is rank correlation difference values across all four matched distances and cell groups.<br><br>Track 1:<br>Two-tailed paired t-test with 0: p = 0.00011245, n = 528<br><br>Track 2:<br>Two-tailed paired t-test with 0: p = 5.0789e-11, n = 324<br><br>Track 3:<br>Two-tailed paired t-test with 0: p = 1.1697e-13, n = 508

**Figure R7**

R7A	Two-way ANOVA between identical and disparate curves with Bonferroni-Holm correction:<br><br>Before-Cue: p = 0.46809, F = 0.52957, I: 74 cells x 4 matched distances; D: 74 cells x 4 matched distances<br><br>At-Cue: p = 0.49512, F = 1.4076, I: 15 cells x 4 matched distances; D: 15 cells x 4 matched distances<br><br>After-Cue: p = 0.63296, F = 1.5855, I: 59 cells x 4 matched distances; D: 59 cells x 4 matched distances
R7B	Two-way ANOVA between identical and disparate curves with Bonferroni-Holm correction:<br><br>Before-Cue: p = 0.24233, F = 3.1175, I: 51 cells x 4 matched distances; D: 51 cells x 4 matched distances<br><br>At-Cue: p = 0.8337, F = 0.045888, I: 8 cells x 4 matched distances; D: 8 cells x 4 matched distances<br><br>After-Cue: p = 0.70472, F = 0.87225, I: 65 cells x 4 matched distances; D: 65 cells x 4 matched distances
R7C	Two-way ANOVA between identical and disparate curves with Bonferroni-Holm correction:<br><br>Before-Cue: p = 0.025194, F = 6.3817, I: 82 cells x 4 matched distances; D: 82 cells x 4 matched distances<br><br>At-Cue: p = 0.015193, F = 9.9085, I: 13 cells x 4 matched distances; D: 13 cells x 4 matched distances<br><br>After-Cue: p = 0.051329, F = 3.9069, I: 56 cells x 4 matched distances; D: 56 cells x 4 matched distances
R7D	Unit for all n is activity variation values across all four matched distances and appropriate cells for both identical and disparate landmark pairs, as specified.<br><br>Two-tailed paired t-tests comparing identical and disparate samples for each cell type (i.e. before, at, and after-cue cells). Bonferroni-Holm correction conducted for each track separately.<br><br>Track 1:<br>p_{Before} = 0.19666, n = 296 for both samples<br>p_{At} = 0.18765, n = 60 for both samples<br>p_{After} = 0.22992, n = 236 for both samples<br><br>Track 2:

	p_{Before} = 0.0032671, n = 204 for both samples<br>p_{At} = 0.80068, n = 32 for both samples<br>p_{After} = 0.12087, n = 260 for both samples<br><br>Track 3:<br>p_{Before} = 7.8307e-05, n = 328 for both samples<br>p_{At} = 0.00034089, n = 52 for both samples<br>p_{After} = 0.00024705, n = 224 for both samples
R7E	Unit for all n is activity variation difference values across all four matched distances and appropriate cells.<br><br>Two-way ANOVA considering effect of Track (p = 0.0349, F = 3.3841), Cell Type (p = 0.4839, F = 0.7272) and the join contribution of Track and Cell Type (p = 0.4084, F = 0.9980) on activity variation difference.<br><br>Track 1: Grouped By Bonferroni-Holm correction family of tests<br><br>n_{Before} = 74, n_{At} = 15, n_{After} = 59<br><br>Two-tailed non-paired t-tests:<br>$p_{Before\text{-}At}$ = 0.42164<br>$p_{At\text{-}After}$ = 0.56926<br>$p_{Before\text{-}After}$ = 0.51617<br><br>Two-tailed paired t-tests:<br>$p_{Before\text{-}0}$ = 0.094584<br>$p_{At\text{-}0}$ = 0.17958<br>$p_{After\text{-}0}$ = 0.063292<br><br>Track 2: Grouped By Bonferroni-Holm correction family of tests<br><br>n_{Before} = 51, n_{At} = 8, n_{After} = 65<br><br>Two-tailed non-paired t-tests:<br>$p_{Before\text{-}At}$ = 0.89304<br>$p_{At\text{-}After}$ = 0.74616<br>$p_{Before\text{-}After}$ = 0.74774<br><br>Two-tailed paired t-tests:<br>$p_{Before\text{-}0}$ = 0.012265<br>$p_{At\text{-}0}$ = 0.71531<br>$p_{After\text{-}0}$ = 0.044146<br><br>Track 3: Grouped By Bonferroni-Holm correction family of tests<br>n_{Before} = 82, n_{At} = 13, n_{After} = 56<br><br>Two-tailed non-paired t-tests:<br>$p_{Before\text{-}At}$ = 0.39339<br>$p_{At\text{-}After}$ = 0.28285

	$p_{Before-After}$ = 0.76006<br><br>Two-tailed paired t-tests:<br>$p_{Before-0}$ = 0.0029775<br>p_{At-0} = 0.0035554<br>$p_{After-0}$ = 0.0020871
R7F	All correlations below are two-tailed Pearson's linear correlations of spatial shifts which have at least 3 cells along with their associated averaged activity variation difference. The analysis was conducted separately for positive-shift and negative-shift cells.<br><br>Track 1:<br>Positive-shift cells' correlation: r = -0.583386, p = 0.128998<br>Negative-shift cells' correlation: r = -0.571155, p = 0.180459<br><br>Track 2:<br>Positive-shift cells' correlation: r = 0.216553, p = 0.680249<br>Negative-shift cells' correlation: r = -0.294973, p = 0.440969<br><br>Track 3:<br>Positive-shift cells' correlation: r = -0.841106, p = 0.00887188<br>Negative-shift cells' correlation: r = -0.58103, p = 0.171304<br><br>Two-tailed paired t-tests: The following p-values are Bonferroni-Holm corrected for all lags within each track. N/A listed for spatial shifts without enough cells for a two-tailed paired t-test calculation.

Spatial shifts (cm)	Track 1	Track 2	Track 3
35	1	N/A	0.7480
30	1	N/A	1
25	1	1	1
20	0.9178	0.9029	1
15	1	1	0.7463
10	1	0.5647	1
5	0.3265	1	0.3392
0	0.9877	1	0.0498
-5	1	1	1
-10	0.1878	0.0962	0.7389
-15	0.6962	1	0.7806
-20	1	1	0.4213
-25	1	1	1
-30	1	1	N/A
-35	N/A	1	1
-40	N/A	1	N/A
-45	N/A	1	N/A

**Figure R8**	
R8A (left)	Track 1 vs Track 2:

	Two-way ANOVA: Bonferroni-Holm Corrected<br><br>All curves: 144 cells x 4 distances<br><br>I Track 1 vs I track 2: p = 0.044275, F = 6.5426<br>D Track 1 vs D track 2: p = 0.18363, F = 1.777<br>Track 1 I vs D: p = 0.28356, F = 2.1713<br>Track 2 I vs D: p = 0.03569, F = 6.41<br><br>Track 1 vs Track 3:<br><br>Two-way ANOVA: Bonferroni-Holm Corrected<br><br>All curves: 164 cells x 4 distances<br><br>I Track 1 vs I track 3: p = 0.45111, F = 1.4748<br>D Track 1 vs D track 3: p = 0.60485, F = 0.26831<br>Track 1 I vs D: p = 0.021601, F = 7.3222<br>Track 3 I vs D: p = 8.1158e-05, F = 18.776<br><br>Track 2 vs Track 3:<br><br>Two-way ANOVA: Bonferroni-Holm Corrected<br><br>All curves: 154 cells x 4 distances<br><br>I Track 2 vs I track 3: p = 0.61435, F = 0.25445<br>D Track 2 vs D track 3: p = 0.0089135, F = 8.983<br>Track 2 I vs D: p = 0.13874, F = 2.2039<br>Track 3 I vs D: p = 0.00021555, F = 16.851
R8A (right)	Unit for all n is activity variation values across all four matched distances and cells.<br><br>Track 1 vs Track 2:<br><br>Two-way ANOVA<br>Track: p = 8.3767e-05, F = 15.708<br>Landmark Pair Type: p = 2.4828e-06, F = 22.658<br>Track x Landmark Pair Type: p = 0.24032, F = 1.3817<br><br>Two-tailed paired t-tests: Bonferroni-Holm Corrected<br><br>Each violin contains n = 576<br><br>I Track 1 vs I track 2: p = 0.00033866<br>D Track 1 vs D track 2: p = 0.028286

	Track 1 I vs D: p = 0.015891<br>Track 2 I vs D: p = 3.6009e-05<br><br><br><br>Track 1 vs Track 3:<br><br>Two-way ANOVA<br>Track: p = 0.62414, F = 0.24035<br>Landmark Pair Type: p = 3.7299e-10, F = 40.659<br>Track x Landmark Pair Type: p = 0.026996, F = 4.9163<br><br>Two-tailed paired t-tests: Bonferroni-Holm Corrected<br><br>Each violin contains n = 656<br><br>I Track 1 vs I track 3: p = 0.10039<br>D Track 1 vs D track 3: p = 0.24926<br>Track 1 I vs D: p = 0.0024328<br>Track 3 I vs D: p = 3.8753e-09<br><br><br><br>Track 2 vs Track 3:<br><br>Two-way ANOVA<br>Track: p = 0.0001663, F = 14.378<br>Landmark Pair Type: p = 6.3474e-10, F = 39.623<br>Track x Landmark Pair Type: p = 0.023582, F = 5.1541<br><br>Two-tailed paired t-tests: Bonferroni-Holm Corrected<br><br>Each violin contains n = 616<br><br>I Track 2 vs I track 3: p = 0.2181<br>D Track 2 vs D track 3: p = 8.5206e-05<br>Track 2 I vs D: p = 0.001363<br>Track 3 I vs D: p = 2.7315e-08
R8B	Unit for all n is change in activity variation values across all four matched distances and cells.<br><br>Track 1 vs Track 2:<br><br>Each curve contains 144 cells x 4 distances.<br><br>p = 0.45852, F = 0.55113<br>Two-tailed paired t-test: p = 0.24032, n = 576 for both violins<br><br><br><br>Track 1 vs Track 3:

	Each curve contains 164 cells x 4 distances.<br><br>p = 0.093308, F = 2.8365<br>Two-tailed paired t-test: p = 0.026996, n = 656 for both violins<br><br>Track 2 vs Track 3:<br><br>Each curve contains 154 cells x 4 distances.<br><br>p = 0.066786, F = 3.3891<br><br>Two-tailed paired t-test: p = 0.023582, n = 616 for both violins
R8C	Unit for all n is activity variation difference values across all four matched distances and cells.<br><br>Track 1 vs Track 2:<br><br>Two-way ANOVA:<br>Each curve contains 144 cells x 4 distances.<br><br>p = 0.061879, F = 3.5161<br><br>Two-tailed paired t-test:<br>p = 0.075604, n = 576 for both violins<br><br>Track 1 vs Track 3:<br><br>Two-way ANOVA:<br>Each curve contains 164 cells x 4 distances.<br><br>p = 0.2798, F = 1.1727<br><br>Two-tailed paired t-test:<br>p = 0.064409, n = 656 for both violins<br><br>Track 2 vs Track 3:<br><br>Two-way ANOVA:<br>Each curve contains 154 cells x 4 distances.<br><br>p = 0.057321, F = 3.6438

	Two-tailed paired t-test:<br>p = 0.082873, n = 616 for both violins
R8D	Each statistical test is a two-tailed paired t-test comparison:<br>Track 1 vs 2: p = 0.12364, n = 144 cells<br>Track 1 vs 3: p = 0.14916, n = 164 cells<br>Track 2 vs 3: p = 0.14866, n = 154 cells

**Figure R9**	
R9A (left)	Track 1 vs Track 2:<br><br>Two-way ANOVA: Bonferroni-Holm Corrected<br><br>All curves: 99 cell groups x 4 distances<br><br>I Track 1 vs I track 2: p = 0.015164, F = 8.5854<br>D Track 1 vs D track 2: p = 0.96361, F = 0.49667<br>Track 1 I vs D: p = 0.22134, F = 3.2313<br>Track 2 I vs D: p = 0.82881, F = 0.04688<br><br>Track 1 vs Track 3:<br><br>Two-way ANOVA: Bonferroni-Holm Corrected<br><br>All curves: 153 cell groups x 4 distances<br><br>I Track 1 vs I track 3: p = 0.059017, F = 4.7827<br>D Track 1 vs D track 3: p = 0.25609, F = 1.2946<br>Track 1 I vs D: p = 2.3461e-07, F = 30.93<br>Track 3 I vs D: p = 0.0046584, F = 10.199<br><br>Track 2 vs Track 3:<br><br>Two-way ANOVA: Bonferroni-Holm Corrected<br><br>All curves: 121 cell groups x 4 distances<br><br>I Track 2 vs I track 3: p = 6.3103e-27, F = 153.1<br>D Track 2 vs D track 3: p = 0.34327, F = 0.90175<br>Track 2 I vs D: p = 1.8742e-21, F = 113.35<br>Track 3 I vs D: p = 0.55026, F = 1.1965
R9A (right)	Unit for all n is rank correlation values across all four matched distances and cell groups.

Track 1 vs Track 2:

Two-way ANOVA
Track: p = 2.1948e-09, F = 37.514
Landmark Pair Type: p = 0.0071981, F = 7.2986
Track x Landmark Pair Type: p = 0.050729, F = 3.8406

Two-tailed paired t-tests: Bonferroni-Holm Corrected

Each violin contains n = 396

I Track 1 vs I track 2: p = 6.9246e-09
D Track 1 vs D track 2: p = 0.63775
Track 1 I vs D: p = 0.0032737
Track 2 I vs D: p = 0.75826

Track 1 vs Track 3:

Two-way ANOVA
Track: p = 0.16727, F = 1.9118
Landmark Pair Type: p = 2.8574e-10, F = 41.127
Track x Landmark Pair Type: p = 2.0497e-05, F = 18.43

Two-tailed paired t-tests: Bonferroni-Holm Corrected

Each violin contains n = 612

I Track 1 vs I track 3: p = 8.5623e-06
D Track 1 vs D track 3: p = 0.056482
Track 1 I vs D: p = 1.1686e-14
Track 3 I vs D: p = 0.001585

Track 2 vs Track 3:

Two-way ANOVA
Track: p = 1.1081e-57, F = 338.52
Landmark Pair Type: p = 7.9543e-17, F = 74.74
Track x Landmark Pair Type: p = 4.3281e-21, F = 97.618

Two-tailed paired t-tests: Bonferroni-Holm Corrected

Each violin contains n = 121

I Track 2 vs I track 3: p = 5.8814e-69
D Track 2 vs D track 3: p = 0.12077
Track 2 I vs D: p = 4.6984e-41
Track 3 I vs D: p = 0.17595

R9B	Unit for all n is change in rank correlation values across all four matched distances and cell groups.<br><br>Track 1 vs Track 2:<br><br>Each curve contains 99 cell groups x 4 distances.<br><br>p = 0.0082755, F = 7.1168<br>Two-tailed paired t-test: p = 0.050729, n = 396 for both violins<br><br>Track 1 vs Track 3:<br><br>Each curve contains 153 cell groups x 4 distances.<br><br>p = 1.3073e-06, F = 24.379<br>Two-tailed paired t-test: p = 2.0497e-05, n = 612 for both violins<br><br>Track 2 vs Track 3:<br><br>Each curve contains 121 cell groups x 4 distances.<br><br>p = 2.7381e-26, F = 143.92<br><br>Two-tailed paired t-test:<br>p = 4.3281e-21, n = 484 for both violins
R9C	Unit for all n is rank correlation difference values across all four matched distances and cell groups.<br><br>Track 1 vs Track 2:<br><br>Two-way ANOVA:<br>Each curve contains 99 cell groups x 4 distances.<br><br>p = 2.5417e-18, F = 93.437<br><br>Two-tailed paired t-test:<br>p = 2.6056e-17, n = 396 for both violins<br><br>Track 1 vs Track 3:<br><br>Two-way ANOVA:<br>Each curve contains 153 cell groups x 4 distances.

	p = 0.00025295, F = 13.712<br><br><br>Two-tailed paired t-test:<br>p = 1.3058e-05, n = 612 for both violins<br><br><br>Track 2 vs Track 3:<br><br>Two-way ANOVA:<br>Each curve contains 121 cell groups x 4 distances.<br><br>p = 1.2826e-45, F = 315.37<br><br><br>Two-tailed paired t-test:<br>p = 6.1135e-44, n = 484 for both violins
R9D	Each statistical test is a two-tailed paired t-test comparison:<br>Track 1 vs 2: p = 2.3376e-13, n = 99 cell groups<br>Track 1 vs 3: p = 0.00015223, n = 153 cell groups<br>Track 2 vs 3: p = 5.7541e-30, n = 121 cell groups

**Figure R10**	
R10A (left panels)	Track 1:<br>Two-way ANOVA: Bonferroni-Holm Corrected<br>All curves: 105 cells x 4 distances<br><br>I Day 1 vs I Day 7: p = 0.0056359, F = 10.522<br>D Day 1 vs D Day 7: 0.049054, F = 5.1412<br>Day 1 I vs D: p = 0.060019, F = 5.5023<br>Day 7 I vs D: p = 0.15636, F = 2.0271<br><br><br>Track 2:<br>Two-way ANOVA: Bonferroni-Holm Corrected<br>All curves: 115 cells x 4 distances<br><br>I Day 1 vs I Day 7: p = 0.043014, F = 4.1447<br>D Day 1 vs D Day 7: p = 0.071284, F = 4.4705<br>Day 1 I vs D: p = 0.030422, F = 6.7269<br>Day 7 I vs D: p = 0.01454, F = 8.6542
R10A (right panels)	Unit for all n is activity variation values across all four matched distances and cells.<br><br><br>Track 1:<br><br>Two-way ANOVA<br>Day: p = 2.7823e-09, F = 37.198

	Landmark Pair Type: p = 0.00069838, F = 11.698<br>Day x Identity: p = 0.17802, F = 1.8213<br><br>Two-tailed paired t-tests: Bonferroni-Holm Corrected<br><br>Each violin contains n = 420<br><br>I Day 1 vs I Day 7: p = 8.1776e-08<br>D Track 1 vs D Day 7: p = 0.00013349<br>Day 1 I vs D: p = 0.0010493<br>Day 7 I vs D: p = 0.080671<br><br>Track 2:<br><br>Two-way ANOVA<br>Day: p = 1.35e-06, F = 24.035<br>Landmark pair type: p = 1.0292e-10, F = 43.945<br>Day x Landmark pair type: p = 0.83708, F = 0.042336<br><br>Two-tailed paired t-tests: Bonferroni-Holm Corrected<br><br>Each violin contains n = 460<br><br>I Day 1 vs I Day 7: p = 0.00013711<br>D Track 1 vs D Day 7: p = 0.00024958<br>Day 1 I vs D: p = 4.0215e-06<br>Day 7 I vs D: p = 3.2181e-06
R10B (left panels)	Track 1:<br>Two-way ANOVA: Both curves: 105 cells x 4 distances<br>p = 0.64472, F = 0.21345<br><br>Track 2:<br>Two-way ANOVA: Both curves: 115 cells x 4 distances<br>p = 0.76402, F = 0.090372
R10B (right panels)	Unit for all n is change in activity variation values across all four matched distances and cells.<br><br>Track 1:<br>Two-tailed paired t-test: Each violin contains n = 420<br>p = 0.17802<br><br>Track 2:<br>Two-tailed paired t-test: Each violin contains n = 460<br>p = 0.83708
R10C (left panels)	Track 1: Two-way ANOVA<br>Both curves: 105 cells x 4 distances<br>Day 1 vs Day 7: p = 0.2952, F = 1.1026<br><br>Track 2: Two-way ANOVA:<br>Both curves: 115 cells x 4 distances<br>Day 1 vs Day 7: p = 0.9622, F = 0.0022519

R10C (right panels)	Unit for all n is change in activity variation values across all four matched distances and cells.<br><br>Track 1: Two-tailed paired t-tests<br>p = 0.17802, n = 420 for both violins<br><br>Track 2: Two-tailed paired t-tests:<br>p = 0.83708, n = 460 for both violins
R10D	Each statistical test is a two-tailed paired t-test comparison:<br>Track 1: p = 0.4188, n = 105 cells<br>Track 2: p = 0.6115, n = 115 cells

**Figure R11**	
R11A (left panels)	Track 1:<br>Two-way ANOVA: Bonferroni-Holm Corrected<br>All curves: 91 cell groups x 4 distances<br><br>I Day 1 vs I Day 7: p = 6.839e-15, F = 76.207<br>D Day 1 vs D Day 7: 1.7626e-08, F = 34.814<br>Day 1 I vs D: p = 4.1245e-09, F = 39.867<br>Day 7 I vs D: p = 5.9452e-11, F = 51.267<br><br>Track 2:<br>Two-way ANOVA: Bonferroni-Holm Corrected<br>All curves: 87 cell groups x 4 distances<br><br>I Day 1 vs I Day 7: p = 0.018604, F = 6.9191<br>D Day 1 vs D Day 7: p = 0.67489, F = 0.17654<br>Day 1 I vs D: p = 6.0477e-20, F = 112.55<br>Day 7 I vs D: p = 4.6749e-12, F = 58.181
R11A (right panels)	Unit for all n is rank correlation values across all four matched distances and cell groups.<br><br>Track 1:<br><br>Two-way ANOVA<br>Day: p = 6.068e-25, F = 123.81<br>Landmark Pair Type: p = 1.5585e-33, F = 179.47<br>Day x Identity: p = 0.62932, F = 0.23338<br><br>Two-tailed paired t-tests: Bonferroni-Holm Corrected<br><br>Each violin contains n = 364<br><br>I Day 1 vs I Day 7: p = 5.8464e-14<br>D Track 1 vs D Day 7: p = 5.3114e-14<br>Day 1 I vs D: p = 3.431e-15

	Day 7 I vs D: p = 8.1924e-16<br><br>Track 2:<br><br>Two-way ANOVA<br>Day: p = 0.0016722, F = 10.035<br>Landmark pair type: p = 2.9261e-67, F = 478.28<br>Day x Landmark pair type: p = 0.070775, F = 3.2851<br><br>Two-tailed paired t-tests: Bonferroni-Holm Corrected<br><br>Each violin contains n = 348<br><br>I Day 1 vs I Day 7: p = 3.7017e-05<br>D Track 1 vs D Day 7: p = 0.45807<br>Day 1 I vs D: p = 9.9783e-45<br>Day 7 I vs D: p = 1.2542e-35
R11B (left panels)	Track 1:<br>Two-way ANOVA: Both curves: 91 cell groups x 4 distances<br>p = 0.67563, F = 0.17566<br><br>Track 2:<br>Two-way ANOVA: Both curves: 87 cell groups x 4 distances<br>p = 0.16982, F = 1.9004
R11B (right panels)	Unit for all n is change in rank correlation values across all four matched distances and cell groups.<br><br>Track 1:<br>Two-tailed paired t-test: Each violin contains n = 364<br>p = 0.62932<br><br>Track 2:<br>Two-tailed paired t-test: Each violin contains n = 348<br>p = 0.070775
R11C (left panels)	Track 1: Two-way ANOVA<br>Both curves: 91 cell groups x 4 distances<br>Day 1 vs Day 7: p = 0.54813, F = 0.36205<br><br>Track 2: Two-way ANOVA:<br>Both curves: 87 cell groups x 4 distances<br>Day 1 vs Day 7: p = 0.20574, F = 1.6133
R11C (right panels)	Unit for all n is rank correlation difference values across all four matched distances and cell groups.<br><br>Track 1: Two-tailed paired t-tests<br>p = 0.62932, n = 364 for both violins<br><br>Track 2: Two-tailed paired t-tests:<br>p = 0.070775, n = 348 for both violins
R11D	Each statistical test is a two-tailed paired t-test comparison:

	Track 1: p = 0.43079, n = 91 cell groups<br>Track 2: p = 0.2333, n = 87 groups

**Figure R13**	
R13A	Each dot represents the p-value resulting from a two-tailed paired t-test comparing the rank correlation difference per cell group with 0. Shown for tracks 1, 2, and 3.

**Figure R14**	
R14A	Rank correlations listed at each landmark are the two-tailed Pearson's linear correlations of each landmark's ranking of the same 60 cells compared to the reference landmark.

**Figure R15**	
R15B	Two-tailed Kolmogorov-Smirnov test between the field center distribution and a uniform distribution: p = 0.72995.
R15G (top)	Two-tailed Kolmogorov-Smirnov test between the field center distribution and a uniform distribution: p = 0.732.
R15G (bottom)	Percentile (p) of the original day 7-10 field center fraction toward the fractions of the 20000 simulated normal distributions. p < 5% indicates the original field center fraction is below the bottom 5% of the simulated distribution's left tail, while p > 95% indicates the original field center fraction is above the top 5% of the simulated distribution's right tail. Both cases are regarded as significant (labeled with an asterisk).

Percentage of distance from the back landmark	Percentile (p)
-140% to -120%:	p=75%
-120% to -100%	p= 15 %
-100% to -80%	p=0.01%
-80% to -60%	p=0%
-60% to -40%	p=0%
-40% to -20%	p=0%
-20% to 0%	p=0%
0% to 20%	p=100%
20% to 40%	p=100%
40% to 60%	p=100%
60% to 80%	p=100%
80% to 100%	p=100%
100% to 120%	p=100%

**Figure R16**	
R16B (left)	Two-way ANOVA: Bonferroni-Holm Corrected<br><br>All curves: 473 cells x 4 distances<br><br>I Day 1 vs I Day 7: p = 7.44497e-06, F = 21.668

	D Day 1 vs D Day 7: p = 1.50895e-05, F = 18.928<br>Day 1 I vs D: p = 9.26712e-06, F = 22.594<br>Day 7 I vs D: p = 7.99049e-06, F = 22.33
R16B (right)	Two-way ANOVA<br>Day: p = 2.2793e-26, F = 116.49<br>Identity: p = 1.8139e-23, F = 102.46<br>Day x Identity: p = 0.88478, F = 0.021004<br><br>Two-tailed paired t-tests: Bonferroni-Holm Corrected<br><br>Each violin contains n = 1892 integrated activity variation values across all four matched distances and cells<br><br>I Day 1 vs I Day 7: p = 4.6109e-17<br>D Track 1 vs D Day 7: p = 1.6069e-15<br>Day 1 I vs D: p = 7.84076e-15<br>Day 7 I vs D: p = 5.35323e-13
R16C (left)	Two-way ANOVA:<br>Both curves: 437 cells x 4 distances<br>p = 0.94423, F = 0.0048962
R16C (right)	Two-tailed paired t-test comparing integrated activity variation difference on day 1 vs day 7: $p_{day1, day7}$ = 0.884784<br><br>Two-tailed paired t-test comparing integrated activity variation difference with 0 for both day 1 and day 7 condition, Bonferroni-Holm corrected:<br><br>$p_{day1, 0}$ = 7.84076e-15<br>$p_{day7, 0}$ = 5.35323e-13<br><br>Each violin contains n = 1892 integrated activity variation difference values across all four matched distances and cells.
R16D (left)	Two-way ANOVA: Bonferroni-Holm Corrected<br><br>All curves: 250 cells x 4 distances<br><br>I Day 1 vs I Day 7: p = 0.773099, F = 0.75112<br>D Day 1 vs D Day 7: p = 0.890309, F = 0.01904<br>Day 1 I vs D: p = 0.234035, F = 3.5958<br>Day 7 I vs D: p = 0.812703, F = 1.215
R16D (right)	Two-way ANOVA<br>Day: p = 0.17029, F = 1.8832<br>Landmark Pair Type: p = 0.011076, F = 6.4777<br>Day x Landmark Pair Type: p = 0.5177, F = 0.41878<br><br>Two-tailed paired t-tests: Bonferroni-Holm Corrected<br><br>Each violin contains n = 1000 integrated activity variation values across all four matched distances and cells<br><br>I Day 1 vs I Day 7: p = 0.372148

<table>
<tr><td></td><td>D Track 1 vs D Day 7: p = 0.539898
Day 1 I vs D: p = 0.0648758
Day 7 I vs D: p = 0.332728</td></tr>
<tr><td>R16E (left)</td><td>Two-way ANOVA:
Both curves: 250 cells x 4 distances
p = 0.45657, F = 0.55518</td></tr>
<tr><td>R16E (right)</td><td>Two-tailed paired t-test comparing integrated activity variation difference on day 1 vs day 7: $p_{day1, day7}$ = 0.517697

Two-tailed paired t-test comparing integrated activity variation difference with 0 for both day 1 and day 7 condition, Bonferroni-Holm corrected:

$p_{day1, 0}$ = 0.0324379
$p_{day7, 0}$ = 0.166364

Each violin contains n = 1000 integrated activity variation difference values across all four matched distances and cells.</td></tr>
<tr><td>R16F</td><td>Unit for n is integrated activity variation values across all four matched distances and cells.

Each violin for cue cells contains n = 1892.
Each violin for putative grid cells contains n = 1000.

Two-tailed non-paired t-tests: Bonferroni-Holm corrected

Day 1 Identical Cue vs Putative Grid: p = 1.04762e-15
Day 1 Disparate Cue vs Putative Grid: p = 1.77083e-05
Day 7 Identical Cue vs Putative Grid: p = 0.00333082
Day 7 Disparate Cue vs Putative Grid: p = 0.338633</td></tr>
<tr><td>R16G</td><td>Unit for n is integrated activity variation difference values across all four matched distances and cells.

Each violin for cue cells contains n = 1892.
Each violin for putative grid cells contains n = 1000.

Two-tailed non-paired t-tests comparing cue and putative grid cells: Bonferroni-Holm corrected

Day 1 Cue vs Putative Grid: 0.0117133
Day 7 Cue vs Putative Grid: 0.00588861</td></tr>
<tr><td>R16H</td><td>Two-tailed paired t-test comparing integrated activity variation difference with 0 for both day 1 and day 7 condition, independently Bonferroni-Holm corrected for each activity range and functional class (i.e. as in Fig. 7C (right) and Fig. 7E (right)).

Cue:
<table>
<tr><td>Activity range (cm)</td><td>p-value, day 1</td><td>p-value, day 7</td></tr>
<tr><td>5</td><td>1.1684e-11</td><td>7.3127e-07</td></tr>
<tr><td>10</td><td>7.5601e-13</td><td>5.5165e-10</td></tr>
<tr><td>15</td><td>4.8072e-13</td><td>1.2703e-11</td></tr>
<tr><td>20</td><td>1.6466e-14</td><td>1.4316e-12</td></tr>
</table></td></tr>
</table>

25	7.8408e-15	5.3532e-13
30	2.1574e-14	4.8219e-13
35	3.0084e-14	5.5234e-14
40	1.4262e-14	3.2451e-14
45	1.1919e-14	7.2625e-14
50	3.4445e-14	3.0239e-14

Putative Grid:

Activity range (cm)	p-value, day 1	p-value, day 7
5	0.0254	0.2499
10	0.0337	0.0766
15	0.0308	0.1787
20	0.0253	0.2790
25	0.0324	0.1664
30	0.0915	0.1443
35	0.1927	0.1133
40	0.1349	0.1490
45	0.1255	0.1818
50	0.1489	0.1853

Figure R17

R17A

In all cases, Bonferroni-Holm p-value correction is conducted separately for each track.

Track 1:

Two-tailed nonpaired t-tests: n = 1736 rank correlation values across all four matched distances and cell groups

I Real vs I Shuffle1: p = 1.7978e-90
I Real vs I Shuffle2: p reported as 0 by MATLAB ttest2 function
I Real vs I Simulated: p reported as 0 by MATLAB ttest2 function

D Real vs D Shuffle1: p = 0.0048289
D Real vs D Shuffle2: p reported as 0 by MATLAB ttest2 function
D Real vs D Simulated: p reported as 0 by MATLAB ttest2 function

Track 2:

Two-tailed nonpaired t-tests: n = 1860 rank correlation values across all four matched distances and cell groups

I Real vs I Shuffle1: p = 5.481e-06
I Real vs I Shuffle2: p reported as 0 by MATLAB ttest2 function
I Real vs I Simulated: p reported as 0 by MATLAB ttest2 function

	D Real vs D Shuffle1: p = 4.9058e-298<br>D Real vs D Shuffle2: p reported as 0 by MATLAB ttest2 function<br>D Real vs D Simulated: p reported as 0 by MATLAB ttest2 function<br><br>Track 3:<br><br>Two-tailed nonpaired t-tests: n = 2032 rank correlation values across all four matched distances and cell groups<br><br>I Real vs I Shuffle1: p = 0.00087871<br>I Real vs I Shuffle2: p reported as 0 by MATLAB ttest2 function<br>I Real vs I Simulated: p reported as 0 by MATLAB ttest2 function<br><br>D Real vs D Shuffle1: p = 1.0867e-241<br>D Real vs D Shuffle2: p = 8.8073e-227<br>D Real vs D Simulated: p = 1.3162e-233
R17C	In all cases, Bonferroni-Holm p-value correction is conducted separately for each track. Units for n are cue cell selections.<br><br>Track 1: Two-tailed nonpaired t-tests<br><br>Day 1 I Real vs Shuffle: p reported as 0 by MATLAB ttest2 function, n = 800<br>Day 1 D Real vs Shuffle: p reported as 0 by MATLAB ttest2 function, n = 800<br>Day 7 I Real vs Shuffle: p = 1.1814e-206, n = 480<br>Day 7 D Real vs Shuffle: p = 1.362e-191, n = 480<br><br>Track 2: Two-tailed nonpaired t-tests<br><br>Day 1 I Real vs Shuffle: p = 3.2029e-301, n = 800<br>Day 1 D Real vs Shuffle: p reported as 0 by MATLAB ttest2 function, n = 800<br>Day 7 I Real vs Shuffle: p reported as 0 by MATLAB ttest2 function, n = 720<br>Day 7 D Real vs Shuffle: p reported as 0 by MATLAB ttest2 function, n = 720

We sincerely thank the reviewers for their positive feedback on our revised manuscript and the editor for providing us with the opportunity to further improve it.

To address the additional comments, we have conducted new analyses and revised the manuscript accordingly:

1. We revised the **Discussion** section to address the comment from Reviewer #1.
2. We performed additional analyses to respond to the comment from Reviewer #2, as highlighted by the editor. All the analyses have been included in **Results** section of the revised manuscript.
3. We improved the text in both the **Results** and **Discussion** sections following the suggestions of Reviewer #3.

Below, we provide our detailed responses to all reviewer comments. Our responses are in **black text**, while the reviewer comments are shown in **blue text**.

Reviewer #1:

I'm overall satisfied with the authors' response. However, I strongly suggest that the authors add additional discussion regarding the difference and relationship between the coding of landmark identity and the previously reported rate remapping in MEC.

We appreciated the reviewer's suggestion and now added the following discussions to lines 625-637.

"Previous studies also found that grid cells exhibit object-selective activity when the objects are associated with reward[1], display rate remapping when environmental features such as shape and color are altered[2], and undergo global remapping in environments that are spatially similar but defined by cues from different sensory modalities[3]. While these forms of remapping occur across distinct environments and likely contribute to context encoding, the activity variations we observe near landmarks occur within a single environment and closely track landmark identity in novel environment. Together, these results suggest that grid cells not only present spatial information but also incorporate nonspatial environmental information into their cognitive maps. Interestingly, whereas changes in environmental shape and color induced only rate remapping in grid cells, nongrid cells showed a complete reorganization of their firing patterns. This further suggests that nongrid cells may distinguish environmental features more robustly, consistent with the stronger landmark-identity encoding by cue cells than by grid cells reported here."

Reviewer #2 (requested by the editor)

In particular, we will need to see new analyses added to fully address the concern regarding lack of justification for use of 80th percentile as the cut-off for cue cells across multiple sessions from reviewer 2.

The original comment of reviewer #2 regarding this issue: What is the justification for use of 80th percentile as the cut-off for cue cells across multiple sessions as opposed to 95th or a larger percentile?

We thank the editor for bringing up this issue. We used an 80th percentile threshold to classify cue cells across multiple sessions (across different tracks and across days within the same track) because this criterion yielded a larger population of cue cells than the 95th percentile threshold, thereby providing sufficient statistical power for group-level analyses (Figs. SR1A and SR3A).

Importantly, cue cells identified using the 80th percentile threshold retained strong landmark-associated activity across sessions (Fig. SR1B-D and SR3B-C). They exhibited largely correlated spatial shifts across environments (Fig. S11B in the manuscript), as reported previously for cue cells classified by the 95th percentile threshold[4]. Their spatial shifts were also strongly correlated within the same environment (Fig. S11E). These results indicate that cue cells classified using the 80th percentile threshold largely preserved cue cell properties.

In addition, we repeated the cross-session analyses using cue cells defined by the more stringent 95th percentile threshold. Despite the smaller sample size, leading to reduced statistical power and some nonsignificant effects, the main findings were recapitulated: cue cells exhibited distinct landmark identity encoding across different environments (Fig. SR2) while maintaining stable encoding within the same environment, independent of experience (Fig. SR3D-I).

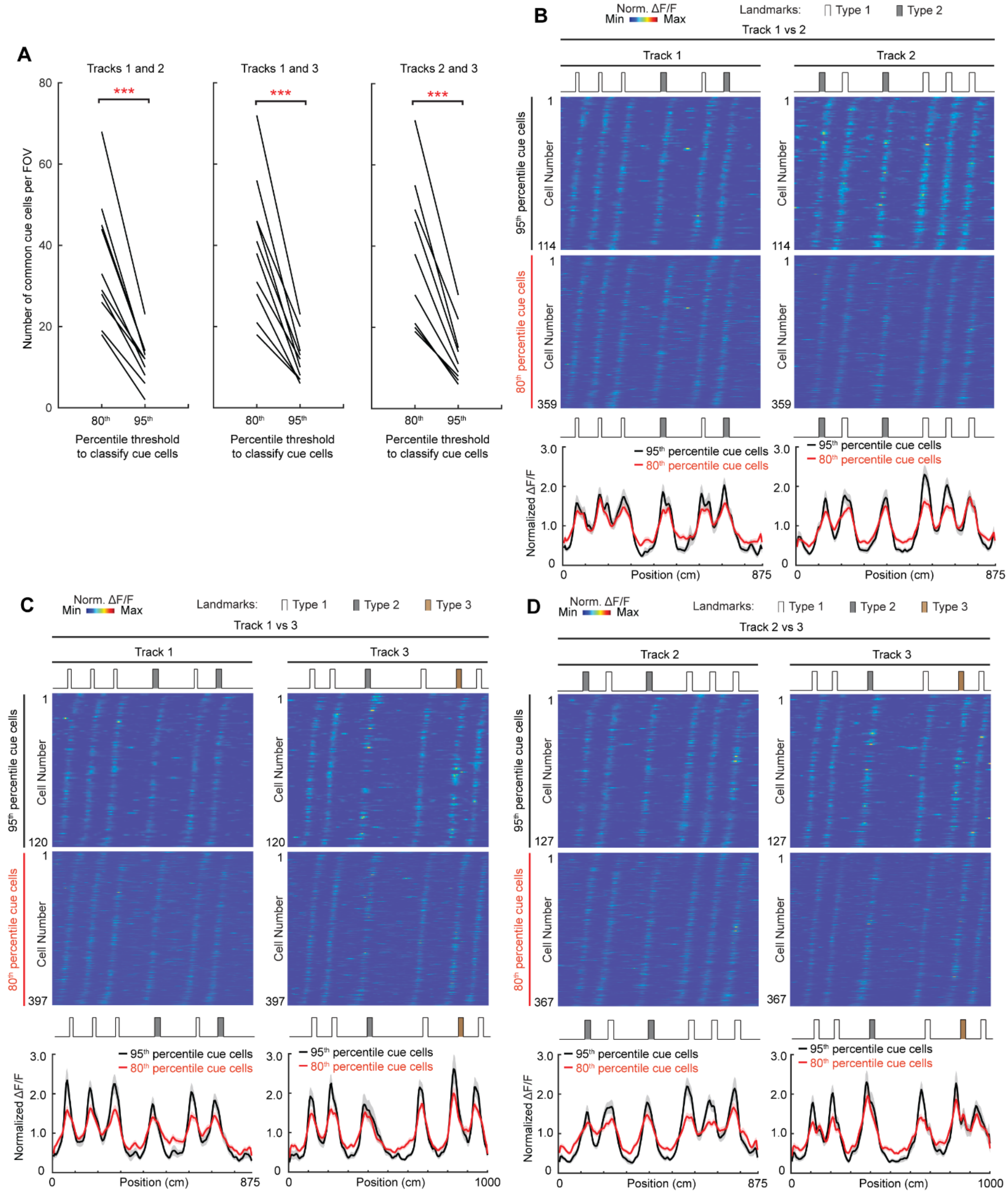

Fig. SR1. Comparison of common cue cells across different tracks, classified using the 80th and 95th percentile thresholds of the shuffled distribution. A. Number of common cue cells across track pairs under the different thresholds. Asterisks indicate the results of two-tailed paired t-tests. Note that the 80th percentile threshold produces more cells. **B.** The activity of common cue cells on tracks 1 and 2 classified using the two

thresholds. Top heat plots: cue cell activity patterns sorted by their spatial shifts in each track. The activity is normalized for each cell by its average activity across all track bins. Bottom: calcium activity of cue cell populations (averaged normalized activity) under the two thresholds. Lines represent mean and shading represents SEM. Note that both thresholds produce cue cells with strongly landmark-associated activity. **C** and **D.** Similar to B, but for tracks 1 and 3 (**C**), as well as 2 and 3 (D).

80th percentile cue threshold data were from 5 animals, including 359, 397, and 367 cells in track comparisons 1 and 2, 1 and 3, as well as 2 and 3, respectively. 95th percentile cue threshold data were from 5 animals, including 114, 120, and 127 cells in track comparisons 1 and 2, 1 and 3, as well as 2 and 3, respectively.

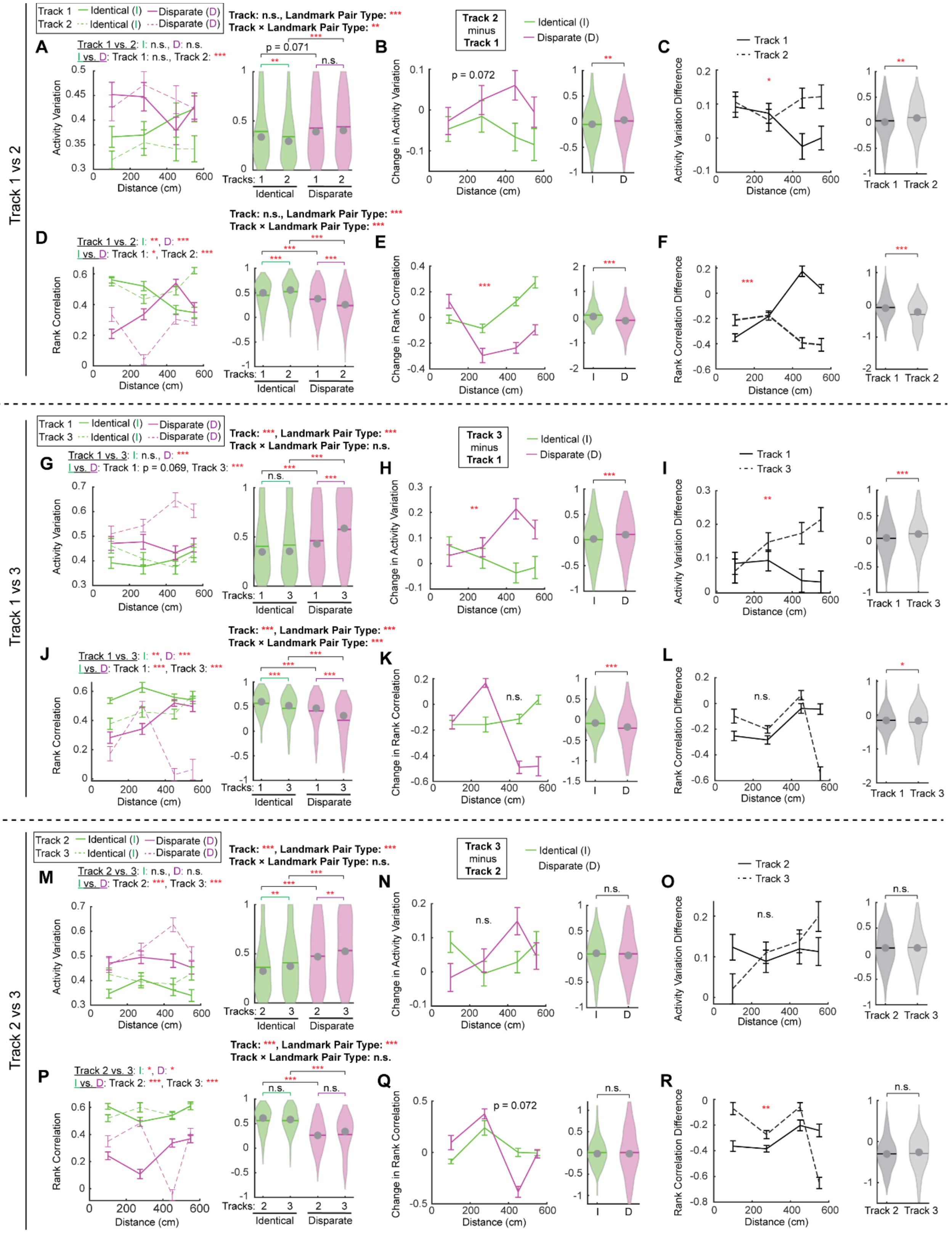

Fig. SR2. 95th percentile cue cells largely show differential landmark identity encoding in different tracks.

A. Left: Activity variation of 95th percentile common cue cells in track 1 (solid line) versus track 2 (dashed line) for identical (I, green) and disparate (D, magenta) landmark pairs across distances. Statistics are two-way ANOVA comparisons with Bonferroni-Holm correction. Right: activity variation calculations for combined distances. Above statistics are two-way ANOVA comparisons. Post-hoc comparisons are Bonferroni-Holm-corrected two-tailed paired t-tests.

B. Track change in activity variation for identical (green) and disparate (magenta) landmark pairs between tracks 1 and 2 at individual (left) and combined (right) distances. Statistical tests are two-way ANOVA (left) and two-tailed paired t-test (right).

C. Similar to B, but for "activity variation difference" for tracks 1 and 2. Statistical tests are the same as those in B.

D-F. Similar to A-C for rank correlations in tracks 1 and 2. Statistical tests are the same as those in A-C. Each cue cell group contains 10 cells.

G-L. Similar to A-F for tracks 1 and 3.

M-R. Similar to A-F for tracks 2 and 3.

Activity variation data were from 5 animals, with 114, 120, and 127 cells for track comparisons 1 and 2, 1 and 3, as well as 2 and 3, respectively. Rank correlation data were from 4 animals (88 cell groups), 3 animals (82 cell groups), and 3 animals (90 cell groups), respectively, for track comparisons 1 and 2, 1 and 3, as well as 2 and 3.

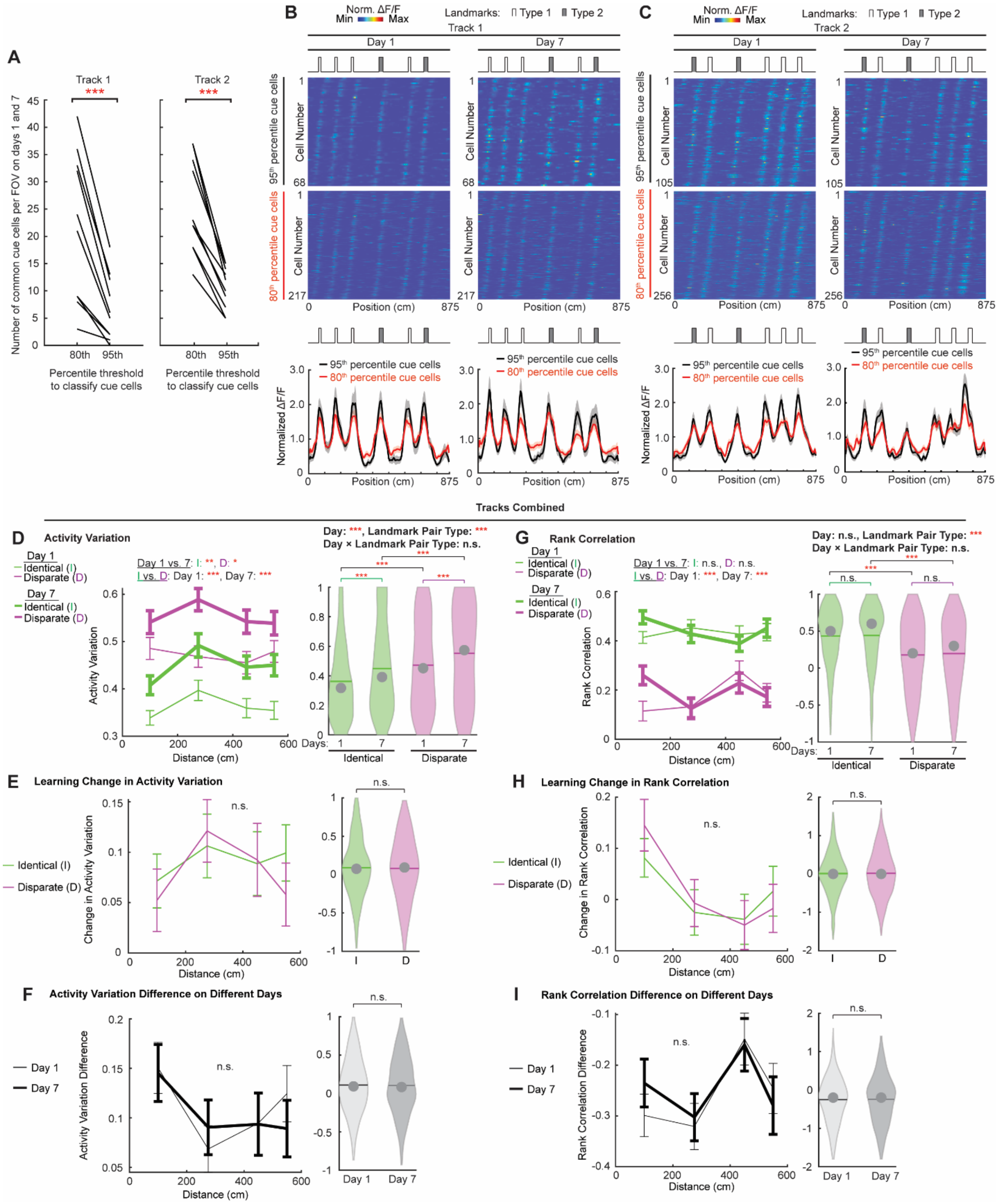

Fig. SR3. The features of cue cells classified using the 80th and 95th percentile thresholds of the shuffled distribution. A. Number of common cue cells across days 1 and 7 of the same tracks under different thresholds. Asterisks indicate the results of two-tailed paired t-tests. Note that 80th percentile threshold produces more cells. **B and C.** The activity of common cue cells on days 1 and 7 of tracks 1 (**B**) and 2 (**C**), classified

using the two thresholds. Top heat plots: cue cell activity patterns sorted by their spatial shifts in each track. The activity is normalized for each cell by its average activity across all track bins. Bottom: calcium activity of cue cell populations (averaged normalized activity) under the two thresholds. Lines represent mean and shading represents SEM. Note that both thresholds produce cue cells with strongly landmark-associated activity. **D.** Left: activity variation of common cue cells on day 1 (dashed lines) vs day 7 (dashed solid lines) for identical (I, green) and disparate (D, magenta) landmark pairs across distances, with data pooled from tracks 1 and 2. Statistics are two-way ANOVA comparisons with Bonferroni-Holm correction. Right: activity variation calculations for combined distances pooled from both tracks. Above statistics are two-way ANOVA comparisons showing that day and landmark pair type modulate activity variation separately, but not together, indicating an experience-independent effect. Post-hoc comparisons are two-tailed paired t-tests with Bonferroni-Holm correction. **E.** Learning change in activity variation for identical (green) and disparate (magenta) landmark pairs on days 1 and 7 for both tracks together at individual (left) as well as combined (right) distances. Statistics are two-way ANOVA (left) and two-tailed paired t-test (right). **F.** Similar to **E**, but for activity variation difference on days 1 and 7. **G-I.** Similar to **D-F** for rank correlations on day 1 and day 7. Statistical tests are the same as those in **D-F**. Each cue cell group contains 5 cells.

80th percentile cue threshold data were from 5 animals, including 217 and 256 cells in tracks 1 and 2, respectively. 95th percentile cue threshold data were from 5 animals, including 68 and 105 cells in tracks 1 and 2, respectively. Track 1 95th percentile rank correlation data were from 4 animals. Track 2 95th percentile rank correlation data were from 5 animals, together producing 153 cue cell groups.

We have included all above information and analyses to the revised manuscript:

(1) In the manuscript, lines 141-143, we clarified that for per session analyses, all cue cells were classified using the 95th percentile of the shuffled distribution.

(2) For common cue cells across tracks, in lines 389-392, and lines 412-414 we added the following sentences:

To increase the statistical power for the following analyses, cue cells in each track were classified using a lower threshold (above the 80th percentile of the shuffled distribution) and common cue cells across tracks were further identified (Fig. S12A). These common cue cells showed strong landmark-associated activity (Fig. S12B-D).

The findings were largely recapitulated when using a more stringent threshold (95th percentile of the shuffled distribution) to classify common cue cells, despite the smaller sample size (Fig. S16).

(3) For common cue cells on days 1 and 7 of the same track, in lines 438-440 and lines 462-464 we added the following sentences:

Common cue cells on the two days, which were similarly classified using an 80th percentile threshold of the shuffled distribution (Fig. S17A), exhibited strong landmark-associated activity (Fig. S17B-C).

The above findings were largely recapitulated when using a more stringent threshold (95th percentile of the shuffled distribution) to classify common cue cells across days, despite the smaller sample size (Fig. S17D-I).

(4) In the “Materials and Methods” section, lines 1638 and 1639, we included the following information for the additional analyses:

To further validate the results, additional analyses were conducted on common cue cells classified using the 95th percentile of shuffles (Figs. S12, 16, and 17).

Reviewer #3:

I am satisfied with the authors' thorough and careful rebuttal, which addresses the previous concerns in detail. The additional analyses, as well as the companion manuscript, greatly strengthen the study. I have no major concerns.

One minor point: the authors state that their new grid cell analyses show grid cells are "spatial and not landmark-driven." Strictly speaking, the analyses demonstrate that grid cells are not landmark-driven; the claim that they are "spatial" is inferred from their known properties, rather than directly shown in these experiments. This is not a major issue but worth noting for precision.

Overall, I find the revisions convincing and the manuscript much improved.

We appreciate the support from the reviewer.

According to the "minor point: we included the following words in the manuscript:

(1) In Results, lines 543-546: Together with previous reports of strong spatial encoding by grid cells[5], these results demonstrate that although both cue and grid cells encode spatial and nonspatial information of landmarks, in the form of their location and identity, respectively, cue cells more robustly encode landmark identity, whereas grid cells primarily represent landmark location (Fig. 7I).
(2) In Discussion, lines 623-625: "This observation suggests that grid cell activity, which primarily carries spatial information based on the current literature[5], can also be modulated by non-spatial visual information."

References

1. Keene, C.S., Bladon, J., McKenzie, S., Liu, C.D., O'Keefe, J., and Eichenbaum, H. (2016). Complementary Functional Organization of Neuronal Activity Patterns in the Perirhinal, Lateral Entorhinal, and Medial Entorhinal Cortices. J Neurosci *36*, 3660-3675. 10.1523/JNEUROSCI.4368-15.2016.
2. Diehl, G.W., Hon, O.J., Leutgeb, S., and Leutgeb, J.K. (2017). Grid and Nongrid Cells in Medial Entorhinal Cortex Represent Spatial Location and Environmental Features with Complementary Coding Schemes. Neuron *94*, 83-92 e86. 10.1016/j.neuron.2017.03.004.
3. Nguyen, D., Wang, G., Wafa, T., Fitzgerald, T., and Gu, Y. (2024). The medial entorhinal cortex encodes multisensory spatial information. Cell Rep *43*, 114813. 10.1016/j.celrep.2024.114813.
4. Kinkhabwala, A.A., Gu, Y., Aronov, D., and Tank, D.W. (2020). Visual cue-related activity of cells in the medial entorhinal cortex during navigation in virtual reality. Elife *9*. 10.7554/eLife.43140.
5. Hafting, T., Fyhn, M., Molden, S., Moser, M.B., and Moser, E.I. (2005). Microstructure of a spatial map in the entorhinal cortex. Nature *436*, 801-806. 10.1038/nature03721.

Figure statistics:

**Figure SR1**	
SR1A	Track 1 and 2: Two-tailed paired t-test: p = 4.6488e-05, n = 10 FOVs<br>Track 1 and 3: Two-tailed paired t-test: p = 4.7637e-05, n = 10 FOVs<br>Track 2 and 3: Two-tailed paired t-test: p = 1.0772e-04, n = 10 FOVs

**Figure SR2**	
SR2A (left)	Two-way ANOVA: Bonferroni-Holm Corrected<br><br>All curves: 114 cells x 4 distances<br><br>**I** Track 1 vs **I** Track 2: p = 0.13603, F = 4.0508<br>D Track 1 vs D Track 2: p = 0.57032, F = 0.3231<br>Track 1 **I** vs D: p = 0.40444, F = 1.6358<br>Track 2 **I** vs D: p = 0.00055003, F = 15.048
SR2A (right)	Two-way ANOVA<br>Track: p = 0.16745, F = 1.9117<br>Landmark Pair Type: p = 2.0393e-08, F = 32.617<br>Track x Landmark Pair Type: p = 0.0066157, F = 7.4434<br><br>Two-tailed paired t-tests: Bonferroni-Holm Corrected<br><br>Each violin contains n = 456 activity variations across all cells and all four matched distances.<br><br>**I** Track 1 vs **I** Track 2: p = 0.0087618<br>D Track 1 vs D Track 2: p = 0.46051<br>Track 1 **I** vs D: p = 0.0711<br>Track 2 **I** vs D: p = 1.0935e-08
SR2B (left)	Two-way ANOVA:<br>Both curves: 114 cells x 4 distances<br>p = 0.072363, F = 3.2593
SR2B (right)	Two-tailed paired t-test: p = 0.0066157, n = 456 changes in activity variation across all cells and all four matched distances for both violins
SR2C (left)	Two-way ANOVA:<br>Both curves: 114 x 4 distances<br>Track 1 vs Track 2: p = 0.020681, F = 5.4302
SR2C (right)	Two-tailed paired t-tests: p = 0.0079011, n = 456 activity variation differences across all cells and all four matched distances for both violins
SR2D (left)	Two-way ANOVA: Bonferroni-Holm Corrected<br>All curves: 88 cell groups x 4 distances<br><br>**I** Track 1 vs **I** Track 2: p = 0.0062256, F = 7.6695

	D Track 1 vs D Track 2: p = 0.00050052, F = 14.809<br>Track 1 I vs D: p = 0.010601, F = 7.9735<br>Track 2 I vs D: p = 6.4903e-16, F = 83.504
SR2D (right)	Two-way ANOVA:<br>Track: p = 0.15954, F = 1.987<br>Landmark Pair Type: p = 5.5591e-30, F = 156.73<br>Track x Landmark Pair Type: p = 1.7066e-12, F = 53.581<br><br>Two-tailed paired t-tests: Bonferroni-Holm Corrected<br><br>Each violin contains n = 352 rank correlations across all cell groups and all four matched distances.<br><br>I Track 1 vs I Track 2: p = 0.00011414<br>D Track 1 vs D Track 2: p = 3.9786e-06<br>Track 1 I vs D: p = 0.00013132<br>Track 2 I vs D: p = 1.8638e-36
SR2E (left)	Two-way ANOVA:<br>Both curves: 88 cell groups x 4 distances<br>p = 8.4896e-06, F = 21.061
SR2E (right)	Two-tailed paired t-test: Each violin contains n = 352 changes in rank correlation across all cell groups and all four matched distances.<br><br>p = 1.7066e-12
SR2F (left)	Two-way ANOVA:<br>Both curves: 88 cell groups x 4 distances<br>Track 1 vs Track 7: p = 1.6713e-08, F = 35.052
SR2F (right)	Two-tailed paired t-tests: p = 2.9258e-11, n = 352 rank correlation differences across all cell groups and all four matched distances for both violins
SR2G (left)	Two-way ANOVA: Bonferroni-Holm Corrected<br><br>All curves: 120 cells x 4 distances<br><br>I Track 1 vs I Track 3: p = 0.94837, F = 0.0042029<br>D Track 1 vs D Track 3: p = 0.00093355, F = 13.419<br>Track 1 I vs D: p = 0.069126, F = 4.5202<br>Track 3 I vs D: p = 1.2007e-07, F = 33.089
SR2G (right)	Two-way ANOVA<br>Track: p = 0.0005448, F = 12.136<br>Landmark Pair Type: p = 5.7382e-17, F = 76.146<br>Track x Landmark Pair Type: p = 8.1014e-06, F = 20.403<br><br>Two-tailed paired t-tests: Bonferroni-Holm Corrected

	Each violin contains n = 480 activity variations across all cells and all four matched distances.<br><br>I Track 1 vs I Track 3: p = 0.63945<br>D Track 1 vs D Track 3: p = 9.2666e-08<br>Track 1 I vs D: p = 0.00075923<br>Track 3 I vs D: p = 6.5324e-18
SR2H (left)	Two-way ANOVA:<br>Both curves: 120 cells x 4 distances<br>p = 0.0041934, F = 8.3832
SR2H (right)	Two-tailed paired t-test: p = 8.1014e-06, n = 480 changes in activity variation across all cells and all four matched distances for both violins
SR2I (left)	Two-way ANOVA:<br>Both curves: 120 cells x 4 distances<br>Track 1 vs Track 3: p = 0.0027233, F = 9.1913
SR2I (right)	Two-tailed paired t-test: p = 3.5808e-05, n = 480 activity variation differences across all cells and all four matched distances for both violins
SR2J (left)	Two-way ANOVA: Bonferroni-Holm Corrected<br>All curves: 82 cell groups x 4 distances<br><br>I Track 1 vs I Track 3: p = 0.00010366, F = 17.65<br>D Track 1 vs D Track 3: p = 7.1303e-05, F = 19.37<br>Track 1 I vs D: p = 5.0011e-07, F = 30.597<br>Track 3 I vs D: p = 0.00078599, F = 12.266
SR2J (right)	Two-way ANOVA:<br>Track: p = 1.139e-15, F = 76.217<br>Landmark Pair Type: p = 3.8216e-22, F = 120.54<br>Track x Landmark Pair Type: p = 0.00073642, F = 11.765<br><br>Two-tailed paired t-tests: Bonferroni-Holm Corrected<br><br>Each violin contains n = 328 rank correlations across all cell groups and all four matched distances.<br><br>I Track 1 vs I Track 3: p = 6.8517e-06<br>D Track 1 vs D Track 3: p = 3.0666e-10<br>Track 1 I vs D: p = 7.317e-14<br>Track 3 I vs D: p = 1.2215e-14
SR2K (left)	Two-way ANOVA:<br>Both curves: 82 cell groups x 4 distances<br>p = 0.82227, F = 0.050819
SR2K (right)	Two-tailed paired t-test: Each violin contains n = 328 changes in rank correlation across all cell groups and all four matched distances.<br><br>p = 0.00073642

SR2L (left)	Two-way ANOVA:<br>Both curves: 82 cell groups x 4 distances<br>Track 1 vs Track 3: p = 0.3625, F = 0.83567
SR2L (right)	Two-tailed paired t-tests: p = 0.011287, n = 328 rank correlation differences across all cell groups and all four matched distances for both violins
SR2M (left)	Two-way ANOVA: Bonferroni-Holm Corrected<br><br>All curves: 127 cells x 4 distances<br><br>I Track 1 vs I Track 3: p = 0.11086, F = 2.5608<br>D Track 1 vs D Track 3: p = 0.16245, F = 3.0658<br>Track 1 I vs D: p = 4.8544e-05, F = 19.339<br>Track 3 I vs D: p = 1.4284e-05, F = 22.564
SR2M (right)	Two-way ANOVA<br>Track: p = 0.00055922, F = 12.07<br>Landmark Pair Type: p = 4.8657e-23, F = 108.6<br>Track x Landmark Pair Type: p = 0.92115, F = 0.0098078<br><br>Two-tailed paired t-tests: Bonferroni-Holm Corrected<br><br>Each violin contains n = 508 activity variations across all cells and all four matched distances.<br><br>I Track 2 vs I Track 3: p = 0.0063519<br>D Track 2 vs D Track 3: p = 0.0055586<br>Track 2 I vs D: p = 7.6963e-11<br>Track 3 I vs D: p = 3.1026e-12
SR2N (left)	Two-way ANOVA:<br>Both curves: 127 cells x 4 distances<br>p = 0.99872, F = 2.56e-06
SR2N (right)	Two-tailed paired t-test: p = 0.92115, n = 508 changes in activity variation across all cells and all four matched distances for both violins
SR2O (left)	Two-way ANOVA:<br>Both curves: 127 cells x 4 distances<br>Track 2 vs Track 3: p = 0.69622, F = 0.1528
SR2O (right)	Two-tailed paired t-tests: p = 0.9987, n = 508 activity variation differences across all cells and all four matched distances for both violins
SR2P (left)	Two-way ANOVA: Bonferroni-Holm Corrected<br>All curves: 90 cell groups x 4 distances<br><br>I Track 2 vs I Track 3: p = 0.01748, F = 7.0785<br>D Track 2 vs D Track 3: p = 0.02974, F = 4.8252<br>Track 2 I vs D: p = 7.6264e-21, F = 118.26<br>Track 3 I vs D: p = 8.3284e-06, F = 24.889

SR2P (right)	Two-way ANOVA:<br>Track: p = 0.00055922, F = 12.07<br>Landmark Pair Type: p = 4.8657e-23, F = 108.6<br>Track x Landmark Pair Type: p = 0.92115, F = 0.0098078<br><br>Two-tailed paired t-tests: Bonferroni-Holm Corrected<br><br>Each violin contains n = 360 rank correlations across all cell groups and all four matched distances.<br><br>I Track 2 vs I Track 3: p = 1<br>D Track 2 vs D Track 3: p = 0.72845<br>Track 2 I vs D: p = 1.6597e-37<br>Track 3 I vs D: p = 5.8143e-18
SR2Q (left)	Two-way ANOVA:<br>Both curves: 90 cell groups x 4 distances<br>p = 0.072363, F = 3.3025
SR2Q (right)	Two-tailed paired t-test: Each violin contains n = 360 changes in rank correlation across all cell groups and all four matched distances.<br><br>p = 0.49479
SR2R (left)	Two-way ANOVA:<br>Both curves: 90 cell groups x 4 distances<br>Track 1 vs Track 7: p = 0.0053847, F = 8.0004
SR2R (right)	Two-tailed paired t-tests: p = 0.4226, n = 360 rank correlation differences across all cell groups and all four matched distances for both violins

**Figure SR3**	
SR3A	Track 1: Two-tailed paired t-test: p = 2.8731e-04, n = 10 FOVs<br>Track 2: Two-tailed paired t-test: p = 1.5393e-05, n = 10 FOVs
SR3D (left)	Two-way ANOVA: Bonferroni-Holm Corrected<br><br>All curves: 173 cells x 4 distances<br><br>I Day 1 vs I Day 7: p = 0.0042486, F = 9.5913<br>D Day 1 vs D Day 7: p = 0.01162, F = 6.4399<br>Day 1 I vs D: p = 1. 1.5562e-05, F = 22.038<br>Day 7 I vs D: p = 1.3424e-05, F = 21.797
SR3D (right)	Two-way ANOVA<br>Day: p = 1.6665e-12, F = 51.834<br>Landmark Pair Type: p = 5.9026e-24, F = 110.35<br>Day x Landmark Pair Type: p = 0.72544, F = 0.12344<br><br>Two-tailed paired t-tests: Bonferroni-Holm Corrected

	Each violin contains n = 692 activity variations across all cells and all four matched distances.<br><br>I Day 1 vs I Day 7: p = 2.1084e-09<br>D Day 1 vs D Day 7: p = 7.7034e-07<br>Day 1 I vs D: p = 2.6593e-14<br>Day 7 I vs D: p = 5.3802e-12
SR3E (left)	Two-way ANOVA:<br>Both curves: 173 cells x 4 distances<br>p = 0.87276, F = 0.025692
SR3E (right)	Two-tailed paired t-test: p = 0.72544, n = 692 changes in activity variation across all cells and all four matched distances for both violins
SR3F (left)	Two-way ANOVA:<br>Both curves: 173 cells x 4 distances<br>Day 1 vs Day 7: p = 0.7696, F = 0.085931
SR3F (right)	Two-tailed paired t-tests: p = 0.72544, n = 692 activity variation differences across all cells and all four matched distances for both violins
SR3G (left)	Two-way ANOVA: Bonferroni-Holm Corrected<br>All curves: 153 cell groups x 4 distances<br><br>I Day 1 vs I Day 2: p = 0.75717, F = 0.095776<br>D Day 1 vs D Day 2: p = 1, F = 0.19312<br>Day 1 I vs D: p = 8.8641e-12, F = 53.596<br>Day 7 I vs D: p = 2.7563e-11, F = 50.326
SR3G (right)	Two-way ANOVA:<br>Day: p = 0.4329, F = 0.61586<br>Landmark Pair Type: p = 1.0574e-36, F = 183.25<br>Day x Landmark Pair Type: p = 0.77457, F = 0.082099<br><br>Two-tailed paired t-tests: Bonferroni-Holm Corrected<br><br>Each violin contains n = 612 rank correlations across all cell groups and all four matched distances.<br><br>I Day 1 vs I Day 7: p = 0.70781<br>D Day 1 vs D Day 7: p = 0.91955<br>Day 1 I vs D: p = 4.2229e-24<br>Day 7 I vs D: p = 5.3678e-20
SR3H (left)	Two-way ANOVA:<br>Both curves: 153 cell groups x 4 distances<br>p = 0.83261, F = 0.044747
SR3H (right)	Two-tailed paired t-test: Each violin contains n = 612 changes in rank correlation across all cell groups and all four matched distances.

	p = 0.77457
SR3I (left)	Two-way ANOVA:<br>Both curves: 153 cell groups x 4 distances<br>Day 1 vs Day 7: p = 0.83356, F = 0.044235
SR3I (right)	Two-tailed paired t-tests: p = 0.77457, n = 612 rank correlation differences across all cell groups and all four matched distances for both violins

Black: Reviewer

Blue: Authors

REVIEWERS' COMMENTS

Reviewer #1 (Remarks to the Author):

I thank the authors for the additional discussion. I have no further comments.

We appreciate the reviewer's support and critical feedback through this revision.
